# Substituting polyunsaturated fat for saturated fat: A health impact assessment of a fat tax in seven European countries

**DOI:** 10.1371/journal.pone.0218464

**Published:** 2019-07-10

**Authors:** Johanna-Katharina Schönbach, Wilma Nusselder, Stefan K. Lhachimi

**Affiliations:** 1 Institute for Public Health and Nursing Research, University of Bremen, Bremen, Germany; 2 Research Group for Evidence-Based Public Health, Leibniz Institute for Prevention Research and Epidemiology–BIPS, Bremen, Germany; 3 Department of Public Health, Erasmus University Medical Center, Rotterdam, The Netherlands; University of Mississippi Medical Center, UNITED STATES

## Abstract

There is evidence that replacing saturated fat (SFA) with polyunsaturated fat (PUFA) lowers ischemic heart disease (IHD). In order to improve the population’s diet, the World Health Organization has called for the taxation of foods that are high in SFA. We aimed to assess the potential health gains of a European fat tax by applying the SFA intake reduction that has been observed under the Danish fat tax to six other European countries. For each country, we created a fat tax scenario with a decreased SFA intake and a corresponding increase in PUFA. We compared this fat tax scenario to a reference scenario with no change in SFA intake, and to a guideline scenario with a population-wide SFA intake in line with dietary recommendations. We used DYNAMO-HIA to dynamically project the policy-attributable IHD cases of these three scenarios 10 years into the future. A fat tax would reduce prevalent IHD cases by a minimum of 500 and 300 among males and females in Denmark, respectively, up to a maximum of 5,600 and 4,000 among males and females in the UK. Thereby, the prevented IHD cases under a fat tax scenario would correspond to between 11.0% (in females in the Netherlands) and 29.5% (in females in Italy) of the prevented IHD cases under a guideline scenario, which represents the maximum preventable disease burden. Henceforth, our quantification of beneficial health impacts makes the case for the policy debate on fat taxes.

## Introduction

Cardiovascular disease (CVD), with its main forms ischemic heart disease (IHD) and stroke, is the leading cause of mortality in Europe. With 3.9 million deaths a year, it corresponds to 45% of all deaths [1]. Current evidence accentuates the role of modifying dietary fat intake for the prevention of CVD [2–12]. A recent Cochrane review suggests that replacing foods that are rich in saturated fat (SFA), such as meat, butter, and cheese, with foods that are rich in polyunsaturated fat (PUFA), such as walnuts, fish, and vegetable oils such as sunflower and safflower oils, would lead to 27% less cardiovascular events [8]. The Global Burden of Disease (GBD) Study 2016 estimated that 36,900 IHD deaths in Western Europe could be attributed to diets low in PUFA [13]. There are a number of studies estimating the potential health benefits that a SFA intake reduction would have [14–20] (see S1 Table).

Even though it is recommended to replace SFA with PUFA, and to consume ≤10 percent of one’s total energy intake (%E) from SFA [21], the majority of the European population exceeds the SFA recommendation [22–24]. Therefore, the World Health Organization calls for the taxation of unhealthy foods, such as those high in SFA, to improve the population’s diet [25, 26]. Reviews based on modelling studies suggest that taxes on unhealthy foods are effective in improving dietary behaviour and have the potential for improving health [27–30]. A range of studies exist that model (hypothetical) taxes on SFA and their potential health impacts [31–37] (illustrated in S1 Table).

Meanwhile, the evidence of a fat tax induced SFA intake change from a real-life setting is available. In October 2011, Denmark was the first country in the world that introduced a tax on meat, dairy products, animal fat, oils, margarine and butter blends with more than 2.3g of SFA per 100g. These products were taxed with 16 DKK (€2.15; $2.84) per kg of SFA [38]. Due to their high content of SFA, butter and margarine were products most affected by the tax, with a price increase over 20% [39]. Since an increase in border trade was anticipated, the tax was abolished again in January 2013, before the health impact of the tax had been evaluated [40]. Household scanner data indicated an average tax-induced SFA intake reduction of 4%. It was modelled that this would translate into 123 fewer deaths annually in Denmark [41].

This study aims to enlarge the body of evidence by modelling the health impacts of a fat tax in seven European countries, using the SFA intake reduction that has been observed under the Danish fat tax.

## Materials and methods

### Policy proposal

**Fig 1:**
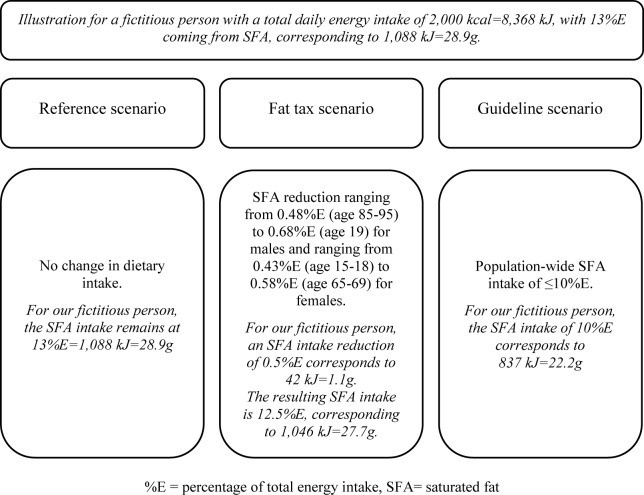
Illustration of reference, fat tax and guideline scenario.

Apart from a fat tax scenario, we modelled a reference and a guideline scenario for comparison purposes. The change in SFA intake for a fictitious person is illustrated in Fig 1. In the reference scenario, SFA intake in the respective countries remained as currently observed. Thus, there was no change in dietary intake. For the fat tax scenario, we assumed the tax would have the same effect that it had in Denmark. Therefore, we derived the age- and sex-specific SFA consumption change that was observed in Denmark after the introduction of a fat tax [41], corresponding to a SFA intake reduction ranging from 0.48%E to 0.68%E for males and ranging from 0.43%E to 0.58%E for females (see S2 Table for detailed calculations). For the guideline scenario, a population-wide SFA intake of ≤10%E was adopted, as recommended by the Food and Agriculture Organization of the United Nations [21]. This scenario represented the maximum effect that a ‘perfect’ policy could have.

### SFA intake

SFA intake by country was drawn from the European Nutrition and Health Report [42], which reports age-group-specific mean and standard deviation (SD) of SFA intake in %E for European countries. We included only those countries where the mean and the SD of SFA intake for at least four age groups were available (because a minimum of four points is required to later smooth intake over age).

For the reference scenario, we smoothed the original age-group-related mean intake of SFA over age. To predict the fat-tax scenario’s age-specific mean intake of SFA, we multiplied the reference scenario’s age-specific mean intake by the age-specific SFA consumption change that was observed in Denmark after the introduction of a fat tax (S2 Table).

For the guideline scenario, all individuals were assumed to have a SFA intake of ≤10%E.

**Fig 2:**
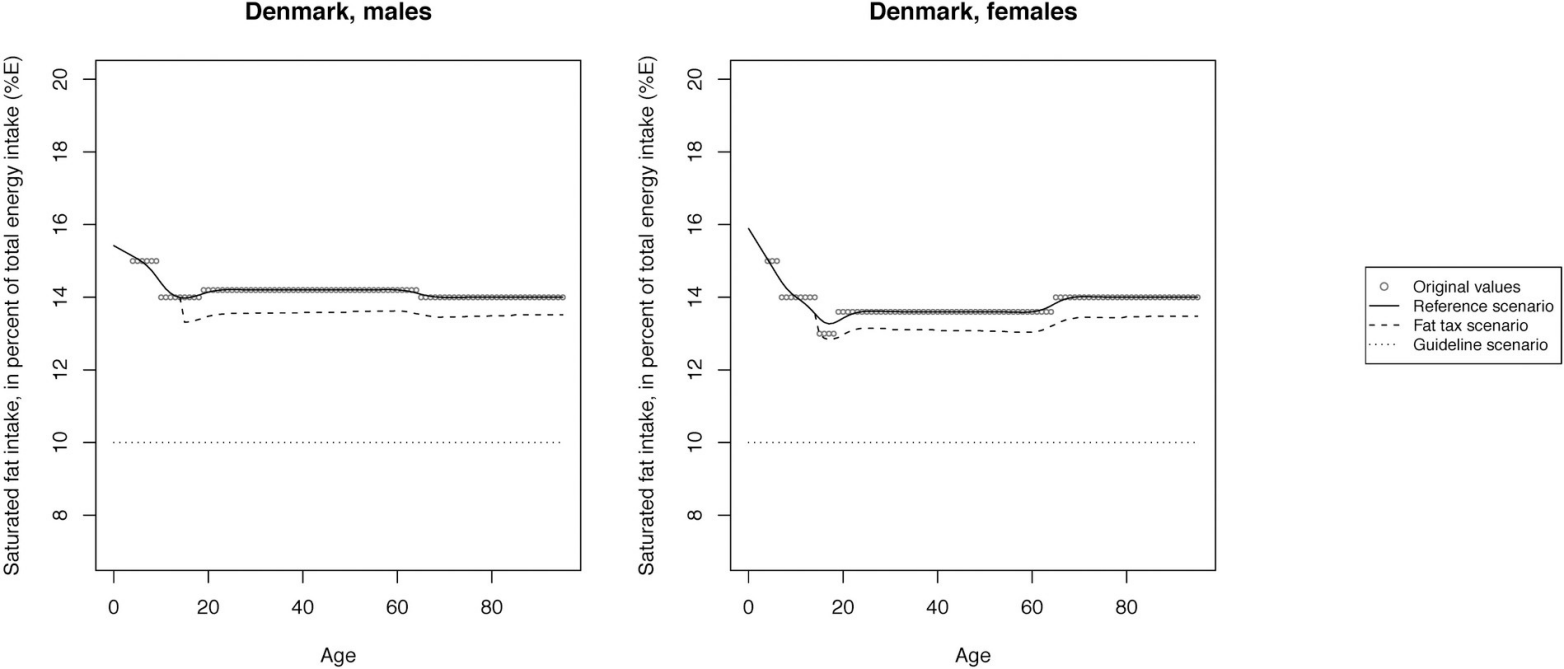
Mean saturated fat intake across reference, fat tax and guideline scenario in Denmark.

**Fig 3:**
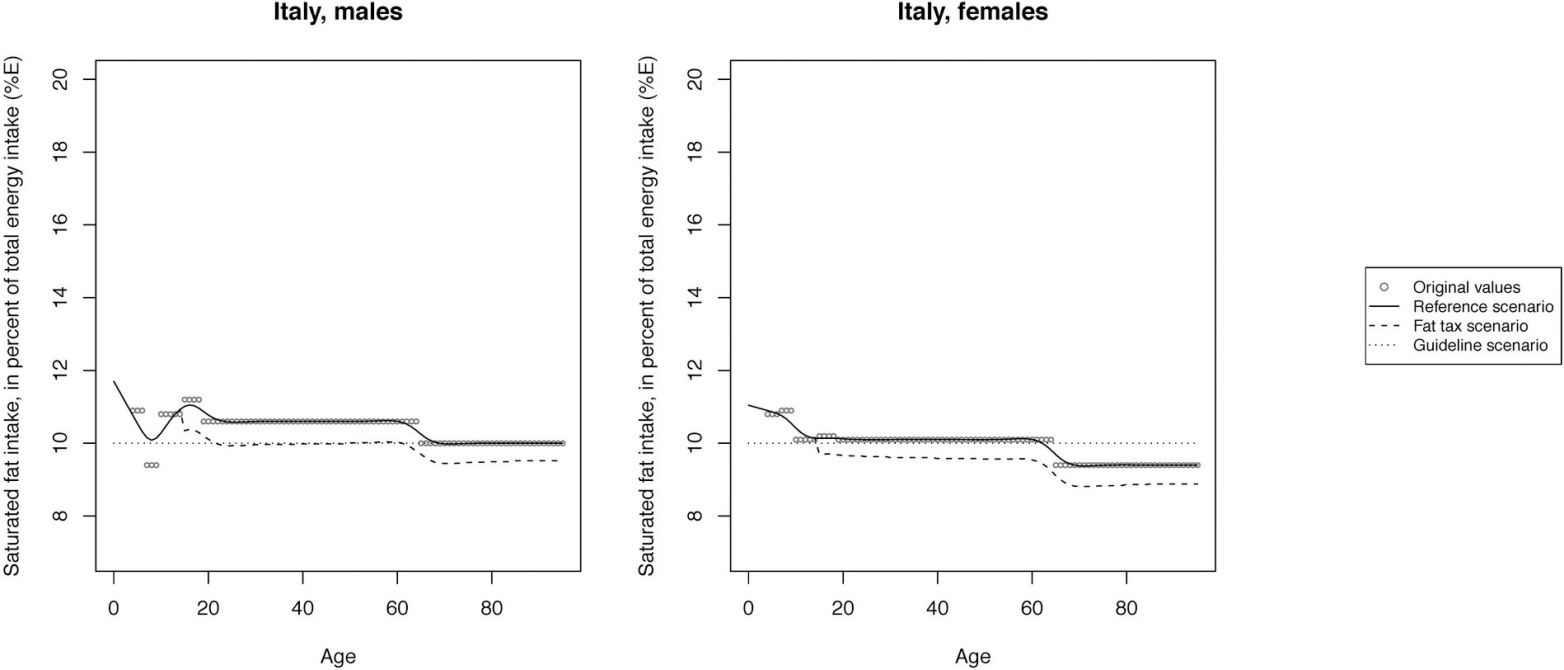
Mean saturated fat intake across reference, fat tax and guideline scenario in Italy.

**Fig 4:**
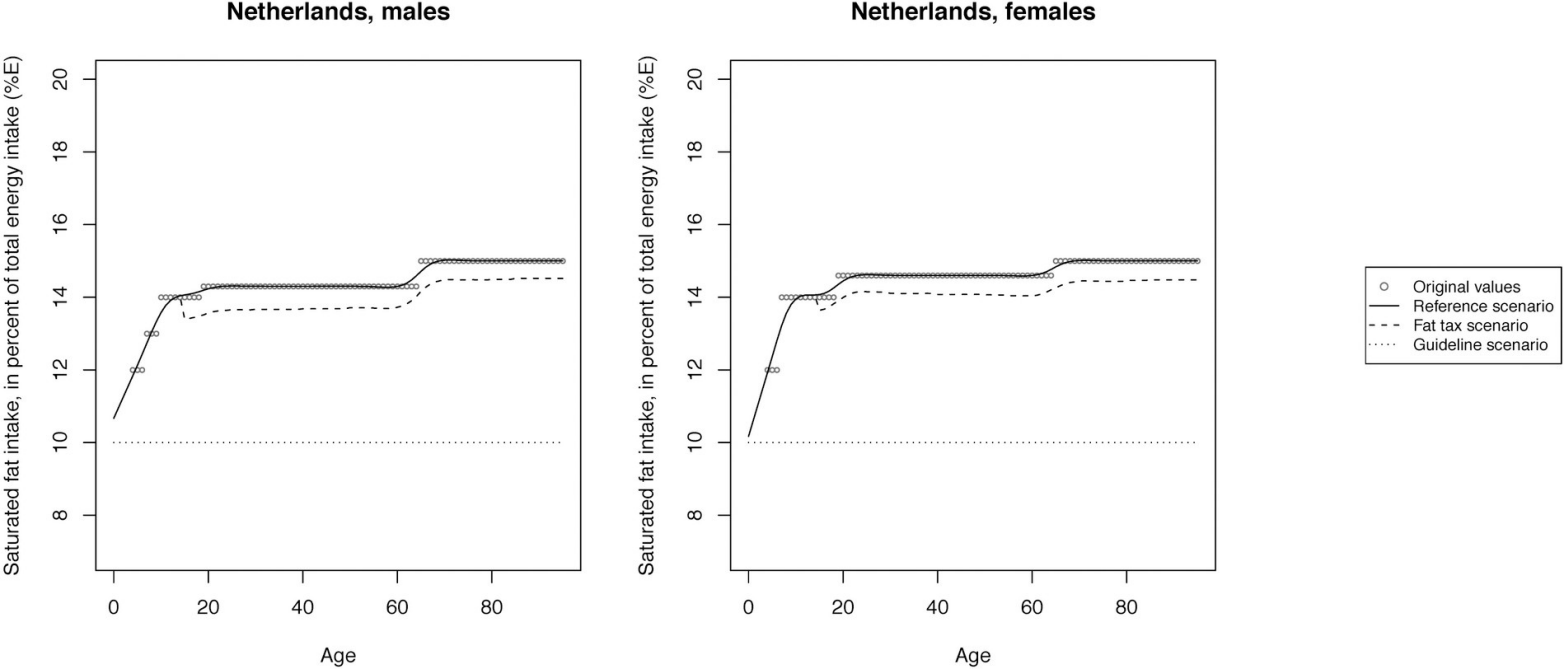
Mean saturated fat intake across reference, fat tax and guideline scenario in the Netherlands.

**Fig 5:**
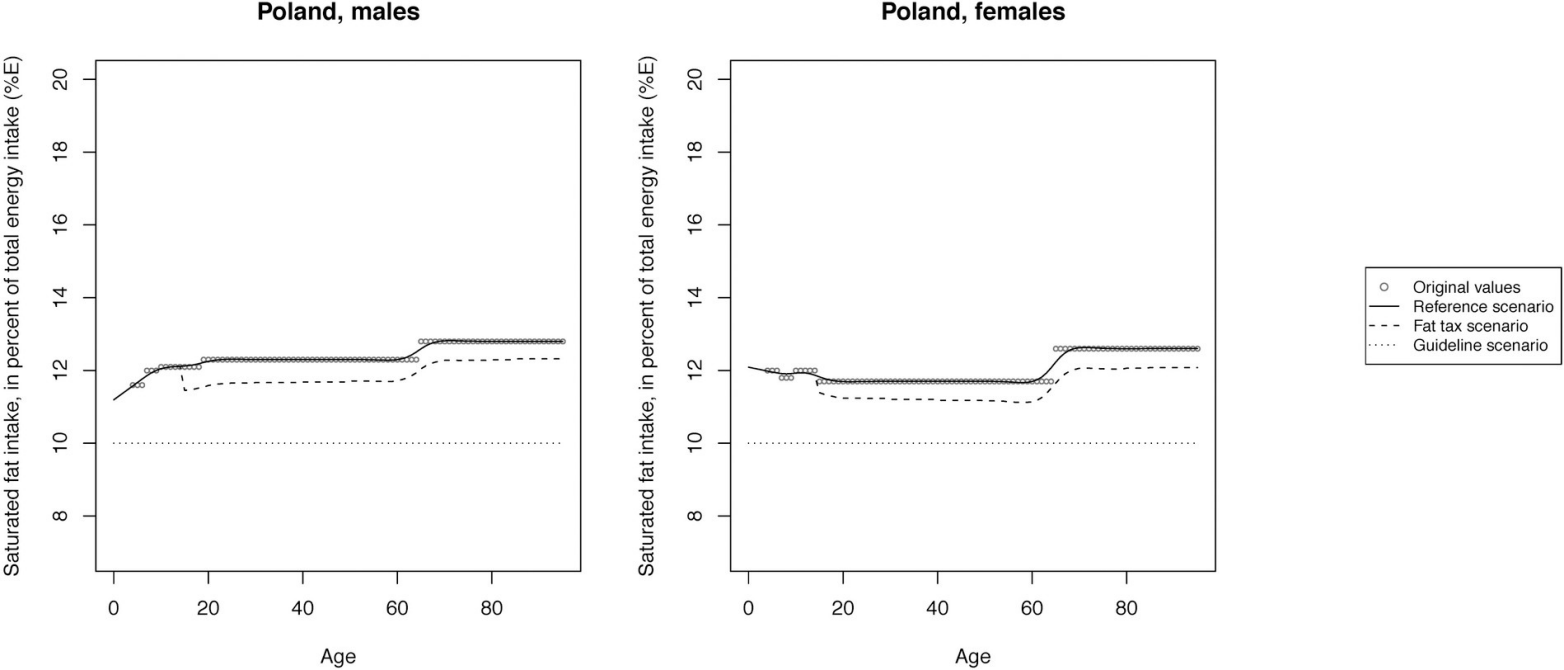
Mean saturated fat intake across reference, fat tax and guideline scenario in Poland.

**Fig 6:**
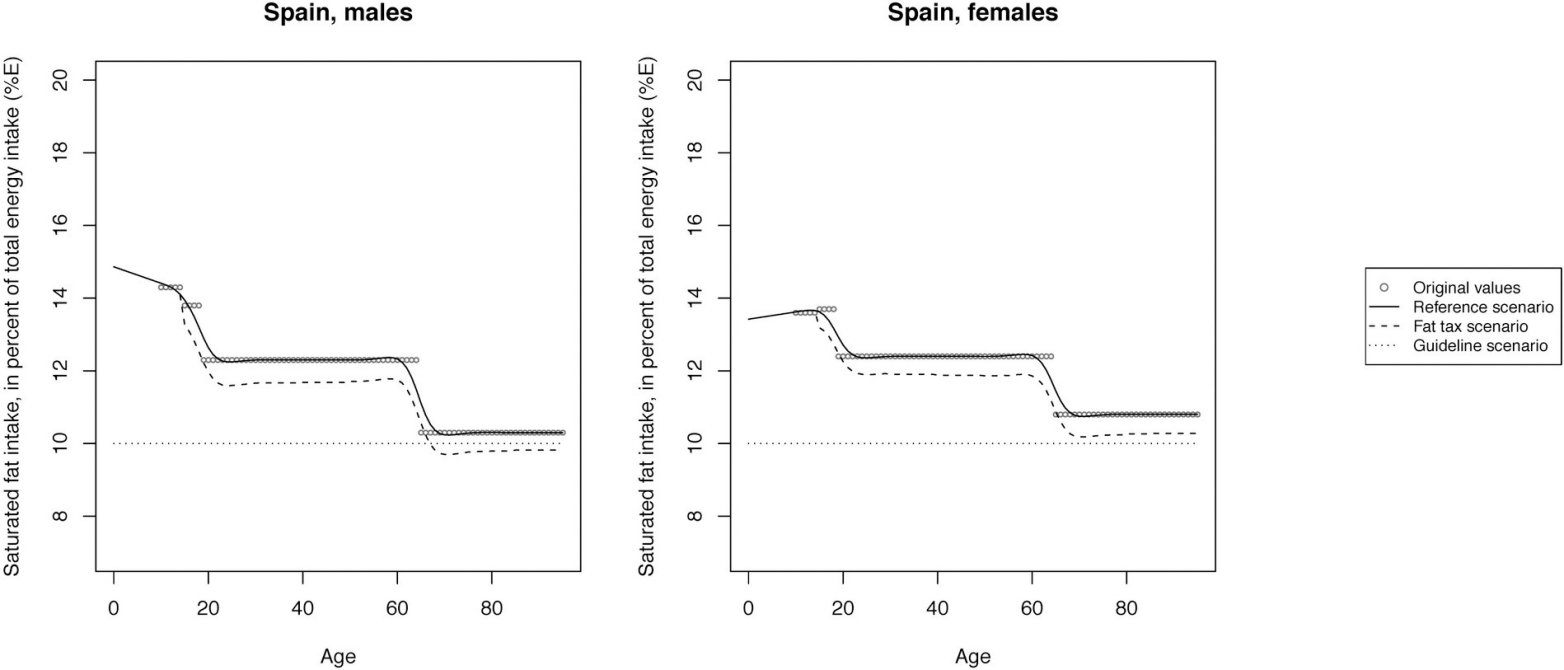
Mean saturated fat intake across reference, fat tax and guideline scenario in Spain.

**Fig 7:**
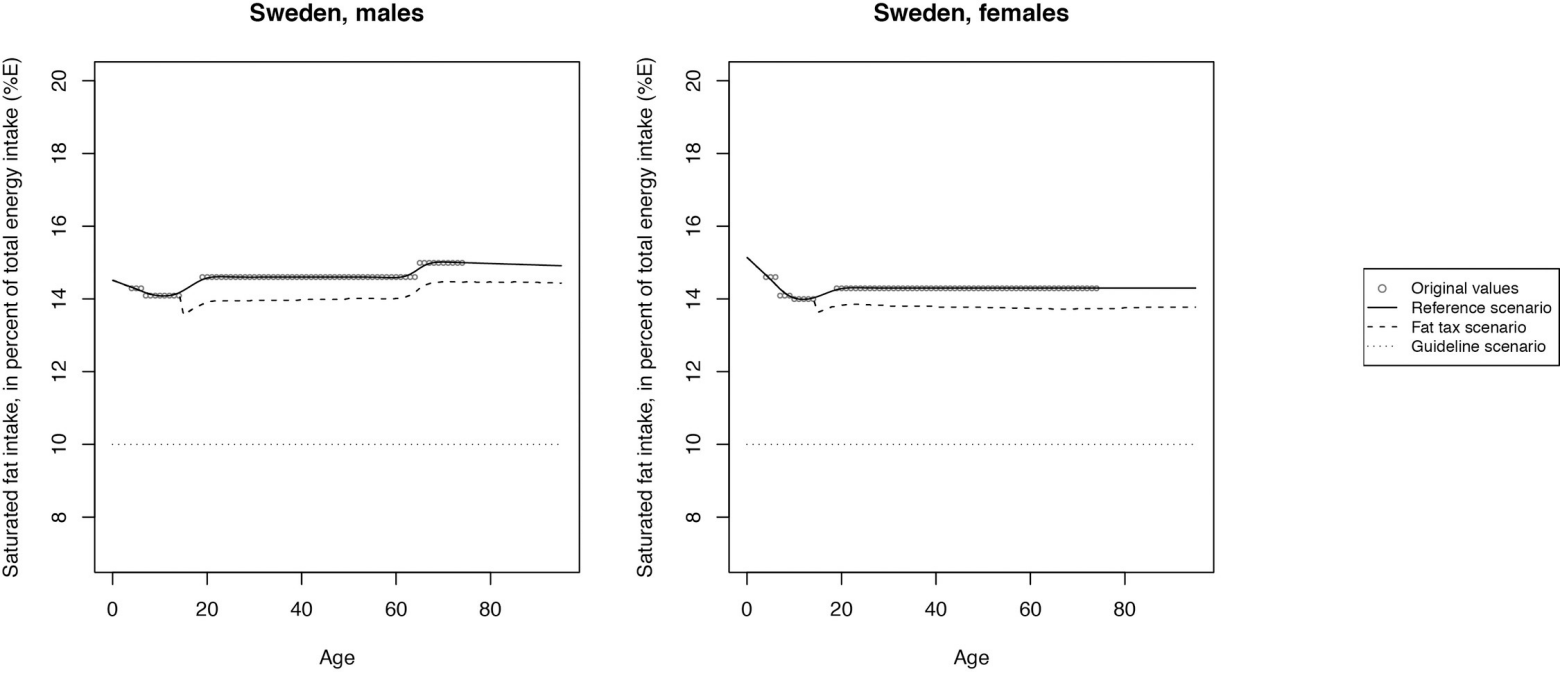
Mean saturated fat intake across reference, fat tax and guideline scenario in Sweden.

**Fig 8:**
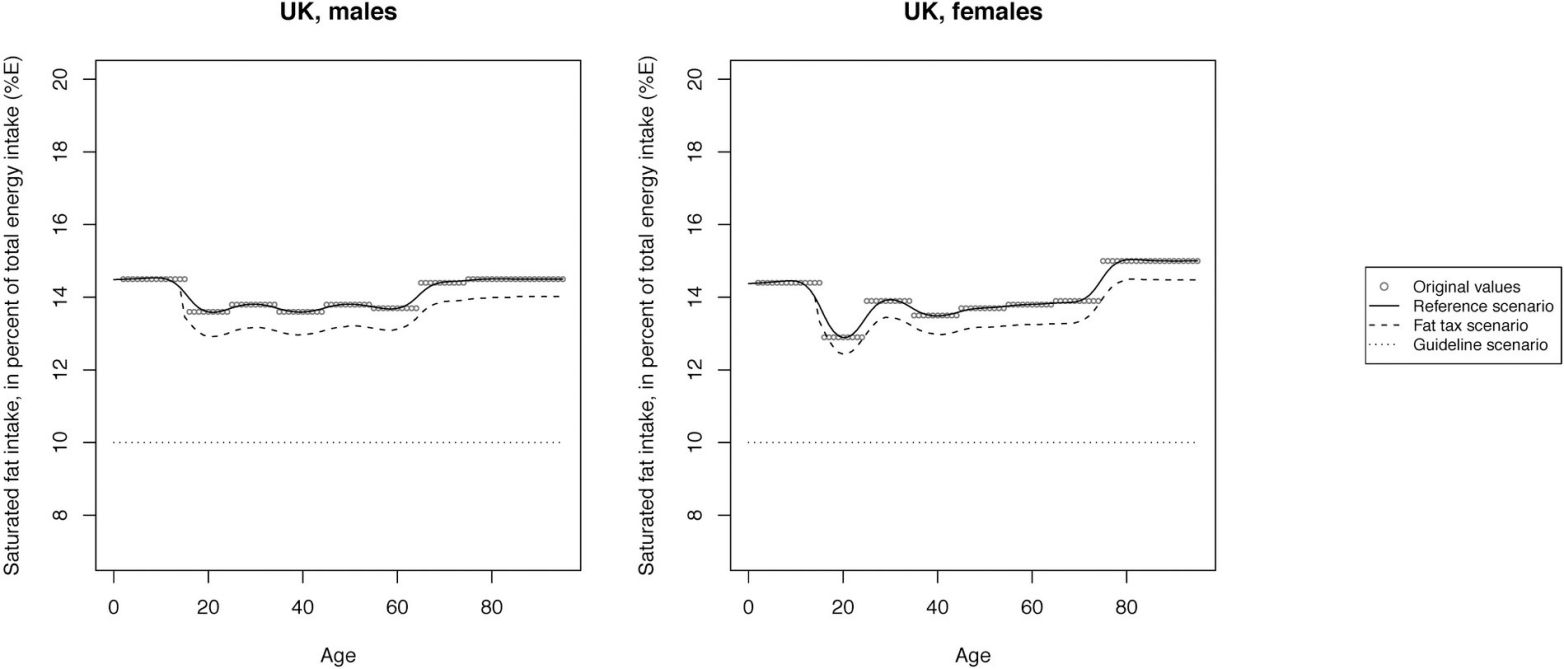
Mean saturated fat intake across reference, fat tax and guideline scenario in the UK.

The mean SFA intake across scenarios is illustrated in Figs 2–8 and presented with corresponding SDs in tabular form in the S3–S9 Tables.

For the reference and fat tax scenario, we presumed a normal distribution of SFA intake [23, 43], and obtained the proportion of persons for the following 10 intake categories, for each age and sex: ≤10%E, >10 ≤12%E, >12 ≤14%E, >14 ≤16%E, >16 ≤18%E, >18 ≤20%E, >20 ≤22%E, >22 ≤24%E, >24 ≤26%E and >26 ≤100%E. For the guideline scenario, all individuals were shifted towards the intake category of ≤10%E SFA. Individuals who were already in the intake category of ≤10%E SFA remained in this category. The shift of SFA intake across the different scenarios, for males and females, respectively, is tabulated by age and sex in the S10–S16 Tables.

We used net transitions, i.e. we set the age-specific SFA intake to be constant for each scenario, so that the age-specific SFA intake in the population does not change over the projection period.

### Relative risks

The relative risks of ischemic heart disease for each 5%E of lowered PUFA intake, being replaced with SFA, were taken from the GBD Study 2016 [13]. We transformed these relative risks as described in S1 Text, in order to obtain the relative risk matching our SFA intake categories (≤10%E, >10 ≤12%E, >12 ≤14%E, >14 ≤16%E, >16 ≤18%E, >18 ≤20%E, >20 ≤22%E, >22 ≤24%E, >24 ≤26%E, >26 ≤100%E). The original and transformed relative risks are illustrated in Table 1.

**Table 1:** Relative risks of ischemic heart disease associated with saturated fat intake.

Age	Original relative risks^a^	Our relative risks^b^									
	5%E increment	≤≤10%E	>10 ≤12%E	>12 ≤14%E	>14 ≤16%E	>16 ≤18%E	>18 ≤20%E	>20 ≤22%E	>22 ≤24%E	>24 ≤26%E	>26 ≤100%E
0–24	1.000	1.00	1.00	1.00	1.00	1.00	1.000	1.00	1.00	1.00	1.00
25–29	1.267	1.00	1.05	1.15	1.27	1.39	1.53	1.68	1.85	2.03	2.24
30–34	1.211	1.00	1.04	1.12	1.21	1.31	1.41	1.52	1.65	1.78	1.92
35–39	1.148	1.00	1.03	1.09	1.15	1.21	1.28	1.35	1.43	1.51	1.60
40–44	1.114	1.00	1.02	1.07	1.11	1.16	1.21	1.27	1.32	1.38	1.44
45–49	1.111	1.00	1.02	1.07	1.11	1.16	1.21	1.26	1.31	1.37	1.43
50–54	1.101	1.00	1.02	1.06	1.10	1.14	1.19	1.24	1.28	1.33	1.39
55–59	1.086	1.00	1.02	1.05	1.09	1.12	1.16	1.20	1.24	1.28	1.32
60–64	1.075	1.00	1.01	1.04	1.08	1.11	1.14	1.17	1.21	1.24	1.28
65–69	1.068	1.00	1.01	1.04	1.07	1.10	1.13	1.16	1.19	1.22	1.25
70–74	1.063	1.00	1.01	1.04	1.06	1.09	1.12	1.14	1.17	1.20	1.23
75–79	1.060	1.00	1.01	1.04	1.06	1.08	1.11	1.14	1.16	1.19	1.22
80–84	1.063	1.00	1.01	1.04	1.06	1.09	1.12	1.14	1.17	1.20	1.23
85–89	1.063	1.00	1.01	1.04	1.06	1.09	1.12	1.14	1.17	1.20	1.23
90–94	1.063	1.00	1.01	1.04	1.06	1.09	1.12	1.14	1.17	1.20	1.23
95+	1.063	1.00	1.01	1.04	1.06	1.09	1.12	1.14	1.17	1.20	1.23

^a^ Original relative risks (for each 5 percent of total energy intake per day of decreased polyunsaturated fat replaced with saturated fat) were derived from the Global Burden of Disease (GBD) Study 2016 [13]

^b^ Rounded to two decimal points

### Epidemiological data on the population and IHD

We derived data on the population size, age-composition, projected births and mortality, as well as data on the IHD incidence, prevalence and excess mortality of Denmark, Italy, the Netherlands, Poland, Spain, Sweden and the UK from the DYNAMO-HIA database [44]. The data is available from the project’s website and accompanied by reports detailing how data was compiled (https://www.dynamo-hia.eu).

### Dynamic modelling

We modeled the three scenarios (reference scenario, fat tax scenario, and guideline scenario) 10 years into the future, using DYNAMO-HIA. DYNAMO-HIA is a dynamic health impact assessment tool, which compares the effects of intervention scenarios with a changed risk factor exposure to a reference scenario with unchanged risk factor exposure. Therefore, it projects a real-life population through risk factor exposure, in this case SFA intake, and associated diseases [44–46]. Full technical details of DYNAMO-HIA are available elsewhere [44–46].

## Results

The cumulative number of prevalent IHD cases in the reference scenario, the fat tax scenario as well as the guideline scenario in projection year 10 are shown in Table 2.

**Table 2:** Number of prevalent ischemic heart disease cases and prevalence in projection year 10.

Country^a^	Scenario	Males				Females			
		Prevalent cases^b^	Prevalence	Difference to reference scenario	% of max. preventable burden^c^	Prevalent cases^b^	Prevalence	Difference to reference scenario	% of max. preventable burden^c^
DK	Reference scenario	86,600	3.20%	-	-	64,800	2.35%	-	-
	Fat tax scenario	86,100	3.18%	-500	15.63%	64,500	2.34%	-300	14.29%
	Guideline scenario	83,400	3.08%	-3,200	100.00%	62,700	2.27%	-2,100	100.00%
IT	Reference scenario	979,900	3.55%	-	-	850,000	2.91%	-	-
	Fat tax scenario	977,100	3.54%	-2,800	26.42%	848,200	2.90%	-1,800	29.51%
	Guideline scenario	969,300	3.51%	-10,600	100.00%	843,900	2.88%	-6,100	100.00%
PL	Reference scenario	627,900	3.47%	-	-	633,400	3.24%	-	-
	Fat tax scenario	625,000	3.45%	-2,900	15.51%	631,100	3.22%	-2,300	15.03%
	Guideline scenario	609,200	3.37%	-18,700	100.00%	618,100	3.16%	-15,300	100.00%
ES	Reference scenario	490,800	2.36%	-	-	427,000	1.97%	-	-
	Fat tax scenario	488,900	2.36%	-1,900	24.36%	425,600	1.97%	-1,400	24.56%
	Guideline scenario	483,000	2.33%	-7,800	100.00%	421,300	1.95%	-5,700	100.00%
SE	Reference scenario	216,900	4.75%	-	-	163,900	3.54%	-	-
	Fat tax scenario	215,900	4.73%	-1,000	12.35%	163,200	3.53%	-700	13.21%
	Guideline scenario	208,800	4.57%	-8,100	100.00%	158,600	3.43%	-5,300	100.00%
NL	Reference scenario	344,300	4.15%	-	-	253,300	3.00%	-	-
	Fat tax scenario	342,700	4.13%	-1,600	11.51%	252,200	2.98%	-1,100	11.00%
	Guideline scenario	330,400	3.98%	-13,900	100.00%	243,300	2.88%	-10,000	100.00%
UK	Reference scenario	1,343,500	4.50%	-	-	1,026,100	3.31%	-	-
	Fat tax scenario	1,337,900	4.48%	-5,600	12.17%	1,022,100	3.30%	-4,000	11.53%
	Guideline scenario	1,297,500	4.34%	-46,000	100.00%	991,400	3.20%	-34,700	100.00%

^a^ DK = Denmark, IT = Italy, PL = Poland, ES = Spain, SE = Sweden, NL = The Netherlands, UK = United Kingdom

^b^ Rounded to the nearest hundred

^c^ Prevented cases in fat tax scenario measured against prevented cases in guideline scenario

In the reference scenario, with no change in dietary intake, prevalent IHD cases would range from an estimated 86,600 among males and 64,800 among females in Denmark, followed by Sweden, the Netherlands, Spain, Poland and Italy, to an estimated 1,343,500 male and 1,026,100 female prevalent IHD cases in the UK.

Compared to the reference scenario, the reduction of prevalent IHD cases under the fat tax scenario would range from 500 among males and 300 among females in Denmark, respectively, followed by Sweden, the Netherlands, Spain, Italy, and Poland, to 5,600 and 4,000 less prevalent IHD cases in males and females in the UK, respectively.

Under the guideline scenario, which represents the maximum preventable disease burden, prevalent IHD cases would be reduced by a minimum of 3,200 and 2,100 among males and females in Denmark, respectively, followed by Sweden and Spain, Italy, the Netherlands, and Poland, up to 46,000 and 34,700 fewer prevalent IHD cases in the UK.

Measured against the guideline scenario, this reduction in IHD cases under the fat tax scenario ranges from 11.5% and 11.0% among males and females in the Netherlands, followed by the UK, Sweden, Denmark and Poland, and Spain up to 26.4% and 29.5% among males and females in Italy.

## Discussion

### Discussion of main results

This is the first study that has applied the SFA intake reduction observed under the Danish fat tax to six other European countries in order to project the potential health impacts.

Our results suggest that a fat tax would reduce prevalent IHD cases by a minimum of 500 and 300 among males and females in Denmark, respectively, up to a maximum of 5,600 and 4,000 among males and females in the UK. In most countries, a fat tax would reach between 11.0% (in females in the Netherlands) and 15.6% (in males in Denmark) of the prevented IHD cases under the guideline scenario, whereby the latter represents the maximum preventable disease burden. A fat tax in Spain and Italy, however, would reach 24.4% (in males in Spain) to 29.5% (in females in Italy) of the maximum preventable disease burden. These results for Spain and Italy can be explained by their relatively low initial SFA intake, especially in higher age groups. In these countries, SFA intake under the fat tax comes very close to the SFA intake under the guideline scenario.

### Comparison to other modelling studies

In our simulation, a fat tax would lead to 104 more persons (65 males and 39 females) being alive in Denmark in projection year 10 (S17 Table). In contrast, the econometric and comparative risk assessment evaluation–from which we derived the age- and sex-specific SFA consumption change–estimated that the change in fat intake observed under the Danish fat tax would translate to 36 saved lives annually. Moreover, the saved lives would total 123, if changes in fruit and vegetable, as well as salt and fibre intake were also considered [41].

Our guideline scenario would lead to 812 more persons (511 males and 301 females) being alive in Denmark in projection year 10 (S17 Table). In comparison to that, the GBD Study 2016 –from which we derived our relative risks–estimated that 400 IHD deaths annually were attributable to low PUFA intake in Denmark [13].

Thus, both scenarios in our simulation seem to underestimate results. However, the number of postponed deaths increases steadily over the projection period in our simulation (S1 and S2 Files), and might therefore differ from static estimates.

In general, the comparability between studies modelling the health impacts from changes in SFA intake is complicated for several reasons. Firstly, modelling studies use different policy scenarios. Whereas we presume an intake reduction in line with the Danish fat tax, where products were taxed with 16 DKK per kg of SFA [41], others model SFA to be taxed with $1.37 per 100g of SFA [37], a value-added tax (VAT) change [31, 32, 34], a price increase by 1% for every percent of SFA [33, 35], a 20% tax on major dietary sources of SFA [36], or no explicit policy [14–20]. Secondly, modelling studies use different conceptual pathways. Studies model a pathway via cholesterol [14–17], or–like us–base calculations on relative risks reflecting a direct link between SFA and IHD [20]. Thirdly, studies use different outcomes. While we model prevalent IHD cases, other studies report Disability-Adjusted Life Years (DALYs) [37] or deaths [34, 36]. Different assumptions and data between modelling studies of SFA taxes have previously been discussed [29].

### Limitations

Several limitations of this health impact assessment must be acknowledged.

First of all, we assumed that the SFA intake change observed under the fat tax in Denmark would occur in other countries as well. To the best of our knowledge, we have no evidence on the contrary, but the precise dietary changes will vary, depending on what food groups contribute to SFA intake in the respective country. Across the different cohorts of the European Prospective Investigation into Cancer and Nutrition (EPIC) study, dairy products, meat and meat products, as well as fats and oils (consisting of vegetable oils, butter and margarine) were main contributors to SFA intake, but the extent differed across EPIC cohorts [24]. For instance, in the Danish Copenhagen cohort, vegetable oils, butter and margarine contributed to SFA intake with 28% in males and 23% in females. In other EPIC cohorts, this food group contributed to SFA intake from 13% in the female Spanish Asturias cohort to 38% in the male German Potsdam cohort. When further differentiating the food group of fats and oils, the biggest contribution to SFA intake in the cohorts of Denmark, France, Germany, the Netherlands, the UK, and Sweden was made by butter and margarine. In the cohorts of Greece, Spain and Italy, in contrast, the biggest contribution to SFA intake within this food group was made by vegetable oils [24]. Vegetable oils, however, are also rich in mono–and polyunsaturated fat. As an example, olive oil, despite containing roughly 20% SFA, contains approximately 70% MUFA [47] and is associated with a lower all-cause-mortality and cardiovascular events [48]. Whereas vegetable oils were among the taxed products in Denmark, this could have adverse health effects in countries where vegetable oils greatly contribute to SFA intake. Thus, a tax must be carefully designed and take into account dietary specific patterns as well as the ratio of SFA to MUFA and PUFA of taxed products.

The second limitation stems from the SFA intake data. SFA intake was drawn from the European Nutrition and Health Report [42], which was released in 2009. The authors of the report stated that the comparability of data was limited because of the use of different methods, such as 24-hour recalls or Food Frequency Questionnaires, different years and periods of data collection, and different age classifications. In the report, SFA intake was only provided by age group, which we then smoothed over age in order to obtain age-specific SFA intake. Nevertheless, this data seemed to be the most recent overview on SFA intake in countries of the European Union.

Similarly, the third restriction refers to the epidemiological data on the population and IHD. We used the DYNAMO-HIA database, which is the only publicly available database for quantitative health impact assessment with homogenous data collection methods. The database was made publicly available in 2011 and contains trend-free incidence, prevalence and mortality data. Nevertheless, the impact of trend-free data on our results is negligible. Our aim was to compare reference, fat tax and guideline scenario with each other, and we used the same disease data in all scenarios [49].

The fourth constraint stems from the relative risks we used. We used the GBD Study 2016’s relative risks that were applied to 195 countries and territories [13]. To the best of our knowledge, this is the only source which provided relative risks by age groups. The GBD provides these relative risk estimates as applicable to all countries. However, it must be acknowledged that across countries risk modifiers may exist, an area of research that seems to be understudied. In line with the GBD Study 2016 [13] and other literature [11], we modelled health impacts only on IHD (and not stroke or CVD events overall) even though the previously mentioned Cochrane review concluded that replacing SFA with PUFA would significantly lower CVD events overall [8]. A recent meta-analysis, which based their findings on randomised controlled trials, concluded that replacing SFA with PUFA would have no effect on major IHD events [50]. Nevertheless, it is argued that prospective cohort studies find the replacement of SFA with PUFA to be beneficial for the prevention of IHD [51], and the majority of reviews and meta-analyses accentuates the role of modifying dietary fat intake for the prevention of CVD [2–12]. It was found that replacing each 1%E of SFA with PUFA reduces total cholesterol (TC) by 0.064 mmol/L, high-density lipoprotein cholesterol (HDL-C) by 0.005 mmol/L, low-density lipoprotein cholesterol (LDL-C) by 0.055 mmol/L, triglycerides by 0.010 mmol/L and the TC:HDL-C ratio by 0.034 mmol/L [52]. LDL-C in particular is an established risk factor for IHD [53, 54].

Fifthly, we conservatively presumed no further health benefit below the recommended SFA intake goal of 10%E [21]. However, there is indication that further decreasing SFA intake may have additional benefits on the overall serum lipoprotein profile [52], in which case our fat tax and guideline scenario would underestimate the respective health benefits.

Finally, a fundamental assumption in our study was that PUFA intake would increase accordingly. This assumption is in line with the dietary recommendations that SFA should be replaced with PUFA [21, 53], and other modelling studies that have assumed the SFA reduction would be replaced by PUFA [14, 16, 17, 20] (see S1 Table). A SFA decrease without a simultaneous increase in PUFA does not seem to be beneficial for health [55, 56]. In Denmark, the decreased SFA intake (ranging from 1.6% in males above age 85 to 4.9% in females aged 55–69) seems to have been accompanied by a changed PUFA intake ranging from a 3.1% decrease in males above age 85 to a 0.3% increase in females aged 60–74 [41]. Therefore, our assumption may lead to an overestimation of the health benefits. Foods contain a mix of SFA, MUFA and PUFA. Therefore, further research needs to examine how this corresponding substitution of PUFA can be assured, rather than a replacement by MUFA or carbohydrates. For instance, a price increase for products high in SFA might need to be coupled with a corresponding price decrease for products high in PUFA.

In conclusion, this study quantifies the magnitude of health improvements under a tax on SFA in European countries and has found a small to medium beneficial impact on health. We hope that our quantification of health impacts can further inform the policy debate on fat taxes.

## Supporting information

S1 Table Overview of studies modelling A) counterfactual saturated fat intake or B) fiscal policies on saturated fat and resulting health impacts.
(DOCX)

S2 Table Calculation of fat tax scenario. (DOCX)

S3 Table Saturated fat intake (mean and standard deviation) across scenarios in Denmark. (DOCX)

S4 Table Saturated fat intake (mean and standard deviation) across scenarios in Italy. (DOCX)

S5 Table Saturated fat intake (mean and standard deviation) across scenarios in the Netherlands. (DOCX)

S6 Table Saturated fat intake (mean and standard deviation) across scenarios in Poland. (DOCX)

S7 Table Saturated fat intake (mean and standard deviation) across scenarios in Spain. (DOCX)

S8 Table Saturated fat intake (mean and standard deviation) across scenarios in Sweden. (DOCX)

S9 Table Saturated fat intake (mean and standard deviation) across scenarios in the UK. (DOCX)

S10 Table Proportion of persons in the respective saturated fat intake categories across scenarios in Denmark. (DOCX)

S11 Table Proportion of persons in the respective saturated fat intake categories across scenarios in Italy. (DOCX)

S12 Table Proportion of persons in the respective saturated fat intake categories across scenarios in the Netherlands. (DOCX)

S13 Table Proportion of persons in the respective saturated fat intake categories across scenarios in Poland. (DOCX)

S14 Table Proportion of persons in the respective saturated fat intake categories across scenarios in Spain. (DOCX)

S15 Table Proportion of persons in the respective saturated fat intake categories across scenarios in Sweden. (DOCX)

S16 Table Proportion of persons in the respective saturated fat intake across scenarios in the UK. (DOCX)

S17 Table Population size and deaths postponed in projection year 10. (DOCX)

S1 Text Calculating relative risks of ischemic heart disease by intake category of saturated fat. (DOCX)

S1 File Excess number of persons being alive in Denmark under the fat tax scenario compared to reference scenario. (DOCX)

S2 File Excess number of persons being alive in Denmark under the guideline scenario compared to reference scenario. (DOCX)
