# Supplementary material for: Substituting polyunsaturated fat for saturated fat: A health impact assessment of a fat tax in seven European countries
Source: PLoS One. 2019 Jul 10;14(7):e0218464. doi: 10.1371/journal.pone.0218464 (PMC6619676; doi:10.1371/journal.pone.0218464)
Supplement: S1 Table — Overview of studies modelling A) counterfactual saturated fat intake or B) fiscal policies on saturated fat and resulting health impacts. (DOCX) [file pone.0218464.s001.docx]

S1 Table. Overview of studies modelling A) counterfactual saturated fat intake or B) fiscal policies on saturated fat and resulting health impacts.

| Author | Year | Country | Policy / Tax scheme | Impact on nutrient intake | Health outcome |
| --- | --- | --- | --- | --- | --- |
| **A) Studies modelling counterfactual saturated fat intake (without defining policy or intervention)** | | | | | |
| Lloyd-Williams et al. [1] | 2008 | 15 European Countries | - | SFA intake reduction of 2.2g = 1%E, replaced with 0.5%E MUFA and 0.5%E PUFA | 9,800 fewer CHD deaths and 3,000 fewer stroke deaths each year |
| Martikainen et al. [2] | 2011 | Finland | - | Replacing 1%E of SFA intake with PUFA | 8,000 to 13,000 prevented CVD cases over 20 years |
| O’Flaherty et al. [3] | 2012 | UK | - | a) Reducing SFA intake by 1%E, with 90% of this SFA reduction replaced by PUFA and 10% by MUFA  b) Reducing SFA intake by 3%E, with 90% of this SFA reduction replaced by PUFA and 10% by MUFA | a) 4,000 fewer deaths by 2015 (baseline year: 2006)  b) 11,200 fewer deaths by 2015 (baseline year: 2006) |
| O’Keeffe et al. [4] | 2013 | Ireland | - | a) Reducing SFA intake by 1%E  b) Reducing SFA intake by 3%E | a) 89 fewer CVD deaths per year  b) 264 fewer CVD deaths per year |
| Bjorck et al. [5] | 2016 | Sweden | - | a) Daily SFA intake decreasing to 10%E, with 90% of this SFA reduction replaced by PUFA and 10% by MUFA  b) Daily SFA intake rising to 20%E, with 90% of this SFA reduction replaced by PUFA and 10% by MUFA | a) 430 prevented or postponed CHD deaths in 2025 (after projection period of 15 years)  b) 400 additional CHD deaths in 2025 (after projection period of 15 years) |
| O’Flaherty et al. [6] | 2016 | 9 European Countries | - | a) Replacing 1%E of SFA intake with 0.1%E MUFA and 0.9%E PUFA, reducing smoking by 5%, salt by 10%, physical activity by 5%  b) Replacing 2%E of SFA intake with 0.2%E MUFA and 1.8%E PUFA, reducing smoking by 10%, salt by 20%, physical activity by 10%  c) Replacing 3%E of SFA intake with 0.3%E MUFA and 2.7%E PUFA, reducing smoking by 15%, salt by 30%, physical activity by 15% | a) 6,900 prevented or postponed deaths in 2020 (baseline: 2010–11)  b) 13,240 deaths prevented or postponed deaths in 2020 (baseline year: 2010–11)  c) 18,670 deaths prevented or postponed deaths in 2020 (baseline year: 2010–11) |
| Sahan et al. [7] | 2016 | Turkey | - | a) Reducing SFA intake from 12%E to 11%E  b) Reducing SFA intake from 12%E to 10%E | a) 6,410 fewer CHD deaths in 2025  b) 13,160 fewer CHD deaths in 2025 |
| **B) Studies modelling fiscal policies on saturated fat*** | | | | | |
| Marshall [8] | 2000 | UK | Extending VAT at 17.5% to the main sources of SFA | Reducing SFA intake by 0.67%E | 1.8% to 2.6% IHD reduction, prevention of 1,800 to 2,500 deaths a year |
| Mytton et al. [9] | 2007 | UK | Extending VAT to main sources of SFA products (whole milk, cheese, butter, cakes, pastries; based on Marshall 2000) | Reducing SFA intake by 0.13%E | 1.3% to 2.0% IHD increase, 1.5% to 1.7% stroke increase, increase in CVD deaths from 2,500 to 3,500 |
| Arnoult et al. [10] | 2008 | UK | Price increase by 1% for every percent of SFA, simultaneous subsidy for fruits and vegetables | Reducing SFA intake from 14.49%E to 13.83%E | Drop in the prevalence risk of IHD (‑4.3%), cancer (‑2.7%) and major chronic diseases (‑1.3%); increase in the risk of type 2 diabetes (+1.3%) (due to the fall in PUFA intake) |
| Nnoaham et al [11] | 2009 | UK | Extending VAT at 17.5% to major sources of SFA in the diet | 2.4% reduction of SFA | Increase of 1,100 to 2,300 deaths per year |
| Tiffin & Arnoult [12] | 2011 | UK | Increase price by 1% for every percent of SFA, plus subsidy on fruits and vegetables | N/A | Before tax: Odds of being affected by IHD due to SFA are 78% higher than they would be if everyone ate in accordance with the dietary guidelines. After tax: 72% higher |
| Ni Mhurchu et al. [13] | 2015 | New Zealand | 20% tax on major dietary sources of SFA | 5.83% reduction in SFA | 1,500 (950 to 2,100) deaths prevented or postponed |
| Cobiac et al. [14] | 2017 | Australia | Taxing products with >2.3% of SFA with $1.37 per 100 g of SFA | N/A | 97,000 (77,000 to 120,000) DALYs averted |

SFA = Saturated fat, PUFA = Polyunsaturated fat, MUFA = Monounsaturated fat, VAT = Value‑added tax, DALYs = Disability‑adjusted life years, CVD = Cardiovascular diseases, N/A = Not available, %E = Percent of total energy

* This part was adapted from Mytton et al. [15].

**References**

1. Lloyd-Williams F, O'Flaherty M, Mwatsama M, Birt C, Ireland R, Capewell S. Estimating the cardiovascular mortality burden attributable to the European Common Agricultural Policy on dietary saturated fats. Bull World Health Organ. 2008;86(7):535-41. doi: 10.2471/blt.08.053728. PMID: 18670665.

2. Martikainen JA, Soini EJ, Laaksonen DE, Niskanen L. Health economic consequences of reducing salt intake and replacing saturated fat with polyunsaturated fat in the adult Finnish population: estimates based on the FINRISK and FINDIET studies. Eur J Clin Nutr. 2011;65(10):1148-55. doi: 10.1038/ejcn.2011.78. PMID: 21587284.

3. O'Flaherty M, Flores-Mateo G, Nnoaham K, Lloyd-Williams F, Capewell S. Potential cardiovascular mortality reductions with stricter food policies in the United Kingdom of Great Britain and Northern Ireland. Bull World Health Organ. 2012;90(7):522-31. doi: 10.2471/blt.11.092643. PMID: 22807598.

4. O'Keeffe C, Kabir Z, O'Flaherty M, Walton J, Capewell S, Perry IJ. Modelling the impact of specific food policy options on coronary heart disease and stroke deaths in Ireland. BMJ Open. 2013;3(7). doi: 10.1136/bmjopen-2013-002837. PMID: 23824313.

5. Björck L, Rosengren A, Winkvist A, Capewell S, Adiels M, Bandosz P, et al. Changes in Dietary Fat Intake and Projections for Coronary Heart Disease Mortality in Sweden: A Simulation Study. PLoS One. 2016;11(8):e0160474. doi: 10.1371/journal.pone.0160474. PMID: 27490257.

6. O'Flaherty M, Bandosz P, Critchley J, Capewell S, Guzman-Castillo M, Aspelund T, et al. Exploring potential mortality reductions in 9 European countries by improving diet and lifestyle: A modelling approach. Int J Cardiol. 2016;207:286-91. doi: 10.1016/j.ijcard.2016.01.147. PMID: 26812643.

7. Sahan C, Sozmen K, Unal B, O'Flaherty M, Critchley J. Potential benefits of healthy food and lifestyle policies for reducing coronary heart disease mortality in Turkish adults by 2025: a modelling study. BMJ Open. 2016;6(7):e011217. doi: 10.1136/bmjopen-2016-011217. PMID: 27388358.

8. Marshall T. Exploring a fiscal food policy: the case of diet and ischaemic heart disease. BMJ. 2000;320(7230):301-5. PMID: 10650031.

9. Mytton O, Gray A, Rayner M, Rutter H. Could targeted food taxes improve health? J Epidemiol Community Health. 2007;61(8):689-94. doi: 10.1136/jech.2006.047746. PMID: 17630367.

10. Arnoult MH, Tiffin R, Traill WB. Models of nutrient demand, tax policy and public health impact. Reading: University of Reading, 2008.

11. Nnoaham KE, Sacks G, Rayner M, Mytton O, Gray A. Modelling income group differences in the health and economic impacts of targeted food taxes and subsidies. Int J Epidemiol. 2009;38(5):1324-33. doi: 10.1093/ije/dyp214. PMID: 19483200.

12. Tiffin R, Arnoult M. The public health impacts of a fat tax. Eur J Clin Nutr. 2011;65(4):427-33. doi: 10.1038/ejcn.2010.281. PMID: 21245880.

13. Ni Mhurchu C, Eyles H, Genc M, Scarborough P, Rayner M, Mizdrak A, et al. Effects of Health-Related Food Taxes and Subsidies on Mortality from Diet-Related Disease in New Zealand: An Econometric-Epidemiologic Modelling Study. PLoS One. 2015;10(7):e0128477. doi: 10.1371/journal.pone.0128477. PMID: 26154289.

14. Cobiac LJ, Tam K, Veerman L, Blakely T. Taxes and Subsidies for Improving Diet and Population Health in Australia: A Cost-Effectiveness Modelling Study. PLoS Med. 2017;14(2):e1002232. doi: 10.1371/journal.pmed.1002232. PMID: 28196089.

15. Mytton OT, Clarke D, Rayner M. Taxing unhealthy food and drinks to improve health. BMJ. 2012;344:e2931. doi: 10.1136/bmj.e2931. PMID: 22589522.
