# Supplementary material for: Substituting polyunsaturated fat for saturated fat: A health impact assessment of a fat tax in seven European countries
Source: PLoS One. 2019 Jul 10;14(7):e0218464. doi: 10.1371/journal.pone.0218464 (PMC6619676; doi:10.1371/journal.pone.0218464)
Supplement: S2 Table — (DOCX) [file pone.0218464.s002.docx]

# S2 Table. Calculation of fat tax scenario.

| Age | Before fat tax | | | | Change | | After fat tax | | | SFA change in %E |
| --- | --- | --- | --- | --- | --- | --- | --- | --- | --- | --- |
|  | Total energy (MJ)^a^ | Total energy (kcal)^b^ | SFA (%E)^c^ | SFA (kcal)^d^ | Energy (kcal, %)^e^ | SFA (kcal, %)^f^ | Total energy (kcal)^g^ | SFA (kcal)^h^ | SFA (%E)^i^ |  |
| Males | | | | | | | | | | |
| 4–6 | 7.8 | 1863 | 15.00 | 279 | N/A | N/A | N/A | N/A | N/A | N/A |
| 7–9 | 9.3 | 2221 | 15.00 | 333 | N/A | N/A | N/A | N/A | N/A | N/A |
| 10–14 | 9.6 | 2293 | 14.00 | 321 | N/A | N/A | N/A | N/A | N/A | N/A |
| 15–18 | 10.3 | 2460 | 14.00 | 344 | 0.40 | -4.40 | 2470 | 329 | 13.33 | -0.67 |
| 19 | 10.6 | 2532 | 14.20 | 360 | 0.40 | -4.40 | 2542 | 344 | 13.52 | -0.68 |
| 20–24 | 10.6 | 2532 | 14.20 | 360 | 0.40 | -4.30 | 2542 | 344 | 13.54 | -0.66 |
| 25–29 | 10.6 | 2532 | 14.20 | 360 | 0.60 | -4.00 | 2547 | 345 | 13.55 | -0.65 |
| 30–34 | 10.6 | 2532 | 14.20 | 360 | 0.80 | -3.70 | 2552 | 346 | 13.57 | -0.63 |
| 35–39 | 10.6 | 2532 | 14.20 | 360 | 1.00 | -3.50 | 2557 | 347 | 13.57 | -0.63 |
| 40–44 | 10.6 | 2532 | 14.20 | 360 | 1.10 | -3.30 | 2560 | 348 | 13.58 | -0.62 |
| 45–49 | 10.6 | 2532 | 14.20 | 360 | 1.30 | -3.10 | 2565 | 348 | 13.58 | -0.62 |
| 50–54 | 10.6 | 2532 | 14.20 | 360 | 1.40 | -2.80 | 2567 | 349 | 13.61 | -0.59 |
| 55–59 | 10.6 | 2532 | 14.20 | 360 | 1.60 | -2.60 | 2572 | 350 | 13.61 | -0.59 |
| 60–64 | 10.6 | 2532 | 14.20 | 360 | 1.70 | -2.40 | 2575 | 351 | 13.63 | -0.57 |
| 65–69 | 9.7 | 2317 | 14 | 324 | 1.80 | -2.20 | 2359 | 317 | 13.45 | -0.55 |
| 70–74 | 9.7 | 2317 | 14 | 324 | 1.90 | -2.00 | 2361 | 318 | 13.46 | -0.54 |
| 75–79 | 9.7 | 2317 | 14 | 324 | 2.00 | -1.80 | 2363 | 319 | 13.48 | -0.52 |
| 80–84 | 9.7 | 2317 | 14 | 324 | 2.00 | -1.70 | 2363 | 319 | 13.49 | -0.51 |
| 85–95 | 9.7 | 2317 | 14 | 324 | 1.90 | -1.60 | 2361 | 319 | 13.52 | -0.48 |
| Females | | | | | | | | | | |
| 4–6 | 7.3 | 1744 | 15 | 262 | N/A | N/A | N/A | N/A | N/A | N/A |
| 7–9 | 8.1 | 1935 | 14 | 271 | N/A | N/A | N/A | N/A | N/A | N/A |
| 10–14 | 8.1 | 1935 | 14 | 271 | N/A | N/A | N/A | N/A | N/A | N/A |
| 15–18 | 8.2 | 1959 | 13 | 255 | -1.30 | -4.60 | 1933 | 243 | 12.57 | -0.43 |
| 19 | 8.2 | 1959 | 13.60 | 266 | -1.30 | -4.60 | 1933 | 254 | 13.15 | -0.45 |
| 20–24 | 8.2 | 1959 | 13.60 | 266 | -1.30 | -4.60 | 1933 | 254 | 13.15 | -0.45 |
| 25–29 | 8.2 | 1959 | 13.60 | 266 | -1.20 | -4.60 | 1935 | 254 | 13.13 | -0.47 |
| 30–34 | 8.2 | 1959 | 13.60 | 266 | -1.10 | -4.70 | 1937 | 254 | 13.10 | -0.50 |
| 35–39 | 8.2 | 1959 | 13.60 | 266 | -1.10 | -4.70 | 1937 | 254 | 13.10 | -0.50 |
| 40–44 | 8.2 | 1959 | 13.60 | 266 | -1.00 | -4.80 | 1939 | 254 | 13.08 | -0.52 |
| 45–49 | 8.2 | 1959 | 13.60 | 266 | -1.00 | -4.80 | 1939 | 254 | 13.08 | -0.52 |
| 50–54 | 8.2 | 1959 | 13.60 | 266 | -0.90 | -4.80 | 1941 | 254 | 13.06 | -0.54 |
| 55–59 | 8.2 | 1959 | 13.60 | 266 | -0.90 | -4.90 | 1941 | 253 | 13.05 | -0.55 |
| 60–64 | 8.2 | 1959 | 13.60 | 266 | -0.80 | -4.90 | 1943 | 253 | 13.04 | -0.56 |
| 65–69 | 7.8 | 1863 | 14.00 | 261 | -0.80 | -4.90 | 1848 | 248 | 13.42 | -0.58 |
| 70–74 | 7.8 | 1863 | 14.00 | 261 | -0.80 | -4.80 | 1848 | 248 | 13.44 | -0.56 |
| 75–79 | 7.8 | 1863 | 14.00 | 261 | -0.70 | -4.70 | 1850 | 249 | 13.44 | -0.56 |
| 80–84 | 7.8 | 1863 | 14.00 | 261 | -0.70 | -4.50 | 1850 | 249 | 13.46 | -0.54 |
| 85–95 | 7.8 | 1863 | 14.00 | 261 | -0.70 | -4.40 | 1850 | 249 | 13.48 | -0.52 |

N/A = Not available, SFA = Saturated fat, %E = Percent of total energy

^a^ from Elmadfa et al [1]

^b^ 1 MJ = 238.85 kcal

^c^ from Elmadfa et al [1]

^d^ ${SFA (kcal)}_{before}={total energy (kcal)}_{before}*{SFA (\%E)}_{before}$

^e^ from Smed et al. [2]

^f^ from Smed et al. [2]

^g^ ${total energy (kcal)}_{after}={total energy (kcal)}_{before}+({total energy \left( kcal \right)}_{before}*{energy \left( kcal, \% \right)}_{change})$

^h^ ${SFA (kcal)}_{after}={SFA (kcal)}_{before}+({SFA (kcal)}_{before}*{SFA (kcal, \%)}_{change}$)

^i^ ${SFA (\%E)}_{after}= \frac{{SFA (kcal)}_{after}}{{total energy (kcal)}_{after}}$

**References**

1. Elmadfa I, Meyer A, Nowak V, Hasenegger V, Putz P, Verstraeten R, et al. European Nutrition and Health Report 2009. Forum Nutr. 2009;62:1-405. doi: 10.1159/000242367. PMID: 20081327.

2. Smed S, Scarborough P, Rayner M, Jensen JD. The effects of the Danish saturated fat tax on food and nutrient intake and modelled health outcomes: an econometric and comparative risk assessment evaluation. Eur J Clin Nutr. 2016;70(6):681-6. doi: 10.1038/ejcn.2016.6. PMID: 27071513.
