# Supplementary material for: Substituting polyunsaturated fat for saturated fat: A health impact assessment of a fat tax in seven European countries
Source: PLoS One. 2019 Jul 10;14(7):e0218464. doi: 10.1371/journal.pone.0218464 (PMC6619676; doi:10.1371/journal.pone.0218464)
Supplement: S3 Table — (DOCX) [file pone.0218464.s003.docx]

# S3 Table. Saturated fat intake (mean and standard deviation) across scenarios in Denmark.

| Age | Males | | | | | | |  | Females | | | | | | |
| --- | --- | --- | --- | --- | --- | --- | --- | --- | --- | --- | --- | --- | --- | --- | --- |
|  | Original | | Reference scenario | | Fat tax scenario | | Guideline scenario |  | Original | | Reference scenario | | Fat tax scenario | | Guideline scenario |
|  | Mean | SD | Mean | SD | Mean | SD | Mean |  | Mean | SD | Mean | SD | Mean | SD | Mean |
| 0 | N/A | N/A | 15.42 | 2.27 | 15.42 | 2.27 | 10 |  | N/A | N/A | 15.89 | 2.45 | 15.89 | 2.45 | 10 |
| 1 | N/A | N/A | 15.35 | 2.24 | 15.35 | 2.24 | 10 |  | N/A | N/A | 15.68 | 2.43 | 15.68 | 2.43 | 10 |
| 2 | N/A | N/A | 15.28 | 2.22 | 15.28 | 2.22 | 10 |  | N/A | N/A | 15.47 | 2.42 | 15.47 | 2.42 | 10 |
| 3 | N/A | N/A | 15.2 | 2.19 | 15.2 | 2.19 | 10 |  | N/A | N/A | 15.26 | 2.41 | 15.26 | 2.41 | 10 |
| 4 | 15 | 2.2 | 15.13 | 2.17 | 15.13 | 2.17 | 10 |  | 15 | 2.4 | 15.05 | 2.39 | 15.05 | 2.39 | 10 |
| 5 | 15 | 2.2 | 15.06 | 2.14 | 15.06 | 2.14 | 10 |  | 15 | 2.4 | 14.84 | 2.38 | 14.84 | 2.38 | 10 |
| 6 | 15 | 2.2 | 14.97 | 2.12 | 14.97 | 2.12 | 10 |  | 15 | 2.4 | 14.62 | 2.36 | 14.62 | 2.36 | 10 |
| 7 | 15 | 2 | 14.87 | 2.11 | 14.87 | 2.11 | 10 |  | 14 | 2.3 | 14.42 | 2.35 | 14.42 | 2.35 | 10 |
| 8 | 15 | 2 | 14.74 | 2.11 | 14.74 | 2.11 | 10 |  | 14 | 2.3 | 14.25 | 2.34 | 14.25 | 2.34 | 10 |
| 9 | 15 | 2 | 14.57 | 2.14 | 14.57 | 2.14 | 10 |  | 14 | 2.3 | 14.11 | 2.34 | 14.11 | 2.34 | 10 |
| 10 | 14 | 2.3 | 14.39 | 2.18 | 14.39 | 2.18 | 10 |  | 14 | 2.4 | 14.01 | 2.35 | 14.01 | 2.35 | 10 |
| 11 | 14 | 2.3 | 14.23 | 2.24 | 14.23 | 2.24 | 10 |  | 14 | 2.4 | 13.91 | 2.37 | 13.91 | 2.37 | 10 |
| 12 | 14 | 2.3 | 14.11 | 2.29 | 14.11 | 2.29 | 10 |  | 14 | 2.4 | 13.81 | 2.39 | 13.81 | 2.39 | 10 |
| 13 | 14 | 2.3 | 14.03 | 2.35 | 14.03 | 2.35 | 10 |  | 14 | 2.4 | 13.69 | 2.41 | 13.69 | 2.41 | 10 |
| 14 | 14 | 2.3 | 13.99 | 2.42 | 13.99 | 2.42 | 10 |  | 14 | 2.4 | 13.55 | 2.44 | 13.55 | 2.44 | 10 |
| 15 | 14 | 2.6 | 13.98 | 2.49 | 13.31 | 2.37 | 10 |  | 13 | 2.5 | 13.41 | 2.48 | 12.98 | 2.4 | 10 |
| 16 | 14 | 2.6 | 13.99 | 2.56 | 13.32 | 2.43 | 10 |  | 13 | 2.5 | 13.31 | 2.52 | 12.88 | 2.43 | 10 |
| 17 | 14 | 2.6 | 14.02 | 2.62 | 13.35 | 2.49 | 10 |  | 13 | 2.5 | 13.27 | 2.56 | 12.83 | 2.48 | 10 |
| 18 | 14 | 2.6 | 14.06 | 2.67 | 13.39 | 2.55 | 10 |  | 13 | 2.5 | 13.29 | 2.62 | 12.85 | 2.53 | 10 |
| 19 | 14.2 | 2.8 | 14.1 | 2.72 | 13.42 | 2.59 | 10 |  | 13.6 | 2.8 | 13.35 | 2.68 | 12.89 | 2.59 | 10 |
| 20 | 14.2 | 2.8 | 14.14 | 2.76 | 13.48 | 2.63 | 10 |  | 13.6 | 2.8 | 13.42 | 2.73 | 12.97 | 2.63 | 10 |
| 21 | 14.2 | 2.8 | 14.17 | 2.79 | 13.51 | 2.66 | 10 |  | 13.6 | 2.8 | 13.49 | 2.76 | 13.04 | 2.67 | 10 |
| 22 | 14.2 | 2.8 | 14.19 | 2.8 | 13.53 | 2.67 | 10 |  | 13.6 | 2.8 | 13.54 | 2.79 | 13.09 | 2.7 | 10 |
| 23 | 14.2 | 2.8 | 14.2 | 2.81 | 13.54 | 2.68 | 10 |  | 13.6 | 2.8 | 13.58 | 2.8 | 13.13 | 2.71 | 10 |
| 24 | 14.2 | 2.8 | 14.21 | 2.81 | 13.54 | 2.68 | 10 |  | 13.6 | 2.8 | 13.6 | 2.81 | 13.15 | 2.72 | 10 |
| 25 | 14.2 | 2.8 | 14.21 | 2.81 | 13.56 | 2.68 | 10 |  | 13.6 | 2.8 | 13.61 | 2.81 | 13.14 | 2.71 | 10 |
| 26 | 14.2 | 2.8 | 14.21 | 2.81 | 13.56 | 2.68 | 10 |  | 13.6 | 2.8 | 13.61 | 2.81 | 13.15 | 2.71 | 10 |
| 27 | 14.2 | 2.8 | 14.21 | 2.8 | 13.56 | 2.68 | 10 |  | 13.6 | 2.8 | 13.61 | 2.81 | 13.15 | 2.71 | 10 |
| 28 | 14.2 | 2.8 | 14.2 | 2.8 | 13.55 | 2.67 | 10 |  | 13.6 | 2.8 | 13.61 | 2.8 | 13.14 | 2.71 | 10 |
| 29 | 14.2 | 2.8 | 14.2 | 2.8 | 13.55 | 2.67 | 10 |  | 13.6 | 2.8 | 13.61 | 2.8 | 13.14 | 2.71 | 10 |
| 30 | 14.2 | 2.8 | 14.2 | 2.8 | 13.57 | 2.68 | 10 |  | 13.6 | 2.8 | 13.6 | 2.8 | 13.11 | 2.7 | 10 |
| 31 | 14.2 | 2.8 | 14.2 | 2.8 | 13.57 | 2.67 | 10 |  | 13.6 | 2.8 | 13.6 | 2.8 | 13.11 | 2.7 | 10 |
| 32 | 14.2 | 2.8 | 14.2 | 2.8 | 13.57 | 2.67 | 10 |  | 13.6 | 2.8 | 13.6 | 2.8 | 13.11 | 2.7 | 10 |
| 33 | 14.2 | 2.8 | 14.2 | 2.8 | 13.57 | 2.67 | 10 |  | 13.6 | 2.8 | 13.6 | 2.8 | 13.1 | 2.7 | 10 |
| 34 | 14.2 | 2.8 | 14.2 | 2.8 | 13.57 | 2.67 | 10 |  | 13.6 | 2.8 | 13.6 | 2.8 | 13.1 | 2.7 | 10 |
| 35 | 14.2 | 2.8 | 14.2 | 2.8 | 13.57 | 2.67 | 10 |  | 13.6 | 2.8 | 13.6 | 2.8 | 13.1 | 2.7 | 10 |
| 36 | 14.2 | 2.8 | 14.2 | 2.8 | 13.57 | 2.68 | 10 |  | 13.6 | 2.8 | 13.6 | 2.8 | 13.1 | 2.7 | 10 |
| 37 | 14.2 | 2.8 | 14.2 | 2.8 | 13.57 | 2.68 | 10 |  | 13.6 | 2.8 | 13.6 | 2.8 | 13.1 | 2.7 | 10 |
| 38 | 14.2 | 2.8 | 14.2 | 2.8 | 13.57 | 2.68 | 10 |  | 13.6 | 2.8 | 13.6 | 2.8 | 13.1 | 2.7 | 10 |
| 39 | 14.2 | 2.8 | 14.2 | 2.8 | 13.57 | 2.68 | 10 |  | 13.6 | 2.8 | 13.6 | 2.8 | 13.1 | 2.7 | 10 |
| 40 | 14.2 | 2.8 | 14.2 | 2.8 | 13.58 | 2.68 | 10 |  | 13.6 | 2.8 | 13.6 | 2.8 | 13.08 | 2.69 | 10 |
| 41 | 14.2 | 2.8 | 14.2 | 2.8 | 13.58 | 2.68 | 10 |  | 13.6 | 2.8 | 13.6 | 2.8 | 13.08 | 2.69 | 10 |
| 42 | 14.2 | 2.8 | 14.2 | 2.8 | 13.58 | 2.68 | 10 |  | 13.6 | 2.8 | 13.6 | 2.8 | 13.08 | 2.69 | 10 |
| 43 | 14.2 | 2.8 | 14.2 | 2.8 | 13.58 | 2.68 | 10 |  | 13.6 | 2.8 | 13.6 | 2.8 | 13.08 | 2.69 | 10 |
| 44 | 14.2 | 2.8 | 14.2 | 2.8 | 13.58 | 2.68 | 10 |  | 13.6 | 2.8 | 13.6 | 2.8 | 13.08 | 2.69 | 10 |
| 45 | 14.2 | 2.8 | 14.2 | 2.8 | 13.58 | 2.68 | 10 |  | 13.6 | 2.8 | 13.6 | 2.8 | 13.08 | 2.69 | 10 |
| 46 | 14.2 | 2.8 | 14.2 | 2.8 | 13.58 | 2.68 | 10 |  | 13.6 | 2.8 | 13.6 | 2.8 | 13.08 | 2.69 | 10 |
| 47 | 14.2 | 2.8 | 14.2 | 2.8 | 13.58 | 2.68 | 10 |  | 13.6 | 2.8 | 13.6 | 2.8 | 13.08 | 2.69 | 10 |
| 48 | 14.2 | 2.8 | 14.2 | 2.8 | 13.58 | 2.68 | 10 |  | 13.6 | 2.8 | 13.6 | 2.8 | 13.08 | 2.69 | 10 |
| 49 | 14.2 | 2.8 | 14.2 | 2.8 | 13.58 | 2.68 | 10 |  | 13.6 | 2.8 | 13.6 | 2.8 | 13.08 | 2.69 | 10 |
| 50 | 14.2 | 2.8 | 14.2 | 2.8 | 13.61 | 2.68 | 10 |  | 13.6 | 2.8 | 13.6 | 2.8 | 13.07 | 2.69 | 10 |
| 51 | 14.2 | 2.8 | 14.2 | 2.8 | 13.61 | 2.68 | 10 |  | 13.6 | 2.8 | 13.6 | 2.8 | 13.06 | 2.69 | 10 |
| 52 | 14.2 | 2.8 | 14.2 | 2.8 | 13.61 | 2.68 | 10 |  | 13.6 | 2.8 | 13.6 | 2.8 | 13.06 | 2.69 | 10 |
| 53 | 14.2 | 2.8 | 14.2 | 2.8 | 13.61 | 2.68 | 10 |  | 13.6 | 2.8 | 13.6 | 2.8 | 13.06 | 2.69 | 10 |
| 54 | 14.2 | 2.8 | 14.2 | 2.8 | 13.61 | 2.68 | 10 |  | 13.6 | 2.8 | 13.6 | 2.8 | 13.06 | 2.69 | 10 |
| 55 | 14.2 | 2.8 | 14.2 | 2.8 | 13.62 | 2.68 | 10 |  | 13.6 | 2.8 | 13.59 | 2.8 | 13.04 | 2.68 | 10 |
| 56 | 14.2 | 2.8 | 14.2 | 2.8 | 13.62 | 2.68 | 10 |  | 13.6 | 2.8 | 13.59 | 2.8 | 13.04 | 2.68 | 10 |
| 57 | 14.2 | 2.8 | 14.21 | 2.79 | 13.62 | 2.68 | 10 |  | 13.6 | 2.8 | 13.59 | 2.79 | 13.04 | 2.68 | 10 |
| 58 | 14.2 | 2.8 | 14.21 | 2.79 | 13.62 | 2.68 | 10 |  | 13.6 | 2.8 | 13.59 | 2.79 | 13.04 | 2.68 | 10 |
| 59 | 14.2 | 2.8 | 14.21 | 2.79 | 13.62 | 2.68 | 10 |  | 13.6 | 2.8 | 13.59 | 2.79 | 13.04 | 2.68 | 10 |
| 60 | 14.2 | 2.8 | 14.2 | 2.8 | 13.63 | 2.69 | 10 |  | 13.6 | 2.8 | 13.6 | 2.8 | 13.04 | 2.68 | 10 |
| 61 | 14.2 | 2.8 | 14.19 | 2.81 | 13.62 | 2.7 | 10 |  | 13.6 | 2.8 | 13.62 | 2.81 | 13.06 | 2.69 | 10 |
| 62 | 14.2 | 2.8 | 14.17 | 2.83 | 13.6 | 2.71 | 10 |  | 13.6 | 2.8 | 13.65 | 2.82 | 13.09 | 2.71 | 10 |
| 63 | 14.2 | 2.8 | 14.15 | 2.85 | 13.58 | 2.74 | 10 |  | 13.6 | 2.8 | 13.7 | 2.85 | 13.14 | 2.73 | 10 |
| 64 | 14.2 | 2.8 | 14.12 | 2.88 | 13.55 | 2.77 | 10 |  | 13.6 | 2.8 | 13.76 | 2.88 | 13.2 | 2.76 | 10 |
| 65 | 14 | 3 | 14.08 | 2.92 | 13.53 | 2.8 | 10 |  | 14 | 3 | 13.84 | 2.92 | 13.26 | 2.8 | 10 |
| 66 | 14 | 3 | 14.05 | 2.95 | 13.5 | 2.84 | 10 |  | 14 | 3 | 13.9 | 2.95 | 13.32 | 2.83 | 10 |
| 67 | 14 | 3 | 14.03 | 2.98 | 13.48 | 2.86 | 10 |  | 14 | 3 | 13.95 | 2.97 | 13.37 | 2.85 | 10 |
| 68 | 14 | 3 | 14.01 | 2.99 | 13.46 | 2.87 | 10 |  | 14 | 3 | 13.98 | 2.99 | 13.4 | 2.87 | 10 |
| 69 | 14 | 3 | 14 | 3 | 13.45 | 2.88 | 10 |  | 14 | 3 | 14 | 3 | 13.42 | 2.88 | 10 |
| 70 | 14 | 3 | 13.99 | 3.01 | 13.46 | 2.89 | 10 |  | 14 | 3 | 14.01 | 3.01 | 13.45 | 2.88 | 10 |
| 71 | 14 | 3 | 13.99 | 3.01 | 13.46 | 2.89 | 10 |  | 14 | 3 | 14.01 | 3.01 | 13.45 | 2.89 | 10 |
| 72 | 14 | 3 | 13.99 | 3.01 | 13.46 | 2.89 | 10 |  | 14 | 3 | 14.01 | 3.01 | 13.45 | 2.89 | 10 |
| 73 | 14 | 3 | 14 | 3 | 13.46 | 2.89 | 10 |  | 14 | 3 | 14.01 | 3 | 13.44 | 2.88 | 10 |
| 74 | 14 | 3 | 14 | 3 | 13.46 | 2.89 | 10 |  | 14 | 3 | 14.01 | 3 | 13.44 | 2.88 | 10 |
| 75 | 14 | 3 | 14 | 3 | 13.48 | 2.89 | 10 |  | 14 | 3 | 14 | 3 | 13.44 | 2.88 | 10 |
| 76 | 14 | 3 | 14 | 3 | 13.48 | 2.89 | 10 |  | 14 | 3 | 14 | 3 | 13.44 | 2.88 | 10 |
| 77 | 14 | 3 | 14 | 3 | 13.48 | 2.89 | 10 |  | 14 | 3 | 14 | 3 | 13.44 | 2.88 | 10 |
| 78 | 14 | 3 | 14 | 3 | 13.48 | 2.89 | 10 |  | 14 | 3 | 14 | 3 | 13.44 | 2.88 | 10 |
| 79 | 14 | 3 | 14 | 3 | 13.48 | 2.89 | 10 |  | 14 | 3 | 14 | 3 | 13.44 | 2.88 | 10 |
| 80 | 14 | 3 | 14 | 3 | 13.49 | 2.89 | 10 |  | 14 | 3 | 14 | 3 | 13.46 | 2.88 | 10 |
| 81 | 14 | 3 | 14 | 3 | 13.49 | 2.89 | 10 |  | 14 | 3 | 14 | 3 | 13.46 | 2.88 | 10 |
| 82 | 14 | 3 | 14 | 3 | 13.49 | 2.89 | 10 |  | 14 | 3 | 14 | 3 | 13.46 | 2.89 | 10 |
| 83 | 14 | 3 | 14 | 3 | 13.49 | 2.89 | 10 |  | 14 | 3 | 14 | 3 | 13.46 | 2.89 | 10 |
| 84 | 14 | 3 | 14 | 3 | 13.49 | 2.89 | 10 |  | 14 | 3 | 14 | 3 | 13.46 | 2.89 | 10 |
| 85 | 14 | 3 | 14 | 3 | 13.52 | 2.9 | 10 |  | 14 | 3 | 14 | 3 | 13.48 | 2.89 | 10 |
| 86 | 14 | 3 | 14 | 3 | 13.52 | 2.9 | 10 |  | 14 | 3 | 14 | 3 | 13.48 | 2.89 | 10 |
| 87 | 14 | 3 | 14 | 3 | 13.52 | 2.9 | 10 |  | 14 | 3 | 14 | 3 | 13.48 | 2.89 | 10 |
| 88 | 14 | 3 | 14 | 3 | 13.52 | 2.9 | 10 |  | 14 | 3 | 14 | 3 | 13.48 | 2.89 | 10 |
| 89 | 14 | 3 | 14 | 3 | 13.52 | 2.9 | 10 |  | 14 | 3 | 14 | 3 | 13.48 | 2.89 | 10 |
| 90 | 14 | 3 | 14 | 3 | 13.52 | 2.9 | 10 |  | 14 | 3 | 14 | 3 | 13.48 | 2.89 | 10 |
| 91 | 14 | 3 | 14 | 3 | 13.52 | 2.9 | 10 |  | 14 | 3 | 14 | 3 | 13.48 | 2.89 | 10 |
| 92 | 14 | 3 | 14 | 3 | 13.52 | 2.9 | 10 |  | 14 | 3 | 14 | 3 | 13.48 | 2.89 | 10 |
| 93 | 14 | 3 | 14 | 3 | 13.52 | 2.9 | 10 |  | 14 | 3 | 14 | 3 | 13.48 | 2.89 | 10 |
| 94 | 14 | 3 | 14 | 3 | 13.52 | 2.9 | 10 |  | 14 | 3 | 14 | 3 | 13.48 | 2.89 | 10 |
| 95 | 14 | 3 | 14 | 3 | 13.52 | 2.9 | 10 |  | 14 | 3 | 14 | 3 | 13.48 | 2.89 | 10 |

SD = Standard deviation, N/A= Not available
