# Supplementary material for: Substituting polyunsaturated fat for saturated fat: A health impact assessment of a fat tax in seven European countries
Source: PLoS One. 2019 Jul 10;14(7):e0218464. doi: 10.1371/journal.pone.0218464 (PMC6619676; doi:10.1371/journal.pone.0218464)
Supplement: S4 Table — (DOCX) [file pone.0218464.s004.docx]

# S4 Table. Saturated fat intake (mean and standard deviation) across scenarios in Italy.

| Age | Males | | | | | | |  | Females | | | | | | |
| --- | --- | --- | --- | --- | --- | --- | --- | --- | --- | --- | --- | --- | --- | --- | --- |
|  | Original | | Reference scenario | | Fat tax scenario | | Guideline scenario |  | Original | | Reference scenario | | Fat tax scenario | | Guideline scenario |
|  | Mean | SD | Mean | SD | Mean | SD | Mean |  | Mean | SD | Mean | SD | Mean | SD | Mean |
| 0 | N/A | N/A | 11.7 | 2.69 | 11.7 | 2.69 | 10 |  | N/A | N/A | 11.04 | 1.66 | 11.04 | 1.66 | 10 |
| 1 | N/A | N/A | 11.47 | 2.64 | 11.47 | 2.64 | 10 |  | N/A | N/A | 11.01 | 1.74 | 11.01 | 1.74 | 10 |
| 2 | N/A | N/A | 11.24 | 2.59 | 11.24 | 2.59 | 10 |  | N/A | N/A | 10.97 | 1.83 | 10.97 | 1.83 | 10 |
| 3 | N/A | N/A | 11.01 | 2.54 | 11.01 | 2.54 | 10 |  | N/A | N/A | 10.94 | 1.91 | 10.94 | 1.91 | 10 |
| 4 | 10.9 | 2.5 | 10.77 | 2.49 | 10.77 | 2.49 | 10 |  | 10.8 | 2 | 10.9 | 1.99 | 10.9 | 1.99 | 10 |
| 5 | 10.9 | 2.5 | 10.54 | 2.44 | 10.54 | 2.44 | 10 |  | 10.8 | 2 | 10.86 | 2.08 | 10.86 | 2.08 | 10 |
| 6 | 10.9 | 2.5 | 10.32 | 2.39 | 10.32 | 2.39 | 10 |  | 10.8 | 2 | 10.82 | 2.16 | 10.82 | 2.16 | 10 |
| 7 | 9.4 | 2.2 | 10.15 | 2.34 | 10.15 | 2.34 | 10 |  | 10.9 | 2.4 | 10.76 | 2.23 | 10.76 | 2.23 | 10 |
| 8 | 9.4 | 2.2 | 10.08 | 2.32 | 10.08 | 2.32 | 10 |  | 10.9 | 2.4 | 10.67 | 2.29 | 10.67 | 2.29 | 10 |
| 9 | 9.4 | 2.2 | 10.14 | 2.32 | 10.14 | 2.32 | 10 |  | 10.9 | 2.4 | 10.55 | 2.32 | 10.55 | 2.32 | 10 |
| 10 | 10.8 | 2.4 | 10.28 | 2.33 | 10.28 | 2.33 | 10 |  | 10.1 | 2.3 | 10.42 | 2.33 | 10.42 | 2.33 | 10 |
| 11 | 10.8 | 2.4 | 10.46 | 2.35 | 10.46 | 2.35 | 10 |  | 10.1 | 2.3 | 10.3 | 2.33 | 10.3 | 2.33 | 10 |
| 12 | 10.8 | 2.4 | 10.64 | 2.37 | 10.64 | 2.37 | 10 |  | 10.1 | 2.3 | 10.22 | 2.32 | 10.22 | 2.32 | 10 |
| 13 | 10.8 | 2.4 | 10.8 | 2.38 | 10.8 | 2.38 | 10 |  | 10.1 | 2.3 | 10.16 | 2.31 | 10.16 | 2.31 | 10 |
| 14 | 10.8 | 2.4 | 10.93 | 2.39 | 10.93 | 2.39 | 10 |  | 10.1 | 2.3 | 10.14 | 2.31 | 10.14 | 2.31 | 10 |
| 15 | 11.2 | 2.4 | 11.01 | 2.4 | 10.34 | 2.25 | 10 |  | 10.2 | 2.3 | 10.13 | 2.31 | 9.7 | 2.22 | 10 |
| 16 | 11.2 | 2.4 | 11.05 | 2.4 | 10.38 | 2.26 | 10 |  | 10.2 | 2.3 | 10.14 | 2.33 | 9.7 | 2.23 | 10 |
| 17 | 11.2 | 2.4 | 11.03 | 2.4 | 10.36 | 2.26 | 10 |  | 10.2 | 2.3 | 10.14 | 2.35 | 9.7 | 2.25 | 10 |
| 18 | 11.2 | 2.4 | 10.96 | 2.4 | 10.29 | 2.26 | 10 |  | 10.2 | 2.3 | 10.14 | 2.38 | 9.7 | 2.28 | 10 |
| 19 | 10.6 | 2.4 | 10.87 | 2.4 | 10.19 | 2.25 | 10 |  | 10.1 | 2.5 | 10.13 | 2.42 | 9.67 | 2.31 | 10 |
| 20 | 10.6 | 2.4 | 10.78 | 2.4 | 10.11 | 2.26 | 10 |  | 10.1 | 2.5 | 10.12 | 2.45 | 9.67 | 2.34 | 10 |
| 21 | 10.6 | 2.4 | 10.7 | 2.4 | 10.03 | 2.25 | 10 |  | 10.1 | 2.5 | 10.11 | 2.47 | 9.66 | 2.36 | 10 |
| 22 | 10.6 | 2.4 | 10.64 | 2.4 | 9.98 | 2.25 | 10 |  | 10.1 | 2.5 | 10.11 | 2.49 | 9.65 | 2.38 | 10 |
| 23 | 10.6 | 2.4 | 10.61 | 2.4 | 9.94 | 2.25 | 10 |  | 10.1 | 2.5 | 10.1 | 2.5 | 9.65 | 2.39 | 10 |
| 24 | 10.6 | 2.4 | 10.59 | 2.4 | 9.92 | 2.25 | 10 |  | 10.1 | 2.5 | 10.1 | 2.51 | 9.65 | 2.39 | 10 |
| 25 | 10.6 | 2.4 | 10.58 | 2.4 | 9.93 | 2.25 | 10 |  | 10.1 | 2.5 | 10.1 | 2.51 | 9.63 | 2.39 | 10 |
| 26 | 10.6 | 2.4 | 10.58 | 2.4 | 9.93 | 2.25 | 10 |  | 10.1 | 2.5 | 10.1 | 2.51 | 9.63 | 2.39 | 10 |
| 27 | 10.6 | 2.4 | 10.59 | 2.4 | 9.94 | 2.25 | 10 |  | 10.1 | 2.5 | 10.1 | 2.5 | 9.63 | 2.39 | 10 |
| 28 | 10.6 | 2.4 | 10.59 | 2.4 | 9.94 | 2.25 | 10 |  | 10.1 | 2.5 | 10.1 | 2.5 | 9.63 | 2.39 | 10 |
| 29 | 10.6 | 2.4 | 10.59 | 2.4 | 9.94 | 2.25 | 10 |  | 10.1 | 2.5 | 10.1 | 2.5 | 9.63 | 2.39 | 10 |
| 30 | 10.6 | 2.4 | 10.6 | 2.4 | 9.96 | 2.26 | 10 |  | 10.1 | 2.5 | 10.1 | 2.5 | 9.6 | 2.38 | 10 |
| 31 | 10.6 | 2.4 | 10.6 | 2.4 | 9.96 | 2.26 | 10 |  | 10.1 | 2.5 | 10.1 | 2.5 | 9.6 | 2.38 | 10 |
| 32 | 10.6 | 2.4 | 10.6 | 2.4 | 9.97 | 2.26 | 10 |  | 10.1 | 2.5 | 10.1 | 2.5 | 9.6 | 2.38 | 10 |
| 33 | 10.6 | 2.4 | 10.6 | 2.4 | 9.97 | 2.26 | 10 |  | 10.1 | 2.5 | 10.1 | 2.5 | 9.6 | 2.38 | 10 |
| 34 | 10.6 | 2.4 | 10.6 | 2.4 | 9.97 | 2.26 | 10 |  | 10.1 | 2.5 | 10.1 | 2.5 | 9.61 | 2.38 | 10 |
| 35 | 10.6 | 2.4 | 10.6 | 2.4 | 9.97 | 2.26 | 10 |  | 10.1 | 2.5 | 10.1 | 2.5 | 9.61 | 2.38 | 10 |
| 36 | 10.6 | 2.4 | 10.6 | 2.4 | 9.97 | 2.26 | 10 |  | 10.1 | 2.5 | 10.1 | 2.5 | 9.61 | 2.38 | 10 |
| 37 | 10.6 | 2.4 | 10.6 | 2.4 | 9.97 | 2.26 | 10 |  | 10.1 | 2.5 | 10.1 | 2.5 | 9.61 | 2.38 | 10 |
| 38 | 10.6 | 2.4 | 10.6 | 2.4 | 9.97 | 2.26 | 10 |  | 10.1 | 2.5 | 10.1 | 2.5 | 9.61 | 2.38 | 10 |
| 39 | 10.6 | 2.4 | 10.6 | 2.4 | 9.97 | 2.26 | 10 |  | 10.1 | 2.5 | 10.1 | 2.5 | 9.61 | 2.38 | 10 |
| 40 | 10.6 | 2.4 | 10.6 | 2.4 | 9.98 | 2.26 | 10 |  | 10.1 | 2.5 | 10.1 | 2.5 | 9.58 | 2.37 | 10 |
| 41 | 10.6 | 2.4 | 10.6 | 2.4 | 9.98 | 2.26 | 10 |  | 10.1 | 2.5 | 10.1 | 2.5 | 9.58 | 2.37 | 10 |
| 42 | 10.6 | 2.4 | 10.6 | 2.4 | 9.98 | 2.26 | 10 |  | 10.1 | 2.5 | 10.1 | 2.5 | 9.58 | 2.37 | 10 |
| 43 | 10.6 | 2.4 | 10.6 | 2.4 | 9.98 | 2.26 | 10 |  | 10.1 | 2.5 | 10.1 | 2.5 | 9.58 | 2.37 | 10 |
| 44 | 10.6 | 2.4 | 10.6 | 2.4 | 9.98 | 2.26 | 10 |  | 10.1 | 2.5 | 10.1 | 2.5 | 9.58 | 2.37 | 10 |
| 45 | 10.6 | 2.4 | 10.6 | 2.4 | 9.98 | 2.26 | 10 |  | 10.1 | 2.5 | 10.1 | 2.5 | 9.58 | 2.37 | 10 |
| 46 | 10.6 | 2.4 | 10.6 | 2.4 | 9.98 | 2.26 | 10 |  | 10.1 | 2.5 | 10.1 | 2.5 | 9.58 | 2.37 | 10 |
| 47 | 10.6 | 2.4 | 10.6 | 2.4 | 9.98 | 2.26 | 10 |  | 10.1 | 2.5 | 10.1 | 2.5 | 9.58 | 2.37 | 10 |
| 48 | 10.6 | 2.4 | 10.6 | 2.4 | 9.98 | 2.26 | 10 |  | 10.1 | 2.5 | 10.1 | 2.5 | 9.58 | 2.37 | 10 |
| 49 | 10.6 | 2.4 | 10.6 | 2.4 | 9.98 | 2.26 | 10 |  | 10.1 | 2.5 | 10.1 | 2.5 | 9.58 | 2.37 | 10 |
| 50 | 10.6 | 2.4 | 10.6 | 2.4 | 10.01 | 2.27 | 10 |  | 10.1 | 2.5 | 10.1 | 2.5 | 9.56 | 2.37 | 10 |
| 51 | 10.6 | 2.4 | 10.6 | 2.4 | 10.01 | 2.27 | 10 |  | 10.1 | 2.5 | 10.1 | 2.5 | 9.56 | 2.37 | 10 |
| 52 | 10.6 | 2.4 | 10.6 | 2.4 | 10.01 | 2.27 | 10 |  | 10.1 | 2.5 | 10.1 | 2.5 | 9.57 | 2.37 | 10 |
| 53 | 10.6 | 2.4 | 10.6 | 2.4 | 10.01 | 2.26 | 10 |  | 10.1 | 2.5 | 10.1 | 2.5 | 9.57 | 2.37 | 10 |
| 54 | 10.6 | 2.4 | 10.61 | 2.39 | 10.02 | 2.26 | 10 |  | 10.1 | 2.5 | 10.11 | 2.5 | 9.57 | 2.37 | 10 |
| 55 | 10.6 | 2.4 | 10.61 | 2.39 | 10.02 | 2.26 | 10 |  | 10.1 | 2.5 | 10.11 | 2.5 | 9.56 | 2.36 | 10 |
| 56 | 10.6 | 2.4 | 10.61 | 2.38 | 10.03 | 2.25 | 10 |  | 10.1 | 2.5 | 10.12 | 2.5 | 9.57 | 2.36 | 10 |
| 57 | 10.6 | 2.4 | 10.62 | 2.38 | 10.03 | 2.25 | 10 |  | 10.1 | 2.5 | 10.12 | 2.5 | 9.57 | 2.36 | 10 |
| 58 | 10.6 | 2.4 | 10.62 | 2.38 | 10.03 | 2.25 | 10 |  | 10.1 | 2.5 | 10.12 | 2.5 | 9.57 | 2.36 | 10 |
| 59 | 10.6 | 2.4 | 10.62 | 2.38 | 10.03 | 2.25 | 10 |  | 10.1 | 2.5 | 10.12 | 2.5 | 9.57 | 2.36 | 10 |
| 60 | 10.6 | 2.4 | 10.6 | 2.4 | 10.03 | 2.27 | 10 |  | 10.1 | 2.5 | 10.1 | 2.5 | 9.54 | 2.36 | 10 |
| 61 | 10.6 | 2.4 | 10.57 | 2.43 | 10 | 2.3 | 10 |  | 10.1 | 2.5 | 10.07 | 2.51 | 9.51 | 2.37 | 10 |
| 62 | 10.6 | 2.4 | 10.52 | 2.48 | 9.95 | 2.35 | 10 |  | 10.1 | 2.5 | 10.01 | 2.51 | 9.45 | 2.37 | 10 |
| 63 | 10.6 | 2.4 | 10.45 | 2.56 | 9.88 | 2.42 | 10 |  | 10.1 | 2.5 | 9.92 | 2.53 | 9.36 | 2.39 | 10 |
| 64 | 10.6 | 2.4 | 10.35 | 2.66 | 9.78 | 2.51 | 10 |  | 10.1 | 2.5 | 9.81 | 2.55 | 9.25 | 2.4 | 10 |
| 65 | 10 | 3 | 10.25 | 2.76 | 9.7 | 2.62 | 10 |  | 9.4 | 2.6 | 9.69 | 2.56 | 9.11 | 2.41 | 10 |
| 66 | 10 | 3 | 10.15 | 2.86 | 9.6 | 2.7 | 10 |  | 9.4 | 2.6 | 9.58 | 2.58 | 9 | 2.42 | 10 |
| 67 | 10 | 3 | 10.08 | 2.93 | 9.53 | 2.77 | 10 |  | 9.4 | 2.6 | 9.49 | 2.59 | 8.91 | 2.43 | 10 |
| 68 | 10 | 3 | 10.03 | 2.98 | 9.48 | 2.81 | 10 |  | 9.4 | 2.6 | 9.43 | 2.6 | 8.85 | 2.44 | 10 |
| 69 | 10 | 3 | 10 | 3 | 9.45 | 2.84 | 10 |  | 9.4 | 2.6 | 9.4 | 2.6 | 8.82 | 2.44 | 10 |
| 70 | 10 | 3 | 9.98 | 3.02 | 9.45 | 2.85 | 10 |  | 9.4 | 2.6 | 9.38 | 2.6 | 8.82 | 2.45 | 10 |
| 71 | 10 | 3 | 9.98 | 3.02 | 9.44 | 2.86 | 10 |  | 9.4 | 2.6 | 9.38 | 2.6 | 8.81 | 2.45 | 10 |
| 72 | 10 | 3 | 9.98 | 3.02 | 9.45 | 2.86 | 10 |  | 9.4 | 2.6 | 9.38 | 2.6 | 8.81 | 2.45 | 10 |
| 73 | 10 | 3 | 9.99 | 3.01 | 9.45 | 2.85 | 10 |  | 9.4 | 2.6 | 9.38 | 2.6 | 8.82 | 2.45 | 10 |
| 74 | 10 | 3 | 9.99 | 3.01 | 9.45 | 2.85 | 10 |  | 9.4 | 2.6 | 9.39 | 2.6 | 8.82 | 2.44 | 10 |
| 75 | 10 | 3 | 9.99 | 3.01 | 9.47 | 2.85 | 10 |  | 9.4 | 2.6 | 9.39 | 2.6 | 8.83 | 2.44 | 10 |
| 76 | 10 | 3 | 10 | 3 | 9.48 | 2.85 | 10 |  | 9.4 | 2.6 | 9.4 | 2.6 | 8.83 | 2.44 | 10 |
| 77 | 10 | 3 | 10 | 3 | 9.48 | 2.84 | 10 |  | 9.4 | 2.6 | 9.4 | 2.6 | 8.84 | 2.44 | 10 |
| 78 | 10 | 3 | 10 | 3 | 9.48 | 2.84 | 10 |  | 9.4 | 2.6 | 9.4 | 2.6 | 8.84 | 2.44 | 10 |
| 79 | 10 | 3 | 10 | 3 | 9.48 | 2.84 | 10 |  | 9.4 | 2.6 | 9.4 | 2.6 | 8.84 | 2.44 | 10 |
| 80 | 10 | 3 | 10 | 3 | 9.49 | 2.85 | 10 |  | 9.4 | 2.6 | 9.4 | 2.6 | 8.87 | 2.45 | 10 |
| 81 | 10 | 3 | 10 | 3 | 9.49 | 2.85 | 10 |  | 9.4 | 2.6 | 9.4 | 2.6 | 8.87 | 2.45 | 10 |
| 82 | 10 | 3 | 10 | 3 | 9.49 | 2.85 | 10 |  | 9.4 | 2.6 | 9.4 | 2.6 | 8.86 | 2.45 | 10 |
| 83 | 10 | 3 | 10 | 3 | 9.49 | 2.85 | 10 |  | 9.4 | 2.6 | 9.4 | 2.6 | 8.86 | 2.45 | 10 |
| 84 | 10 | 3 | 10 | 3 | 9.49 | 2.85 | 10 |  | 9.4 | 2.6 | 9.4 | 2.6 | 8.86 | 2.45 | 10 |
| 85 | 10 | 3 | 10 | 3 | 9.52 | 2.86 | 10 |  | 9.4 | 2.6 | 9.4 | 2.6 | 8.88 | 2.46 | 10 |
| 86 | 10 | 3 | 10 | 3 | 9.52 | 2.86 | 10 |  | 9.4 | 2.6 | 9.4 | 2.6 | 8.88 | 2.46 | 10 |
| 87 | 10 | 3 | 10 | 3 | 9.52 | 2.86 | 10 |  | 9.4 | 2.6 | 9.4 | 2.6 | 8.88 | 2.46 | 10 |
| 88 | 10 | 3 | 10 | 3 | 9.52 | 2.86 | 10 |  | 9.4 | 2.6 | 9.4 | 2.6 | 8.88 | 2.46 | 10 |
| 89 | 10 | 3 | 10 | 3 | 9.52 | 2.86 | 10 |  | 9.4 | 2.6 | 9.4 | 2.6 | 8.88 | 2.46 | 10 |
| 90 | 10 | 3 | 10 | 3 | 9.52 | 2.86 | 10 |  | 9.4 | 2.6 | 9.4 | 2.6 | 8.88 | 2.46 | 10 |
| 91 | 10 | 3 | 10 | 3 | 9.52 | 2.86 | 10 |  | 9.4 | 2.6 | 9.4 | 2.6 | 8.88 | 2.46 | 10 |
| 92 | 10 | 3 | 10 | 3 | 9.52 | 2.86 | 10 |  | 9.4 | 2.6 | 9.4 | 2.6 | 8.88 | 2.46 | 10 |
| 93 | 10 | 3 | 10 | 3 | 9.52 | 2.86 | 10 |  | 9.4 | 2.6 | 9.4 | 2.6 | 8.88 | 2.46 | 10 |
| 94 | 10 | 3 | 10 | 3 | 9.52 | 2.86 | 10 |  | 9.4 | 2.6 | 9.4 | 2.6 | 8.88 | 2.46 | 10 |
| 95 | 10 | 3 | 10 | 3 | 9.52 | 2.86 | 10 |  | 9.4 | 2.6 | 9.4 | 2.6 | 8.88 | 2.46 | 10 |

SD = Standard deviation, N/A = Not available
