# Supplementary material for: Substituting polyunsaturated fat for saturated fat: A health impact assessment of a fat tax in seven European countries
Source: PLoS One. 2019 Jul 10;14(7):e0218464. doi: 10.1371/journal.pone.0218464 (PMC6619676; doi:10.1371/journal.pone.0218464)
Supplement: S5 Table — (DOCX) [file pone.0218464.s005.docx]

# S5 Table. Saturated fat intake (mean and standard deviation) across scenarios in the Netherlands.

| Age | Males | | | | | | |  | Females | | | | | |  |
| --- | --- | --- | --- | --- | --- | --- | --- | --- | --- | --- | --- | --- | --- | --- | --- |
|  | Original | | Reference scenario | | Fat tax scenario | | Guideline scenario |  | Original | | Reference scenario | | Fat tax scenario | | Guideline scenario |
|  | Mean | SD | Mean | SD | Mean | SD | Mean |  | Mean | SD | Mean | SD | Mean | SD | Mean |
| 0 | N/A | N/A | 10.67 | 1.22 | 10.67 | 1.22 | 10 |  | N/A | N/A | 10.17 | 1.25 | 10.17 | 1.25 | 10 |
| 1 | N/A | N/A | 10.96 | 1.4 | 10.96 | 1.4 | 10 |  | N/A | N/A | 10.61 | 1.42 | 10.61 | 1.42 | 10 |
| 2 | N/A | N/A | 11.25 | 1.58 | 11.25 | 1.58 | 10 |  | N/A | N/A | 11.05 | 1.59 | 11.05 | 1.59 | 10 |
| 3 | N/A | N/A | 11.54 | 1.77 | 11.54 | 1.77 | 10 |  | N/A | N/A | 11.48 | 1.77 | 11.48 | 1.77 | 10 |
| 4 | 12 | 2 | 11.83 | 1.97 | 11.83 | 1.97 | 10 |  | 12 | 2 | 11.92 | 1.96 | 11.92 | 1.96 | 10 |
| 5 | 12 | 2 | 12.12 | 2.17 | 12.12 | 2.17 | 10 |  | 12 | 2 | 12.36 | 2.17 | 12.36 | 2.17 | 10 |
| 6 | 12 | 2 | 12.43 | 2.39 | 12.43 | 2.39 | 10 |  | 12 | 2 | 12.8 | 2.38 | 12.8 | 2.38 | 10 |
| 7 | 13 | 3 | 12.74 | 2.59 | 12.74 | 2.59 | 10 |  | 14 | 3 | 13.22 | 2.59 | 13.22 | 2.59 | 10 |
| 8 | 13 | 3 | 13.04 | 2.77 | 13.04 | 2.77 | 10 |  | 14 | 3 | 13.56 | 2.76 | 13.56 | 2.76 | 10 |
| 9 | 13 | 3 | 13.33 | 2.9 | 13.33 | 2.9 | 10 |  | 14 | 3 | 13.8 | 2.89 | 13.8 | 2.89 | 10 |
| 10 | 14 | 3 | 13.58 | 2.98 | 13.58 | 2.98 | 10 |  | 14 | 3 | 13.95 | 2.97 | 13.95 | 2.97 | 10 |
| 11 | 14 | 3 | 13.77 | 3.02 | 13.77 | 3.02 | 10 |  | 14 | 3 | 14.03 | 3.01 | 14.03 | 3.01 | 10 |
| 12 | 14 | 3 | 13.91 | 3.04 | 13.91 | 3.04 | 10 |  | 14 | 3 | 14.06 | 3.03 | 14.06 | 3.03 | 10 |
| 13 | 14 | 3 | 13.99 | 3.04 | 13.99 | 3.04 | 10 |  | 14 | 3 | 14.06 | 3.03 | 14.06 | 3.03 | 10 |
| 14 | 14 | 3 | 14.04 | 3.04 | 14.04 | 3.04 | 10 |  | 14 | 3 | 14.06 | 3.03 | 14.06 | 3.03 | 10 |
| 15 | 14 | 3 | 14.07 | 3.03 | 13.4 | 2.89 | 10 |  | 14 | 3 | 14.07 | 3.04 | 13.64 | 2.95 | 10 |
| 16 | 14 | 3 | 14.09 | 3.04 | 13.42 | 2.9 | 10 |  | 14 | 3 | 14.11 | 3.07 | 13.67 | 2.97 | 10 |
| 17 | 14 | 3 | 14.11 | 3.06 | 13.44 | 2.91 | 10 |  | 14 | 3 | 14.17 | 3.11 | 13.73 | 3.01 | 10 |
| 18 | 14 | 3 | 14.15 | 3.08 | 13.48 | 2.94 | 10 |  | 14 | 3 | 14.25 | 3.17 | 13.82 | 3.07 | 10 |
| 19 | 14.3 | 3.2 | 14.19 | 3.12 | 13.51 | 2.97 | 10 |  | 14.6 | 3.4 | 14.35 | 3.23 | 13.9 | 3.13 | 10 |
| 20 | 14.3 | 3.2 | 14.23 | 3.15 | 13.57 | 3 | 10 |  | 14.6 | 3.4 | 14.45 | 3.3 | 13.99 | 3.19 | 10 |
| 21 | 14.3 | 3.2 | 14.26 | 3.17 | 13.6 | 3.02 | 10 |  | 14.6 | 3.4 | 14.52 | 3.35 | 14.07 | 3.24 | 10 |
| 22 | 14.3 | 3.2 | 14.29 | 3.19 | 13.62 | 3.04 | 10 |  | 14.6 | 3.4 | 14.57 | 3.38 | 14.12 | 3.27 | 10 |
| 23 | 14.3 | 3.2 | 14.3 | 3.2 | 13.63 | 3.05 | 10 |  | 14.6 | 3.4 | 14.6 | 3.4 | 14.15 | 3.29 | 10 |
| 24 | 14.3 | 3.2 | 14.31 | 3.2 | 13.64 | 3.06 | 10 |  | 14.6 | 3.4 | 14.61 | 3.41 | 14.16 | 3.3 | 10 |
| 25 | 14.3 | 3.2 | 14.31 | 3.21 | 13.66 | 3.06 | 10 |  | 14.6 | 3.4 | 14.62 | 3.41 | 14.15 | 3.3 | 10 |
| 26 | 14.3 | 3.2 | 14.31 | 3.21 | 13.66 | 3.06 | 10 |  | 14.6 | 3.4 | 14.62 | 3.41 | 14.15 | 3.3 | 10 |
| 27 | 14.3 | 3.2 | 14.31 | 3.2 | 13.66 | 3.06 | 10 |  | 14.6 | 3.4 | 14.61 | 3.41 | 14.15 | 3.3 | 10 |
| 28 | 14.3 | 3.2 | 14.3 | 3.2 | 13.65 | 3.06 | 10 |  | 14.6 | 3.4 | 14.61 | 3.41 | 14.14 | 3.3 | 10 |
| 29 | 14.3 | 3.2 | 14.3 | 3.2 | 13.65 | 3.06 | 10 |  | 14.6 | 3.4 | 14.61 | 3.4 | 14.14 | 3.29 | 10 |
| 30 | 14.3 | 3.2 | 14.3 | 3.2 | 13.67 | 3.06 | 10 |  | 14.6 | 3.4 | 14.6 | 3.4 | 14.11 | 3.29 | 10 |
| 31 | 14.3 | 3.2 | 14.3 | 3.2 | 13.67 | 3.06 | 10 |  | 14.6 | 3.4 | 14.6 | 3.4 | 14.11 | 3.29 | 10 |
| 32 | 14.3 | 3.2 | 14.3 | 3.2 | 13.67 | 3.06 | 10 |  | 14.6 | 3.4 | 14.6 | 3.4 | 14.1 | 3.28 | 10 |
| 33 | 14.3 | 3.2 | 14.3 | 3.2 | 13.67 | 3.06 | 10 |  | 14.6 | 3.4 | 14.6 | 3.4 | 14.1 | 3.28 | 10 |
| 34 | 14.3 | 3.2 | 14.3 | 3.2 | 13.67 | 3.06 | 10 |  | 14.6 | 3.4 | 14.6 | 3.4 | 14.1 | 3.28 | 10 |
| 35 | 14.3 | 3.2 | 14.3 | 3.2 | 13.67 | 3.06 | 10 |  | 14.6 | 3.4 | 14.6 | 3.4 | 14.1 | 3.28 | 10 |
| 36 | 14.3 | 3.2 | 14.3 | 3.2 | 13.67 | 3.06 | 10 |  | 14.6 | 3.4 | 14.6 | 3.4 | 14.1 | 3.28 | 10 |
| 37 | 14.3 | 3.2 | 14.3 | 3.2 | 13.67 | 3.06 | 10 |  | 14.6 | 3.4 | 14.6 | 3.4 | 14.1 | 3.28 | 10 |
| 38 | 14.3 | 3.2 | 14.3 | 3.2 | 13.67 | 3.06 | 10 |  | 14.6 | 3.4 | 14.6 | 3.4 | 14.1 | 3.28 | 10 |
| 39 | 14.3 | 3.2 | 14.3 | 3.2 | 13.67 | 3.06 | 10 |  | 14.6 | 3.4 | 14.6 | 3.4 | 14.1 | 3.28 | 10 |
| 40 | 14.3 | 3.2 | 14.3 | 3.2 | 13.68 | 3.06 | 10 |  | 14.6 | 3.4 | 14.6 | 3.4 | 14.08 | 3.28 | 10 |
| 41 | 14.3 | 3.2 | 14.3 | 3.2 | 13.68 | 3.06 | 10 |  | 14.6 | 3.4 | 14.6 | 3.4 | 14.08 | 3.28 | 10 |
| 42 | 14.3 | 3.2 | 14.3 | 3.2 | 13.68 | 3.06 | 10 |  | 14.6 | 3.4 | 14.6 | 3.4 | 14.08 | 3.28 | 10 |
| 43 | 14.3 | 3.2 | 14.3 | 3.2 | 13.68 | 3.06 | 10 |  | 14.6 | 3.4 | 14.6 | 3.4 | 14.08 | 3.28 | 10 |
| 44 | 14.3 | 3.2 | 14.3 | 3.2 | 13.68 | 3.06 | 10 |  | 14.6 | 3.4 | 14.6 | 3.4 | 14.08 | 3.28 | 10 |
| 45 | 14.3 | 3.2 | 14.3 | 3.2 | 13.68 | 3.06 | 10 |  | 14.6 | 3.4 | 14.6 | 3.4 | 14.08 | 3.28 | 10 |
| 46 | 14.3 | 3.2 | 14.3 | 3.2 | 13.68 | 3.06 | 10 |  | 14.6 | 3.4 | 14.6 | 3.4 | 14.08 | 3.28 | 10 |
| 47 | 14.3 | 3.2 | 14.3 | 3.2 | 13.68 | 3.06 | 10 |  | 14.6 | 3.4 | 14.6 | 3.4 | 14.08 | 3.28 | 10 |
| 48 | 14.3 | 3.2 | 14.3 | 3.2 | 13.68 | 3.06 | 10 |  | 14.6 | 3.4 | 14.6 | 3.4 | 14.08 | 3.28 | 10 |
| 49 | 14.3 | 3.2 | 14.3 | 3.2 | 13.68 | 3.06 | 10 |  | 14.6 | 3.4 | 14.6 | 3.4 | 14.08 | 3.28 | 10 |
| 50 | 14.3 | 3.2 | 14.3 | 3.2 | 13.71 | 3.07 | 10 |  | 14.6 | 3.4 | 14.6 | 3.4 | 14.07 | 3.28 | 10 |
| 51 | 14.3 | 3.2 | 14.3 | 3.2 | 13.71 | 3.07 | 10 |  | 14.6 | 3.4 | 14.6 | 3.4 | 14.06 | 3.28 | 10 |
| 52 | 14.3 | 3.2 | 14.3 | 3.2 | 13.71 | 3.07 | 10 |  | 14.6 | 3.4 | 14.6 | 3.4 | 14.06 | 3.27 | 10 |
| 53 | 14.3 | 3.2 | 14.3 | 3.2 | 13.71 | 3.07 | 10 |  | 14.6 | 3.4 | 14.6 | 3.4 | 14.06 | 3.27 | 10 |
| 54 | 14.3 | 3.2 | 14.29 | 3.19 | 13.71 | 3.06 | 10 |  | 14.6 | 3.4 | 14.6 | 3.39 | 14.06 | 3.27 | 10 |
| 55 | 14.3 | 3.2 | 14.29 | 3.19 | 13.7 | 3.06 | 10 |  | 14.6 | 3.4 | 14.59 | 3.39 | 14.04 | 3.26 | 10 |
| 56 | 14.3 | 3.2 | 14.28 | 3.18 | 13.7 | 3.05 | 10 |  | 14.6 | 3.4 | 14.59 | 3.39 | 14.04 | 3.26 | 10 |
| 57 | 14.3 | 3.2 | 14.28 | 3.18 | 13.69 | 3.05 | 10 |  | 14.6 | 3.4 | 14.59 | 3.38 | 14.04 | 3.25 | 10 |
| 58 | 14.3 | 3.2 | 14.28 | 3.17 | 13.69 | 3.04 | 10 |  | 14.6 | 3.4 | 14.59 | 3.38 | 14.04 | 3.25 | 10 |
| 59 | 14.3 | 3.2 | 14.28 | 3.18 | 13.69 | 3.05 | 10 |  | 14.6 | 3.4 | 14.59 | 3.38 | 14.04 | 3.26 | 10 |
| 60 | 14.3 | 3.2 | 14.3 | 3.2 | 13.72 | 3.07 | 10 |  | 14.6 | 3.4 | 14.6 | 3.4 | 14.04 | 3.27 | 10 |
| 61 | 14.3 | 3.2 | 14.33 | 3.23 | 13.76 | 3.1 | 10 |  | 14.6 | 3.4 | 14.62 | 3.43 | 14.06 | 3.29 | 10 |
| 62 | 14.3 | 3.2 | 14.39 | 3.3 | 13.82 | 3.17 | 10 |  | 14.6 | 3.4 | 14.65 | 3.47 | 14.09 | 3.34 | 10 |
| 63 | 14.3 | 3.2 | 14.48 | 3.39 | 13.9 | 3.26 | 10 |  | 14.6 | 3.4 | 14.7 | 3.55 | 14.14 | 3.41 | 10 |
| 64 | 14.3 | 3.2 | 14.59 | 3.52 | 14.01 | 3.38 | 10 |  | 14.6 | 3.4 | 14.76 | 3.64 | 14.2 | 3.5 | 10 |
| 65 | 15 | 4 | 14.71 | 3.66 | 14.16 | 3.53 | 10 |  | 15 | 4 | 14.84 | 3.75 | 14.26 | 3.6 | 10 |
| 66 | 15 | 4 | 14.82 | 3.79 | 14.27 | 3.65 | 10 |  | 15 | 4 | 14.9 | 3.85 | 14.32 | 3.7 | 10 |
| 67 | 15 | 4 | 14.91 | 3.9 | 14.36 | 3.75 | 10 |  | 15 | 4 | 14.95 | 3.92 | 14.37 | 3.77 | 10 |
| 68 | 15 | 4 | 14.97 | 3.96 | 14.42 | 3.82 | 10 |  | 15 | 4 | 14.98 | 3.97 | 14.4 | 3.82 | 10 |
| 69 | 15 | 4 | 15 | 4 | 14.45 | 3.86 | 10 |  | 15 | 4 | 15 | 4 | 14.42 | 3.85 | 10 |
| 70 | 15 | 4 | 15.02 | 4.02 | 14.48 | 3.88 | 10 |  | 15 | 4 | 15.01 | 4.02 | 14.45 | 3.87 | 10 |
| 71 | 15 | 4 | 15.02 | 4.03 | 14.49 | 3.88 | 10 |  | 15 | 4 | 15.01 | 4.02 | 14.45 | 3.87 | 10 |
| 72 | 15 | 4 | 15.02 | 4.03 | 14.49 | 3.88 | 10 |  | 15 | 4 | 15.01 | 4.02 | 14.45 | 3.87 | 10 |
| 73 | 15 | 4 | 15.02 | 4.02 | 14.48 | 3.88 | 10 |  | 15 | 4 | 15.01 | 4.01 | 14.44 | 3.86 | 10 |
| 74 | 15 | 4 | 15.01 | 4.01 | 14.48 | 3.87 | 10 |  | 15 | 4 | 15.01 | 4.01 | 14.44 | 3.86 | 10 |
| 75 | 15 | 4 | 15.01 | 4.01 | 14.49 | 3.87 | 10 |  | 15 | 4 | 15 | 4.01 | 14.44 | 3.86 | 10 |
| 76 | 15 | 4 | 15 | 4 | 14.48 | 3.86 | 10 |  | 15 | 4 | 15 | 4 | 14.44 | 3.85 | 10 |
| 77 | 15 | 4 | 15 | 4 | 14.48 | 3.86 | 10 |  | 15 | 4 | 15 | 4 | 14.44 | 3.85 | 10 |
| 78 | 15 | 4 | 15 | 4 | 14.48 | 3.86 | 10 |  | 15 | 4 | 15 | 4 | 14.44 | 3.85 | 10 |
| 79 | 15 | 4 | 15 | 4 | 14.48 | 3.86 | 10 |  | 15 | 4 | 15 | 4 | 14.44 | 3.85 | 10 |
| 80 | 15 | 4 | 15 | 4 | 14.49 | 3.86 | 10 |  | 15 | 4 | 15 | 4 | 14.46 | 3.86 | 10 |
| 81 | 15 | 4 | 15 | 4 | 14.49 | 3.86 | 10 |  | 15 | 4 | 15 | 4 | 14.46 | 3.86 | 10 |
| 82 | 15 | 4 | 15 | 4 | 14.49 | 3.86 | 10 |  | 15 | 4 | 15 | 4 | 14.46 | 3.86 | 10 |
| 83 | 15 | 4 | 15 | 4 | 14.49 | 3.86 | 10 |  | 15 | 4 | 15 | 4 | 14.46 | 3.86 | 10 |
| 84 | 15 | 4 | 15 | 4 | 14.49 | 3.86 | 10 |  | 15 | 4 | 15 | 4 | 14.46 | 3.86 | 10 |
| 85 | 15 | 4 | 15 | 4 | 14.52 | 3.87 | 10 |  | 15 | 4 | 15 | 4 | 14.48 | 3.86 | 10 |
| 86 | 15 | 4 | 15 | 4 | 14.52 | 3.87 | 10 |  | 15 | 4 | 15 | 4 | 14.48 | 3.86 | 10 |
| 87 | 15 | 4 | 15 | 4 | 14.52 | 3.87 | 10 |  | 15 | 4 | 15 | 4 | 14.48 | 3.86 | 10 |
| 88 | 15 | 4 | 15 | 4 | 14.52 | 3.87 | 10 |  | 15 | 4 | 15 | 4 | 14.48 | 3.86 | 10 |
| 89 | 15 | 4 | 15 | 4 | 14.52 | 3.87 | 10 |  | 15 | 4 | 15 | 4 | 14.48 | 3.86 | 10 |
| 90 | 15 | 4 | 15 | 4 | 14.52 | 3.87 | 10 |  | 15 | 4 | 15 | 4 | 14.48 | 3.86 | 10 |
| 91 | 15 | 4 | 15 | 4 | 14.52 | 3.87 | 10 |  | 15 | 4 | 15 | 4 | 14.48 | 3.86 | 10 |
| 92 | 15 | 4 | 15 | 4 | 14.52 | 3.87 | 10 |  | 15 | 4 | 15 | 4 | 14.48 | 3.86 | 10 |
| 93 | 15 | 4 | 15 | 4 | 14.52 | 3.87 | 10 |  | 15 | 4 | 15 | 4 | 14.48 | 3.86 | 10 |
| 94 | 15 | 4 | 15 | 4 | 14.52 | 3.87 | 10 |  | 15 | 4 | 15 | 4 | 14.48 | 3.86 | 10 |
| 95 | 15 | 4 | 15 | 4 | 14.52 | 3.87 | 10 |  | 15 | 4 | 15 | 4 | 14.48 | 3.86 | 10 |

SD = Standard deviation, N/A = Not available
