# Supplementary material for: Substituting polyunsaturated fat for saturated fat: A health impact assessment of a fat tax in seven European countries
Source: PLoS One. 2019 Jul 10;14(7):e0218464. doi: 10.1371/journal.pone.0218464 (PMC6619676; doi:10.1371/journal.pone.0218464)
Supplement: S6 Table — (DOCX) [file pone.0218464.s006.docx]

# S6 Table. Saturated fat intake (mean and standard deviation) across scenarios in Poland.

| Age | Males | | | | | | |  | Females | | | | | | |
| --- | --- | --- | --- | --- | --- | --- | --- | --- | --- | --- | --- | --- | --- | --- | --- |
|  | Original | | Reference scenario | | Fat tax scenario | | Guideline scenario |  | Original | | Reference scenario | | Fat tax scenario | | Guideline scenario |
|  | Mean | SD | Mean | SD | Mean | SD | Mean |  | Mean | SD | Mean | SD | Mean | SD | Mean |
| 0 | N/A | N/A | 11.19 | 3.98 | 11.19 | 3.98 | 10 |  | N/A | N/A | 12.09 | 2.99 | 12.09 | 2.99 | 10 |
| 1 | N/A | N/A | 11.29 | 3.96 | 11.29 | 3.96 | 10 |  | N/A | N/A | 12.06 | 3.06 | 12.06 | 3.06 | 10 |
| 2 | N/A | N/A | 11.38 | 3.94 | 11.38 | 3.94 | 10 |  | N/A | N/A | 12.04 | 3.13 | 12.04 | 3.13 | 10 |
| 3 | N/A | N/A | 11.48 | 3.92 | 11.48 | 3.92 | 10 |  | N/A | N/A | 12.01 | 3.2 | 12.01 | 3.2 | 10 |
| 4 | 11.6 | 3.9 | 11.57 | 3.9 | 11.57 | 3.9 | 10 |  | 12 | 3.3 | 11.98 | 3.27 | 11.98 | 3.27 | 10 |
| 5 | 11.6 | 3.9 | 11.67 | 3.88 | 11.67 | 3.88 | 10 |  | 12 | 3.3 | 11.96 | 3.34 | 11.96 | 3.34 | 10 |
| 6 | 11.6 | 3.9 | 11.76 | 3.86 | 11.76 | 3.86 | 10 |  | 12 | 3.3 | 11.93 | 3.41 | 11.93 | 3.41 | 10 |
| 7 | 12 | 3.8 | 11.86 | 3.84 | 11.86 | 3.84 | 10 |  | 11.8 | 3.6 | 11.91 | 3.48 | 11.91 | 3.48 | 10 |
| 8 | 12 | 3.8 | 11.94 | 3.82 | 11.94 | 3.82 | 10 |  | 11.8 | 3.6 | 11.91 | 3.54 | 11.91 | 3.54 | 10 |
| 9 | 12 | 3.8 | 12 | 3.81 | 12 | 3.81 | 10 |  | 11.8 | 3.6 | 11.91 | 3.6 | 11.91 | 3.6 | 10 |
| 10 | 12.1 | 3.8 | 12.05 | 3.8 | 12.05 | 3.8 | 10 |  | 12 | 3.7 | 11.93 | 3.65 | 11.93 | 3.65 | 10 |
| 11 | 12.1 | 3.8 | 12.08 | 3.79 | 12.08 | 3.79 | 10 |  | 12 | 3.7 | 11.93 | 3.71 | 11.93 | 3.71 | 10 |
| 12 | 12.1 | 3.8 | 12.1 | 3.79 | 12.1 | 3.79 | 10 |  | 12 | 3.7 | 11.93 | 3.77 | 11.93 | 3.77 | 10 |
| 13 | 12.1 | 3.8 | 12.11 | 3.79 | 12.11 | 3.79 | 10 |  | 12 | 3.7 | 11.91 | 3.84 | 11.91 | 3.84 | 10 |
| 14 | 12.1 | 3.8 | 12.11 | 3.8 | 12.11 | 3.8 | 10 |  | 12 | 3.7 | 11.87 | 3.92 | 11.87 | 3.92 | 10 |
| 15 | 12.1 | 3.8 | 12.12 | 3.8 | 11.45 | 3.59 | 10 |  | 11.7 | 4.2 | 11.82 | 4 | 11.39 | 3.86 | 10 |
| 16 | 12.1 | 3.8 | 12.13 | 3.81 | 11.47 | 3.6 | 10 |  | 11.7 | 4.2 | 11.78 | 4.07 | 11.34 | 3.92 | 10 |
| 17 | 12.1 | 3.8 | 12.16 | 3.82 | 11.49 | 3.61 | 10 |  | 11.7 | 4.2 | 11.74 | 4.12 | 11.31 | 3.97 | 10 |
| 18 | 12.1 | 3.8 | 12.19 | 3.84 | 11.52 | 3.63 | 10 |  | 11.7 | 4.2 | 11.72 | 4.14 | 11.28 | 3.99 | 10 |
| 19 | 12.3 | 3.9 | 12.22 | 3.86 | 11.54 | 3.64 | 10 |  | 11.7 | 4.1 | 11.7 | 4.15 | 11.25 | 3.98 | 10 |
| 20 | 12.3 | 3.9 | 12.25 | 3.88 | 11.59 | 3.66 | 10 |  | 11.7 | 4.1 | 11.69 | 4.14 | 11.24 | 3.98 | 10 |
| 21 | 12.3 | 3.9 | 12.27 | 3.89 | 11.61 | 3.68 | 10 |  | 11.7 | 4.1 | 11.69 | 4.13 | 11.24 | 3.97 | 10 |
| 22 | 12.3 | 3.9 | 12.29 | 3.9 | 11.63 | 3.69 | 10 |  | 11.7 | 4.1 | 11.69 | 4.12 | 11.24 | 3.96 | 10 |
| 23 | 12.3 | 3.9 | 12.3 | 3.9 | 11.64 | 3.69 | 10 |  | 11.7 | 4.1 | 11.69 | 4.11 | 11.24 | 3.95 | 10 |
| 24 | 12.3 | 3.9 | 12.31 | 3.9 | 11.64 | 3.69 | 10 |  | 11.7 | 4.1 | 11.7 | 4.1 | 11.24 | 3.95 | 10 |
| 25 | 12.3 | 3.9 | 12.31 | 3.9 | 11.66 | 3.7 | 10 |  | 11.7 | 4.1 | 11.7 | 4.1 | 11.23 | 3.94 | 10 |
| 26 | 12.3 | 3.9 | 12.31 | 3.9 | 11.66 | 3.7 | 10 |  | 11.7 | 4.1 | 11.7 | 4.1 | 11.23 | 3.93 | 10 |
| 27 | 12.3 | 3.9 | 12.3 | 3.9 | 11.66 | 3.7 | 10 |  | 11.7 | 4.1 | 11.7 | 4.1 | 11.23 | 3.93 | 10 |
| 28 | 12.3 | 3.9 | 12.3 | 3.9 | 11.65 | 3.7 | 10 |  | 11.7 | 4.1 | 11.7 | 4.1 | 11.23 | 3.93 | 10 |
| 29 | 12.3 | 3.9 | 12.3 | 3.9 | 11.65 | 3.7 | 10 |  | 11.7 | 4.1 | 11.7 | 4.1 | 11.23 | 3.93 | 10 |
| 30 | 12.3 | 3.9 | 12.3 | 3.9 | 11.67 | 3.7 | 10 |  | 11.7 | 4.1 | 11.7 | 4.1 | 11.21 | 3.93 | 10 |
| 31 | 12.3 | 3.9 | 12.3 | 3.9 | 11.67 | 3.7 | 10 |  | 11.7 | 4.1 | 11.7 | 4.1 | 11.21 | 3.93 | 10 |
| 32 | 12.3 | 3.9 | 12.3 | 3.9 | 11.67 | 3.7 | 10 |  | 11.7 | 4.1 | 11.7 | 4.1 | 11.21 | 3.93 | 10 |
| 33 | 12.3 | 3.9 | 12.3 | 3.9 | 11.67 | 3.7 | 10 |  | 11.7 | 4.1 | 11.7 | 4.1 | 11.21 | 3.93 | 10 |
| 34 | 12.3 | 3.9 | 12.3 | 3.9 | 11.67 | 3.7 | 10 |  | 11.7 | 4.1 | 11.7 | 4.1 | 11.21 | 3.93 | 10 |
| 35 | 12.3 | 3.9 | 12.3 | 3.9 | 11.67 | 3.7 | 10 |  | 11.7 | 4.1 | 11.7 | 4.1 | 11.21 | 3.93 | 10 |
| 36 | 12.3 | 3.9 | 12.3 | 3.9 | 11.67 | 3.7 | 10 |  | 11.7 | 4.1 | 11.7 | 4.1 | 11.2 | 3.93 | 10 |
| 37 | 12.3 | 3.9 | 12.3 | 3.9 | 11.67 | 3.7 | 10 |  | 11.7 | 4.1 | 11.7 | 4.1 | 11.2 | 3.93 | 10 |
| 38 | 12.3 | 3.9 | 12.3 | 3.9 | 11.67 | 3.7 | 10 |  | 11.7 | 4.1 | 11.7 | 4.1 | 11.2 | 3.93 | 10 |
| 39 | 12.3 | 3.9 | 12.3 | 3.9 | 11.67 | 3.7 | 10 |  | 11.7 | 4.1 | 11.7 | 4.1 | 11.2 | 3.93 | 10 |
| 40 | 12.3 | 3.9 | 12.3 | 3.9 | 11.68 | 3.7 | 10 |  | 11.7 | 4.1 | 11.7 | 4.1 | 11.18 | 3.92 | 10 |
| 41 | 12.3 | 3.9 | 12.3 | 3.9 | 11.68 | 3.7 | 10 |  | 11.7 | 4.1 | 11.7 | 4.1 | 11.18 | 3.92 | 10 |
| 42 | 12.3 | 3.9 | 12.3 | 3.9 | 11.68 | 3.7 | 10 |  | 11.7 | 4.1 | 11.7 | 4.1 | 11.18 | 3.92 | 10 |
| 43 | 12.3 | 3.9 | 12.3 | 3.9 | 11.68 | 3.7 | 10 |  | 11.7 | 4.1 | 11.7 | 4.1 | 11.18 | 3.92 | 10 |
| 44 | 12.3 | 3.9 | 12.3 | 3.9 | 11.68 | 3.7 | 10 |  | 11.7 | 4.1 | 11.7 | 4.1 | 11.18 | 3.92 | 10 |
| 45 | 12.3 | 3.9 | 12.3 | 3.9 | 11.68 | 3.7 | 10 |  | 11.7 | 4.1 | 11.7 | 4.1 | 11.18 | 3.92 | 10 |
| 46 | 12.3 | 3.9 | 12.3 | 3.9 | 11.68 | 3.7 | 10 |  | 11.7 | 4.1 | 11.7 | 4.1 | 11.18 | 3.92 | 10 |
| 47 | 12.3 | 3.9 | 12.3 | 3.9 | 11.68 | 3.7 | 10 |  | 11.7 | 4.1 | 11.7 | 4.1 | 11.18 | 3.92 | 10 |
| 48 | 12.3 | 3.9 | 12.3 | 3.9 | 11.68 | 3.7 | 10 |  | 11.7 | 4.1 | 11.7 | 4.1 | 11.18 | 3.92 | 10 |
| 49 | 12.3 | 3.9 | 12.3 | 3.9 | 11.68 | 3.7 | 10 |  | 11.7 | 4.1 | 11.7 | 4.1 | 11.18 | 3.92 | 10 |
| 50 | 12.3 | 3.9 | 12.3 | 3.9 | 11.71 | 3.71 | 10 |  | 11.7 | 4.1 | 11.7 | 4.1 | 11.17 | 3.91 | 10 |
| 51 | 12.3 | 3.9 | 12.3 | 3.9 | 11.71 | 3.71 | 10 |  | 11.7 | 4.1 | 11.7 | 4.1 | 11.17 | 3.91 | 10 |
| 52 | 12.3 | 3.9 | 12.3 | 3.9 | 11.71 | 3.71 | 10 |  | 11.7 | 4.1 | 11.7 | 4.1 | 11.16 | 3.91 | 10 |
| 53 | 12.3 | 3.9 | 12.3 | 3.9 | 11.71 | 3.71 | 10 |  | 11.7 | 4.1 | 11.7 | 4.1 | 11.16 | 3.91 | 10 |
| 54 | 12.3 | 3.9 | 12.3 | 3.9 | 11.71 | 3.71 | 10 |  | 11.7 | 4.1 | 11.69 | 4.1 | 11.16 | 3.91 | 10 |
| 55 | 12.3 | 3.9 | 12.29 | 3.9 | 11.7 | 3.71 | 10 |  | 11.7 | 4.1 | 11.69 | 4.1 | 11.14 | 3.91 | 10 |
| 56 | 12.3 | 3.9 | 12.29 | 3.89 | 11.7 | 3.71 | 10 |  | 11.7 | 4.1 | 11.68 | 4.1 | 11.13 | 3.91 | 10 |
| 57 | 12.3 | 3.9 | 12.28 | 3.89 | 11.7 | 3.71 | 10 |  | 11.7 | 4.1 | 11.67 | 4.1 | 11.12 | 3.91 | 10 |
| 58 | 12.3 | 3.9 | 12.28 | 3.89 | 11.7 | 3.7 | 10 |  | 11.7 | 4.1 | 11.67 | 4.1 | 11.12 | 3.91 | 10 |
| 59 | 12.3 | 3.9 | 12.29 | 3.89 | 11.7 | 3.71 | 10 |  | 11.7 | 4.1 | 11.68 | 4.1 | 11.13 | 3.91 | 10 |
| 60 | 12.3 | 3.9 | 12.3 | 3.9 | 11.73 | 3.72 | 10 |  | 11.7 | 4.1 | 11.7 | 4.1 | 11.13 | 3.9 | 10 |
| 61 | 12.3 | 3.9 | 12.32 | 3.91 | 11.75 | 3.73 | 10 |  | 11.7 | 4.1 | 11.74 | 4.1 | 11.18 | 3.9 | 10 |
| 62 | 12.3 | 3.9 | 12.36 | 3.94 | 11.79 | 3.75 | 10 |  | 11.7 | 4.1 | 11.81 | 4.09 | 11.25 | 3.9 | 10 |
| 63 | 12.3 | 3.9 | 12.43 | 3.97 | 11.85 | 3.79 | 10 |  | 11.7 | 4.1 | 11.93 | 4.08 | 11.36 | 3.89 | 10 |
| 64 | 12.3 | 3.9 | 12.51 | 4.02 | 11.93 | 3.84 | 10 |  | 11.7 | 4.1 | 12.07 | 4.07 | 11.51 | 3.88 | 10 |
| 65 | 12.8 | 4.2 | 12.59 | 4.08 | 12.04 | 3.9 | 10 |  | 12.6 | 4 | 12.23 | 4.05 | 11.65 | 3.86 | 10 |
| 66 | 12.8 | 4.2 | 12.67 | 4.12 | 12.12 | 3.94 | 10 |  | 12.6 | 4 | 12.37 | 4.03 | 11.8 | 3.84 | 10 |
| 67 | 12.8 | 4.2 | 12.74 | 4.16 | 12.19 | 3.98 | 10 |  | 12.6 | 4 | 12.49 | 4.02 | 11.91 | 3.83 | 10 |
| 68 | 12.8 | 4.2 | 12.78 | 4.19 | 12.23 | 4.01 | 10 |  | 12.6 | 4 | 12.56 | 4.01 | 11.98 | 3.82 | 10 |
| 69 | 12.8 | 4.2 | 12.8 | 4.2 | 12.25 | 4.02 | 10 |  | 12.6 | 4 | 12.6 | 4 | 12.03 | 3.82 | 10 |
| 70 | 12.8 | 4.2 | 12.81 | 4.21 | 12.28 | 4.03 | 10 |  | 12.6 | 4 | 12.62 | 4 | 12.06 | 3.82 | 10 |
| 71 | 12.8 | 4.2 | 12.82 | 4.21 | 12.28 | 4.03 | 10 |  | 12.6 | 4 | 12.63 | 4 | 12.07 | 3.82 | 10 |
| 72 | 12.8 | 4.2 | 12.82 | 4.21 | 12.28 | 4.03 | 10 |  | 12.6 | 4 | 12.63 | 4 | 12.06 | 3.82 | 10 |
| 73 | 12.8 | 4.2 | 12.81 | 4.21 | 12.28 | 4.03 | 10 |  | 12.6 | 4 | 12.62 | 4 | 12.06 | 3.82 | 10 |
| 74 | 12.8 | 4.2 | 12.81 | 4.2 | 12.27 | 4.03 | 10 |  | 12.6 | 4 | 12.61 | 4 | 12.05 | 3.82 | 10 |
| 75 | 12.8 | 4.2 | 12.8 | 4.2 | 12.28 | 4.03 | 10 |  | 12.6 | 4 | 12.61 | 4 | 12.04 | 3.82 | 10 |
| 76 | 12.8 | 4.2 | 12.8 | 4.2 | 12.28 | 4.03 | 10 |  | 12.6 | 4 | 12.6 | 4 | 12.04 | 3.82 | 10 |
| 77 | 12.8 | 4.2 | 12.8 | 4.2 | 12.28 | 4.03 | 10 |  | 12.6 | 4 | 12.6 | 4 | 12.04 | 3.82 | 10 |
| 78 | 12.8 | 4.2 | 12.8 | 4.2 | 12.28 | 4.03 | 10 |  | 12.6 | 4 | 12.6 | 4 | 12.04 | 3.82 | 10 |
| 79 | 12.8 | 4.2 | 12.8 | 4.2 | 12.28 | 4.03 | 10 |  | 12.6 | 4 | 12.6 | 4 | 12.03 | 3.82 | 10 |
| 80 | 12.8 | 4.2 | 12.8 | 4.2 | 12.29 | 4.03 | 10 |  | 12.6 | 4 | 12.6 | 4 | 12.06 | 3.83 | 10 |
| 81 | 12.8 | 4.2 | 12.8 | 4.2 | 12.29 | 4.03 | 10 |  | 12.6 | 4 | 12.6 | 4 | 12.06 | 3.83 | 10 |
| 82 | 12.8 | 4.2 | 12.8 | 4.2 | 12.29 | 4.03 | 10 |  | 12.6 | 4 | 12.6 | 4 | 12.06 | 3.83 | 10 |
| 83 | 12.8 | 4.2 | 12.8 | 4.2 | 12.29 | 4.03 | 10 |  | 12.6 | 4 | 12.6 | 4 | 12.06 | 3.83 | 10 |
| 84 | 12.8 | 4.2 | 12.8 | 4.2 | 12.29 | 4.03 | 10 |  | 12.6 | 4 | 12.6 | 4 | 12.06 | 3.83 | 10 |
| 85 | 12.8 | 4.2 | 12.8 | 4.2 | 12.32 | 4.04 | 10 |  | 12.6 | 4 | 12.6 | 4 | 12.08 | 3.83 | 10 |
| 86 | 12.8 | 4.2 | 12.8 | 4.2 | 12.32 | 4.04 | 10 |  | 12.6 | 4 | 12.6 | 4 | 12.08 | 3.83 | 10 |
| 87 | 12.8 | 4.2 | 12.8 | 4.2 | 12.32 | 4.04 | 10 |  | 12.6 | 4 | 12.6 | 4 | 12.08 | 3.83 | 10 |
| 88 | 12.8 | 4.2 | 12.8 | 4.2 | 12.32 | 4.04 | 10 |  | 12.6 | 4 | 12.6 | 4 | 12.08 | 3.83 | 10 |
| 89 | 12.8 | 4.2 | 12.8 | 4.2 | 12.32 | 4.04 | 10 |  | 12.6 | 4 | 12.6 | 4 | 12.08 | 3.83 | 10 |
| 90 | 12.8 | 4.2 | 12.8 | 4.2 | 12.32 | 4.04 | 10 |  | 12.6 | 4 | 12.6 | 4 | 12.08 | 3.83 | 10 |
| 91 | 12.8 | 4.2 | 12.8 | 4.2 | 12.32 | 4.04 | 10 |  | 12.6 | 4 | 12.6 | 4 | 12.08 | 3.83 | 10 |
| 92 | 12.8 | 4.2 | 12.8 | 4.2 | 12.32 | 4.04 | 10 |  | 12.6 | 4 | 12.6 | 4 | 12.08 | 3.83 | 10 |
| 93 | 12.8 | 4.2 | 12.8 | 4.2 | 12.32 | 4.04 | 10 |  | 12.6 | 4 | 12.6 | 4 | 12.08 | 3.83 | 10 |
| 94 | 12.8 | 4.2 | 12.8 | 4.2 | 12.32 | 4.04 | 10 |  | 12.6 | 4 | 12.6 | 4 | 12.08 | 3.83 | 10 |
| 95 | 12.8 | 4.2 | 12.8 | 4.2 | 12.32 | 4.04 | 10 |  | 12.6 | 4 | 12.6 | 4 | 12.08 | 3.83 | 10 |

SD = Standard deviation, N/A = Not available
