# Supplementary material for: Substituting polyunsaturated fat for saturated fat: A health impact assessment of a fat tax in seven European countries
Source: PLoS One. 2019 Jul 10;14(7):e0218464. doi: 10.1371/journal.pone.0218464 (PMC6619676; doi:10.1371/journal.pone.0218464)
Supplement: S7 Table — (DOCX) [file pone.0218464.s007.docx]

# S7 Table. Saturated fat intake (mean and standard deviation) across scenarios in Spain.

| Age | Males | | | | | | |  | Females | | | | | | |
| --- | --- | --- | --- | --- | --- | --- | --- | --- | --- | --- | --- | --- | --- | --- | --- |
|  | Original | | Reference scenario | | Fat tax scenario | | Guideline scenario |  | Original | | Reference scenario | | Fat tax scenario | | Guideline scenario |
|  | Mean | SD | Mean | SD | Mean | SD | Mean |  | Mean | SD | Mean | SD | Mean | SD | Mean |
| 0 | N/A | N/A | 14.86 | 1.79 | 14.86 | 1.79 | 10 |  | N/A | N/A | 13.42 | 1.1 | 13.42 | 1.1 | 10 |
| 1 | N/A | N/A | 14.82 | 1.79 | 14.82 | 1.79 | 10 |  | N/A | N/A | 13.44 | 1.11 | 13.44 | 1.11 | 10 |
| 2 | N/A | N/A | 14.77 | 1.79 | 14.77 | 1.79 | 10 |  | N/A | N/A | 13.46 | 1.12 | 13.46 | 1.12 | 10 |
| 3 | N/A | N/A | 14.73 | 1.79 | 14.73 | 1.79 | 10 |  | N/A | N/A | 13.48 | 1.12 | 13.48 | 1.12 | 10 |
| 4 | N/A | N/A | 14.68 | 1.8 | 14.68 | 1.8 | 10 |  | N/A | N/A | 13.5 | 1.13 | 13.5 | 1.13 | 10 |
| 5 | N/A | N/A | 14.64 | 1.8 | 14.64 | 1.8 | 10 |  | N/A | N/A | 13.52 | 1.14 | 13.52 | 1.14 | 10 |
| 6 | N/A | N/A | 14.59 | 1.8 | 14.59 | 1.8 | 10 |  | N/A | N/A | 13.54 | 1.14 | 13.54 | 1.14 | 10 |
| 7 | N/A | N/A | 14.55 | 1.8 | 14.55 | 1.8 | 10 |  | N/A | N/A | 13.56 | 1.15 | 13.56 | 1.15 | 10 |
| 8 | N/A | N/A | 14.5 | 1.8 | 14.5 | 1.8 | 10 |  | N/A | N/A | 13.58 | 1.16 | 13.58 | 1.16 | 10 |
| 9 | N/A | N/A | 14.46 | 1.8 | 14.46 | 1.8 | 10 |  | N/A | N/A | 13.6 | 1.16 | 13.6 | 1.16 | 10 |
| 10 | 14.3 | 1.8 | 14.41 | 1.8 | 14.41 | 1.8 | 10 |  | 13.6 | 1.2 | 13.62 | 1.17 | 13.62 | 1.17 | 10 |
| 11 | 14.3 | 1.8 | 14.36 | 1.8 | 14.36 | 1.8 | 10 |  | 13.6 | 1.2 | 13.64 | 1.17 | 13.64 | 1.17 | 10 |
| 12 | 14.3 | 1.8 | 14.31 | 1.8 | 14.31 | 1.8 | 10 |  | 13.6 | 1.2 | 13.66 | 1.18 | 13.66 | 1.18 | 10 |
| 13 | 14.3 | 1.8 | 14.23 | 1.8 | 14.23 | 1.8 | 10 |  | 13.6 | 1.2 | 13.67 | 1.2 | 13.67 | 1.2 | 10 |
| 14 | 14.3 | 1.8 | 14.11 | 1.8 | 14.11 | 1.8 | 10 |  | 13.6 | 1.2 | 13.66 | 1.23 | 13.66 | 1.23 | 10 |
| 15 | 13.8 | 1.8 | 13.95 | 1.8 | 13.28 | 1.71 | 10 |  | 13.7 | 1.3 | 13.62 | 1.28 | 13.19 | 1.24 | 10 |
| 16 | 13.8 | 1.8 | 13.74 | 1.79 | 13.07 | 1.7 | 10 |  | 13.7 | 1.3 | 13.53 | 1.35 | 13.1 | 1.31 | 10 |
| 17 | 13.8 | 1.8 | 13.49 | 1.78 | 12.82 | 1.69 | 10 |  | 13.7 | 1.3 | 13.38 | 1.45 | 12.95 | 1.4 | 10 |
| 18 | 13.8 | 1.8 | 13.2 | 1.76 | 12.53 | 1.67 | 10 |  | 13.7 | 1.3 | 13.17 | 1.56 | 12.74 | 1.51 | 10 |
| 19 | 12.3 | 1.7 | 12.9 | 1.74 | 12.22 | 1.65 | 10 |  | 12.4 | 1.9 | 12.93 | 1.68 | 12.48 | 1.62 | 10 |
| 20 | 12.3 | 1.7 | 12.64 | 1.73 | 11.97 | 1.63 | 10 |  | 12.4 | 1.9 | 12.71 | 1.78 | 12.26 | 1.71 | 10 |
| 21 | 12.3 | 1.7 | 12.45 | 1.71 | 11.79 | 1.62 | 10 |  | 12.4 | 1.9 | 12.55 | 1.84 | 12.09 | 1.78 | 10 |
| 22 | 12.3 | 1.7 | 12.33 | 1.7 | 11.67 | 1.61 | 10 |  | 12.4 | 1.9 | 12.44 | 1.89 | 11.99 | 1.82 | 10 |
| 23 | 12.3 | 1.7 | 12.27 | 1.7 | 11.61 | 1.61 | 10 |  | 12.4 | 1.9 | 12.38 | 1.91 | 11.93 | 1.84 | 10 |
| 24 | 12.3 | 1.7 | 12.25 | 1.7 | 11.58 | 1.6 | 10 |  | 12.4 | 1.9 | 12.36 | 1.92 | 11.91 | 1.85 | 10 |
| 25 | 12.3 | 1.7 | 12.25 | 1.7 | 11.6 | 1.61 | 10 |  | 12.4 | 1.9 | 12.36 | 1.92 | 11.89 | 1.85 | 10 |
| 26 | 12.3 | 1.7 | 12.26 | 1.7 | 11.61 | 1.61 | 10 |  | 12.4 | 1.9 | 12.36 | 1.91 | 11.9 | 1.84 | 10 |
| 27 | 12.3 | 1.7 | 12.27 | 1.7 | 11.62 | 1.61 | 10 |  | 12.4 | 1.9 | 12.37 | 1.91 | 11.91 | 1.84 | 10 |
| 28 | 12.3 | 1.7 | 12.28 | 1.7 | 11.63 | 1.61 | 10 |  | 12.4 | 1.9 | 12.38 | 1.91 | 11.92 | 1.83 | 10 |
| 29 | 12.3 | 1.7 | 12.29 | 1.7 | 11.64 | 1.61 | 10 |  | 12.4 | 1.9 | 12.39 | 1.9 | 11.92 | 1.83 | 10 |
| 30 | 12.3 | 1.7 | 12.3 | 1.7 | 11.66 | 1.61 | 10 |  | 12.4 | 1.9 | 12.4 | 1.9 | 11.9 | 1.83 | 10 |
| 31 | 12.3 | 1.7 | 12.3 | 1.7 | 11.67 | 1.61 | 10 |  | 12.4 | 1.9 | 12.4 | 1.9 | 11.91 | 1.82 | 10 |
| 32 | 12.3 | 1.7 | 12.3 | 1.7 | 11.67 | 1.61 | 10 |  | 12.4 | 1.9 | 12.4 | 1.9 | 11.91 | 1.82 | 10 |
| 33 | 12.3 | 1.7 | 12.3 | 1.7 | 11.67 | 1.61 | 10 |  | 12.4 | 1.9 | 12.4 | 1.9 | 11.91 | 1.82 | 10 |
| 34 | 12.3 | 1.7 | 12.3 | 1.7 | 11.67 | 1.61 | 10 |  | 12.4 | 1.9 | 12.4 | 1.9 | 11.91 | 1.82 | 10 |
| 35 | 12.3 | 1.7 | 12.3 | 1.7 | 11.67 | 1.61 | 10 |  | 12.4 | 1.9 | 12.4 | 1.9 | 11.91 | 1.82 | 10 |
| 36 | 12.3 | 1.7 | 12.3 | 1.7 | 11.67 | 1.61 | 10 |  | 12.4 | 1.9 | 12.4 | 1.9 | 11.91 | 1.82 | 10 |
| 37 | 12.3 | 1.7 | 12.3 | 1.7 | 11.67 | 1.61 | 10 |  | 12.4 | 1.9 | 12.4 | 1.9 | 11.91 | 1.82 | 10 |
| 38 | 12.3 | 1.7 | 12.3 | 1.7 | 11.67 | 1.61 | 10 |  | 12.4 | 1.9 | 12.4 | 1.9 | 11.91 | 1.82 | 10 |
| 39 | 12.3 | 1.7 | 12.3 | 1.7 | 11.67 | 1.61 | 10 |  | 12.4 | 1.9 | 12.4 | 1.9 | 11.91 | 1.82 | 10 |
| 40 | 12.3 | 1.7 | 12.3 | 1.7 | 11.68 | 1.61 | 10 |  | 12.4 | 1.9 | 12.4 | 1.9 | 11.88 | 1.82 | 10 |
| 41 | 12.3 | 1.7 | 12.3 | 1.7 | 11.68 | 1.61 | 10 |  | 12.4 | 1.9 | 12.4 | 1.9 | 11.88 | 1.82 | 10 |
| 42 | 12.3 | 1.7 | 12.3 | 1.7 | 11.68 | 1.61 | 10 |  | 12.4 | 1.9 | 12.4 | 1.9 | 11.88 | 1.82 | 10 |
| 43 | 12.3 | 1.7 | 12.3 | 1.7 | 11.68 | 1.61 | 10 |  | 12.4 | 1.9 | 12.4 | 1.9 | 11.88 | 1.82 | 10 |
| 44 | 12.3 | 1.7 | 12.3 | 1.7 | 11.68 | 1.61 | 10 |  | 12.4 | 1.9 | 12.4 | 1.9 | 11.88 | 1.82 | 10 |
| 45 | 12.3 | 1.7 | 12.3 | 1.7 | 11.68 | 1.61 | 10 |  | 12.4 | 1.9 | 12.4 | 1.9 | 11.88 | 1.82 | 10 |
| 46 | 12.3 | 1.7 | 12.3 | 1.7 | 11.68 | 1.61 | 10 |  | 12.4 | 1.9 | 12.4 | 1.9 | 11.88 | 1.82 | 10 |
| 47 | 12.3 | 1.7 | 12.3 | 1.7 | 11.68 | 1.61 | 10 |  | 12.4 | 1.9 | 12.4 | 1.9 | 11.88 | 1.82 | 10 |
| 48 | 12.3 | 1.7 | 12.3 | 1.7 | 11.68 | 1.61 | 10 |  | 12.4 | 1.9 | 12.4 | 1.9 | 11.88 | 1.82 | 10 |
| 49 | 12.3 | 1.7 | 12.3 | 1.7 | 11.68 | 1.61 | 10 |  | 12.4 | 1.9 | 12.4 | 1.9 | 11.88 | 1.82 | 10 |
| 50 | 12.3 | 1.7 | 12.3 | 1.7 | 11.71 | 1.62 | 10 |  | 12.4 | 1.9 | 12.4 | 1.9 | 11.86 | 1.82 | 10 |
| 51 | 12.3 | 1.7 | 12.3 | 1.7 | 11.71 | 1.62 | 10 |  | 12.4 | 1.9 | 12.4 | 1.9 | 11.86 | 1.82 | 10 |
| 52 | 12.3 | 1.7 | 12.3 | 1.7 | 11.71 | 1.62 | 10 |  | 12.4 | 1.9 | 12.4 | 1.9 | 11.86 | 1.82 | 10 |
| 53 | 12.3 | 1.7 | 12.3 | 1.7 | 11.72 | 1.62 | 10 |  | 12.4 | 1.9 | 12.4 | 1.9 | 11.87 | 1.82 | 10 |
| 54 | 12.3 | 1.7 | 12.31 | 1.7 | 11.72 | 1.62 | 10 |  | 12.4 | 1.9 | 12.41 | 1.91 | 11.87 | 1.82 | 10 |
| 55 | 12.3 | 1.7 | 12.32 | 1.7 | 11.74 | 1.62 | 10 |  | 12.4 | 1.9 | 12.42 | 1.91 | 11.87 | 1.83 | 10 |
| 56 | 12.3 | 1.7 | 12.34 | 1.7 | 11.75 | 1.62 | 10 |  | 12.4 | 1.9 | 12.43 | 1.92 | 11.88 | 1.83 | 10 |
| 57 | 12.3 | 1.7 | 12.36 | 1.7 | 11.77 | 1.62 | 10 |  | 12.4 | 1.9 | 12.45 | 1.92 | 11.9 | 1.84 | 10 |
| 58 | 12.3 | 1.7 | 12.37 | 1.7 | 11.78 | 1.62 | 10 |  | 12.4 | 1.9 | 12.45 | 1.93 | 11.9 | 1.84 | 10 |
| 59 | 12.3 | 1.7 | 12.36 | 1.7 | 11.78 | 1.62 | 10 |  | 12.4 | 1.9 | 12.45 | 1.93 | 11.9 | 1.84 | 10 |
| 60 | 12.3 | 1.7 | 12.33 | 1.7 | 11.75 | 1.62 | 10 |  | 12.4 | 1.9 | 12.42 | 1.91 | 11.86 | 1.83 | 10 |
| 61 | 12.3 | 1.7 | 12.24 | 1.7 | 11.67 | 1.62 | 10 |  | 12.4 | 1.9 | 12.35 | 1.87 | 11.79 | 1.79 | 10 |
| 62 | 12.3 | 1.7 | 12.08 | 1.69 | 11.5 | 1.61 | 10 |  | 12.4 | 1.9 | 12.22 | 1.8 | 11.66 | 1.72 | 10 |
| 63 | 12.3 | 1.7 | 11.82 | 1.68 | 11.25 | 1.6 | 10 |  | 12.4 | 1.9 | 12.02 | 1.69 | 11.46 | 1.62 | 10 |
| 64 | 12.3 | 1.7 | 11.49 | 1.67 | 10.92 | 1.58 | 10 |  | 12.4 | 1.9 | 11.75 | 1.56 | 11.19 | 1.48 | 10 |
| 65 | 10.3 | 1.6 | 11.11 | 1.65 | 10.56 | 1.57 | 10 |  | 10.8 | 1.1 | 11.45 | 1.4 | 10.87 | 1.33 | 10 |
| 66 | 10.3 | 1.6 | 10.78 | 1.63 | 10.23 | 1.55 | 10 |  | 10.8 | 1.1 | 11.18 | 1.28 | 10.6 | 1.21 | 10 |
| 67 | 10.3 | 1.6 | 10.52 | 1.61 | 9.97 | 1.53 | 10 |  | 10.8 | 1.1 | 10.98 | 1.18 | 10.4 | 1.12 | 10 |
| 68 | 10.3 | 1.6 | 10.36 | 1.6 | 9.81 | 1.52 | 10 |  | 10.8 | 1.1 | 10.85 | 1.12 | 10.27 | 1.06 | 10 |
| 69 | 10.3 | 1.6 | 10.27 | 1.6 | 9.72 | 1.51 | 10 |  | 10.8 | 1.1 | 10.78 | 1.09 | 10.2 | 1.03 | 10 |
| 70 | 10.3 | 1.6 | 10.24 | 1.6 | 9.7 | 1.51 | 10 |  | 10.8 | 1.1 | 10.75 | 1.08 | 10.19 | 1.02 | 10 |
| 71 | 10.3 | 1.6 | 10.23 | 1.6 | 9.7 | 1.51 | 10 |  | 10.8 | 1.1 | 10.75 | 1.08 | 10.18 | 1.02 | 10 |
| 72 | 10.3 | 1.6 | 10.24 | 1.6 | 9.71 | 1.51 | 10 |  | 10.8 | 1.1 | 10.75 | 1.08 | 10.19 | 1.02 | 10 |
| 73 | 10.3 | 1.6 | 10.26 | 1.6 | 9.72 | 1.51 | 10 |  | 10.8 | 1.1 | 10.77 | 1.09 | 10.2 | 1.03 | 10 |
| 74 | 10.3 | 1.6 | 10.28 | 1.6 | 9.74 | 1.52 | 10 |  | 10.8 | 1.1 | 10.78 | 1.09 | 10.22 | 1.03 | 10 |
| 75 | 10.3 | 1.6 | 10.29 | 1.6 | 9.77 | 1.52 | 10 |  | 10.8 | 1.1 | 10.79 | 1.1 | 10.23 | 1.04 | 10 |
| 76 | 10.3 | 1.6 | 10.3 | 1.6 | 9.77 | 1.52 | 10 |  | 10.8 | 1.1 | 10.8 | 1.1 | 10.23 | 1.04 | 10 |
| 77 | 10.3 | 1.6 | 10.3 | 1.6 | 9.78 | 1.52 | 10 |  | 10.8 | 1.1 | 10.8 | 1.1 | 10.24 | 1.04 | 10 |
| 78 | 10.3 | 1.6 | 10.3 | 1.6 | 9.78 | 1.52 | 10 |  | 10.8 | 1.1 | 10.8 | 1.1 | 10.24 | 1.04 | 10 |
| 79 | 10.3 | 1.6 | 10.3 | 1.6 | 9.78 | 1.52 | 10 |  | 10.8 | 1.1 | 10.8 | 1.1 | 10.24 | 1.04 | 10 |
| 80 | 10.3 | 1.6 | 10.3 | 1.6 | 9.79 | 1.52 | 10 |  | 10.8 | 1.1 | 10.8 | 1.1 | 10.27 | 1.05 | 10 |
| 81 | 10.3 | 1.6 | 10.3 | 1.6 | 9.79 | 1.52 | 10 |  | 10.8 | 1.1 | 10.8 | 1.1 | 10.27 | 1.05 | 10 |
| 82 | 10.3 | 1.6 | 10.3 | 1.6 | 9.79 | 1.52 | 10 |  | 10.8 | 1.1 | 10.8 | 1.1 | 10.27 | 1.05 | 10 |
| 83 | 10.3 | 1.6 | 10.3 | 1.6 | 9.79 | 1.52 | 10 |  | 10.8 | 1.1 | 10.8 | 1.1 | 10.26 | 1.05 | 10 |
| 84 | 10.3 | 1.6 | 10.3 | 1.6 | 9.79 | 1.52 | 10 |  | 10.8 | 1.1 | 10.8 | 1.1 | 10.26 | 1.05 | 10 |
| 85 | 10.3 | 1.6 | 10.3 | 1.6 | 9.82 | 1.53 | 10 |  | 10.8 | 1.1 | 10.8 | 1.1 | 10.28 | 1.05 | 10 |
| 86 | 10.3 | 1.6 | 10.3 | 1.6 | 9.82 | 1.53 | 10 |  | 10.8 | 1.1 | 10.8 | 1.1 | 10.28 | 1.05 | 10 |
| 87 | 10.3 | 1.6 | 10.3 | 1.6 | 9.82 | 1.53 | 10 |  | 10.8 | 1.1 | 10.8 | 1.1 | 10.28 | 1.05 | 10 |
| 88 | 10.3 | 1.6 | 10.3 | 1.6 | 9.82 | 1.53 | 10 |  | 10.8 | 1.1 | 10.8 | 1.1 | 10.28 | 1.05 | 10 |
| 89 | 10.3 | 1.6 | 10.3 | 1.6 | 9.82 | 1.53 | 10 |  | 10.8 | 1.1 | 10.8 | 1.1 | 10.28 | 1.05 | 10 |
| 90 | 10.3 | 1.6 | 10.3 | 1.6 | 9.82 | 1.53 | 10 |  | 10.8 | 1.1 | 10.8 | 1.1 | 10.28 | 1.05 | 10 |
| 91 | 10.3 | 1.6 | 10.3 | 1.6 | 9.82 | 1.53 | 10 |  | 10.8 | 1.1 | 10.8 | 1.1 | 10.28 | 1.05 | 10 |
| 92 | 10.3 | 1.6 | 10.3 | 1.6 | 9.82 | 1.53 | 10 |  | 10.8 | 1.1 | 10.8 | 1.1 | 10.28 | 1.05 | 10 |
| 93 | 10.3 | 1.6 | 10.3 | 1.6 | 9.82 | 1.53 | 10 |  | 10.8 | 1.1 | 10.8 | 1.1 | 10.28 | 1.05 | 10 |
| 94 | 10.3 | 1.6 | 10.3 | 1.6 | 9.82 | 1.53 | 10 |  | 10.8 | 1.1 | 10.8 | 1.1 | 10.28 | 1.05 | 10 |
| 95 | 10.3 | 1.6 | 10.3 | 1.6 | 9.82 | 1.53 | 10 |  | 10.8 | 1.1 | 10.8 | 1.1 | 10.28 | 1.05 | 10 |

SD = Standard deviation, N/A = Not available
