# Supplementary material for: Substituting polyunsaturated fat for saturated fat: A health impact assessment of a fat tax in seven European countries
Source: PLoS One. 2019 Jul 10;14(7):e0218464. doi: 10.1371/journal.pone.0218464 (PMC6619676; doi:10.1371/journal.pone.0218464)
Supplement: S8 Table — (DOCX) [file pone.0218464.s008.docx]

# S8 Table. Saturated fat intake (mean and standard deviation) across scenarios in Sweden.

| Age | Males | | | | | | |  | Females | | | | | | |
| --- | --- | --- | --- | --- | --- | --- | --- | --- | --- | --- | --- | --- | --- | --- | --- |
|  | Original | | Reference scenario | | Fat tax scenario | | Guideline scenario |  | Original | | Reference scenario | | Fat tax scenario | | Guideline scenario |
|  | Mean | SD | Mean | SD | Mean | SD | Mean |  | Mean | SD | Mean | SD | Mean | SD | Mean |
| 0 | N/A | N/A | 14.51 | 2.49 | 14.51 | 2.49 | 10 |  | N/A | N/A | 15.14 | 2.72 | 15.14 | 2.72 | 10 |
| 1 | N/A | N/A | 14.47 | 2.47 | 14.47 | 2.47 | 10 |  | N/A | N/A | 15.02 | 2.67 | 15.02 | 2.67 | 10 |
| 2 | N/A | N/A | 14.42 | 2.45 | 14.42 | 2.45 | 10 |  | N/A | N/A | 14.9 | 2.62 | 14.9 | 2.62 | 10 |
| 3 | N/A | N/A | 14.37 | 2.42 | 14.37 | 2.42 | 10 |  | N/A | N/A | 14.78 | 2.57 | 14.78 | 2.57 | 10 |
| 4 | 14.3 | 2.4 | 14.32 | 2.4 | 14.32 | 2.4 | 10 |  | 14.6 | 2.5 | 14.66 | 2.52 | 14.66 | 2.52 | 10 |
| 5 | 14.3 | 2.4 | 14.27 | 2.38 | 14.27 | 2.38 | 10 |  | 14.6 | 2.5 | 14.53 | 2.47 | 14.53 | 2.47 | 10 |
| 6 | 14.3 | 2.4 | 14.22 | 2.36 | 14.22 | 2.36 | 10 |  | 14.6 | 2.5 | 14.4 | 2.42 | 14.4 | 2.42 | 10 |
| 7 | 14.1 | 2.3 | 14.17 | 2.34 | 14.17 | 2.34 | 10 |  | 14.1 | 2.3 | 14.27 | 2.37 | 14.27 | 2.37 | 10 |
| 8 | 14.1 | 2.3 | 14.13 | 2.33 | 14.13 | 2.33 | 10 |  | 14.1 | 2.3 | 14.16 | 2.33 | 14.16 | 2.33 | 10 |
| 9 | 14.1 | 2.3 | 14.1 | 2.34 | 14.1 | 2.34 | 10 |  | 14.1 | 2.3 | 14.08 | 2.31 | 14.08 | 2.31 | 10 |
| 10 | 14.1 | 2.4 | 14.09 | 2.35 | 14.09 | 2.35 | 10 |  | 14 | 2.3 | 14.02 | 2.3 | 14.02 | 2.3 | 10 |
| 11 | 14.1 | 2.4 | 14.08 | 2.37 | 14.08 | 2.37 | 10 |  | 14 | 2.3 | 13.99 | 2.29 | 13.99 | 2.29 | 10 |
| 12 | 14.1 | 2.4 | 14.09 | 2.39 | 14.09 | 2.39 | 10 |  | 14 | 2.3 | 13.99 | 2.29 | 13.99 | 2.29 | 10 |
| 13 | 14.1 | 2.4 | 14.12 | 2.41 | 14.12 | 2.41 | 10 |  | 14 | 2.3 | 14 | 2.29 | 14 | 2.29 | 10 |
| 14 | 14.1 | 2.4 | 14.17 | 2.45 | 14.17 | 2.45 | 10 |  | 14 | 2.3 | 14.03 | 2.3 | 14.03 | 2.3 | 10 |
| 15 | N/A | N/A | 14.23 | 2.49 | 13.56 | 2.37 | 10 |  | N/A | N/A | 14.07 | 2.3 | 13.63 | 2.23 | 10 |
| 16 | N/A | N/A | 14.31 | 2.53 | 13.64 | 2.41 | 10 |  | N/A | N/A | 14.12 | 2.3 | 13.68 | 2.23 | 10 |
| 17 | N/A | N/A | 14.39 | 2.58 | 13.72 | 2.46 | 10 |  | N/A | N/A | 14.17 | 2.3 | 13.73 | 2.23 | 10 |
| 18 | N/A | N/A | 14.47 | 2.62 | 13.8 | 2.5 | 10 |  | N/A | N/A | 14.22 | 2.3 | 13.78 | 2.23 | 10 |
| 19 | 14.6 | 2.7 | 14.53 | 2.66 | 13.85 | 2.54 | 10 |  | 14.3 | 2.3 | 14.26 | 2.3 | 13.8 | 2.23 | 10 |
| 20 | 14.6 | 2.7 | 14.58 | 2.69 | 13.91 | 2.56 | 10 |  | 14.3 | 2.3 | 14.28 | 2.3 | 13.83 | 2.23 | 10 |
| 21 | 14.6 | 2.7 | 14.6 | 2.7 | 13.94 | 2.58 | 10 |  | 14.3 | 2.3 | 14.3 | 2.3 | 13.85 | 2.23 | 10 |
| 22 | 14.6 | 2.7 | 14.61 | 2.71 | 13.94 | 2.58 | 10 |  | 14.3 | 2.3 | 14.31 | 2.3 | 13.85 | 2.23 | 10 |
| 23 | 14.6 | 2.7 | 14.61 | 2.71 | 13.95 | 2.58 | 10 |  | 14.3 | 2.3 | 14.31 | 2.3 | 13.85 | 2.23 | 10 |
| 24 | 14.6 | 2.7 | 14.61 | 2.71 | 13.94 | 2.58 | 10 |  | 14.3 | 2.3 | 14.31 | 2.3 | 13.85 | 2.23 | 10 |
| 25 | 14.6 | 2.7 | 14.61 | 2.7 | 13.96 | 2.58 | 10 |  | 14.3 | 2.3 | 14.3 | 2.3 | 13.84 | 2.22 | 10 |
| 26 | 14.6 | 2.7 | 14.6 | 2.7 | 13.95 | 2.58 | 10 |  | 14.3 | 2.3 | 14.3 | 2.3 | 13.83 | 2.22 | 10 |
| 27 | 14.6 | 2.7 | 14.6 | 2.7 | 13.95 | 2.58 | 10 |  | 14.3 | 2.3 | 14.3 | 2.3 | 13.83 | 2.22 | 10 |
| 28 | 14.6 | 2.7 | 14.6 | 2.7 | 13.95 | 2.58 | 10 |  | 14.3 | 2.3 | 14.3 | 2.3 | 13.83 | 2.22 | 10 |
| 29 | 14.6 | 2.7 | 14.6 | 2.7 | 13.95 | 2.58 | 10 |  | 14.3 | 2.3 | 14.3 | 2.3 | 13.83 | 2.22 | 10 |
| 30 | 14.6 | 2.7 | 14.6 | 2.7 | 13.97 | 2.58 | 10 |  | 14.3 | 2.3 | 14.3 | 2.3 | 13.8 | 2.22 | 10 |
| 31 | 14.6 | 2.7 | 14.6 | 2.7 | 13.97 | 2.58 | 10 |  | 14.3 | 2.3 | 14.3 | 2.3 | 13.8 | 2.22 | 10 |
| 32 | 14.6 | 2.7 | 14.6 | 2.7 | 13.97 | 2.58 | 10 |  | 14.3 | 2.3 | 14.3 | 2.3 | 13.8 | 2.22 | 10 |
| 33 | 14.6 | 2.7 | 14.6 | 2.7 | 13.97 | 2.58 | 10 |  | 14.3 | 2.3 | 14.3 | 2.3 | 13.8 | 2.22 | 10 |
| 34 | 14.6 | 2.7 | 14.6 | 2.7 | 13.97 | 2.58 | 10 |  | 14.3 | 2.3 | 14.3 | 2.3 | 13.8 | 2.22 | 10 |
| 35 | 14.6 | 2.7 | 14.6 | 2.7 | 13.97 | 2.58 | 10 |  | 14.3 | 2.3 | 14.3 | 2.3 | 13.8 | 2.22 | 10 |
| 36 | 14.6 | 2.7 | 14.6 | 2.7 | 13.97 | 2.58 | 10 |  | 14.3 | 2.3 | 14.3 | 2.3 | 13.8 | 2.22 | 10 |
| 37 | 14.6 | 2.7 | 14.6 | 2.7 | 13.97 | 2.58 | 10 |  | 14.3 | 2.3 | 14.3 | 2.3 | 13.8 | 2.22 | 10 |
| 38 | 14.6 | 2.7 | 14.6 | 2.7 | 13.97 | 2.58 | 10 |  | 14.3 | 2.3 | 14.3 | 2.3 | 13.8 | 2.22 | 10 |
| 39 | 14.6 | 2.7 | 14.6 | 2.7 | 13.97 | 2.58 | 10 |  | 14.3 | 2.3 | 14.3 | 2.3 | 13.8 | 2.22 | 10 |
| 40 | 14.6 | 2.7 | 14.6 | 2.7 | 13.98 | 2.59 | 10 |  | 14.3 | 2.3 | 14.3 | 2.3 | 13.78 | 2.22 | 10 |
| 41 | 14.6 | 2.7 | 14.6 | 2.7 | 13.98 | 2.59 | 10 |  | 14.3 | 2.3 | 14.3 | 2.3 | 13.78 | 2.22 | 10 |
| 42 | 14.6 | 2.7 | 14.6 | 2.7 | 13.98 | 2.59 | 10 |  | 14.3 | 2.3 | 14.3 | 2.3 | 13.78 | 2.22 | 10 |
| 43 | 14.6 | 2.7 | 14.6 | 2.7 | 13.98 | 2.59 | 10 |  | 14.3 | 2.3 | 14.3 | 2.3 | 13.78 | 2.22 | 10 |
| 44 | 14.6 | 2.7 | 14.6 | 2.7 | 13.98 | 2.59 | 10 |  | 14.3 | 2.3 | 14.3 | 2.3 | 13.78 | 2.22 | 10 |
| 45 | 14.6 | 2.7 | 14.6 | 2.7 | 13.98 | 2.59 | 10 |  | 14.3 | 2.3 | 14.3 | 2.3 | 13.78 | 2.22 | 10 |
| 46 | 14.6 | 2.7 | 14.6 | 2.7 | 13.98 | 2.59 | 10 |  | 14.3 | 2.3 | 14.3 | 2.3 | 13.78 | 2.22 | 10 |
| 47 | 14.6 | 2.7 | 14.6 | 2.7 | 13.98 | 2.59 | 10 |  | 14.3 | 2.3 | 14.3 | 2.3 | 13.78 | 2.22 | 10 |
| 48 | 14.6 | 2.7 | 14.6 | 2.7 | 13.98 | 2.59 | 10 |  | 14.3 | 2.3 | 14.3 | 2.3 | 13.78 | 2.22 | 10 |
| 49 | 14.6 | 2.7 | 14.6 | 2.7 | 13.98 | 2.59 | 10 |  | 14.3 | 2.3 | 14.3 | 2.3 | 13.78 | 2.22 | 10 |
| 50 | 14.6 | 2.7 | 14.6 | 2.7 | 14.01 | 2.59 | 10 |  | 14.3 | 2.3 | 14.3 | 2.3 | 13.76 | 2.21 | 10 |
| 51 | 14.6 | 2.7 | 14.6 | 2.7 | 14.01 | 2.59 | 10 |  | 14.3 | 2.3 | 14.3 | 2.3 | 13.76 | 2.21 | 10 |
| 52 | 14.6 | 2.7 | 14.6 | 2.7 | 14.01 | 2.59 | 10 |  | 14.3 | 2.3 | 14.3 | 2.3 | 13.76 | 2.21 | 10 |
| 53 | 14.6 | 2.7 | 14.6 | 2.7 | 14.01 | 2.59 | 10 |  | 14.3 | 2.3 | 14.3 | 2.3 | 13.76 | 2.21 | 10 |
| 54 | 14.6 | 2.7 | 14.6 | 2.7 | 14.01 | 2.59 | 10 |  | 14.3 | 2.3 | 14.3 | 2.3 | 13.76 | 2.21 | 10 |
| 55 | 14.6 | 2.7 | 14.6 | 2.7 | 14.01 | 2.59 | 10 |  | 14.3 | 2.3 | 14.3 | 2.3 | 13.75 | 2.21 | 10 |
| 56 | 14.6 | 2.7 | 14.6 | 2.7 | 14.01 | 2.59 | 10 |  | 14.3 | 2.3 | 14.3 | 2.3 | 13.75 | 2.21 | 10 |
| 57 | 14.6 | 2.7 | 14.59 | 2.69 | 14.01 | 2.58 | 10 |  | 14.3 | 2.3 | 14.3 | 2.3 | 13.75 | 2.21 | 10 |
| 58 | 14.6 | 2.7 | 14.59 | 2.69 | 14 | 2.58 | 10 |  | 14.3 | 2.3 | 14.3 | 2.3 | 13.75 | 2.21 | 10 |
| 59 | 14.6 | 2.7 | 14.59 | 2.68 | 14 | 2.58 | 10 |  | 14.3 | 2.3 | 14.3 | 2.3 | 13.75 | 2.21 | 10 |
| 60 | 14.6 | 2.7 | 14.59 | 2.69 | 14.02 | 2.58 | 10 |  | 14.3 | 2.3 | 14.3 | 2.3 | 13.74 | 2.21 | 10 |
| 61 | 14.6 | 2.7 | 14.6 | 2.7 | 14.03 | 2.59 | 10 |  | 14.3 | 2.3 | 14.3 | 2.3 | 13.74 | 2.21 | 10 |
| 62 | 14.6 | 2.7 | 14.63 | 2.73 | 14.05 | 2.63 | 10 |  | 14.3 | 2.3 | 14.3 | 2.29 | 13.74 | 2.2 | 10 |
| 63 | 14.6 | 2.7 | 14.68 | 2.8 | 14.11 | 2.69 | 10 |  | 14.3 | 2.3 | 14.3 | 2.28 | 13.74 | 2.19 | 10 |
| 64 | 14.6 | 2.7 | 14.75 | 2.89 | 14.18 | 2.78 | 10 |  | 14.3 | 2.3 | 14.3 | 2.26 | 13.74 | 2.17 | 10 |
| 65 | 15 | 3.2 | 14.84 | 3 | 14.29 | 2.89 | 10 |  | 14.3 | 2.2 | 14.3 | 2.24 | 13.72 | 2.15 | 10 |
| 66 | 15 | 3.2 | 14.92 | 3.1 | 14.37 | 2.99 | 10 |  | 14.3 | 2.2 | 14.3 | 2.22 | 13.72 | 2.13 | 10 |
| 67 | 15 | 3.2 | 14.97 | 3.17 | 14.42 | 3.05 | 10 |  | 14.3 | 2.2 | 14.3 | 2.21 | 13.72 | 2.12 | 10 |
| 68 | 15 | 3.2 | 15 | 3.2 | 14.45 | 3.08 | 10 |  | 14.3 | 2.2 | 14.3 | 2.2 | 13.72 | 2.11 | 10 |
| 69 | 15 | 3.2 | 15.01 | 3.22 | 14.46 | 3.1 | 10 |  | 14.3 | 2.2 | 14.3 | 2.2 | 13.72 | 2.11 | 10 |
| 70 | 15 | 3.2 | 15.01 | 3.22 | 14.48 | 3.1 | 10 |  | 14.3 | 2.2 | 14.3 | 2.2 | 13.74 | 2.11 | 10 |
| 71 | 15 | 3.2 | 15.01 | 3.21 | 14.48 | 3.1 | 10 |  | 14.3 | 2.2 | 14.3 | 2.2 | 13.74 | 2.11 | 10 |
| 72 | 15 | 3.2 | 15.01 | 3.21 | 14.47 | 3.09 | 10 |  | 14.3 | 2.2 | 14.3 | 2.2 | 13.74 | 2.11 | 10 |
| 73 | 15 | 3.2 | 15 | 3.2 | 14.47 | 3.09 | 10 |  | 14.3 | 2.2 | 14.3 | 2.2 | 13.74 | 2.11 | 10 |
| 74 | 15 | 3.2 | 15 | 3.2 | 14.46 | 3.08 | 10 |  | 14.3 | 2.2 | 14.3 | 2.2 | 13.74 | 2.11 | 10 |
| 75 | N/A | N/A | 14.99 | 3.19 | 14.47 | 3.08 | 10 |  | N/A | N/A | 14.3 | 2.2 | 13.74 | 2.11 | 10 |
| 76 | N/A | N/A | 14.99 | 3.19 | 14.47 | 3.08 | 10 |  | N/A | N/A | 14.3 | 2.2 | 13.74 | 2.12 | 10 |
| 77 | N/A | N/A | 14.99 | 3.18 | 14.46 | 3.07 | 10 |  | N/A | N/A | 14.3 | 2.2 | 13.74 | 2.12 | 10 |
| 78 | N/A | N/A | 14.98 | 3.18 | 14.46 | 3.07 | 10 |  | N/A | N/A | 14.3 | 2.2 | 13.74 | 2.12 | 10 |
| 79 | N/A | N/A | 14.98 | 3.17 | 14.46 | 3.06 | 10 |  | N/A | N/A | 14.3 | 2.21 | 13.74 | 2.12 | 10 |
| 80 | N/A | N/A | 14.97 | 3.17 | 14.47 | 3.06 | 10 |  | N/A | N/A | 14.3 | 2.21 | 13.76 | 2.12 | 10 |
| 81 | N/A | N/A | 14.97 | 3.16 | 14.46 | 3.05 | 10 |  | N/A | N/A | 14.3 | 2.21 | 13.76 | 2.12 | 10 |
| 82 | N/A | N/A | 14.97 | 3.16 | 14.46 | 3.05 | 10 |  | N/A | N/A | 14.3 | 2.21 | 13.76 | 2.13 | 10 |
| 83 | N/A | N/A | 14.96 | 3.15 | 14.45 | 3.04 | 10 |  | N/A | N/A | 14.3 | 2.21 | 13.76 | 2.13 | 10 |
| 84 | N/A | N/A | 14.96 | 3.15 | 14.45 | 3.04 | 10 |  | N/A | N/A | 14.3 | 2.21 | 13.76 | 2.13 | 10 |
| 85 | N/A | N/A | 14.95 | 3.14 | 14.47 | 3.04 | 10 |  | N/A | N/A | 14.3 | 2.21 | 13.78 | 2.13 | 10 |
| 86 | N/A | N/A | 14.95 | 3.14 | 14.47 | 3.03 | 10 |  | N/A | N/A | 14.3 | 2.21 | 13.78 | 2.13 | 10 |
| 87 | N/A | N/A | 14.95 | 3.13 | 14.46 | 3.03 | 10 |  | N/A | N/A | 14.3 | 2.21 | 13.78 | 2.13 | 10 |
| 88 | N/A | N/A | 14.94 | 3.13 | 14.46 | 3.02 | 10 |  | N/A | N/A | 14.3 | 2.21 | 13.78 | 2.13 | 10 |
| 89 | N/A | N/A | 14.94 | 3.12 | 14.46 | 3.02 | 10 |  | N/A | N/A | 14.3 | 2.22 | 13.78 | 2.13 | 10 |
| 90 | N/A | N/A | 14.93 | 3.12 | 14.45 | 3.01 | 10 |  | N/A | N/A | 14.3 | 2.22 | 13.78 | 2.14 | 10 |
| 91 | N/A | N/A | 14.93 | 3.11 | 14.45 | 3.01 | 10 |  | N/A | N/A | 14.3 | 2.22 | 13.78 | 2.14 | 10 |
| 92 | N/A | N/A | 14.93 | 3.1 | 14.44 | 3 | 10 |  | N/A | N/A | 14.3 | 2.22 | 13.78 | 2.14 | 10 |
| 93 | N/A | N/A | 14.92 | 3.1 | 14.44 | 3 | 10 |  | N/A | N/A | 14.3 | 2.22 | 13.78 | 2.14 | 10 |
| 94 | N/A | N/A | 14.92 | 3.09 | 14.44 | 2.99 | 10 |  | N/A | N/A | 14.3 | 2.22 | 13.78 | 2.14 | 10 |
| 95 | N/A | N/A | 14.91 | 3.09 | 14.43 | 2.99 | 10 |  | N/A | N/A | 14.3 | 2.22 | 13.78 | 2.14 | 10 |

SD =Standard deviation, N/A = Not available
