# Supplementary material for: Substituting polyunsaturated fat for saturated fat: A health impact assessment of a fat tax in seven European countries
Source: PLoS One. 2019 Jul 10;14(7):e0218464. doi: 10.1371/journal.pone.0218464 (PMC6619676; doi:10.1371/journal.pone.0218464)
Supplement: S9 Table — (DOCX) [file pone.0218464.s009.docx]

# S9 Table. Saturated fat intake (mean and standard deviation) across scenarios in the UK.

| Age | Males | | | | | | |  | Females | | | | | | |
| --- | --- | --- | --- | --- | --- | --- | --- | --- | --- | --- | --- | --- | --- | --- | --- |
|  | Original | | Reference scenario | | Fat tax scenario | | Guideline scenario |  | Original | | Reference scenario | | Fat tax scenario | | Guideline scenario |
|  | Mean | SD | Mean | SD | Mean | SD | Mean |  | Mean | SD | Mean | SD | Mean | SD | Mean |
| 0 | N/A | N/A | 14.49 | 2.9 | 14.49 | 2.9 | 10 |  | N/A | N/A | 14.38 | 3.01 | 14.38 | 3.01 | 10 |
| 1 | N/A | N/A | 14.49 | 2.9 | 14.49 | 2.9 | 10 |  | N/A | N/A | 14.38 | 3.01 | 14.38 | 3.01 | 10 |
| 2 | 14.5 | 2.9 | 14.49 | 2.9 | 14.49 | 2.9 | 10 |  | 14.4 | 3 | 14.39 | 3 | 14.39 | 3 | 10 |
| 3 | 14.5 | 2.9 | 14.5 | 2.9 | 14.5 | 2.9 | 10 |  | 14.4 | 3 | 14.4 | 3 | 14.4 | 3 | 10 |
| 4 | 14.5 | 2.9 | 14.5 | 2.9 | 14.5 | 2.9 | 10 |  | 14.4 | 3 | 14.41 | 3 | 14.41 | 3 | 10 |
| 5 | 14.5 | 2.9 | 14.51 | 2.9 | 14.51 | 2.9 | 10 |  | 14.4 | 3 | 14.42 | 3 | 14.42 | 3 | 10 |
| 6 | 14.5 | 2.9 | 14.52 | 2.9 | 14.52 | 2.9 | 10 |  | 14.4 | 3 | 14.43 | 2.99 | 14.43 | 2.99 | 10 |
| 7 | 14.5 | 2.9 | 14.52 | 2.9 | 14.52 | 2.9 | 10 |  | 14.4 | 3 | 14.44 | 2.99 | 14.44 | 2.99 | 10 |
| 8 | 14.5 | 2.9 | 14.53 | 2.9 | 14.53 | 2.9 | 10 |  | 14.4 | 3 | 14.45 | 2.98 | 14.45 | 2.98 | 10 |
| 9 | 14.5 | 2.9 | 14.53 | 2.9 | 14.53 | 2.9 | 10 |  | 14.4 | 3 | 14.45 | 2.98 | 14.45 | 2.98 | 10 |
| 10 | 14.5 | 2.9 | 14.53 | 2.9 | 14.53 | 2.9 | 10 |  | 14.4 | 3 | 14.44 | 2.99 | 14.44 | 2.99 | 10 |
| 11 | 14.5 | 2.9 | 14.5 | 2.9 | 14.5 | 2.9 | 10 |  | 14.4 | 3 | 14.41 | 3 | 14.41 | 3 | 10 |
| 12 | 14.5 | 2.9 | 14.46 | 2.9 | 14.46 | 2.9 | 10 |  | 14.4 | 3 | 14.33 | 3.02 | 14.33 | 3.02 | 10 |
| 13 | 14.5 | 2.9 | 14.38 | 2.91 | 14.38 | 2.91 | 10 |  | 14.4 | 3 | 14.2 | 3.06 | 14.2 | 3.06 | 10 |
| 14 | 14.5 | 2.9 | 14.27 | 2.92 | 14.27 | 2.92 | 10 |  | 14.4 | 3 | 14.01 | 3.11 | 14.01 | 3.11 | 10 |
| 15 | 14.5 | 2.9 | 14.13 | 2.94 | 13.46 | 2.8 | 10 |  | 14.4 | 3 | 13.77 | 3.18 | 13.33 | 3.08 | 10 |
| 16 | 13.6 | 3 | 13.97 | 2.95 | 13.3 | 2.81 | 10 |  | 12.9 | 3.4 | 13.49 | 3.25 | 13.06 | 3.14 | 10 |
| 17 | 13.6 | 3 | 13.82 | 2.96 | 13.15 | 2.82 | 10 |  | 12.9 | 3.4 | 13.24 | 3.31 | 12.81 | 3.2 | 10 |
| 18 | 13.6 | 3 | 13.71 | 2.97 | 13.04 | 2.83 | 10 |  | 12.9 | 3.4 | 13.06 | 3.35 | 12.62 | 3.24 | 10 |
| 19 | 13.6 | 3 | 13.63 | 2.98 | 12.96 | 2.83 | 10 |  | 12.9 | 3.4 | 12.94 | 3.38 | 12.48 | 3.26 | 10 |
| 20 | 13.6 | 3 | 13.59 | 3 | 12.93 | 2.85 | 10 |  | 12.9 | 3.4 | 12.89 | 3.4 | 12.43 | 3.28 | 10 |
| 21 | 13.6 | 3 | 13.58 | 3.02 | 12.92 | 2.88 | 10 |  | 12.9 | 3.4 | 12.9 | 3.42 | 12.45 | 3.3 | 10 |
| 22 | 13.6 | 3 | 13.6 | 3.07 | 12.93 | 2.92 | 10 |  | 12.9 | 3.4 | 12.98 | 3.45 | 12.52 | 3.33 | 10 |
| 23 | 13.6 | 3 | 13.62 | 3.13 | 12.96 | 2.97 | 10 |  | 12.9 | 3.4 | 13.11 | 3.49 | 12.65 | 3.37 | 10 |
| 24 | 13.6 | 3 | 13.66 | 3.21 | 13 | 3.05 | 10 |  | 12.9 | 3.4 | 13.28 | 3.54 | 12.83 | 3.42 | 10 |
| 25 | 13.8 | 3.5 | 13.71 | 3.29 | 13.06 | 3.14 | 10 |  | 13.9 | 3.7 | 13.47 | 3.59 | 13 | 3.47 | 10 |
| 26 | 13.8 | 3.5 | 13.75 | 3.37 | 13.1 | 3.21 | 10 |  | 13.9 | 3.7 | 13.64 | 3.64 | 13.18 | 3.52 | 10 |
| 27 | 13.8 | 3.5 | 13.78 | 3.43 | 13.13 | 3.27 | 10 |  | 13.9 | 3.7 | 13.78 | 3.68 | 13.31 | 3.55 | 10 |
| 28 | 13.8 | 3.5 | 13.8 | 3.47 | 13.15 | 3.31 | 10 |  | 13.9 | 3.7 | 13.87 | 3.7 | 13.4 | 3.58 | 10 |
| 29 | 13.8 | 3.5 | 13.81 | 3.5 | 13.16 | 3.33 | 10 |  | 13.9 | 3.7 | 13.92 | 3.72 | 13.45 | 3.59 | 10 |
| 30 | 13.8 | 3.5 | 13.81 | 3.51 | 13.17 | 3.35 | 10 |  | 13.9 | 3.7 | 13.93 | 3.71 | 13.44 | 3.58 | 10 |
| 31 | 13.8 | 3.5 | 13.8 | 3.52 | 13.17 | 3.36 | 10 |  | 13.9 | 3.7 | 13.92 | 3.69 | 13.42 | 3.56 | 10 |
| 32 | 13.8 | 3.5 | 13.78 | 3.53 | 13.15 | 3.37 | 10 |  | 13.9 | 3.7 | 13.88 | 3.64 | 13.39 | 3.51 | 10 |
| 33 | 13.8 | 3.5 | 13.75 | 3.54 | 13.12 | 3.37 | 10 |  | 13.9 | 3.7 | 13.82 | 3.58 | 13.33 | 3.45 | 10 |
| 34 | 13.8 | 3.5 | 13.72 | 3.55 | 13.09 | 3.38 | 10 |  | 13.9 | 3.7 | 13.75 | 3.49 | 13.26 | 3.37 | 10 |
| 35 | 13.6 | 3.6 | 13.68 | 3.56 | 13.05 | 3.4 | 10 |  | 13.5 | 3.2 | 13.67 | 3.4 | 13.18 | 3.28 | 10 |
| 36 | 13.6 | 3.6 | 13.65 | 3.58 | 13.01 | 3.41 | 10 |  | 13.5 | 3.2 | 13.6 | 3.32 | 13.11 | 3.19 | 10 |
| 37 | 13.6 | 3.6 | 13.62 | 3.59 | 12.99 | 3.42 | 10 |  | 13.5 | 3.2 | 13.55 | 3.25 | 13.05 | 3.13 | 10 |
| 38 | 13.6 | 3.6 | 13.6 | 3.59 | 12.97 | 3.43 | 10 |  | 13.5 | 3.2 | 13.51 | 3.21 | 13.02 | 3.09 | 10 |
| 39 | 13.6 | 3.6 | 13.59 | 3.6 | 12.96 | 3.43 | 10 |  | 13.5 | 3.2 | 13.49 | 3.19 | 13 | 3.07 | 10 |
| 40 | 13.6 | 3.6 | 13.59 | 3.6 | 12.98 | 3.44 | 10 |  | 13.5 | 3.2 | 13.49 | 3.19 | 12.96 | 3.06 | 10 |
| 41 | 13.6 | 3.6 | 13.6 | 3.61 | 12.98 | 3.44 | 10 |  | 13.5 | 3.2 | 13.49 | 3.2 | 12.97 | 3.08 | 10 |
| 42 | 13.6 | 3.6 | 13.62 | 3.62 | 13 | 3.45 | 10 |  | 13.5 | 3.2 | 13.51 | 3.23 | 12.99 | 3.11 | 10 |
| 43 | 13.6 | 3.6 | 13.65 | 3.63 | 13.03 | 3.46 | 10 |  | 13.5 | 3.2 | 13.54 | 3.28 | 13.02 | 3.16 | 10 |
| 44 | 13.6 | 3.6 | 13.68 | 3.64 | 13.06 | 3.48 | 10 |  | 13.5 | 3.2 | 13.57 | 3.35 | 13.05 | 3.22 | 10 |
| 45 | 13.8 | 3.7 | 13.72 | 3.66 | 13.1 | 3.49 | 10 |  | 13.7 | 3.6 | 13.61 | 3.42 | 13.09 | 3.29 | 10 |
| 46 | 13.8 | 3.7 | 13.75 | 3.67 | 13.13 | 3.51 | 10 |  | 13.7 | 3.6 | 13.65 | 3.48 | 13.12 | 3.35 | 10 |
| 47 | 13.8 | 3.7 | 13.78 | 3.68 | 13.16 | 3.52 | 10 |  | 13.7 | 3.6 | 13.67 | 3.53 | 13.15 | 3.39 | 10 |
| 48 | 13.8 | 3.7 | 13.8 | 3.69 | 13.18 | 3.53 | 10 |  | 13.7 | 3.6 | 13.69 | 3.56 | 13.17 | 3.42 | 10 |
| 49 | 13.8 | 3.7 | 13.8 | 3.7 | 13.19 | 3.53 | 10 |  | 13.7 | 3.6 | 13.7 | 3.58 | 13.18 | 3.45 | 10 |
| 50 | 13.8 | 3.7 | 13.81 | 3.7 | 13.22 | 3.54 | 10 |  | 13.7 | 3.6 | 13.71 | 3.61 | 13.17 | 3.47 | 10 |
| 51 | 13.8 | 3.7 | 13.8 | 3.71 | 13.21 | 3.55 | 10 |  | 13.7 | 3.6 | 13.71 | 3.65 | 13.18 | 3.5 | 10 |
| 52 | 13.8 | 3.7 | 13.79 | 3.72 | 13.2 | 3.56 | 10 |  | 13.7 | 3.6 | 13.72 | 3.7 | 13.18 | 3.56 | 10 |
| 53 | 13.8 | 3.7 | 13.78 | 3.73 | 13.19 | 3.57 | 10 |  | 13.7 | 3.6 | 13.73 | 3.79 | 13.19 | 3.64 | 10 |
| 54 | 13.8 | 3.7 | 13.76 | 3.74 | 13.17 | 3.58 | 10 |  | 13.7 | 3.6 | 13.74 | 3.89 | 13.21 | 3.74 | 10 |
| 55 | 13.7 | 3.8 | 13.73 | 3.76 | 13.14 | 3.59 | 10 |  | 13.8 | 4.3 | 13.76 | 4.02 | 13.21 | 3.86 | 10 |
| 56 | 13.7 | 3.8 | 13.71 | 3.77 | 13.12 | 3.61 | 10 |  | 13.8 | 4.3 | 13.77 | 4.13 | 13.23 | 3.96 | 10 |
| 57 | 13.7 | 3.8 | 13.69 | 3.78 | 13.1 | 3.62 | 10 |  | 13.8 | 4.3 | 13.79 | 4.21 | 13.24 | 4.05 | 10 |
| 58 | 13.7 | 3.8 | 13.68 | 3.79 | 13.09 | 3.62 | 10 |  | 13.8 | 4.3 | 13.79 | 4.27 | 13.24 | 4.1 | 10 |
| 59 | 13.7 | 3.8 | 13.68 | 3.79 | 13.09 | 3.63 | 10 |  | 13.8 | 4.3 | 13.8 | 4.31 | 13.25 | 4.13 | 10 |
| 60 | 13.7 | 3.8 | 13.69 | 3.8 | 13.12 | 3.64 | 10 |  | 13.8 | 4.3 | 13.8 | 4.32 | 13.24 | 4.14 | 10 |
| 61 | 13.7 | 3.8 | 13.73 | 3.82 | 13.15 | 3.66 | 10 |  | 13.8 | 4.3 | 13.81 | 4.32 | 13.25 | 4.14 | 10 |
| 62 | 13.7 | 3.8 | 13.79 | 3.84 | 13.21 | 3.68 | 10 |  | 13.8 | 4.3 | 13.81 | 4.31 | 13.25 | 4.13 | 10 |
| 63 | 13.7 | 3.8 | 13.87 | 3.88 | 13.3 | 3.72 | 10 |  | 13.8 | 4.3 | 13.82 | 4.29 | 13.26 | 4.12 | 10 |
| 64 | 13.7 | 3.8 | 13.98 | 3.92 | 13.41 | 3.76 | 10 |  | 13.8 | 4.3 | 13.83 | 4.27 | 13.27 | 4.1 | 10 |
| 65 | 14.4 | 4.1 | 14.11 | 3.98 | 13.56 | 3.82 | 10 |  | 13.9 | 4.2 | 13.84 | 4.25 | 13.26 | 4.07 | 10 |
| 66 | 14.4 | 4.1 | 14.22 | 4.03 | 13.67 | 3.87 | 10 |  | 13.9 | 4.2 | 13.85 | 4.23 | 13.27 | 4.05 | 10 |
| 67 | 14.4 | 4.1 | 14.31 | 4.06 | 13.76 | 3.91 | 10 |  | 13.9 | 4.2 | 13.85 | 4.21 | 13.27 | 4.04 | 10 |
| 68 | 14.4 | 4.1 | 14.37 | 4.09 | 13.82 | 3.93 | 10 |  | 13.9 | 4.2 | 13.86 | 4.2 | 13.28 | 4.03 | 10 |
| 69 | 14.4 | 4.1 | 14.4 | 4.1 | 13.85 | 3.95 | 10 |  | 13.9 | 4.2 | 13.87 | 4.2 | 13.29 | 4.02 | 10 |
| 70 | 14.4 | 4.1 | 14.42 | 4.11 | 13.88 | 3.96 | 10 |  | 13.9 | 4.2 | 13.9 | 4.2 | 13.33 | 4.03 | 10 |
| 71 | 14.4 | 4.1 | 14.43 | 4.11 | 13.89 | 3.95 | 10 |  | 13.9 | 4.2 | 13.95 | 4.2 | 13.39 | 4.03 | 10 |
| 72 | 14.4 | 4.1 | 14.43 | 4.1 | 13.9 | 3.94 | 10 |  | 13.9 | 4.2 | 14.04 | 4.2 | 13.48 | 4.03 | 10 |
| 73 | 14.4 | 4.1 | 14.44 | 4.08 | 13.91 | 3.93 | 10 |  | 13.9 | 4.2 | 14.18 | 4.2 | 13.61 | 4.03 | 10 |
| 74 | 14.4 | 4.1 | 14.45 | 4.06 | 13.92 | 3.91 | 10 |  | 13.9 | 4.2 | 14.35 | 4.2 | 13.79 | 4.04 | 10 |
| 75 | 14.5 | 4 | 14.47 | 4.04 | 13.94 | 3.9 | 10 |  | 15 | 4.2 | 14.55 | 4.2 | 13.98 | 4.04 | 10 |
| 76 | 14.5 | 4 | 14.48 | 4.03 | 13.96 | 3.88 | 10 |  | 15 | 4.2 | 14.73 | 4.2 | 14.16 | 4.04 | 10 |
| 77 | 14.5 | 4 | 14.49 | 4.01 | 13.97 | 3.87 | 10 |  | 15 | 4.2 | 14.86 | 4.2 | 14.3 | 4.04 | 10 |
| 78 | 14.5 | 4 | 14.5 | 4 | 13.97 | 3.86 | 10 |  | 15 | 4.2 | 14.95 | 4.2 | 14.39 | 4.04 | 10 |
| 79 | 14.5 | 4 | 14.5 | 4 | 13.98 | 3.86 | 10 |  | 15 | 4.2 | 15 | 4.2 | 14.44 | 4.04 | 10 |
| 80 | 14.5 | 4 | 14.5 | 4 | 13.99 | 3.86 | 10 |  | 15 | 4.2 | 15.03 | 4.2 | 14.49 | 4.05 | 10 |
| 81 | 14.5 | 4 | 14.5 | 4 | 13.99 | 3.86 | 10 |  | 15 | 4.2 | 15.04 | 4.2 | 14.5 | 4.05 | 10 |
| 82 | 14.5 | 4 | 14.5 | 4 | 13.99 | 3.86 | 10 |  | 15 | 4.2 | 15.03 | 4.2 | 14.5 | 4.05 | 10 |
| 83 | 14.5 | 4 | 14.5 | 4 | 13.99 | 3.86 | 10 |  | 15 | 4.2 | 15.03 | 4.2 | 14.49 | 4.05 | 10 |
| 84 | 14.5 | 4 | 14.5 | 4 | 13.99 | 3.86 | 10 |  | 15 | 4.2 | 15.02 | 4.2 | 14.48 | 4.05 | 10 |
| 85 | 14.5 | 4 | 14.5 | 4 | 14.02 | 3.87 | 10 |  | 15 | 4.2 | 15.01 | 4.2 | 14.49 | 4.05 | 10 |
| 86 | 14.5 | 4 | 14.5 | 4 | 14.02 | 3.87 | 10 |  | 15 | 4.2 | 15.01 | 4.2 | 14.48 | 4.05 | 10 |
| 87 | 14.5 | 4 | 14.5 | 4 | 14.02 | 3.87 | 10 |  | 15 | 4.2 | 15 | 4.2 | 14.48 | 4.05 | 10 |
| 88 | 14.5 | 4 | 14.5 | 4 | 14.02 | 3.87 | 10 |  | 15 | 4.2 | 15 | 4.2 | 14.48 | 4.05 | 10 |
| 89 | 14.5 | 4 | 14.5 | 4 | 14.02 | 3.87 | 10 |  | 15 | 4.2 | 15 | 4.2 | 14.48 | 4.05 | 10 |
| 90 | 14.5 | 4 | 14.5 | 4 | 14.02 | 3.87 | 10 |  | 15 | 4.2 | 15 | 4.2 | 14.48 | 4.05 | 10 |
| 91 | 14.5 | 4 | 14.5 | 4 | 14.02 | 3.87 | 10 |  | 15 | 4.2 | 15 | 4.2 | 14.48 | 4.05 | 10 |
| 92 | 14.5 | 4 | 14.5 | 4 | 14.02 | 3.87 | 10 |  | 15 | 4.2 | 15 | 4.2 | 14.48 | 4.05 | 10 |
| 93 | 14.5 | 4 | 14.5 | 4 | 14.02 | 3.87 | 10 |  | 15 | 4.2 | 15 | 4.2 | 14.48 | 4.05 | 10 |
| 94 | 14.5 | 4 | 14.5 | 4 | 14.02 | 3.87 | 10 |  | 15 | 4.2 | 15 | 4.2 | 14.48 | 4.05 | 10 |
| 95 | 14.5 | 4 | 14.5 | 4 | 14.02 | 3.87 | 10 |  | 15 | 4.2 | 15 | 4.2 | 14.48 | 4.05 | 10 |

SD = Standard deviation, N/A = Not available
