# Supplementary material for: Substituting polyunsaturated fat for saturated fat: A health impact assessment of a fat tax in seven European countries
Source: PLoS One. 2019 Jul 10;14(7):e0218464. doi: 10.1371/journal.pone.0218464 (PMC6619676; doi:10.1371/journal.pone.0218464)
Supplement: S10 Table — (DOCX) [file pone.0218464.s010.docx]

# S10 Table. Proportion of persons in the respective saturated fat intake categories across scenarios in Denmark.

| Age | Reference scenario^a^ | | | | | | | | | |  | Fat tax scenario^a^ | | | | | | | | | |  | Guideline scenario | |
| --- | --- | --- | --- | --- | --- | --- | --- | --- | --- | --- | --- | --- | --- | --- | --- | --- | --- | --- | --- | --- | --- | --- | --- | --- |
|  | Category of saturated fat intake (in %E)^b^ | | | | | | | | | |  | Category of saturated fat intake (in %E)^b^ | | | | | | | | | |  | Category of saturated fat intake (in %E)^b^ | |
|  | ≤10 | >10 ≤12 | >12 ≤14 | >14 ≤16 | >16 ≤18 | >18 ≤20 | >20 ≤22 | >22 ≤24 | >24 ≤26 | >26 ≤100 |  | ≤10 | >10 ≤12 | >12 ≤14 | >14 ≤16 | >16 ≤18 | >18 ≤20 | >20 ≤22 | >22 ≤24 | >24 ≤26 | >26 ≤100 |  | ≤10 | >10 ≤100 |
|  |  |  | Males | | | | | | | | | | | | | | | | | | | | | |
| 0 | 100 | 0 | 0 | 0 | 0 | 0 | 0 | 0 | 0 | 0 |  | 100 | 0 | 0 | 0 | 0 | 0 | 0 | 0 | 0 | 0 |  | 100 | 0 |
| 1 | 100 | 0 | 0 | 0 | 0 | 0 | 0 | 0 | 0 | 0 |  | 100 | 0 | 0 | 0 | 0 | 0 | 0 | 0 | 0 | 0 |  | 100 | 0 |
| 2 | 100 | 0 | 0 | 0 | 0 | 0 | 0 | 0 | 0 | 0 |  | 100 | 0 | 0 | 0 | 0 | 0 | 0 | 0 | 0 | 0 |  | 100 | 0 |
| 3 | 100 | 0 | 0 | 0 | 0 | 0 | 0 | 0 | 0 | 0 |  | 100 | 0 | 0 | 0 | 0 | 0 | 0 | 0 | 0 | 0 |  | 100 | 0 |
| 4 | 100 | 0 | 0 | 0 | 0 | 0 | 0 | 0 | 0 | 0 |  | 100 | 0 | 0 | 0 | 0 | 0 | 0 | 0 | 0 | 0 |  | 100 | 0 |
| 5 | 100 | 0 | 0 | 0 | 0 | 0 | 0 | 0 | 0 | 0 |  | 100 | 0 | 0 | 0 | 0 | 0 | 0 | 0 | 0 | 0 |  | 100 | 0 |
| 6 | 0.95 | 7.08 | 24.26 | 36.29 | 23.75 | 6.79 | 0.84 | 0.04 | 0 | 0 |  | 0.95 | 7.08 | 24.26 | 36.29 | 23.75 | 6.79 | 0.84 | 0.04 | 0 | 0 |  | 100 | 0 |
| 7 | 1.04 | 7.61 | 25.31 | 36.41 | 22.73 | 6.14 | 0.71 | 0.03 | 0 | 0 |  | 1.04 | 7.61 | 25.31 | 36.41 | 22.73 | 6.14 | 0.71 | 0.03 | 0 | 0 |  | 100 | 0 |
| 8 | 1.25 | 8.51 | 26.59 | 36.13 | 21.38 | 5.5 | 0.61 | 0.03 | 0 | 0 |  | 1.25 | 8.51 | 26.59 | 36.13 | 21.38 | 5.5 | 0.61 | 0.03 | 0 | 0 |  | 100 | 0 |
| 9 | 1.64 | 9.86 | 27.99 | 35.28 | 19.76 | 4.9 | 0.53 | 0.03 | 0 | 0 |  | 1.64 | 9.86 | 27.99 | 35.28 | 19.76 | 4.9 | 0.53 | 0.03 | 0 | 0 |  | 100 | 0 |
| 10 | 2.22 | 11.45 | 29.19 | 34.04 | 18.17 | 4.42 | 0.49 | 0.02 | 0 | 0 |  | 2.22 | 11.45 | 29.19 | 34.04 | 18.17 | 4.42 | 0.49 | 0.02 | 0 | 0 |  | 100 | 0 |
| 11 | 2.92 | 12.97 | 29.95 | 32.68 | 16.87 | 4.11 | 0.47 | 0.02 | 0 | 0 |  | 2.92 | 12.97 | 29.95 | 32.68 | 16.87 | 4.11 | 0.47 | 0.02 | 0 | 0 |  | 100 | 0 |
| 12 | 3.63 | 14.19 | 30.21 | 31.46 | 16.02 | 3.98 | 0.48 | 0.03 | 0 | 0 |  | 3.63 | 14.19 | 30.21 | 31.46 | 16.02 | 3.98 | 0.48 | 0.03 | 0 | 0 |  | 100 | 0 |
| 13 | 4.31 | 15.04 | 30.07 | 30.42 | 15.57 | 4.03 | 0.52 | 0.03 | 0 | 0 |  | 4.31 | 15.04 | 30.07 | 30.42 | 15.57 | 4.03 | 0.52 | 0.03 | 0 | 0 |  | 100 | 0 |
| 14 | 4.94 | 15.56 | 29.63 | 29.56 | 15.45 | 4.22 | 0.6 | 0.04 | 0 | 0 |  | 4.94 | 15.56 | 29.63 | 29.56 | 15.45 | 4.22 | 0.6 | 0.04 | 0 | 0 |  | 100 | 0 |
| 15 | 5.48 | 15.82 | 29.02 | 28.84 | 15.54 | 4.53 | 0.71 | 0.06 | 0 | 0 |  | 8.11 | 20.89 | 32.45 | 25.74 | 10.43 | 2.15 | 0.22 | 0.01 | 0 | 0 |  | 100 | 0 |
| 16 | 5.92 | 15.88 | 28.34 | 28.27 | 15.76 | 4.9 | 0.85 | 0.08 | 0 | 0 |  | 8.61 | 20.73 | 31.62 | 25.47 | 10.83 | 2.43 | 0.29 | 0.02 | 0 | 0 |  | 100 | 0 |
| 17 | 6.23 | 15.79 | 27.68 | 27.83 | 16.05 | 5.3 | 1 | 0.11 | 0.01 | 0 |  | 8.95 | 20.46 | 30.88 | 25.33 | 11.28 | 2.72 | 0.36 | 0.02 | 0 | 0 |  | 100 | 0 |
| 18 | 6.45 | 15.61 | 27.05 | 27.49 | 16.37 | 5.71 | 1.17 | 0.14 | 0.01 | 0 |  | 9.16 | 20.1 | 30.21 | 25.26 | 11.76 | 3.04 | 0.44 | 0.03 | 0 | 0 |  | 100 | 0 |
| 19 | 6.59 | 15.41 | 26.5 | 27.21 | 16.68 | 6.1 | 1.33 | 0.17 | 0.01 | 0 |  | 9.32 | 19.82 | 29.66 | 25.19 | 12.13 | 3.31 | 0.51 | 0.04 | 0 | 0 |  | 100 | 0 |
| 20 | 6.68 | 15.22 | 26.06 | 27 | 16.93 | 6.42 | 1.47 | 0.2 | 0.02 | 0 |  | 9.32 | 19.42 | 29.16 | 25.24 | 12.59 | 3.62 | 0.6 | 0.06 | 0 | 0 |  | 100 | 0 |
| 21 | 6.72 | 15.08 | 25.76 | 26.87 | 17.11 | 6.64 | 1.57 | 0.23 | 0.02 | 0 |  | 9.34 | 19.2 | 28.85 | 25.24 | 12.85 | 3.8 | 0.65 | 0.06 | 0 | 0 |  | 100 | 0 |
| 22 | 6.73 | 14.98 | 25.58 | 26.8 | 17.22 | 6.79 | 1.64 | 0.24 | 0.02 | 0 |  | 9.34 | 19.05 | 28.67 | 25.25 | 13.01 | 3.92 | 0.69 | 0.07 | 0 | 0 |  | 100 | 0 |
| 23 | 6.73 | 14.92 | 25.49 | 26.77 | 17.29 | 6.86 | 1.67 | 0.25 | 0.02 | 0 |  | 9.32 | 18.97 | 28.57 | 25.26 | 13.1 | 3.98 | 0.71 | 0.07 | 0 | 0 |  | 100 | 0 |
| 24 | 6.72 | 14.89 | 25.45 | 26.76 | 17.32 | 6.89 | 1.69 | 0.25 | 0.02 | 0 |  | 9.3 | 18.94 | 28.54 | 25.27 | 13.14 | 4.01 | 0.72 | 0.08 | 0 | 0 |  | 100 | 0 |
| 25 | 6.71 | 14.89 | 25.45 | 26.77 | 17.32 | 6.89 | 1.68 | 0.25 | 0.02 | 0 |  | 9.22 | 18.83 | 28.49 | 25.34 | 13.25 | 4.07 | 0.73 | 0.08 | 0 | 0 |  | 100 | 0 |
| 26 | 6.7 | 14.89 | 25.47 | 26.79 | 17.32 | 6.88 | 1.68 | 0.25 | 0.02 | 0 |  | 9.21 | 18.83 | 28.51 | 25.35 | 13.24 | 4.06 | 0.73 | 0.08 | 0 | 0 |  | 100 | 0 |
| 27 | 6.69 | 14.89 | 25.49 | 26.8 | 17.31 | 6.87 | 1.67 | 0.25 | 0.02 | 0 |  | 9.2 | 18.85 | 28.53 | 25.35 | 13.22 | 4.04 | 0.72 | 0.08 | 0 | 0 |  | 100 | 0 |
| 28 | 6.68 | 14.9 | 25.52 | 26.82 | 17.3 | 6.85 | 1.66 | 0.25 | 0.02 | 0 |  | 9.19 | 18.86 | 28.56 | 25.36 | 13.2 | 4.03 | 0.72 | 0.08 | 0 | 0 |  | 100 | 0 |
| 29 | 6.68 | 14.91 | 25.53 | 26.82 | 17.29 | 6.84 | 1.66 | 0.24 | 0.02 | 0 |  | 9.19 | 18.87 | 28.57 | 25.36 | 13.19 | 4.02 | 0.72 | 0.07 | 0 | 0 |  | 100 | 0 |
| 30 | 6.68 | 14.92 | 25.54 | 26.83 | 17.29 | 6.83 | 1.65 | 0.24 | 0.02 | 0 |  | 9.12 | 18.78 | 28.52 | 25.41 | 13.28 | 4.07 | 0.73 | 0.08 | 0 | 0 |  | 100 | 0 |
| 31 | 6.68 | 14.92 | 25.55 | 26.83 | 17.28 | 6.82 | 1.65 | 0.24 | 0.02 | 0 |  | 9.12 | 18.79 | 28.53 | 25.41 | 13.28 | 4.06 | 0.73 | 0.08 | 0 | 0 |  | 100 | 0 |
| 32 | 6.68 | 14.92 | 25.55 | 26.83 | 17.28 | 6.82 | 1.65 | 0.24 | 0.02 | 0 |  | 9.12 | 18.79 | 28.54 | 25.41 | 13.27 | 4.06 | 0.73 | 0.08 | 0 | 0 |  | 100 | 0 |
| 33 | 6.68 | 14.92 | 25.56 | 26.83 | 17.28 | 6.82 | 1.65 | 0.24 | 0.02 | 0 |  | 9.12 | 18.79 | 28.54 | 25.41 | 13.27 | 4.06 | 0.73 | 0.08 | 0 | 0 |  | 100 | 0 |
| 34 | 6.68 | 14.92 | 25.55 | 26.83 | 17.28 | 6.82 | 1.65 | 0.24 | 0.02 | 0 |  | 9.12 | 18.79 | 28.54 | 25.41 | 13.27 | 4.06 | 0.73 | 0.08 | 0 | 0 |  | 100 | 0 |
| 35 | 6.68 | 14.92 | 25.55 | 26.83 | 17.28 | 6.82 | 1.65 | 0.24 | 0.02 | 0 |  | 9.12 | 18.78 | 28.53 | 25.42 | 13.28 | 4.06 | 0.73 | 0.08 | 0 | 0 |  | 100 | 0 |
| 36 | 6.68 | 14.92 | 25.55 | 26.83 | 17.28 | 6.82 | 1.65 | 0.24 | 0.02 | 0 |  | 9.12 | 18.78 | 28.53 | 25.42 | 13.28 | 4.07 | 0.73 | 0.08 | 0 | 0 |  | 100 | 0 |
| 37 | 6.68 | 14.92 | 25.55 | 26.83 | 17.28 | 6.82 | 1.65 | 0.24 | 0.02 | 0 |  | 9.12 | 18.78 | 28.53 | 25.42 | 13.28 | 4.07 | 0.73 | 0.08 | 0 | 0 |  | 100 | 0 |
| 38 | 6.68 | 14.92 | 25.55 | 26.83 | 17.28 | 6.82 | 1.65 | 0.24 | 0.02 | 0 |  | 9.12 | 18.78 | 28.53 | 25.42 | 13.28 | 4.07 | 0.73 | 0.08 | 0 | 0 |  | 100 | 0 |
| 39 | 6.68 | 14.92 | 25.55 | 26.83 | 17.28 | 6.82 | 1.65 | 0.24 | 0.02 | 0 |  | 9.12 | 18.78 | 28.53 | 25.42 | 13.28 | 4.07 | 0.73 | 0.08 | 0 | 0 |  | 100 | 0 |
| 40 | 6.68 | 14.92 | 25.55 | 26.83 | 17.28 | 6.82 | 1.65 | 0.24 | 0.02 | 0 |  | 9.05 | 18.68 | 28.47 | 25.47 | 13.38 | 4.12 | 0.74 | 0.08 | 0 | 0 |  | 100 | 0 |
| 41 | 6.68 | 14.92 | 25.55 | 26.83 | 17.28 | 6.82 | 1.65 | 0.24 | 0.02 | 0 |  | 9.05 | 18.68 | 28.47 | 25.47 | 13.38 | 4.12 | 0.74 | 0.08 | 0 | 0 |  | 100 | 0 |
| 42 | 6.68 | 14.92 | 25.55 | 26.83 | 17.28 | 6.82 | 1.65 | 0.24 | 0.02 | 0 |  | 9.05 | 18.68 | 28.46 | 25.47 | 13.38 | 4.12 | 0.74 | 0.08 | 0 | 0 |  | 100 | 0 |
| 43 | 6.68 | 14.92 | 25.55 | 26.83 | 17.28 | 6.82 | 1.65 | 0.24 | 0.02 | 0 |  | 9.05 | 18.68 | 28.46 | 25.47 | 13.38 | 4.12 | 0.74 | 0.08 | 0 | 0 |  | 100 | 0 |
| 44 | 6.68 | 14.92 | 25.55 | 26.83 | 17.28 | 6.82 | 1.65 | 0.24 | 0.02 | 0 |  | 9.05 | 18.68 | 28.46 | 25.47 | 13.38 | 4.12 | 0.74 | 0.08 | 0 | 0 |  | 100 | 0 |
| 45 | 6.68 | 14.92 | 25.55 | 26.83 | 17.28 | 6.82 | 1.65 | 0.24 | 0.02 | 0 |  | 9.05 | 18.68 | 28.46 | 25.47 | 13.39 | 4.13 | 0.75 | 0.08 | 0 | 0 |  | 100 | 0 |
| 46 | 6.68 | 14.92 | 25.55 | 26.83 | 17.28 | 6.82 | 1.65 | 0.24 | 0.02 | 0 |  | 9.05 | 18.68 | 28.46 | 25.47 | 13.39 | 4.13 | 0.75 | 0.08 | 0 | 0 |  | 100 | 0 |
| 47 | 6.68 | 14.92 | 25.55 | 26.83 | 17.28 | 6.82 | 1.65 | 0.24 | 0.02 | 0 |  | 9.05 | 18.68 | 28.46 | 25.47 | 13.39 | 4.13 | 0.75 | 0.08 | 0 | 0 |  | 100 | 0 |
| 48 | 6.68 | 14.92 | 25.55 | 26.83 | 17.28 | 6.82 | 1.65 | 0.24 | 0.02 | 0 |  | 9.05 | 18.68 | 28.46 | 25.47 | 13.39 | 4.13 | 0.75 | 0.08 | 0 | 0 |  | 100 | 0 |
| 49 | 6.68 | 14.92 | 25.55 | 26.83 | 17.28 | 6.82 | 1.65 | 0.24 | 0.02 | 0 |  | 9.05 | 18.68 | 28.46 | 25.47 | 13.39 | 4.13 | 0.75 | 0.08 | 0 | 0 |  | 100 | 0 |
| 50 | 6.68 | 14.92 | 25.55 | 26.83 | 17.28 | 6.82 | 1.65 | 0.24 | 0.02 | 0 |  | 8.92 | 18.49 | 28.34 | 25.57 | 13.58 | 4.24 | 0.78 | 0.08 | 0 | 0 |  | 100 | 0 |
| 51 | 6.68 | 14.92 | 25.55 | 26.83 | 17.28 | 6.82 | 1.65 | 0.24 | 0.02 | 0 |  | 8.92 | 18.49 | 28.34 | 25.57 | 13.58 | 4.24 | 0.78 | 0.08 | 0 | 0 |  | 100 | 0 |
| 52 | 6.68 | 14.92 | 25.55 | 26.83 | 17.28 | 6.82 | 1.65 | 0.24 | 0.02 | 0 |  | 8.92 | 18.49 | 28.34 | 25.57 | 13.58 | 4.24 | 0.78 | 0.08 | 0 | 0 |  | 100 | 0 |
| 53 | 6.67 | 14.92 | 25.55 | 26.84 | 17.29 | 6.82 | 1.65 | 0.24 | 0.02 | 0 |  | 8.91 | 18.48 | 28.35 | 25.58 | 13.58 | 4.24 | 0.78 | 0.08 | 0 | 0 |  | 100 | 0 |
| 54 | 6.66 | 14.91 | 25.56 | 26.85 | 17.29 | 6.82 | 1.65 | 0.24 | 0.02 | 0 |  | 8.89 | 18.48 | 28.36 | 25.59 | 13.58 | 4.24 | 0.78 | 0.08 | 0 | 0 |  | 100 | 0 |
| 55 | 6.64 | 14.9 | 25.56 | 26.87 | 17.3 | 6.82 | 1.64 | 0.24 | 0.02 | 0 |  | 8.87 | 18.46 | 28.36 | 25.61 | 13.6 | 4.24 | 0.78 | 0.08 | 0 | 0 |  | 100 | 0 |
| 56 | 6.62 | 14.89 | 25.57 | 26.88 | 17.31 | 6.82 | 1.64 | 0.24 | 0.02 | 0 |  | 8.85 | 18.45 | 28.37 | 25.63 | 13.6 | 4.24 | 0.77 | 0.08 | 0 | 0 |  | 100 | 0 |
| 57 | 6.61 | 14.88 | 25.57 | 26.9 | 17.32 | 6.82 | 1.64 | 0.24 | 0.02 | 0 |  | 8.83 | 18.44 | 28.38 | 25.64 | 13.61 | 4.23 | 0.77 | 0.08 | 0 | 0 |  | 100 | 0 |
| 58 | 6.6 | 14.87 | 25.57 | 26.91 | 17.32 | 6.82 | 1.64 | 0.24 | 0.02 | 0 |  | 8.82 | 18.44 | 28.38 | 25.65 | 13.61 | 4.23 | 0.77 | 0.08 | 0 | 0 |  | 100 | 0 |
| 59 | 6.62 | 14.88 | 25.57 | 26.89 | 17.31 | 6.82 | 1.64 | 0.24 | 0.02 | 0 |  | 8.84 | 18.45 | 28.38 | 25.64 | 13.6 | 4.24 | 0.77 | 0.08 | 0 | 0 |  | 100 | 0 |
| 60 | 6.67 | 14.91 | 25.55 | 26.84 | 17.29 | 6.82 | 1.65 | 0.24 | 0.02 | 0 |  | 8.84 | 18.38 | 28.28 | 25.64 | 13.68 | 4.3 | 0.79 | 0.09 | 0 | 0 |  | 100 | 0 |
| 61 | 6.78 | 14.98 | 25.52 | 26.73 | 17.23 | 6.82 | 1.66 | 0.25 | 0.02 | 0 |  | 8.97 | 18.44 | 28.22 | 25.53 | 13.65 | 4.31 | 0.8 | 0.09 | 0.01 | 0 |  | 100 | 0 |
| 62 | 6.98 | 15.1 | 25.46 | 26.55 | 17.12 | 6.83 | 1.68 | 0.26 | 0.02 | 0 |  | 9.2 | 18.53 | 28.1 | 25.34 | 13.59 | 4.33 | 0.82 | 0.09 | 0.01 | 0 |  | 100 | 0 |
| 63 | 7.27 | 15.26 | 25.36 | 26.28 | 16.98 | 6.83 | 1.71 | 0.27 | 0.03 | 0 |  | 9.55 | 18.66 | 27.93 | 25.07 | 13.49 | 4.35 | 0.84 | 0.1 | 0.01 | 0 |  | 100 | 0 |
| 64 | 7.66 | 15.47 | 25.24 | 25.94 | 16.79 | 6.84 | 1.75 | 0.28 | 0.03 | 0 |  | 10 | 18.82 | 27.71 | 24.73 | 13.38 | 4.38 | 0.87 | 0.1 | 0.01 | 0 |  | 100 | 0 |
| 65 | 8.1 | 15.68 | 25.1 | 25.57 | 16.58 | 6.84 | 1.8 | 0.3 | 0.03 | 0 |  | 10.4 | 18.85 | 27.38 | 24.43 | 13.39 | 4.5 | 0.93 | 0.12 | 0.01 | 0 |  | 100 | 0 |
| 66 | 8.49 | 15.87 | 24.96 | 25.24 | 16.4 | 6.85 | 1.84 | 0.32 | 0.03 | 0 |  | 10.85 | 18.99 | 27.16 | 24.11 | 13.27 | 4.53 | 0.96 | 0.12 | 0.01 | 0 |  | 100 | 0 |
| 67 | 8.8 | 16 | 24.86 | 25 | 16.27 | 6.85 | 1.86 | 0.33 | 0.04 | 0 |  | 11.2 | 19.09 | 26.99 | 23.86 | 13.18 | 4.55 | 0.98 | 0.13 | 0.01 | 0 |  | 100 | 0 |
| 68 | 9.01 | 16.09 | 24.79 | 24.83 | 16.18 | 6.85 | 1.88 | 0.34 | 0.04 | 0 |  | 11.44 | 19.15 | 26.88 | 23.7 | 13.12 | 4.56 | 0.99 | 0.14 | 0.01 | 0 |  | 100 | 0 |
| 69 | 9.13 | 16.13 | 24.75 | 24.74 | 16.12 | 6.85 | 1.89 | 0.34 | 0.04 | 0 |  | 11.58 | 19.18 | 26.82 | 23.61 | 13.09 | 4.57 | 1 | 0.14 | 0.01 | 0 |  | 100 | 0 |
| 70 | 9.19 | 16.16 | 24.73 | 24.7 | 16.1 | 6.85 | 1.9 | 0.34 | 0.04 | 0 |  | 11.57 | 19.12 | 26.74 | 23.61 | 13.16 | 4.62 | 1.03 | 0.14 | 0.01 | 0 |  | 100 | 0 |
| 71 | 9.21 | 16.16 | 24.72 | 24.69 | 16.09 | 6.85 | 1.9 | 0.34 | 0.04 | 0 |  | 11.59 | 19.12 | 26.73 | 23.6 | 13.15 | 4.63 | 1.03 | 0.14 | 0.01 | 0 |  | 100 | 0 |
| 72 | 9.2 | 16.16 | 24.72 | 24.69 | 16.1 | 6.85 | 1.9 | 0.34 | 0.04 | 0 |  | 11.58 | 19.12 | 26.73 | 23.6 | 13.15 | 4.63 | 1.03 | 0.14 | 0.01 | 0 |  | 100 | 0 |
| 73 | 9.18 | 16.15 | 24.73 | 24.7 | 16.1 | 6.85 | 1.9 | 0.34 | 0.04 | 0 |  | 11.56 | 19.11 | 26.74 | 23.62 | 13.16 | 4.62 | 1.02 | 0.14 | 0.01 | 0 |  | 100 | 0 |
| 74 | 9.16 | 16.14 | 24.74 | 24.72 | 16.11 | 6.85 | 1.9 | 0.34 | 0.04 | 0 |  | 11.54 | 19.11 | 26.75 | 23.63 | 13.16 | 4.62 | 1.02 | 0.14 | 0.01 | 0 |  | 100 | 0 |
| 75 | 9.14 | 16.14 | 24.74 | 24.73 | 16.12 | 6.85 | 1.89 | 0.34 | 0.04 | 0 |  | 11.45 | 19.02 | 26.72 | 23.69 | 13.25 | 4.68 | 1.04 | 0.15 | 0.01 | 0 |  | 100 | 0 |
| 76 | 9.13 | 16.13 | 24.75 | 24.74 | 16.12 | 6.85 | 1.89 | 0.34 | 0.04 | 0 |  | 11.44 | 19.02 | 26.72 | 23.69 | 13.26 | 4.68 | 1.04 | 0.14 | 0.01 | 0 |  | 100 | 0 |
| 77 | 9.12 | 16.13 | 24.75 | 24.75 | 16.13 | 6.85 | 1.89 | 0.34 | 0.04 | 0 |  | 11.43 | 19.02 | 26.73 | 23.7 | 13.26 | 4.68 | 1.04 | 0.14 | 0.01 | 0 |  | 100 | 0 |
| 78 | 9.12 | 16.13 | 24.75 | 24.75 | 16.13 | 6.85 | 1.89 | 0.34 | 0.04 | 0 |  | 11.42 | 19.01 | 26.73 | 23.7 | 13.26 | 4.68 | 1.04 | 0.14 | 0.01 | 0 |  | 100 | 0 |
| 79 | 9.12 | 16.13 | 24.75 | 24.75 | 16.13 | 6.85 | 1.89 | 0.34 | 0.04 | 0 |  | 11.42 | 19.01 | 26.73 | 23.71 | 13.26 | 4.68 | 1.04 | 0.14 | 0.01 | 0 |  | 100 | 0 |
| 80 | 9.12 | 16.13 | 24.75 | 24.75 | 16.13 | 6.85 | 1.89 | 0.34 | 0.04 | 0 |  | 11.35 | 18.93 | 26.68 | 23.75 | 13.34 | 4.73 | 1.06 | 0.15 | 0.01 | 0 |  | 100 | 0 |
| 81 | 9.12 | 16.13 | 24.75 | 24.75 | 16.13 | 6.85 | 1.89 | 0.34 | 0.04 | 0 |  | 11.35 | 18.93 | 26.68 | 23.75 | 13.34 | 4.73 | 1.06 | 0.15 | 0.01 | 0 |  | 100 | 0 |
| 82 | 9.12 | 16.13 | 24.75 | 24.75 | 16.13 | 6.85 | 1.89 | 0.34 | 0.04 | 0 |  | 11.35 | 18.93 | 26.68 | 23.74 | 13.34 | 4.73 | 1.06 | 0.15 | 0.01 | 0 |  | 100 | 0 |
| 83 | 9.12 | 16.13 | 24.75 | 24.75 | 16.13 | 6.85 | 1.89 | 0.34 | 0.04 | 0 |  | 11.35 | 18.93 | 26.68 | 23.74 | 13.34 | 4.73 | 1.06 | 0.15 | 0.01 | 0 |  | 100 | 0 |
| 84 | 9.12 | 16.13 | 24.75 | 24.75 | 16.13 | 6.85 | 1.89 | 0.34 | 0.04 | 0 |  | 11.35 | 18.93 | 26.68 | 23.74 | 13.34 | 4.73 | 1.06 | 0.15 | 0.01 | 0 |  | 100 | 0 |
| 85 | 9.12 | 16.13 | 24.75 | 24.75 | 16.13 | 6.85 | 1.89 | 0.34 | 0.04 | 0 |  | 11.22 | 18.78 | 26.59 | 23.82 | 13.49 | 4.83 | 1.09 | 0.16 | 0.01 | 0 |  | 100 | 0 |
| 86 | 9.12 | 16.13 | 24.75 | 24.75 | 16.13 | 6.85 | 1.89 | 0.34 | 0.04 | 0 |  | 11.22 | 18.78 | 26.59 | 23.82 | 13.49 | 4.83 | 1.09 | 0.16 | 0.01 | 0 |  | 100 | 0 |
| 87 | 9.12 | 16.13 | 24.75 | 24.75 | 16.13 | 6.85 | 1.89 | 0.34 | 0.04 | 0 |  | 11.22 | 18.78 | 26.59 | 23.82 | 13.49 | 4.83 | 1.09 | 0.16 | 0.01 | 0 |  | 100 | 0 |
| 88 | 9.12 | 16.13 | 24.75 | 24.75 | 16.13 | 6.85 | 1.89 | 0.34 | 0.04 | 0 |  | 11.22 | 18.78 | 26.59 | 23.82 | 13.49 | 4.83 | 1.09 | 0.16 | 0.01 | 0 |  | 100 | 0 |
| 89 | 9.12 | 16.13 | 24.75 | 24.75 | 16.13 | 6.85 | 1.89 | 0.34 | 0.04 | 0 |  | 11.22 | 18.78 | 26.59 | 23.82 | 13.49 | 4.83 | 1.09 | 0.16 | 0.01 | 0 |  | 100 | 0 |
| 90 | 9.12 | 16.13 | 24.75 | 24.75 | 16.13 | 6.85 | 1.89 | 0.34 | 0.04 | 0 |  | 11.22 | 18.78 | 26.59 | 23.82 | 13.49 | 4.83 | 1.09 | 0.16 | 0.01 | 0 |  | 100 | 0 |
| 91 | 9.12 | 16.13 | 24.75 | 24.75 | 16.13 | 6.85 | 1.89 | 0.34 | 0.04 | 0 |  | 11.22 | 18.78 | 26.59 | 23.82 | 13.49 | 4.83 | 1.09 | 0.16 | 0.01 | 0 |  | 100 | 0 |
| 92 | 9.12 | 16.13 | 24.75 | 24.75 | 16.13 | 6.85 | 1.89 | 0.34 | 0.04 | 0 |  | 11.22 | 18.78 | 26.59 | 23.82 | 13.49 | 4.83 | 1.09 | 0.16 | 0.01 | 0 |  | 100 | 0 |
| 93 | 9.12 | 16.13 | 24.75 | 24.75 | 16.13 | 6.85 | 1.89 | 0.34 | 0.04 | 0 |  | 11.22 | 18.78 | 26.59 | 23.82 | 13.49 | 4.83 | 1.09 | 0.16 | 0.01 | 0 |  | 100 | 0 |
| 94 | 9.12 | 16.13 | 24.75 | 24.75 | 16.13 | 6.85 | 1.89 | 0.34 | 0.04 | 0 |  | 11.22 | 18.78 | 26.59 | 23.82 | 13.49 | 4.83 | 1.09 | 0.16 | 0.01 | 0 |  | 100 | 0 |
| 95 | 9.12 | 16.13 | 24.75 | 24.75 | 16.13 | 6.85 | 1.89 | 0.34 | 0.04 | 0 |  | 11.22 | 18.78 | 26.59 | 23.82 | 13.49 | 4.83 | 1.09 | 0.16 | 0.01 | 0 |  | 100 | 0 |
|  | |  | Females | | | | | | | | | | | | | | | | | | | | | |
| 0 | 100 | 0 | 0 | 0 | 0 | 0 | 0 | 0 | 0 | 0 |  | 100 | 0 | 0 | 0 | 0 | 0 | 0 | 0 | 0 | 0 |  | 100 | 0 |
| 1 | 100 | 0 | 0 | 0 | 0 | 0 | 0 | 0 | 0 | 0 |  | 100 | 0 | 0 | 0 | 0 | 0 | 0 | 0 | 0 | 0 |  | 100 | 0 |
| 2 | 100 | 0 | 0 | 0 | 0 | 0 | 0 | 0 | 0 | 0 |  | 100 | 0 | 0 | 0 | 0 | 0 | 0 | 0 | 0 | 0 |  | 100 | 0 |
| 3 | 100 | 0 | 0 | 0 | 0 | 0 | 0 | 0 | 0 | 0 |  | 100 | 0 | 0 | 0 | 0 | 0 | 0 | 0 | 0 | 0 |  | 100 | 0 |
| 4 | 100 | 0 | 0 | 0 | 0 | 0 | 0 | 0 | 0 | 0 |  | 100 | 0 | 0 | 0 | 0 | 0 | 0 | 0 | 0 | 0 |  | 100 | 0 |
| 5 | 100 | 0 | 0 | 0 | 0 | 0 | 0 | 0 | 0 | 0 |  | 100 | 0 | 0 | 0 | 0 | 0 | 0 | 0 | 0 | 0 |  | 100 | 0 |
| 6 | 2.51 | 10.82 | 26.28 | 32.43 | 20.35 | 6.48 | 1.04 | 0.08 | 0 | 0 |  | 2.51 | 10.82 | 26.28 | 32.43 | 20.35 | 6.48 | 1.04 | 0.08 | 0 | 0 |  | 100 | 0 |
| 7 | 2.99 | 12.15 | 27.78 | 32.05 | 18.68 | 5.49 | 0.81 | 0.06 | 0 | 0 |  | 2.99 | 12.15 | 27.78 | 32.05 | 18.68 | 5.49 | 0.81 | 0.06 | 0 | 0 |  | 100 | 0 |
| 8 | 3.48 | 13.38 | 28.94 | 31.5 | 17.25 | 4.75 | 0.65 | 0.04 | 0 | 0 |  | 3.48 | 13.38 | 28.94 | 31.5 | 17.25 | 4.75 | 0.65 | 0.04 | 0 | 0 |  | 100 | 0 |
| 9 | 3.96 | 14.4 | 29.71 | 30.88 | 16.18 | 4.26 | 0.56 | 0.04 | 0 | 0 |  | 3.96 | 14.4 | 29.71 | 30.88 | 16.18 | 4.26 | 0.56 | 0.04 | 0 | 0 |  | 100 | 0 |
| 10 | 4.43 | 15.25 | 30.18 | 30.26 | 15.38 | 3.95 | 0.51 | 0.03 | 0 | 0 |  | 4.43 | 15.25 | 30.18 | 30.26 | 15.38 | 3.95 | 0.51 | 0.03 | 0 | 0 |  | 100 | 0 |
| 11 | 4.92 | 16.02 | 30.48 | 29.62 | 14.71 | 3.72 | 0.48 | 0.03 | 0 | 0 |  | 4.92 | 16.02 | 30.48 | 29.62 | 14.71 | 3.72 | 0.48 | 0.03 | 0 | 0 |  | 100 | 0 |
| 12 | 5.52 | 16.87 | 30.73 | 28.89 | 14.02 | 3.5 | 0.45 | 0.03 | 0 | 0 |  | 5.52 | 16.87 | 30.73 | 28.89 | 14.02 | 3.5 | 0.45 | 0.03 | 0 | 0 |  | 100 | 0 |
| 13 | 6.29 | 17.84 | 30.92 | 28 | 13.24 | 3.26 | 0.42 | 0.03 | 0 | 0 |  | 6.29 | 17.84 | 30.92 | 28 | 13.24 | 3.26 | 0.42 | 0.03 | 0 | 0 |  | 100 | 0 |
| 14 | 7.28 | 18.96 | 31.03 | 26.93 | 12.38 | 3.01 | 0.39 | 0.03 | 0 | 0 |  | 7.28 | 18.96 | 31.03 | 26.93 | 12.38 | 3.01 | 0.39 | 0.03 | 0 | 0 |  | 100 | 0 |
| 15 | 8.4 | 20 | 30.95 | 25.83 | 11.62 | 2.81 | 0.36 | 0.02 | 0 | 0 |  | 10.68 | 23.45 | 32.36 | 23.14 | 8.57 | 1.64 | 0.16 | 0.01 | 0 | 0 |  | 100 | 0 |
| 16 | 9.43 | 20.71 | 30.66 | 24.94 | 11.14 | 2.73 | 0.36 | 0.03 | 0 | 0 |  | 11.88 | 24.08 | 31.83 | 22.24 | 8.2 | 1.59 | 0.16 | 0.01 | 0 | 0 |  | 100 | 0 |
| 17 | 10.13 | 20.93 | 30.18 | 24.42 | 11.08 | 2.82 | 0.4 | 0.03 | 0 | 0 |  | 12.68 | 24.18 | 31.25 | 21.81 | 8.22 | 1.67 | 0.18 | 0.01 | 0 | 0 |  | 100 | 0 |
| 18 | 10.48 | 20.68 | 29.56 | 24.26 | 11.42 | 3.08 | 0.48 | 0.04 | 0 | 0 |  | 13.01 | 23.81 | 30.64 | 21.82 | 8.6 | 1.87 | 0.22 | 0.02 | 0 | 0 |  | 100 | 0 |
| 19 | 10.55 | 20.18 | 28.9 | 24.29 | 11.98 | 3.46 | 0.59 | 0.06 | 0 | 0 |  | 13.15 | 23.33 | 30.08 | 21.96 | 9.06 | 2.11 | 0.28 | 0.02 | 0 | 0 |  | 100 | 0 |
| 20 | 10.46 | 19.62 | 28.29 | 24.39 | 12.57 | 3.87 | 0.71 | 0.08 | 0 | 0 |  | 12.99 | 22.66 | 29.57 | 22.28 | 9.69 | 2.43 | 0.35 | 0.03 | 0 | 0 |  | 100 | 0 |
| 21 | 10.33 | 19.15 | 27.82 | 24.5 | 13.06 | 4.22 | 0.82 | 0.1 | 0.01 | 0 |  | 12.78 | 22.12 | 29.18 | 22.56 | 10.21 | 2.7 | 0.42 | 0.04 | 0 | 0 |  | 100 | 0 |
| 22 | 10.19 | 18.8 | 27.5 | 24.58 | 13.43 | 4.48 | 0.91 | 0.11 | 0.01 | 0 |  | 12.58 | 21.71 | 28.92 | 22.77 | 10.59 | 2.91 | 0.47 | 0.04 | 0 | 0 |  | 100 | 0 |
| 23 | 10.08 | 18.57 | 27.3 | 24.65 | 13.66 | 4.64 | 0.97 | 0.12 | 0.01 | 0 |  | 12.43 | 21.45 | 28.76 | 22.91 | 10.84 | 3.04 | 0.51 | 0.05 | 0 | 0 |  | 100 | 0 |
| 24 | 10 | 18.44 | 27.2 | 24.7 | 13.79 | 4.74 | 1 | 0.13 | 0.01 | 0 |  | 12.33 | 21.31 | 28.69 | 23 | 10.98 | 3.12 | 0.52 | 0.05 | 0 | 0 |  | 100 | 0 |
| 25 | 9.95 | 18.38 | 27.17 | 24.73 | 13.85 | 4.77 | 1.01 | 0.13 | 0.01 | 0 |  | 12.34 | 21.34 | 28.7 | 22.98 | 10.95 | 3.1 | 0.52 | 0.05 | 0 | 0 |  | 100 | 0 |
| 26 | 9.92 | 18.36 | 27.18 | 24.75 | 13.87 | 4.78 | 1.01 | 0.13 | 0.01 | 0 |  | 12.31 | 21.32 | 28.72 | 23.01 | 10.96 | 3.1 | 0.52 | 0.05 | 0 | 0 |  | 100 | 0 |
| 27 | 9.9 | 18.37 | 27.2 | 24.76 | 13.85 | 4.76 | 1 | 0.13 | 0.01 | 0 |  | 12.3 | 21.34 | 28.74 | 23.01 | 10.95 | 3.09 | 0.52 | 0.05 | 0 | 0 |  | 100 | 0 |
| 28 | 9.9 | 18.39 | 27.23 | 24.76 | 13.83 | 4.74 | 1 | 0.13 | 0.01 | 0 |  | 12.3 | 21.36 | 28.77 | 23 | 10.92 | 3.08 | 0.51 | 0.05 | 0 | 0 |  | 100 | 0 |
| 29 | 9.91 | 18.41 | 27.26 | 24.76 | 13.81 | 4.72 | 0.99 | 0.13 | 0.01 | 0 |  | 12.31 | 21.39 | 28.79 | 23 | 10.9 | 3.06 | 0.51 | 0.05 | 0 | 0 |  | 100 | 0 |
| 30 | 9.91 | 18.43 | 27.27 | 24.76 | 13.79 | 4.71 | 0.98 | 0.13 | 0.01 | 0 |  | 12.47 | 21.59 | 28.87 | 22.86 | 10.71 | 2.97 | 0.48 | 0.05 | 0 | 0 |  | 100 | 0 |
| 31 | 9.92 | 18.45 | 27.29 | 24.76 | 13.78 | 4.7 | 0.98 | 0.12 | 0.01 | 0 |  | 12.48 | 21.6 | 28.88 | 22.85 | 10.69 | 2.96 | 0.48 | 0.05 | 0 | 0 |  | 100 | 0 |
| 32 | 9.92 | 18.45 | 27.3 | 24.76 | 13.77 | 4.69 | 0.98 | 0.12 | 0.01 | 0 |  | 12.48 | 21.61 | 28.89 | 22.84 | 10.68 | 2.95 | 0.48 | 0.05 | 0 | 0 |  | 100 | 0 |
| 33 | 9.93 | 18.46 | 27.3 | 24.75 | 13.76 | 4.69 | 0.98 | 0.12 | 0.01 | 0 |  | 12.49 | 21.62 | 28.89 | 22.84 | 10.68 | 2.95 | 0.48 | 0.05 | 0 | 0 |  | 100 | 0 |
| 34 | 9.93 | 18.46 | 27.3 | 24.75 | 13.76 | 4.69 | 0.98 | 0.12 | 0.01 | 0 |  | 12.49 | 21.62 | 28.89 | 22.84 | 10.68 | 2.95 | 0.48 | 0.05 | 0 | 0 |  | 100 | 0 |
| 35 | 9.93 | 18.46 | 27.3 | 24.75 | 13.76 | 4.69 | 0.98 | 0.12 | 0.01 | 0 |  | 12.49 | 21.62 | 28.89 | 22.84 | 10.68 | 2.95 | 0.48 | 0.05 | 0 | 0 |  | 100 | 0 |
| 36 | 9.93 | 18.46 | 27.3 | 24.75 | 13.76 | 4.69 | 0.98 | 0.12 | 0.01 | 0 |  | 12.49 | 21.62 | 28.89 | 22.84 | 10.68 | 2.95 | 0.48 | 0.05 | 0 | 0 |  | 100 | 0 |
| 37 | 9.93 | 18.46 | 27.3 | 24.75 | 13.76 | 4.69 | 0.98 | 0.12 | 0.01 | 0 |  | 12.49 | 21.62 | 28.89 | 22.84 | 10.68 | 2.95 | 0.48 | 0.05 | 0 | 0 |  | 100 | 0 |
| 38 | 9.93 | 18.46 | 27.3 | 24.75 | 13.76 | 4.69 | 0.98 | 0.12 | 0.01 | 0 |  | 12.49 | 21.62 | 28.89 | 22.84 | 10.68 | 2.95 | 0.48 | 0.05 | 0 | 0 |  | 100 | 0 |
| 39 | 9.93 | 18.46 | 27.3 | 24.75 | 13.76 | 4.69 | 0.98 | 0.12 | 0.01 | 0 |  | 12.49 | 21.62 | 28.89 | 22.84 | 10.68 | 2.95 | 0.48 | 0.05 | 0 | 0 |  | 100 | 0 |
| 40 | 9.93 | 18.46 | 27.3 | 24.75 | 13.76 | 4.69 | 0.98 | 0.12 | 0.01 | 0 |  | 12.65 | 21.8 | 28.95 | 22.71 | 10.51 | 2.87 | 0.46 | 0.04 | 0 | 0 |  | 100 | 0 |
| 41 | 9.93 | 18.46 | 27.29 | 24.75 | 13.76 | 4.69 | 0.98 | 0.12 | 0.01 | 0 |  | 12.65 | 21.8 | 28.95 | 22.71 | 10.51 | 2.87 | 0.46 | 0.04 | 0 | 0 |  | 100 | 0 |
| 42 | 9.93 | 18.46 | 27.29 | 24.75 | 13.76 | 4.69 | 0.98 | 0.12 | 0.01 | 0 |  | 12.65 | 21.8 | 28.95 | 22.71 | 10.51 | 2.87 | 0.46 | 0.04 | 0 | 0 |  | 100 | 0 |
| 43 | 9.93 | 18.46 | 27.29 | 24.75 | 13.76 | 4.69 | 0.98 | 0.12 | 0.01 | 0 |  | 12.65 | 21.8 | 28.95 | 22.71 | 10.51 | 2.87 | 0.46 | 0.04 | 0 | 0 |  | 100 | 0 |
| 44 | 9.93 | 18.46 | 27.29 | 24.75 | 13.76 | 4.69 | 0.98 | 0.12 | 0.01 | 0 |  | 12.65 | 21.8 | 28.95 | 22.71 | 10.51 | 2.87 | 0.46 | 0.04 | 0 | 0 |  | 100 | 0 |
| 45 | 9.93 | 18.46 | 27.29 | 24.75 | 13.76 | 4.69 | 0.98 | 0.12 | 0.01 | 0 |  | 12.65 | 21.8 | 28.95 | 22.71 | 10.51 | 2.87 | 0.46 | 0.04 | 0 | 0 |  | 100 | 0 |
| 46 | 9.93 | 18.46 | 27.29 | 24.75 | 13.77 | 4.69 | 0.98 | 0.12 | 0.01 | 0 |  | 12.65 | 21.79 | 28.95 | 22.71 | 10.52 | 2.87 | 0.46 | 0.04 | 0 | 0 |  | 100 | 0 |
| 47 | 9.93 | 18.46 | 27.29 | 24.75 | 13.77 | 4.69 | 0.98 | 0.12 | 0.01 | 0 |  | 12.65 | 21.79 | 28.95 | 22.71 | 10.52 | 2.87 | 0.46 | 0.04 | 0 | 0 |  | 100 | 0 |
| 48 | 9.93 | 18.45 | 27.29 | 24.75 | 13.77 | 4.69 | 0.98 | 0.12 | 0.01 | 0 |  | 12.65 | 21.79 | 28.95 | 22.71 | 10.52 | 2.87 | 0.46 | 0.04 | 0 | 0 |  | 100 | 0 |
| 49 | 9.93 | 18.45 | 27.29 | 24.75 | 13.77 | 4.69 | 0.98 | 0.12 | 0.01 | 0 |  | 12.65 | 21.79 | 28.95 | 22.71 | 10.52 | 2.87 | 0.46 | 0.04 | 0 | 0 |  | 100 | 0 |
| 50 | 9.93 | 18.45 | 27.29 | 24.75 | 13.77 | 4.69 | 0.98 | 0.12 | 0.01 | 0 |  | 12.72 | 21.88 | 28.98 | 22.65 | 10.44 | 2.83 | 0.45 | 0.04 | 0 | 0 |  | 100 | 0 |
| 51 | 9.93 | 18.46 | 27.29 | 24.75 | 13.77 | 4.69 | 0.98 | 0.12 | 0.01 | 0 |  | 12.73 | 21.88 | 28.98 | 22.65 | 10.43 | 2.83 | 0.45 | 0.04 | 0 | 0 |  | 100 | 0 |
| 52 | 9.93 | 18.46 | 27.3 | 24.75 | 13.76 | 4.69 | 0.98 | 0.12 | 0.01 | 0 |  | 12.73 | 21.89 | 28.99 | 22.64 | 10.43 | 2.83 | 0.45 | 0.04 | 0 | 0 |  | 100 | 0 |
| 53 | 9.93 | 18.47 | 27.31 | 24.75 | 13.75 | 4.68 | 0.98 | 0.12 | 0.01 | 0 |  | 12.73 | 21.9 | 28.99 | 22.64 | 10.42 | 2.82 | 0.45 | 0.04 | 0 | 0 |  | 100 | 0 |
| 54 | 9.94 | 18.48 | 27.32 | 24.75 | 13.74 | 4.67 | 0.97 | 0.12 | 0.01 | 0 |  | 12.74 | 21.91 | 29 | 22.63 | 10.4 | 2.82 | 0.45 | 0.04 | 0 | 0 |  | 100 | 0 |
| 55 | 9.94 | 18.5 | 27.34 | 24.75 | 13.72 | 4.66 | 0.97 | 0.12 | 0.01 | 0 |  | 12.83 | 22.02 | 29.05 | 22.55 | 10.3 | 2.76 | 0.43 | 0.04 | 0 | 0 |  | 100 | 0 |
| 56 | 9.95 | 18.52 | 27.36 | 24.74 | 13.7 | 4.64 | 0.96 | 0.12 | 0.01 | 0 |  | 12.84 | 22.05 | 29.07 | 22.54 | 10.28 | 2.75 | 0.43 | 0.04 | 0 | 0 |  | 100 | 0 |
| 57 | 9.96 | 18.54 | 27.37 | 24.74 | 13.68 | 4.63 | 0.96 | 0.12 | 0.01 | 0 |  | 12.85 | 22.07 | 29.08 | 22.53 | 10.26 | 2.74 | 0.43 | 0.04 | 0 | 0 |  | 100 | 0 |
| 58 | 9.96 | 18.55 | 27.38 | 24.74 | 13.67 | 4.62 | 0.95 | 0.12 | 0.01 | 0 |  | 12.86 | 22.08 | 29.09 | 22.52 | 10.25 | 2.74 | 0.43 | 0.04 | 0 | 0 |  | 100 | 0 |
| 59 | 9.95 | 18.53 | 27.37 | 24.74 | 13.69 | 4.63 | 0.96 | 0.12 | 0.01 | 0 |  | 12.85 | 22.06 | 29.07 | 22.53 | 10.27 | 2.75 | 0.43 | 0.04 | 0 | 0 |  | 100 | 0 |
| 60 | 9.93 | 18.47 | 27.31 | 24.75 | 13.75 | 4.68 | 0.98 | 0.12 | 0.01 | 0 |  | 12.89 | 22.08 | 29.06 | 22.5 | 10.25 | 2.74 | 0.43 | 0.04 | 0 | 0 |  | 100 | 0 |
| 61 | 9.89 | 18.35 | 27.18 | 24.77 | 13.88 | 4.78 | 1.01 | 0.13 | 0.01 | 0 |  | 12.83 | 21.93 | 28.96 | 22.58 | 10.39 | 2.82 | 0.45 | 0.04 | 0 | 0 |  | 100 | 0 |
| 62 | 9.81 | 18.14 | 26.97 | 24.8 | 14.1 | 4.95 | 1.07 | 0.14 | 0.01 | 0 |  | 12.71 | 21.68 | 28.79 | 22.7 | 10.63 | 2.95 | 0.49 | 0.05 | 0 | 0 |  | 100 | 0 |
| 63 | 9.7 | 17.83 | 26.65 | 24.83 | 14.42 | 5.21 | 1.17 | 0.16 | 0.01 | 0 |  | 12.54 | 21.31 | 28.52 | 22.88 | 10.99 | 3.16 | 0.54 | 0.06 | 0 | 0 |  | 100 | 0 |
| 64 | 9.57 | 17.45 | 26.24 | 24.85 | 14.81 | 5.55 | 1.31 | 0.19 | 0.02 | 0 |  | 12.33 | 20.85 | 28.18 | 23.07 | 11.44 | 3.43 | 0.62 | 0.07 | 0 | 0 |  | 100 | 0 |
| 65 | 9.43 | 17.03 | 25.79 | 24.85 | 15.24 | 5.94 | 1.47 | 0.23 | 0.02 | 0 |  | 12.2 | 20.45 | 27.83 | 23.2 | 11.84 | 3.69 | 0.7 | 0.08 | 0.01 | 0 |  | 100 | 0 |
| 66 | 9.3 | 16.67 | 25.38 | 24.82 | 15.6 | 6.29 | 1.63 | 0.27 | 0.03 | 0 |  | 12 | 20.01 | 27.48 | 23.35 | 12.27 | 3.99 | 0.8 | 0.1 | 0.01 | 0 |  | 100 | 0 |
| 67 | 9.21 | 16.4 | 25.07 | 24.79 | 15.87 | 6.57 | 1.76 | 0.3 | 0.03 | 0 |  | 11.85 | 19.68 | 27.2 | 23.45 | 12.6 | 4.22 | 0.88 | 0.11 | 0.01 | 0 |  | 100 | 0 |
| 68 | 9.15 | 16.22 | 24.86 | 24.77 | 16.04 | 6.75 | 1.85 | 0.33 | 0.04 | 0 |  | 11.76 | 19.46 | 27.01 | 23.5 | 12.82 | 4.38 | 0.94 | 0.12 | 0.01 | 0 |  | 100 | 0 |
| 69 | 9.12 | 16.12 | 24.74 | 24.75 | 16.14 | 6.86 | 1.9 | 0.34 | 0.04 | 0 |  | 11.7 | 19.34 | 26.9 | 23.53 | 12.94 | 4.47 | 0.97 | 0.13 | 0.01 | 0 |  | 100 | 0 |
| 70 | 9.1 | 16.07 | 24.68 | 24.74 | 16.18 | 6.91 | 1.92 | 0.35 | 0.04 | 0 |  | 11.61 | 19.19 | 26.81 | 23.59 | 13.08 | 4.57 | 1 | 0.14 | 0.01 | 0 |  | 100 | 0 |
| 71 | 9.1 | 16.06 | 24.67 | 24.74 | 16.2 | 6.92 | 1.93 | 0.35 | 0.04 | 0 |  | 11.6 | 19.18 | 26.79 | 23.59 | 13.1 | 4.58 | 1.01 | 0.14 | 0.01 | 0 |  | 100 | 0 |
| 72 | 9.1 | 16.06 | 24.67 | 24.74 | 16.19 | 6.92 | 1.93 | 0.35 | 0.04 | 0 |  | 11.6 | 19.19 | 26.8 | 23.59 | 13.09 | 4.57 | 1.01 | 0.14 | 0.01 | 0 |  | 100 | 0 |
| 73 | 9.1 | 16.08 | 24.69 | 24.74 | 16.18 | 6.9 | 1.92 | 0.35 | 0.04 | 0 |  | 11.61 | 19.2 | 26.82 | 23.59 | 13.07 | 4.56 | 1 | 0.14 | 0.01 | 0 |  | 100 | 0 |
| 74 | 9.11 | 16.09 | 24.71 | 24.74 | 16.16 | 6.88 | 1.91 | 0.34 | 0.04 | 0 |  | 11.62 | 19.22 | 26.83 | 23.58 | 13.05 | 4.54 | 1 | 0.14 | 0.01 | 0 |  | 100 | 0 |
| 75 | 9.11 | 16.11 | 24.73 | 24.75 | 16.15 | 6.87 | 1.9 | 0.34 | 0.04 | 0 |  | 11.62 | 19.24 | 26.85 | 23.58 | 13.04 | 4.53 | 0.99 | 0.14 | 0.01 | 0 |  | 100 | 0 |
| 76 | 9.12 | 16.12 | 24.74 | 24.75 | 16.14 | 6.86 | 1.9 | 0.34 | 0.04 | 0 |  | 11.63 | 19.25 | 26.86 | 23.58 | 13.02 | 4.52 | 0.99 | 0.14 | 0.01 | 0 |  | 100 | 0 |
| 77 | 9.12 | 16.12 | 24.75 | 24.75 | 16.13 | 6.85 | 1.89 | 0.34 | 0.04 | 0 |  | 11.63 | 19.26 | 26.86 | 23.58 | 13.02 | 4.52 | 0.98 | 0.14 | 0.01 | 0 |  | 100 | 0 |
| 78 | 9.12 | 16.13 | 24.75 | 24.75 | 16.13 | 6.84 | 1.89 | 0.34 | 0.04 | 0 |  | 11.64 | 19.26 | 26.87 | 23.58 | 13.01 | 4.51 | 0.98 | 0.14 | 0.01 | 0 |  | 100 | 0 |
| 79 | 9.12 | 16.13 | 24.75 | 24.75 | 16.12 | 6.84 | 1.89 | 0.34 | 0.04 | 0 |  | 11.64 | 19.27 | 26.87 | 23.57 | 13.01 | 4.51 | 0.98 | 0.13 | 0.01 | 0 |  | 100 | 0 |
| 80 | 9.12 | 16.13 | 24.75 | 24.75 | 16.12 | 6.84 | 1.89 | 0.34 | 0.04 | 0 |  | 11.49 | 19.1 | 26.78 | 23.66 | 13.17 | 4.62 | 1.02 | 0.14 | 0.01 | 0 |  | 100 | 0 |
| 81 | 9.12 | 16.13 | 24.75 | 24.75 | 16.13 | 6.84 | 1.89 | 0.34 | 0.04 | 0 |  | 11.49 | 19.1 | 26.78 | 23.66 | 13.17 | 4.62 | 1.02 | 0.14 | 0.01 | 0 |  | 100 | 0 |
| 82 | 9.12 | 16.13 | 24.75 | 24.75 | 16.13 | 6.84 | 1.89 | 0.34 | 0.04 | 0 |  | 11.49 | 19.1 | 26.78 | 23.66 | 13.17 | 4.62 | 1.02 | 0.14 | 0.01 | 0 |  | 100 | 0 |
| 83 | 9.12 | 16.13 | 24.75 | 24.75 | 16.13 | 6.84 | 1.89 | 0.34 | 0.04 | 0 |  | 11.49 | 19.1 | 26.78 | 23.66 | 13.18 | 4.62 | 1.02 | 0.14 | 0.01 | 0 |  | 100 | 0 |
| 84 | 9.12 | 16.13 | 24.75 | 24.75 | 16.13 | 6.84 | 1.89 | 0.34 | 0.04 | 0 |  | 11.49 | 19.1 | 26.78 | 23.66 | 13.18 | 4.62 | 1.02 | 0.14 | 0.01 | 0 |  | 100 | 0 |
| 85 | 9.12 | 16.13 | 24.75 | 24.75 | 16.13 | 6.85 | 1.89 | 0.34 | 0.04 | 0 |  | 11.42 | 19.02 | 26.73 | 23.7 | 13.26 | 4.67 | 1.04 | 0.14 | 0.01 | 0 |  | 100 | 0 |
| 86 | 9.12 | 16.13 | 24.75 | 24.75 | 16.13 | 6.85 | 1.89 | 0.34 | 0.04 | 0 |  | 11.42 | 19.01 | 26.73 | 23.7 | 13.26 | 4.67 | 1.04 | 0.14 | 0.01 | 0 |  | 100 | 0 |
| 87 | 9.12 | 16.13 | 24.75 | 24.75 | 16.13 | 6.85 | 1.89 | 0.34 | 0.04 | 0 |  | 11.42 | 19.01 | 26.73 | 23.7 | 13.26 | 4.68 | 1.04 | 0.14 | 0.01 | 0 |  | 100 | 0 |
| 88 | 9.12 | 16.13 | 24.75 | 24.75 | 16.13 | 6.85 | 1.89 | 0.34 | 0.04 | 0 |  | 11.42 | 19.01 | 26.73 | 23.7 | 13.26 | 4.68 | 1.04 | 0.14 | 0.01 | 0 |  | 100 | 0 |
| 89 | 9.12 | 16.13 | 24.75 | 24.75 | 16.13 | 6.85 | 1.89 | 0.34 | 0.04 | 0 |  | 11.42 | 19.01 | 26.73 | 23.7 | 13.26 | 4.68 | 1.04 | 0.14 | 0.01 | 0 |  | 100 | 0 |
| 90 | 9.12 | 16.13 | 24.75 | 24.75 | 16.13 | 6.85 | 1.89 | 0.34 | 0.04 | 0 |  | 11.42 | 19.01 | 26.73 | 23.7 | 13.26 | 4.68 | 1.04 | 0.14 | 0.01 | 0 |  | 100 | 0 |
| 91 | 9.12 | 16.13 | 24.75 | 24.75 | 16.13 | 6.85 | 1.89 | 0.34 | 0.04 | 0 |  | 11.42 | 19.01 | 26.73 | 23.7 | 13.26 | 4.68 | 1.04 | 0.14 | 0.01 | 0 |  | 100 | 0 |
| 92 | 9.12 | 16.13 | 24.75 | 24.75 | 16.13 | 6.85 | 1.89 | 0.34 | 0.04 | 0 |  | 11.42 | 19.01 | 26.73 | 23.7 | 13.26 | 4.68 | 1.04 | 0.14 | 0.01 | 0 |  | 100 | 0 |
| 93 | 9.12 | 16.13 | 24.75 | 24.75 | 16.13 | 6.85 | 1.89 | 0.34 | 0.04 | 0 |  | 11.42 | 19.01 | 26.73 | 23.7 | 13.26 | 4.67 | 1.04 | 0.14 | 0.01 | 0 |  | 100 | 0 |
| 94 | 9.12 | 16.13 | 24.75 | 24.75 | 16.13 | 6.85 | 1.89 | 0.34 | 0.04 | 0 |  | 11.42 | 19.01 | 26.73 | 23.7 | 13.26 | 4.67 | 1.04 | 0.14 | 0.01 | 0 |  | 100 | 0 |
| 95 | 9.12 | 16.13 | 24.75 | 24.75 | 16.13 | 6.85 | 1.89 | 0.34 | 0.04 | 0 |  | 11.42 | 19.01 | 26.73 | 23.7 | 13.26 | 4.67 | 1.04 | 0.14 | 0.01 | 0 |  | 100 | 0 |

^a^ Rounded to two decimal points

^b^ %E = Percent of total energy intake
