# Supplementary material for: Substituting polyunsaturated fat for saturated fat: A health impact assessment of a fat tax in seven European countries
Source: PLoS One. 2019 Jul 10;14(7):e0218464. doi: 10.1371/journal.pone.0218464 (PMC6619676; doi:10.1371/journal.pone.0218464)
Supplement: S11 Table — (DOCX) [file pone.0218464.s011.docx]

# S11 Table. Proportion of persons in the respective saturated fat intake categories across sceanrios in Italy.

| Age | Reference scenario^a^ | | | | | | | | | |  | Fat tax scenario^a^ | | | | | | | | | |  | Guideline scenario | |
| --- | --- | --- | --- | --- | --- | --- | --- | --- | --- | --- | --- | --- | --- | --- | --- | --- | --- | --- | --- | --- | --- | --- | --- | --- |
|  | Category of saturated fat intake (in %E)^b^ | | | | | | | | | |  | Category of saturated fat intake (in %E)^b^ | | | | | | | | | |  | Category of saturated fat intake (in %E)^b^ | |
|  | ≤10 | >10 ≤12 | >12 ≤14 | >14 ≤16 | >16 ≤18 | >18 ≤20 | >20 ≤22 | >22 ≤24 | >24 ≤26 | >26 ≤100 |  | ≤10 | >10 ≤12 | >12 ≤14 | >14 ≤16 | >16 ≤18 | >18 ≤20 | >20 ≤22 | >22 ≤24 | >24 ≤26 | >26 ≤100 |  | ≤10 | >10 ≤100 |
|  |  |  | Males | | | | | | | | | | | | | | | | | | | | | |
| 0 | 100 | 0 | 0 | 0 | 0 | 0 | 0 | 0 | 0 | 0 |  | 100 | 0 | 0 | 0 | 0 | 0 | 0 | 0 | 0 | 0 |  | 100 | 0 |
| 1 | 100 | 0 | 0 | 0 | 0 | 0 | 0 | 0 | 0 | 0 |  | 100 | 0 | 0 | 0 | 0 | 0 | 0 | 0 | 0 | 0 |  | 100 | 0 |
| 2 | 100 | 0 | 0 | 0 | 0 | 0 | 0 | 0 | 0 | 0 |  | 100 | 0 | 0 | 0 | 0 | 0 | 0 | 0 | 0 | 0 |  | 100 | 0 |
| 3 | 100 | 0 | 0 | 0 | 0 | 0 | 0 | 0 | 0 | 0 |  | 100 | 0 | 0 | 0 | 0 | 0 | 0 | 0 | 0 | 0 |  | 100 | 0 |
| 4 | 100 | 0 | 0 | 0 | 0 | 0 | 0 | 0 | 0 | 0 |  | 100 | 0 | 0 | 0 | 0 | 0 | 0 | 0 | 0 | 0 |  | 100 | 0 |
| 5 | 100 | 0 | 0 | 0 | 0 | 0 | 0 | 0 | 0 | 0 |  | 100 | 0 | 0 | 0 | 0 | 0 | 0 | 0 | 0 | 0 |  | 100 | 0 |
| 6 | 44.61 | 31.28 | 17.95 | 5.3 | 0.8 | 0.06 | 0 | 0 | 0 | 0 |  | 44.61 | 31.28 | 17.95 | 5.3 | 0.8 | 0.06 | 0 | 0 | 0 | 0 |  | 100 | 0 |
| 7 | 47.39 | 31.06 | 16.5 | 4.41 | 0.59 | 0.04 | 0 | 0 | 0 | 0 |  | 47.39 | 31.06 | 16.5 | 4.41 | 0.59 | 0.04 | 0 | 0 | 0 | 0 |  | 100 | 0 |
| 8 | 48.58 | 30.99 | 15.86 | 4.03 | 0.51 | 0.03 | 0 | 0 | 0 | 0 |  | 48.58 | 30.99 | 15.86 | 4.03 | 0.51 | 0.03 | 0 | 0 | 0 | 0 |  | 100 | 0 |
| 9 | 47.65 | 31.29 | 16.29 | 4.2 | 0.53 | 0.03 | 0 | 0 | 0 | 0 |  | 47.65 | 31.29 | 16.29 | 4.2 | 0.53 | 0.03 | 0 | 0 | 0 | 0 |  | 100 | 0 |
| 10 | 45.21 | 31.77 | 17.5 | 4.81 | 0.66 | 0.04 | 0 | 0 | 0 | 0 |  | 45.21 | 31.77 | 17.5 | 4.81 | 0.66 | 0.04 | 0 | 0 | 0 | 0 |  | 100 | 0 |
| 11 | 42.17 | 32.17 | 19.05 | 5.69 | 0.86 | 0.06 | 0 | 0 | 0 | 0 |  | 42.17 | 32.17 | 19.05 | 5.69 | 0.86 | 0.06 | 0 | 0 | 0 | 0 |  | 100 | 0 |
| 12 | 39.28 | 32.38 | 20.52 | 6.63 | 1.09 | 0.09 | 0 | 0 | 0 | 0 |  | 39.28 | 32.38 | 20.52 | 6.63 | 1.09 | 0.09 | 0 | 0 | 0 | 0 |  | 100 | 0 |
| 13 | 36.83 | 32.43 | 21.77 | 7.51 | 1.33 | 0.12 | 0.01 | 0 | 0 | 0 |  | 36.83 | 32.43 | 21.77 | 7.51 | 1.33 | 0.12 | 0.01 | 0 | 0 | 0 |  | 100 | 0 |
| 14 | 34.92 | 32.39 | 22.74 | 8.25 | 1.54 | 0.15 | 0.01 | 0 | 0 | 0 |  | 34.92 | 32.39 | 22.74 | 8.25 | 1.54 | 0.15 | 0.01 | 0 | 0 | 0 |  | 100 | 0 |
| 15 | 33.65 | 32.32 | 23.38 | 8.77 | 1.7 | 0.17 | 0.01 | 0 | 0 | 0 |  | 43.94 | 32.94 | 17.88 | 4.63 | 0.57 | 0.03 | 0 | 0 | 0 | 0 |  | 100 | 0 |
| 16 | 33.12 | 32.27 | 23.65 | 9 | 1.78 | 0.18 | 0.01 | 0 | 0 | 0 |  | 43.32 | 33.04 | 18.21 | 4.8 | 0.6 | 0.04 | 0 | 0 | 0 | 0 |  | 100 | 0 |
| 17 | 33.41 | 32.25 | 23.5 | 8.9 | 1.75 | 0.18 | 0.01 | 0 | 0 | 0 |  | 43.65 | 32.95 | 18.05 | 4.73 | 0.59 | 0.03 | 0 | 0 | 0 | 0 |  | 100 | 0 |
| 18 | 34.44 | 32.24 | 22.98 | 8.52 | 1.64 | 0.16 | 0.01 | 0 | 0 | 0 |  | 44.82 | 32.68 | 17.46 | 4.46 | 0.54 | 0.03 | 0 | 0 | 0 | 0 |  | 100 | 0 |
| 19 | 35.88 | 32.21 | 22.27 | 8.01 | 1.49 | 0.14 | 0.01 | 0 | 0 | 0 |  | 46.62 | 32.26 | 16.56 | 4.06 | 0.47 | 0.03 | 0 | 0 | 0 | 0 |  | 100 | 0 |
| 20 | 37.35 | 32.13 | 21.54 | 7.5 | 1.35 | 0.13 | 0.01 | 0 | 0 | 0 |  | 48.04 | 31.85 | 15.88 | 3.78 | 0.43 | 0.02 | 0 | 0 | 0 | 0 |  | 100 | 0 |
| 21 | 38.56 | 32.04 | 20.93 | 7.1 | 1.25 | 0.11 | 0 | 0 | 0 | 0 |  | 49.41 | 31.46 | 15.22 | 3.51 | 0.38 | 0.02 | 0 | 0 | 0 | 0 |  | 100 | 0 |
| 22 | 39.45 | 31.96 | 20.49 | 6.82 | 1.17 | 0.1 | 0 | 0 | 0 | 0 |  | 50.4 | 31.15 | 14.75 | 3.33 | 0.36 | 0.02 | 0 | 0 | 0 | 0 |  | 100 | 0 |
| 23 | 40.01 | 31.9 | 20.21 | 6.64 | 1.13 | 0.1 | 0 | 0 | 0 | 0 |  | 51.01 | 30.96 | 14.46 | 3.21 | 0.34 | 0.02 | 0 | 0 | 0 | 0 |  | 100 | 0 |
| 24 | 40.3 | 31.87 | 20.07 | 6.56 | 1.11 | 0.1 | 0 | 0 | 0 | 0 |  | 51.34 | 30.86 | 14.31 | 3.16 | 0.33 | 0.02 | 0 | 0 | 0 | 0 |  | 100 | 0 |
| 25 | 40.41 | 31.85 | 20.01 | 6.52 | 1.1 | 0.1 | 0 | 0 | 0 | 0 |  | 51.19 | 30.87 | 14.39 | 3.2 | 0.34 | 0.02 | 0 | 0 | 0 | 0 |  | 100 | 0 |
| 26 | 40.42 | 31.85 | 20.01 | 6.52 | 1.1 | 0.1 | 0 | 0 | 0 | 0 |  | 51.19 | 30.87 | 14.39 | 3.19 | 0.34 | 0.02 | 0 | 0 | 0 | 0 |  | 100 | 0 |
| 27 | 40.37 | 31.86 | 20.04 | 6.53 | 1.1 | 0.1 | 0 | 0 | 0 | 0 |  | 51.14 | 30.89 | 14.41 | 3.2 | 0.34 | 0.02 | 0 | 0 | 0 | 0 |  | 100 | 0 |
| 28 | 40.3 | 31.87 | 20.07 | 6.55 | 1.11 | 0.1 | 0 | 0 | 0 | 0 |  | 51.06 | 30.91 | 14.45 | 3.22 | 0.34 | 0.02 | 0 | 0 | 0 | 0 |  | 100 | 0 |
| 29 | 40.24 | 31.87 | 20.1 | 6.57 | 1.11 | 0.1 | 0 | 0 | 0 | 0 |  | 50.99 | 30.94 | 14.48 | 3.23 | 0.34 | 0.02 | 0 | 0 | 0 | 0 |  | 100 | 0 |
| 30 | 40.19 | 31.88 | 20.13 | 6.59 | 1.12 | 0.1 | 0 | 0 | 0 | 0 |  | 50.66 | 31.01 | 14.65 | 3.31 | 0.35 | 0.02 | 0 | 0 | 0 | 0 |  | 100 | 0 |
| 31 | 40.15 | 31.89 | 20.14 | 6.6 | 1.12 | 0.1 | 0 | 0 | 0 | 0 |  | 50.62 | 31.02 | 14.67 | 3.31 | 0.36 | 0.02 | 0 | 0 | 0 | 0 |  | 100 | 0 |
| 32 | 40.13 | 31.89 | 20.15 | 6.61 | 1.12 | 0.1 | 0 | 0 | 0 | 0 |  | 50.6 | 31.03 | 14.68 | 3.32 | 0.36 | 0.02 | 0 | 0 | 0 | 0 |  | 100 | 0 |
| 33 | 40.12 | 31.89 | 20.16 | 6.61 | 1.12 | 0.1 | 0 | 0 | 0 | 0 |  | 50.59 | 31.03 | 14.68 | 3.32 | 0.36 | 0.02 | 0 | 0 | 0 | 0 |  | 100 | 0 |
| 34 | 40.12 | 31.89 | 20.16 | 6.61 | 1.12 | 0.1 | 0 | 0 | 0 | 0 |  | 50.59 | 31.04 | 14.69 | 3.32 | 0.36 | 0.02 | 0 | 0 | 0 | 0 |  | 100 | 0 |
| 35 | 40.12 | 31.89 | 20.16 | 6.61 | 1.12 | 0.1 | 0 | 0 | 0 | 0 |  | 50.56 | 31.04 | 14.7 | 3.32 | 0.36 | 0.02 | 0 | 0 | 0 | 0 |  | 100 | 0 |
| 36 | 40.12 | 31.89 | 20.16 | 6.61 | 1.12 | 0.1 | 0 | 0 | 0 | 0 |  | 50.57 | 31.04 | 14.7 | 3.32 | 0.36 | 0.02 | 0 | 0 | 0 | 0 |  | 100 | 0 |
| 37 | 40.12 | 31.89 | 20.16 | 6.61 | 1.12 | 0.1 | 0 | 0 | 0 | 0 |  | 50.57 | 31.04 | 14.69 | 3.32 | 0.36 | 0.02 | 0 | 0 | 0 | 0 |  | 100 | 0 |
| 38 | 40.12 | 31.89 | 20.16 | 6.61 | 1.12 | 0.1 | 0 | 0 | 0 | 0 |  | 50.57 | 31.04 | 14.69 | 3.32 | 0.36 | 0.02 | 0 | 0 | 0 | 0 |  | 100 | 0 |
| 39 | 40.13 | 31.89 | 20.16 | 6.61 | 1.12 | 0.1 | 0 | 0 | 0 | 0 |  | 50.58 | 31.04 | 14.69 | 3.32 | 0.36 | 0.02 | 0 | 0 | 0 | 0 |  | 100 | 0 |
| 40 | 40.13 | 31.89 | 20.16 | 6.61 | 1.12 | 0.1 | 0 | 0 | 0 | 0 |  | 50.32 | 31.09 | 14.82 | 3.38 | 0.37 | 0.02 | 0 | 0 | 0 | 0 |  | 100 | 0 |
| 41 | 40.13 | 31.89 | 20.16 | 6.61 | 1.12 | 0.1 | 0 | 0 | 0 | 0 |  | 50.32 | 31.09 | 14.82 | 3.38 | 0.37 | 0.02 | 0 | 0 | 0 | 0 |  | 100 | 0 |
| 42 | 40.13 | 31.89 | 20.15 | 6.61 | 1.12 | 0.1 | 0 | 0 | 0 | 0 |  | 50.32 | 31.09 | 14.82 | 3.38 | 0.37 | 0.02 | 0 | 0 | 0 | 0 |  | 100 | 0 |
| 43 | 40.13 | 31.89 | 20.15 | 6.61 | 1.12 | 0.1 | 0 | 0 | 0 | 0 |  | 50.32 | 31.09 | 14.82 | 3.38 | 0.37 | 0.02 | 0 | 0 | 0 | 0 |  | 100 | 0 |
| 44 | 40.13 | 31.89 | 20.15 | 6.61 | 1.12 | 0.1 | 0 | 0 | 0 | 0 |  | 50.32 | 31.09 | 14.82 | 3.38 | 0.37 | 0.02 | 0 | 0 | 0 | 0 |  | 100 | 0 |
| 45 | 40.13 | 31.88 | 20.15 | 6.61 | 1.12 | 0.1 | 0 | 0 | 0 | 0 |  | 50.3 | 31.09 | 14.83 | 3.39 | 0.37 | 0.02 | 0 | 0 | 0 | 0 |  | 100 | 0 |
| 46 | 40.14 | 31.88 | 20.15 | 6.61 | 1.12 | 0.1 | 0 | 0 | 0 | 0 |  | 50.3 | 31.08 | 14.83 | 3.39 | 0.37 | 0.02 | 0 | 0 | 0 | 0 |  | 100 | 0 |
| 47 | 40.14 | 31.88 | 20.15 | 6.61 | 1.12 | 0.1 | 0 | 0 | 0 | 0 |  | 50.31 | 31.08 | 14.83 | 3.39 | 0.37 | 0.02 | 0 | 0 | 0 | 0 |  | 100 | 0 |
| 48 | 40.14 | 31.88 | 20.15 | 6.61 | 1.12 | 0.1 | 0 | 0 | 0 | 0 |  | 50.31 | 31.08 | 14.83 | 3.39 | 0.37 | 0.02 | 0 | 0 | 0 | 0 |  | 100 | 0 |
| 49 | 40.15 | 31.87 | 20.15 | 6.61 | 1.12 | 0.1 | 0 | 0 | 0 | 0 |  | 50.31 | 31.08 | 14.83 | 3.39 | 0.37 | 0.02 | 0 | 0 | 0 | 0 |  | 100 | 0 |
| 50 | 40.14 | 31.87 | 20.15 | 6.61 | 1.12 | 0.1 | 0 | 0 | 0 | 0 |  | 49.81 | 31.17 | 15.09 | 3.52 | 0.39 | 0.02 | 0 | 0 | 0 | 0 |  | 100 | 0 |
| 51 | 40.14 | 31.88 | 20.15 | 6.61 | 1.12 | 0.1 | 0 | 0 | 0 | 0 |  | 49.8 | 31.18 | 15.1 | 3.51 | 0.39 | 0.02 | 0 | 0 | 0 | 0 |  | 100 | 0 |
| 52 | 40.11 | 31.9 | 20.16 | 6.61 | 1.12 | 0.1 | 0 | 0 | 0 | 0 |  | 49.78 | 31.2 | 15.1 | 3.51 | 0.39 | 0.02 | 0 | 0 | 0 | 0 |  | 100 | 0 |
| 53 | 40.07 | 31.93 | 20.18 | 6.6 | 1.12 | 0.1 | 0 | 0 | 0 | 0 |  | 49.74 | 31.23 | 15.11 | 3.51 | 0.39 | 0.02 | 0 | 0 | 0 | 0 |  | 100 | 0 |
| 54 | 40.01 | 31.97 | 20.2 | 6.6 | 1.11 | 0.1 | 0 | 0 | 0 | 0 |  | 49.69 | 31.28 | 15.12 | 3.5 | 0.39 | 0.02 | 0 | 0 | 0 | 0 |  | 100 | 0 |
| 55 | 39.93 | 32.04 | 20.24 | 6.59 | 1.1 | 0.1 | 0 | 0 | 0 | 0 |  | 49.6 | 31.35 | 15.15 | 3.5 | 0.38 | 0.02 | 0 | 0 | 0 | 0 |  | 100 | 0 |
| 56 | 39.84 | 32.1 | 20.28 | 6.59 | 1.1 | 0.09 | 0 | 0 | 0 | 0 |  | 49.52 | 31.42 | 15.17 | 3.49 | 0.38 | 0.02 | 0 | 0 | 0 | 0 |  | 100 | 0 |
| 57 | 39.75 | 32.17 | 20.31 | 6.58 | 1.09 | 0.09 | 0 | 0 | 0 | 0 |  | 49.44 | 31.49 | 15.19 | 3.48 | 0.38 | 0.02 | 0 | 0 | 0 | 0 |  | 100 | 0 |
| 58 | 39.71 | 32.2 | 20.33 | 6.58 | 1.09 | 0.09 | 0 | 0 | 0 | 0 |  | 49.41 | 31.52 | 15.2 | 3.48 | 0.38 | 0.02 | 0 | 0 | 0 | 0 |  | 100 | 0 |
| 59 | 39.79 | 32.14 | 20.3 | 6.58 | 1.09 | 0.09 | 0 | 0 | 0 | 0 |  | 49.48 | 31.46 | 15.18 | 3.48 | 0.38 | 0.02 | 0 | 0 | 0 | 0 |  | 100 | 0 |
| 60 | 40.07 | 31.93 | 20.18 | 6.6 | 1.12 | 0.1 | 0 | 0 | 0 | 0 |  | 49.46 | 31.28 | 15.25 | 3.58 | 0.4 | 0.02 | 0 | 0 | 0 | 0 |  | 100 | 0 |
| 61 | 40.65 | 31.5 | 19.94 | 6.64 | 1.16 | 0.11 | 0 | 0 | 0 | 0 |  | 49.97 | 30.82 | 15.13 | 3.63 | 0.43 | 0.02 | 0 | 0 | 0 | 0 |  | 100 | 0 |
| 62 | 41.63 | 30.78 | 19.54 | 6.69 | 1.23 | 0.12 | 0.01 | 0 | 0 | 0 |  | 50.82 | 30.05 | 14.91 | 3.72 | 0.47 | 0.03 | 0 | 0 | 0 | 0 |  | 100 | 0 |
| 63 | 43.03 | 29.75 | 18.96 | 6.76 | 1.34 | 0.15 | 0.01 | 0 | 0 | 0 |  | 52.02 | 28.97 | 14.59 | 3.84 | 0.53 | 0.04 | 0 | 0 | 0 | 0 |  | 100 | 0 |
| 64 | 44.7 | 28.52 | 18.28 | 6.82 | 1.48 | 0.19 | 0.01 | 0 | 0 | 0 |  | 53.47 | 27.69 | 14.2 | 3.98 | 0.61 | 0.05 | 0 | 0 | 0 | 0 |  | 100 | 0 |
| 65 | 46.45 | 27.26 | 17.57 | 6.86 | 1.62 | 0.23 | 0.02 | 0 | 0 | 0 |  | 54.62 | 26.45 | 13.93 | 4.2 | 0.72 | 0.07 | 0 | 0 | 0 | 0 |  | 100 | 0 |
| 66 | 47.9 | 26.22 | 16.98 | 6.87 | 1.73 | 0.27 | 0.03 | 0 | 0 | 0 |  | 55.88 | 25.39 | 13.55 | 4.28 | 0.8 | 0.09 | 0.01 | 0 | 0 | 0 |  | 100 | 0 |
| 67 | 48.97 | 25.47 | 16.54 | 6.86 | 1.82 | 0.31 | 0.03 | 0 | 0 | 0 |  | 56.8 | 24.62 | 13.27 | 4.34 | 0.86 | 0.1 | 0.01 | 0 | 0 | 0 |  | 100 | 0 |
| 68 | 49.65 | 24.99 | 16.27 | 6.85 | 1.87 | 0.33 | 0.04 | 0 | 0 | 0 |  | 57.39 | 24.13 | 13.09 | 4.37 | 0.9 | 0.11 | 0.01 | 0 | 0 | 0 |  | 100 | 0 |
| 69 | 50.04 | 24.72 | 16.11 | 6.84 | 1.9 | 0.34 | 0.04 | 0 | 0 | 0 |  | 57.73 | 23.86 | 12.98 | 4.38 | 0.92 | 0.12 | 0.01 | 0 | 0 | 0 |  | 100 | 0 |
| 70 | 50.22 | 24.6 | 16.04 | 6.84 | 1.91 | 0.35 | 0.04 | 0 | 0 | 0 |  | 57.68 | 23.77 | 13.02 | 4.45 | 0.95 | 0.12 | 0.01 | 0 | 0 | 0 |  | 100 | 0 |
| 71 | 50.27 | 24.57 | 16.02 | 6.84 | 1.91 | 0.35 | 0.04 | 0 | 0 | 0 |  | 57.72 | 23.74 | 13.01 | 4.45 | 0.95 | 0.13 | 0.01 | 0 | 0 | 0 |  | 100 | 0 |
| 72 | 50.24 | 24.58 | 16.03 | 6.84 | 1.91 | 0.35 | 0.04 | 0 | 0 | 0 |  | 57.7 | 23.75 | 13.01 | 4.45 | 0.95 | 0.13 | 0.01 | 0 | 0 | 0 |  | 100 | 0 |
| 73 | 50.19 | 24.62 | 16.05 | 6.84 | 1.91 | 0.35 | 0.04 | 0 | 0 | 0 |  | 57.65 | 23.79 | 13.03 | 4.45 | 0.94 | 0.12 | 0.01 | 0 | 0 | 0 |  | 100 | 0 |
| 74 | 50.13 | 24.66 | 16.08 | 6.84 | 1.9 | 0.34 | 0.04 | 0 | 0 | 0 |  | 57.6 | 23.84 | 13.05 | 4.45 | 0.94 | 0.12 | 0.01 | 0 | 0 | 0 |  | 100 | 0 |
| 75 | 50.08 | 24.7 | 16.1 | 6.84 | 1.9 | 0.34 | 0.04 | 0 | 0 | 0 |  | 57.34 | 23.91 | 13.15 | 4.5 | 0.96 | 0.13 | 0.01 | 0 | 0 | 0 |  | 100 | 0 |
| 76 | 50.04 | 24.73 | 16.11 | 6.84 | 1.9 | 0.34 | 0.04 | 0 | 0 | 0 |  | 57.31 | 23.94 | 13.16 | 4.5 | 0.96 | 0.13 | 0.01 | 0 | 0 | 0 |  | 100 | 0 |
| 77 | 50.01 | 24.74 | 16.12 | 6.85 | 1.89 | 0.34 | 0.04 | 0 | 0 | 0 |  | 57.29 | 23.96 | 13.17 | 4.5 | 0.96 | 0.13 | 0.01 | 0 | 0 | 0 |  | 100 | 0 |
| 78 | 50 | 24.75 | 16.13 | 6.85 | 1.89 | 0.34 | 0.04 | 0 | 0 | 0 |  | 57.27 | 23.97 | 13.17 | 4.5 | 0.95 | 0.13 | 0.01 | 0 | 0 | 0 |  | 100 | 0 |
| 79 | 49.99 | 24.76 | 16.13 | 6.85 | 1.89 | 0.34 | 0.04 | 0 | 0 | 0 |  | 57.27 | 23.97 | 13.17 | 4.5 | 0.95 | 0.12 | 0.01 | 0 | 0 | 0 |  | 100 | 0 |
| 80 | 49.99 | 24.76 | 16.13 | 6.85 | 1.89 | 0.34 | 0.04 | 0 | 0 | 0 |  | 57.07 | 24.01 | 13.26 | 4.56 | 0.97 | 0.13 | 0.01 | 0 | 0 | 0 |  | 100 | 0 |
| 81 | 49.99 | 24.76 | 16.13 | 6.85 | 1.89 | 0.34 | 0.04 | 0 | 0 | 0 |  | 57.07 | 24.01 | 13.26 | 4.56 | 0.97 | 0.13 | 0.01 | 0 | 0 | 0 |  | 100 | 0 |
| 82 | 49.99 | 24.76 | 16.13 | 6.85 | 1.89 | 0.34 | 0.04 | 0 | 0 | 0 |  | 57.07 | 24 | 13.26 | 4.56 | 0.97 | 0.13 | 0.01 | 0 | 0 | 0 |  | 100 | 0 |
| 83 | 49.99 | 24.75 | 16.13 | 6.85 | 1.89 | 0.34 | 0.04 | 0 | 0 | 0 |  | 57.07 | 24 | 13.26 | 4.56 | 0.97 | 0.13 | 0.01 | 0 | 0 | 0 |  | 100 | 0 |
| 84 | 50 | 24.75 | 16.13 | 6.85 | 1.89 | 0.34 | 0.04 | 0 | 0 | 0 |  | 57.07 | 24 | 13.26 | 4.56 | 0.97 | 0.13 | 0.01 | 0 | 0 | 0 |  | 100 | 0 |
| 85 | 50 | 24.75 | 16.13 | 6.85 | 1.89 | 0.34 | 0.04 | 0 | 0 | 0 |  | 56.68 | 24.07 | 13.42 | 4.67 | 1.01 | 0.14 | 0.01 | 0 | 0 | 0 |  | 100 | 0 |
| 86 | 50 | 24.75 | 16.13 | 6.85 | 1.89 | 0.34 | 0.04 | 0 | 0 | 0 |  | 56.69 | 24.06 | 13.42 | 4.67 | 1.01 | 0.14 | 0.01 | 0 | 0 | 0 |  | 100 | 0 |
| 87 | 50 | 24.75 | 16.13 | 6.85 | 1.89 | 0.34 | 0.04 | 0 | 0 | 0 |  | 56.69 | 24.06 | 13.42 | 4.67 | 1.01 | 0.14 | 0.01 | 0 | 0 | 0 |  | 100 | 0 |
| 88 | 50 | 24.75 | 16.13 | 6.85 | 1.89 | 0.34 | 0.04 | 0 | 0 | 0 |  | 56.69 | 24.06 | 13.42 | 4.67 | 1.01 | 0.14 | 0.01 | 0 | 0 | 0 |  | 100 | 0 |
| 89 | 50 | 24.75 | 16.13 | 6.85 | 1.89 | 0.34 | 0.04 | 0 | 0 | 0 |  | 56.69 | 24.06 | 13.42 | 4.67 | 1.01 | 0.14 | 0.01 | 0 | 0 | 0 |  | 100 | 0 |
| 90 | 50 | 24.75 | 16.13 | 6.85 | 1.89 | 0.34 | 0.04 | 0 | 0 | 0 |  | 56.69 | 24.06 | 13.42 | 4.67 | 1.01 | 0.14 | 0.01 | 0 | 0 | 0 |  | 100 | 0 |
| 91 | 50 | 24.75 | 16.13 | 6.85 | 1.89 | 0.34 | 0.04 | 0 | 0 | 0 |  | 56.69 | 24.06 | 13.42 | 4.67 | 1.01 | 0.14 | 0.01 | 0 | 0 | 0 |  | 100 | 0 |
| 92 | 50 | 24.75 | 16.13 | 6.85 | 1.89 | 0.34 | 0.04 | 0 | 0 | 0 |  | 56.69 | 24.06 | 13.42 | 4.67 | 1.01 | 0.14 | 0.01 | 0 | 0 | 0 |  | 100 | 0 |
| 93 | 50 | 24.75 | 16.13 | 6.85 | 1.89 | 0.34 | 0.04 | 0 | 0 | 0 |  | 56.69 | 24.06 | 13.42 | 4.67 | 1.01 | 0.14 | 0.01 | 0 | 0 | 0 |  | 100 | 0 |
| 94 | 50 | 24.75 | 16.13 | 6.85 | 1.89 | 0.34 | 0.04 | 0 | 0 | 0 |  | 56.69 | 24.06 | 13.42 | 4.67 | 1.01 | 0.14 | 0.01 | 0 | 0 | 0 |  | 100 | 0 |
| 95 | 50 | 24.75 | 16.13 | 6.85 | 1.89 | 0.34 | 0.04 | 0 | 0 | 0 |  | 56.69 | 24.06 | 13.42 | 4.67 | 1.01 | 0.14 | 0.01 | 0 | 0 | 0 |  | 100 | 0 |
|  | |  | Females | | | | | | | | | | | | | | | | | | | | | |
| 0 | 100 | 0 | 0 | 0 | 0 | 0 | 0 | 0 | 0 | 0 |  | 100 | 0 | 0 | 0 | 0 | 0 | 0 | 0 | 0 | 0 |  | 100 | 0 |
| 1 | 100 | 0 | 0 | 0 | 0 | 0 | 0 | 0 | 0 | 0 |  | 100 | 0 | 0 | 0 | 0 | 0 | 0 | 0 | 0 | 0 |  | 100 | 0 |
| 2 | 100 | 0 | 0 | 0 | 0 | 0 | 0 | 0 | 0 | 0 |  | 100 | 0 | 0 | 0 | 0 | 0 | 0 | 0 | 0 | 0 |  | 100 | 0 |
| 3 | 100 | 0 | 0 | 0 | 0 | 0 | 0 | 0 | 0 | 0 |  | 100 | 0 | 0 | 0 | 0 | 0 | 0 | 0 | 0 | 0 |  | 100 | 0 |
| 4 | 100 | 0 | 0 | 0 | 0 | 0 | 0 | 0 | 0 | 0 |  | 100 | 0 | 0 | 0 | 0 | 0 | 0 | 0 | 0 | 0 |  | 100 | 0 |
| 5 | 100 | 0 | 0 | 0 | 0 | 0 | 0 | 0 | 0 | 0 |  | 100 | 0 | 0 | 0 | 0 | 0 | 0 | 0 | 0 | 0 |  | 100 | 0 |
| 6 | 35.24 | 35.58 | 22.17 | 6.2 | 0.77 | 0.04 | 0 | 0 | 0 | 0 |  | 35.24 | 35.58 | 22.17 | 6.2 | 0.77 | 0.04 | 0 | 0 | 0 | 0 |  | 100 | 0 |
| 7 | 36.74 | 34.4 | 21.56 | 6.36 | 0.88 | 0.06 | 0 | 0 | 0 | 0 |  | 36.74 | 34.4 | 21.56 | 6.36 | 0.88 | 0.06 | 0 | 0 | 0 | 0 |  | 100 | 0 |
| 8 | 38.52 | 33.47 | 20.75 | 6.27 | 0.92 | 0.06 | 0 | 0 | 0 | 0 |  | 38.52 | 33.47 | 20.75 | 6.27 | 0.92 | 0.06 | 0 | 0 | 0 | 0 |  | 100 | 0 |
| 9 | 40.65 | 32.79 | 19.74 | 5.89 | 0.87 | 0.06 | 0 | 0 | 0 | 0 |  | 40.65 | 32.79 | 19.74 | 5.89 | 0.87 | 0.06 | 0 | 0 | 0 | 0 |  | 100 | 0 |
| 10 | 42.87 | 32.27 | 18.65 | 5.38 | 0.77 | 0.06 | 0 | 0 | 0 | 0 |  | 42.87 | 32.27 | 18.65 | 5.38 | 0.77 | 0.06 | 0 | 0 | 0 | 0 |  | 100 | 0 |
| 11 | 44.84 | 31.87 | 17.68 | 4.89 | 0.67 | 0.05 | 0 | 0 | 0 | 0 |  | 44.84 | 31.87 | 17.68 | 4.89 | 0.67 | 0.05 | 0 | 0 | 0 | 0 |  | 100 | 0 |
| 12 | 46.29 | 31.61 | 16.95 | 4.52 | 0.6 | 0.04 | 0 | 0 | 0 | 0 |  | 46.29 | 31.61 | 16.95 | 4.52 | 0.6 | 0.04 | 0 | 0 | 0 | 0 |  | 100 | 0 |
| 13 | 47.19 | 31.45 | 16.5 | 4.28 | 0.55 | 0.03 | 0 | 0 | 0 | 0 |  | 47.19 | 31.45 | 16.5 | 4.28 | 0.55 | 0.03 | 0 | 0 | 0 | 0 |  | 100 | 0 |
| 14 | 47.59 | 31.37 | 16.3 | 4.18 | 0.53 | 0.03 | 0 | 0 | 0 | 0 |  | 47.59 | 31.37 | 16.3 | 4.18 | 0.53 | 0.03 | 0 | 0 | 0 | 0 |  | 100 | 0 |
| 15 | 47.69 | 31.3 | 16.27 | 4.18 | 0.53 | 0.03 | 0 | 0 | 0 | 0 |  | 55.39 | 29.65 | 12.34 | 2.39 | 0.21 | 0.01 | 0 | 0 | 0 | 0 |  | 100 | 0 |
| 16 | 47.65 | 31.17 | 16.32 | 4.26 | 0.55 | 0.03 | 0 | 0 | 0 | 0 |  | 55.31 | 29.57 | 12.43 | 2.45 | 0.22 | 0.01 | 0 | 0 | 0 | 0 |  | 100 | 0 |
| 17 | 47.64 | 30.94 | 16.4 | 4.39 | 0.59 | 0.04 | 0 | 0 | 0 | 0 |  | 55.23 | 29.4 | 12.56 | 2.55 | 0.24 | 0.01 | 0 | 0 | 0 | 0 |  | 100 | 0 |
| 18 | 47.72 | 30.59 | 16.46 | 4.54 | 0.64 | 0.05 | 0 | 0 | 0 | 0 |  | 55.21 | 29.13 | 12.7 | 2.68 | 0.27 | 0.01 | 0 | 0 | 0 | 0 |  | 100 | 0 |
| 19 | 47.87 | 30.19 | 16.48 | 4.7 | 0.7 | 0.05 | 0 | 0 | 0 | 0 |  | 55.61 | 28.71 | 12.64 | 2.74 | 0.29 | 0.02 | 0 | 0 | 0 | 0 |  | 100 | 0 |
| 20 | 48.04 | 29.82 | 16.48 | 4.84 | 0.75 | 0.06 | 0 | 0 | 0 | 0 |  | 55.68 | 28.4 | 12.72 | 2.86 | 0.32 | 0.02 | 0 | 0 | 0 | 0 |  | 100 | 0 |
| 21 | 48.19 | 29.54 | 16.47 | 4.94 | 0.79 | 0.07 | 0 | 0 | 0 | 0 |  | 55.76 | 28.17 | 12.77 | 2.94 | 0.34 | 0.02 | 0 | 0 | 0 | 0 |  | 100 | 0 |
| 22 | 48.3 | 29.35 | 16.46 | 5 | 0.82 | 0.07 | 0 | 0 | 0 | 0 |  | 55.82 | 28 | 12.8 | 3 | 0.36 | 0.02 | 0 | 0 | 0 | 0 |  | 100 | 0 |
| 23 | 48.37 | 29.23 | 16.44 | 5.04 | 0.84 | 0.08 | 0 | 0 | 0 | 0 |  | 55.87 | 27.9 | 12.81 | 3.03 | 0.37 | 0.02 | 0 | 0 | 0 | 0 |  | 100 | 0 |
| 24 | 48.41 | 29.18 | 16.43 | 5.05 | 0.84 | 0.08 | 0 | 0 | 0 | 0 |  | 55.9 | 27.86 | 12.81 | 3.04 | 0.37 | 0.02 | 0 | 0 | 0 | 0 |  | 100 | 0 |
| 25 | 48.43 | 29.16 | 16.43 | 5.05 | 0.85 | 0.08 | 0 | 0 | 0 | 0 |  | 56.14 | 27.78 | 12.7 | 2.99 | 0.36 | 0.02 | 0 | 0 | 0 | 0 |  | 100 | 0 |
| 26 | 48.44 | 29.17 | 16.42 | 5.05 | 0.85 | 0.08 | 0 | 0 | 0 | 0 |  | 56.15 | 27.78 | 12.7 | 2.99 | 0.36 | 0.02 | 0 | 0 | 0 | 0 |  | 100 | 0 |
| 27 | 48.43 | 29.18 | 16.42 | 5.04 | 0.84 | 0.08 | 0 | 0 | 0 | 0 |  | 56.15 | 27.79 | 12.69 | 2.99 | 0.36 | 0.02 | 0 | 0 | 0 | 0 |  | 100 | 0 |
| 28 | 48.43 | 29.2 | 16.42 | 5.04 | 0.84 | 0.08 | 0 | 0 | 0 | 0 |  | 56.15 | 27.81 | 12.69 | 2.98 | 0.36 | 0.02 | 0 | 0 | 0 | 0 |  | 100 | 0 |
| 29 | 48.42 | 29.21 | 16.42 | 5.03 | 0.84 | 0.08 | 0 | 0 | 0 | 0 |  | 56.14 | 27.82 | 12.69 | 2.97 | 0.36 | 0.02 | 0 | 0 | 0 | 0 |  | 100 | 0 |
| 30 | 48.41 | 29.22 | 16.42 | 5.03 | 0.84 | 0.08 | 0 | 0 | 0 | 0 |  | 56.6 | 27.71 | 12.46 | 2.87 | 0.34 | 0.02 | 0 | 0 | 0 | 0 |  | 100 | 0 |
| 31 | 48.41 | 29.23 | 16.42 | 5.03 | 0.84 | 0.08 | 0 | 0 | 0 | 0 |  | 56.6 | 27.71 | 12.46 | 2.87 | 0.34 | 0.02 | 0 | 0 | 0 | 0 |  | 100 | 0 |
| 32 | 48.41 | 29.23 | 16.42 | 5.02 | 0.84 | 0.08 | 0 | 0 | 0 | 0 |  | 56.6 | 27.71 | 12.46 | 2.87 | 0.34 | 0.02 | 0 | 0 | 0 | 0 |  | 100 | 0 |
| 33 | 48.4 | 29.23 | 16.42 | 5.02 | 0.83 | 0.08 | 0 | 0 | 0 | 0 |  | 56.6 | 27.72 | 12.46 | 2.87 | 0.34 | 0.02 | 0 | 0 | 0 | 0 |  | 100 | 0 |
| 34 | 48.4 | 29.24 | 16.43 | 5.02 | 0.83 | 0.08 | 0 | 0 | 0 | 0 |  | 56.6 | 27.72 | 12.46 | 2.87 | 0.34 | 0.02 | 0 | 0 | 0 | 0 |  | 100 | 0 |
| 35 | 48.4 | 29.23 | 16.43 | 5.02 | 0.83 | 0.08 | 0 | 0 | 0 | 0 |  | 56.6 | 27.72 | 12.46 | 2.87 | 0.34 | 0.02 | 0 | 0 | 0 | 0 |  | 100 | 0 |
| 36 | 48.4 | 29.23 | 16.43 | 5.02 | 0.84 | 0.08 | 0 | 0 | 0 | 0 |  | 56.6 | 27.72 | 12.46 | 2.87 | 0.34 | 0.02 | 0 | 0 | 0 | 0 |  | 100 | 0 |
| 37 | 48.4 | 29.23 | 16.43 | 5.02 | 0.84 | 0.08 | 0 | 0 | 0 | 0 |  | 56.6 | 27.72 | 12.46 | 2.87 | 0.34 | 0.02 | 0 | 0 | 0 | 0 |  | 100 | 0 |
| 38 | 48.4 | 29.23 | 16.43 | 5.02 | 0.84 | 0.08 | 0 | 0 | 0 | 0 |  | 56.6 | 27.71 | 12.46 | 2.87 | 0.34 | 0.02 | 0 | 0 | 0 | 0 |  | 100 | 0 |
| 39 | 48.4 | 29.23 | 16.43 | 5.02 | 0.84 | 0.08 | 0 | 0 | 0 | 0 |  | 56.6 | 27.71 | 12.46 | 2.87 | 0.34 | 0.02 | 0 | 0 | 0 | 0 |  | 100 | 0 |
| 40 | 48.4 | 29.23 | 16.43 | 5.02 | 0.84 | 0.08 | 0 | 0 | 0 | 0 |  | 57.06 | 27.59 | 12.24 | 2.77 | 0.32 | 0.02 | 0 | 0 | 0 | 0 |  | 100 | 0 |
| 41 | 48.4 | 29.23 | 16.43 | 5.02 | 0.84 | 0.08 | 0 | 0 | 0 | 0 |  | 57.06 | 27.59 | 12.24 | 2.77 | 0.32 | 0.02 | 0 | 0 | 0 | 0 |  | 100 | 0 |
| 42 | 48.4 | 29.23 | 16.43 | 5.02 | 0.84 | 0.08 | 0 | 0 | 0 | 0 |  | 57.06 | 27.59 | 12.24 | 2.77 | 0.32 | 0.02 | 0 | 0 | 0 | 0 |  | 100 | 0 |
| 43 | 48.41 | 29.23 | 16.43 | 5.02 | 0.84 | 0.08 | 0 | 0 | 0 | 0 |  | 57.06 | 27.59 | 12.24 | 2.77 | 0.32 | 0.02 | 0 | 0 | 0 | 0 |  | 100 | 0 |
| 44 | 48.41 | 29.23 | 16.42 | 5.02 | 0.84 | 0.08 | 0 | 0 | 0 | 0 |  | 57.07 | 27.59 | 12.24 | 2.77 | 0.32 | 0.02 | 0 | 0 | 0 | 0 |  | 100 | 0 |
| 45 | 48.41 | 29.23 | 16.42 | 5.02 | 0.84 | 0.08 | 0 | 0 | 0 | 0 |  | 57.07 | 27.59 | 12.24 | 2.77 | 0.32 | 0.02 | 0 | 0 | 0 | 0 |  | 100 | 0 |
| 46 | 48.41 | 29.23 | 16.42 | 5.02 | 0.84 | 0.08 | 0 | 0 | 0 | 0 |  | 57.07 | 27.58 | 12.24 | 2.77 | 0.32 | 0.02 | 0 | 0 | 0 | 0 |  | 100 | 0 |
| 47 | 48.41 | 29.23 | 16.42 | 5.02 | 0.84 | 0.08 | 0 | 0 | 0 | 0 |  | 57.07 | 27.58 | 12.24 | 2.77 | 0.32 | 0.02 | 0 | 0 | 0 | 0 |  | 100 | 0 |
| 48 | 48.42 | 29.23 | 16.42 | 5.02 | 0.83 | 0.08 | 0 | 0 | 0 | 0 |  | 57.08 | 27.58 | 12.23 | 2.77 | 0.32 | 0.02 | 0 | 0 | 0 | 0 |  | 100 | 0 |
| 49 | 48.42 | 29.23 | 16.42 | 5.02 | 0.83 | 0.08 | 0 | 0 | 0 | 0 |  | 57.08 | 27.58 | 12.23 | 2.77 | 0.32 | 0.02 | 0 | 0 | 0 | 0 |  | 100 | 0 |
| 50 | 48.42 | 29.23 | 16.42 | 5.02 | 0.83 | 0.08 | 0 | 0 | 0 | 0 |  | 57.31 | 27.52 | 12.13 | 2.72 | 0.31 | 0.02 | 0 | 0 | 0 | 0 |  | 100 | 0 |
| 51 | 48.41 | 29.23 | 16.42 | 5.02 | 0.84 | 0.08 | 0 | 0 | 0 | 0 |  | 57.3 | 27.52 | 12.13 | 2.72 | 0.31 | 0.02 | 0 | 0 | 0 | 0 |  | 100 | 0 |
| 52 | 48.39 | 29.24 | 16.43 | 5.03 | 0.84 | 0.08 | 0 | 0 | 0 | 0 |  | 57.28 | 27.53 | 12.14 | 2.72 | 0.31 | 0.02 | 0 | 0 | 0 | 0 |  | 100 | 0 |
| 53 | 48.35 | 29.25 | 16.45 | 5.03 | 0.84 | 0.08 | 0 | 0 | 0 | 0 |  | 57.24 | 27.55 | 12.15 | 2.73 | 0.31 | 0.02 | 0 | 0 | 0 | 0 |  | 100 | 0 |
| 54 | 48.3 | 29.27 | 16.47 | 5.04 | 0.84 | 0.08 | 0 | 0 | 0 | 0 |  | 57.19 | 27.57 | 12.17 | 2.73 | 0.31 | 0.02 | 0 | 0 | 0 | 0 |  | 100 | 0 |
| 55 | 48.22 | 29.3 | 16.5 | 5.06 | 0.84 | 0.08 | 0 | 0 | 0 | 0 |  | 57.35 | 27.54 | 12.09 | 2.7 | 0.3 | 0.02 | 0 | 0 | 0 | 0 |  | 100 | 0 |
| 56 | 48.14 | 29.33 | 16.54 | 5.07 | 0.84 | 0.08 | 0 | 0 | 0 | 0 |  | 57.26 | 27.59 | 12.12 | 2.7 | 0.3 | 0.02 | 0 | 0 | 0 | 0 |  | 100 | 0 |
| 57 | 48.06 | 29.35 | 16.57 | 5.09 | 0.85 | 0.08 | 0 | 0 | 0 | 0 |  | 57.18 | 27.62 | 12.16 | 2.71 | 0.31 | 0.02 | 0 | 0 | 0 | 0 |  | 100 | 0 |
| 58 | 48.02 | 29.37 | 16.59 | 5.09 | 0.85 | 0.08 | 0 | 0 | 0 | 0 |  | 57.15 | 27.64 | 12.17 | 2.72 | 0.31 | 0.02 | 0 | 0 | 0 | 0 |  | 100 | 0 |
| 59 | 48.09 | 29.34 | 16.56 | 5.08 | 0.84 | 0.08 | 0 | 0 | 0 | 0 |  | 57.22 | 27.61 | 12.14 | 2.71 | 0.31 | 0.02 | 0 | 0 | 0 | 0 |  | 100 | 0 |
| 60 | 48.35 | 29.25 | 16.45 | 5.03 | 0.84 | 0.08 | 0 | 0 | 0 | 0 |  | 57.7 | 27.42 | 11.93 | 2.63 | 0.29 | 0.02 | 0 | 0 | 0 | 0 |  | 100 | 0 |
| 61 | 48.89 | 29.06 | 16.22 | 4.94 | 0.82 | 0.07 | 0 | 0 | 0 | 0 |  | 58.24 | 27.16 | 11.72 | 2.57 | 0.29 | 0.02 | 0 | 0 | 0 | 0 |  | 100 | 0 |
| 62 | 49.81 | 28.73 | 15.82 | 4.78 | 0.79 | 0.07 | 0 | 0 | 0 | 0 |  | 59.17 | 26.7 | 11.37 | 2.47 | 0.27 | 0.02 | 0 | 0 | 0 | 0 |  | 100 | 0 |
| 63 | 51.19 | 28.22 | 15.24 | 4.54 | 0.74 | 0.07 | 0 | 0 | 0 | 0 |  | 60.54 | 26.02 | 10.85 | 2.33 | 0.26 | 0.01 | 0 | 0 | 0 | 0 |  | 100 | 0 |
| 64 | 52.93 | 27.55 | 14.51 | 4.25 | 0.69 | 0.06 | 0 | 0 | 0 | 0 |  | 62.26 | 25.14 | 10.21 | 2.15 | 0.23 | 0.01 | 0 | 0 | 0 | 0 |  | 100 | 0 |
| 65 | 54.85 | 26.8 | 13.73 | 3.94 | 0.63 | 0.06 | 0 | 0 | 0 | 0 |  | 64.42 | 24.06 | 9.4 | 1.91 | 0.2 | 0.01 | 0 | 0 | 0 | 0 |  | 100 | 0 |
| 66 | 56.54 | 26.11 | 13.05 | 3.67 | 0.58 | 0.05 | 0 | 0 | 0 | 0 |  | 66.06 | 23.18 | 8.81 | 1.75 | 0.18 | 0.01 | 0 | 0 | 0 | 0 |  | 100 | 0 |
| 67 | 57.83 | 25.56 | 12.53 | 3.48 | 0.55 | 0.05 | 0 | 0 | 0 | 0 |  | 67.31 | 22.5 | 8.37 | 1.64 | 0.17 | 0.01 | 0 | 0 | 0 | 0 |  | 100 | 0 |
| 68 | 58.68 | 25.2 | 12.2 | 3.35 | 0.52 | 0.05 | 0 | 0 | 0 | 0 |  | 68.12 | 22.05 | 8.09 | 1.57 | 0.16 | 0.01 | 0 | 0 | 0 | 0 |  | 100 | 0 |
| 69 | 59.18 | 24.99 | 12 | 3.28 | 0.51 | 0.04 | 0 | 0 | 0 | 0 |  | 68.6 | 21.79 | 7.93 | 1.52 | 0.15 | 0.01 | 0 | 0 | 0 | 0 |  | 100 | 0 |
| 70 | 59.41 | 24.89 | 11.91 | 3.25 | 0.5 | 0.04 | 0 | 0 | 0 | 0 |  | 68.58 | 21.77 | 7.95 | 1.54 | 0.16 | 0.01 | 0 | 0 | 0 | 0 |  | 100 | 0 |
| 71 | 59.47 | 24.86 | 11.89 | 3.24 | 0.5 | 0.04 | 0 | 0 | 0 | 0 |  | 68.64 | 21.73 | 7.92 | 1.53 | 0.16 | 0.01 | 0 | 0 | 0 | 0 |  | 100 | 0 |
| 72 | 59.44 | 24.87 | 11.9 | 3.24 | 0.5 | 0.04 | 0 | 0 | 0 | 0 |  | 68.62 | 21.75 | 7.94 | 1.53 | 0.16 | 0.01 | 0 | 0 | 0 | 0 |  | 100 | 0 |
| 73 | 59.37 | 24.9 | 11.93 | 3.25 | 0.5 | 0.04 | 0 | 0 | 0 | 0 |  | 68.55 | 21.79 | 7.96 | 1.54 | 0.16 | 0.01 | 0 | 0 | 0 | 0 |  | 100 | 0 |
| 74 | 59.29 | 24.94 | 11.96 | 3.26 | 0.5 | 0.04 | 0 | 0 | 0 | 0 |  | 68.47 | 21.83 | 7.99 | 1.55 | 0.16 | 0.01 | 0 | 0 | 0 | 0 |  | 100 | 0 |
| 75 | 59.22 | 24.97 | 11.98 | 3.27 | 0.51 | 0.04 | 0 | 0 | 0 | 0 |  | 68.4 | 21.87 | 8.01 | 1.55 | 0.16 | 0.01 | 0 | 0 | 0 | 0 |  | 100 | 0 |
| 76 | 59.17 | 24.99 | 12.01 | 3.28 | 0.51 | 0.04 | 0 | 0 | 0 | 0 |  | 68.35 | 21.9 | 8.03 | 1.56 | 0.16 | 0.01 | 0 | 0 | 0 | 0 |  | 100 | 0 |
| 77 | 59.14 | 25 | 12.02 | 3.28 | 0.51 | 0.04 | 0 | 0 | 0 | 0 |  | 68.32 | 21.92 | 8.04 | 1.56 | 0.16 | 0.01 | 0 | 0 | 0 | 0 |  | 100 | 0 |
| 78 | 59.12 | 25.01 | 12.03 | 3.29 | 0.51 | 0.04 | 0 | 0 | 0 | 0 |  | 68.3 | 21.92 | 8.04 | 1.56 | 0.16 | 0.01 | 0 | 0 | 0 | 0 |  | 100 | 0 |
| 79 | 59.11 | 25.01 | 12.03 | 3.29 | 0.51 | 0.04 | 0 | 0 | 0 | 0 |  | 68.29 | 21.93 | 8.05 | 1.56 | 0.16 | 0.01 | 0 | 0 | 0 | 0 |  | 100 | 0 |
| 80 | 59.11 | 25.02 | 12.03 | 3.29 | 0.51 | 0.04 | 0 | 0 | 0 | 0 |  | 67.83 | 22.12 | 8.24 | 1.63 | 0.17 | 0.01 | 0 | 0 | 0 | 0 |  | 100 | 0 |
| 81 | 59.11 | 25.01 | 12.03 | 3.29 | 0.51 | 0.04 | 0 | 0 | 0 | 0 |  | 67.83 | 22.12 | 8.24 | 1.63 | 0.17 | 0.01 | 0 | 0 | 0 | 0 |  | 100 | 0 |
| 82 | 59.12 | 25.01 | 12.03 | 3.29 | 0.51 | 0.04 | 0 | 0 | 0 | 0 |  | 67.83 | 22.12 | 8.24 | 1.63 | 0.17 | 0.01 | 0 | 0 | 0 | 0 |  | 100 | 0 |
| 83 | 59.12 | 25.01 | 12.03 | 3.29 | 0.51 | 0.04 | 0 | 0 | 0 | 0 |  | 67.83 | 22.12 | 8.24 | 1.63 | 0.17 | 0.01 | 0 | 0 | 0 | 0 |  | 100 | 0 |
| 84 | 59.12 | 25.01 | 12.02 | 3.29 | 0.51 | 0.04 | 0 | 0 | 0 | 0 |  | 67.84 | 22.12 | 8.24 | 1.63 | 0.17 | 0.01 | 0 | 0 | 0 | 0 |  | 100 | 0 |
| 85 | 59.12 | 25.01 | 12.02 | 3.29 | 0.51 | 0.04 | 0 | 0 | 0 | 0 |  | 67.61 | 22.21 | 8.33 | 1.66 | 0.18 | 0.01 | 0 | 0 | 0 | 0 |  | 100 | 0 |
| 86 | 59.13 | 25.01 | 12.02 | 3.29 | 0.51 | 0.04 | 0 | 0 | 0 | 0 |  | 67.61 | 22.21 | 8.33 | 1.66 | 0.18 | 0.01 | 0 | 0 | 0 | 0 |  | 100 | 0 |
| 87 | 59.13 | 25.01 | 12.02 | 3.29 | 0.51 | 0.04 | 0 | 0 | 0 | 0 |  | 67.61 | 22.21 | 8.33 | 1.66 | 0.18 | 0.01 | 0 | 0 | 0 | 0 |  | 100 | 0 |
| 88 | 59.13 | 25.01 | 12.02 | 3.29 | 0.51 | 0.04 | 0 | 0 | 0 | 0 |  | 67.61 | 22.21 | 8.33 | 1.66 | 0.18 | 0.01 | 0 | 0 | 0 | 0 |  | 100 | 0 |
| 89 | 59.13 | 25.01 | 12.02 | 3.29 | 0.51 | 0.04 | 0 | 0 | 0 | 0 |  | 67.61 | 22.21 | 8.33 | 1.66 | 0.18 | 0.01 | 0 | 0 | 0 | 0 |  | 100 | 0 |
| 90 | 59.13 | 25.01 | 12.02 | 3.29 | 0.51 | 0.04 | 0 | 0 | 0 | 0 |  | 67.61 | 22.21 | 8.33 | 1.66 | 0.18 | 0.01 | 0 | 0 | 0 | 0 |  | 100 | 0 |
| 91 | 59.13 | 25.01 | 12.02 | 3.29 | 0.51 | 0.04 | 0 | 0 | 0 | 0 |  | 67.61 | 22.21 | 8.33 | 1.66 | 0.18 | 0.01 | 0 | 0 | 0 | 0 |  | 100 | 0 |
| 92 | 59.13 | 25.01 | 12.02 | 3.29 | 0.51 | 0.04 | 0 | 0 | 0 | 0 |  | 67.61 | 22.21 | 8.33 | 1.66 | 0.18 | 0.01 | 0 | 0 | 0 | 0 |  | 100 | 0 |
| 93 | 59.13 | 25.01 | 12.02 | 3.29 | 0.51 | 0.04 | 0 | 0 | 0 | 0 |  | 67.61 | 22.21 | 8.33 | 1.66 | 0.18 | 0.01 | 0 | 0 | 0 | 0 |  | 100 | 0 |
| 94 | 59.13 | 25.01 | 12.02 | 3.29 | 0.51 | 0.04 | 0 | 0 | 0 | 0 |  | 67.61 | 22.21 | 8.33 | 1.66 | 0.18 | 0.01 | 0 | 0 | 0 | 0 |  | 100 | 0 |
| 95 | 59.13 | 25.01 | 12.02 | 3.29 | 0.51 | 0.04 | 0 | 0 | 0 | 0 |  | 67.61 | 22.21 | 8.33 | 1.66 | 0.18 | 0.01 | 0 | 0 | 0 | 0 |  | 100 | 0 |

^a^ Rounded to two decimal points

^b^ %E = Percent of total energy intake
