# Supplementary material for: Substituting polyunsaturated fat for saturated fat: A health impact assessment of a fat tax in seven European countries
Source: PLoS One. 2019 Jul 10;14(7):e0218464. doi: 10.1371/journal.pone.0218464 (PMC6619676; doi:10.1371/journal.pone.0218464)
Supplement: S12 Table — (DOCX) [file pone.0218464.s012.docx]

# S12 Table. Proportion of persons in the respective saturated fat intake categories across scenarios in the Netherlands.

| Age | Reference scenario^a^ | | | | | | | | | |  | Fat tax scenario^a^ | | | | | | | | | |  | Guideline scenario | |
| --- | --- | --- | --- | --- | --- | --- | --- | --- | --- | --- | --- | --- | --- | --- | --- | --- | --- | --- | --- | --- | --- | --- | --- | --- |
|  | Category of saturated fat intake (in %E)^b^ | | | | | | | | | |  | Category of saturated fat intake (in %E)^b^ | | | | | | | | | |  | Category of saturated fat intake (in %E)^b^ | |
|  | ≤10 | >10 ≤12 | >12 ≤14 | >14 ≤16 | >16 ≤18 | >18 ≤20 | >20 ≤22 | >22 ≤24 | >24 ≤26 | >26 ≤100 |  | ≤10 | >10 ≤12 | >12 ≤14 | >14 ≤16 | >16 ≤18 | >18 ≤20 | >20 ≤22 | >22 ≤24 | >24 ≤26 | >26 ≤100 |  | ≤10 | >10 ≤100 |
|  |  |  | Males | | | | | | | | | | | | | | | | | | | | | |
| 0 | 100 | 0 | 0 | 0 | 0 | 0 | 0 | 0 | 0 | 0 |  | 100 | 0 | 0 | 0 | 0 | 0 | 0 | 0 | 0 | 0 |  | 100 | 0 |
| 1 | 100 | 0 | 0 | 0 | 0 | 0 | 0 | 0 | 0 | 0 |  | 100 | 0 | 0 | 0 | 0 | 0 | 0 | 0 | 0 | 0 |  | 100 | 0 |
| 2 | 100 | 0 | 0 | 0 | 0 | 0 | 0 | 0 | 0 | 0 |  | 100 | 0 | 0 | 0 | 0 | 0 | 0 | 0 | 0 | 0 |  | 100 | 0 |
| 3 | 100 | 0 | 0 | 0 | 0 | 0 | 0 | 0 | 0 | 0 |  | 100 | 0 | 0 | 0 | 0 | 0 | 0 | 0 | 0 | 0 |  | 100 | 0 |
| 4 | 100 | 0 | 0 | 0 | 0 | 0 | 0 | 0 | 0 | 0 |  | 100 | 0 | 0 | 0 | 0 | 0 | 0 | 0 | 0 | 0 |  | 100 | 0 |
| 5 | 100 | 0 | 0 | 0 | 0 | 0 | 0 | 0 | 0 | 0 |  | 100 | 0 | 0 | 0 | 0 | 0 | 0 | 0 | 0 | 0 |  | 100 | 0 |
| 6 | 15.45 | 27.45 | 31.62 | 18.77 | 5.74 | 0.9 | 0.07 | 0 | 0 | 0 |  | 15.45 | 27.45 | 31.62 | 18.77 | 5.74 | 0.9 | 0.07 | 0 | 0 | 0 |  | 100 | 0 |
| 7 | 14.57 | 24.25 | 29.87 | 20.89 | 8.29 | 1.86 | 0.24 | 0.02 | 0 | 0 |  | 14.57 | 24.25 | 29.87 | 20.89 | 8.29 | 1.86 | 0.24 | 0.02 | 0 | 0 |  | 100 | 0 |
| 8 | 13.62 | 21.74 | 28.19 | 22.18 | 10.6 | 3.07 | 0.54 | 0.06 | 0 | 0 |  | 13.62 | 21.74 | 28.19 | 22.18 | 10.6 | 3.07 | 0.54 | 0.06 | 0 | 0 |  | 100 | 0 |
| 9 | 12.56 | 19.8 | 26.82 | 22.99 | 12.47 | 4.28 | 0.93 | 0.13 | 0.01 | 0 |  | 12.56 | 19.8 | 26.82 | 22.99 | 12.47 | 4.28 | 0.93 | 0.13 | 0.01 | 0 |  | 100 | 0 |
| 10 | 11.5 | 18.33 | 25.8 | 23.55 | 13.92 | 5.33 | 1.32 | 0.21 | 0.02 | 0 |  | 11.5 | 18.33 | 25.8 | 23.55 | 13.92 | 5.33 | 1.32 | 0.21 | 0.02 | 0 |  | 100 | 0 |
| 11 | 10.59 | 17.27 | 25.11 | 23.95 | 14.97 | 6.13 | 1.65 | 0.29 | 0.03 | 0 |  | 10.59 | 17.27 | 25.11 | 23.95 | 14.97 | 6.13 | 1.65 | 0.29 | 0.03 | 0 |  | 100 | 0 |
| 12 | 9.92 | 16.58 | 24.7 | 24.24 | 15.66 | 6.66 | 1.86 | 0.34 | 0.04 | 0 |  | 9.92 | 16.58 | 24.7 | 24.24 | 15.66 | 6.66 | 1.86 | 0.34 | 0.04 | 0 |  | 100 | 0 |
| 13 | 9.45 | 16.16 | 24.49 | 24.45 | 16.08 | 6.96 | 1.98 | 0.37 | 0.05 | 0 |  | 9.45 | 16.16 | 24.49 | 24.45 | 16.08 | 6.96 | 1.98 | 0.37 | 0.05 | 0 |  | 100 | 0 |
| 14 | 9.17 | 15.93 | 24.4 | 24.6 | 16.31 | 7.12 | 2.04 | 0.38 | 0.05 | 0 |  | 9.17 | 15.93 | 24.4 | 24.6 | 16.31 | 7.12 | 2.04 | 0.38 | 0.05 | 0 |  | 100 | 0 |
| 15 | 9.02 | 15.79 | 24.33 | 24.67 | 16.45 | 7.21 | 2.08 | 0.39 | 0.05 | 0 |  | 12 | 19.45 | 26.82 | 23.34 | 12.82 | 4.44 | 0.97 | 0.13 | 0.01 | 0 |  | 100 | 0 |
| 16 | 8.95 | 15.68 | 24.23 | 24.67 | 16.55 | 7.32 | 2.13 | 0.41 | 0.05 | 0 |  | 11.91 | 19.33 | 26.74 | 23.4 | 12.95 | 4.53 | 1 | 0.14 | 0.01 | 0 |  | 100 | 0 |
| 17 | 8.93 | 15.55 | 24.05 | 24.62 | 16.68 | 7.47 | 2.21 | 0.43 | 0.06 | 0 |  | 11.86 | 19.15 | 26.57 | 23.42 | 13.12 | 4.67 | 1.05 | 0.15 | 0.01 | 0 |  | 100 | 0 |
| 18 | 8.93 | 15.36 | 23.77 | 24.5 | 16.84 | 7.71 | 2.35 | 0.48 | 0.06 | 0.01 |  | 11.81 | 18.91 | 26.29 | 23.42 | 13.36 | 4.88 | 1.14 | 0.17 | 0.02 | 0 |  | 100 | 0 |
| 19 | 8.93 | 15.16 | 23.45 | 24.36 | 17 | 7.97 | 2.51 | 0.53 | 0.08 | 0.01 |  | 11.82 | 18.68 | 26 | 23.38 | 13.58 | 5.09 | 1.23 | 0.19 | 0.02 | 0 |  | 100 | 0 |
| 20 | 8.94 | 14.97 | 23.14 | 24.22 | 17.16 | 8.22 | 2.67 | 0.58 | 0.09 | 0.01 |  | 11.72 | 18.34 | 25.66 | 23.39 | 13.9 | 5.38 | 1.36 | 0.22 | 0.02 | 0 |  | 100 | 0 |
| 21 | 8.94 | 14.82 | 22.91 | 24.11 | 17.27 | 8.42 | 2.79 | 0.63 | 0.1 | 0.01 |  | 11.7 | 18.14 | 25.42 | 23.36 | 14.09 | 5.57 | 1.44 | 0.24 | 0.03 | 0 |  | 100 | 0 |
| 22 | 8.95 | 14.72 | 22.75 | 24.02 | 17.34 | 8.55 | 2.88 | 0.66 | 0.1 | 0.01 |  | 11.68 | 18.01 | 25.26 | 23.34 | 14.21 | 5.7 | 1.5 | 0.26 | 0.03 | 0 |  | 100 | 0 |
| 23 | 8.95 | 14.67 | 22.66 | 23.97 | 17.38 | 8.63 | 2.94 | 0.68 | 0.11 | 0.01 |  | 11.67 | 17.93 | 25.16 | 23.33 | 14.29 | 5.78 | 1.54 | 0.27 | 0.03 | 0 |  | 100 | 0 |
| 24 | 8.95 | 14.64 | 22.61 | 23.95 | 17.4 | 8.67 | 2.96 | 0.69 | 0.11 | 0.01 |  | 11.67 | 17.89 | 25.11 | 23.32 | 14.32 | 5.82 | 1.56 | 0.28 | 0.03 | 0 |  | 100 | 0 |
| 25 | 8.95 | 14.63 | 22.59 | 23.94 | 17.41 | 8.68 | 2.97 | 0.7 | 0.11 | 0.01 |  | 11.6 | 17.8 | 25.05 | 23.35 | 14.41 | 5.89 | 1.59 | 0.29 | 0.03 | 0 |  | 100 | 0 |
| 26 | 8.95 | 14.63 | 22.6 | 23.94 | 17.41 | 8.68 | 2.97 | 0.7 | 0.11 | 0.01 |  | 11.6 | 17.8 | 25.05 | 23.35 | 14.41 | 5.89 | 1.59 | 0.29 | 0.03 | 0 |  | 100 | 0 |
| 27 | 8.95 | 14.64 | 22.61 | 23.95 | 17.4 | 8.67 | 2.96 | 0.69 | 0.11 | 0.01 |  | 11.6 | 17.81 | 25.06 | 23.35 | 14.4 | 5.88 | 1.59 | 0.28 | 0.03 | 0 |  | 100 | 0 |
| 28 | 8.95 | 14.64 | 22.62 | 23.96 | 17.4 | 8.66 | 2.96 | 0.69 | 0.11 | 0.01 |  | 11.6 | 17.82 | 25.07 | 23.35 | 14.39 | 5.87 | 1.58 | 0.28 | 0.03 | 0 |  | 100 | 0 |
| 29 | 8.95 | 14.65 | 22.63 | 23.96 | 17.39 | 8.65 | 2.95 | 0.69 | 0.11 | 0.01 |  | 11.6 | 17.83 | 25.09 | 23.35 | 14.38 | 5.86 | 1.58 | 0.28 | 0.03 | 0 |  | 100 | 0 |
| 30 | 8.95 | 14.66 | 22.64 | 23.97 | 17.39 | 8.64 | 2.94 | 0.69 | 0.11 | 0.01 |  | 11.53 | 17.76 | 25.04 | 23.38 | 14.45 | 5.91 | 1.6 | 0.29 | 0.03 | 0 |  | 100 | 0 |
| 31 | 8.95 | 14.66 | 22.65 | 23.97 | 17.39 | 8.64 | 2.94 | 0.68 | 0.11 | 0.01 |  | 11.53 | 17.76 | 25.05 | 23.38 | 14.45 | 5.91 | 1.6 | 0.29 | 0.03 | 0 |  | 100 | 0 |
| 32 | 8.95 | 14.66 | 22.65 | 23.97 | 17.38 | 8.63 | 2.94 | 0.68 | 0.11 | 0.01 |  | 11.53 | 17.76 | 25.05 | 23.39 | 14.45 | 5.9 | 1.6 | 0.29 | 0.03 | 0 |  | 100 | 0 |
| 33 | 8.95 | 14.67 | 22.65 | 23.97 | 17.38 | 8.63 | 2.94 | 0.68 | 0.11 | 0.01 |  | 11.53 | 17.77 | 25.05 | 23.39 | 14.44 | 5.9 | 1.6 | 0.29 | 0.03 | 0 |  | 100 | 0 |
| 34 | 8.95 | 14.67 | 22.65 | 23.97 | 17.38 | 8.63 | 2.94 | 0.68 | 0.11 | 0.01 |  | 11.53 | 17.77 | 25.06 | 23.39 | 14.44 | 5.9 | 1.6 | 0.29 | 0.03 | 0 |  | 100 | 0 |
| 35 | 8.95 | 14.67 | 22.65 | 23.97 | 17.38 | 8.63 | 2.94 | 0.68 | 0.11 | 0.01 |  | 11.53 | 17.76 | 25.05 | 23.39 | 14.45 | 5.91 | 1.6 | 0.29 | 0.03 | 0 |  | 100 | 0 |
| 36 | 8.95 | 14.66 | 22.65 | 23.97 | 17.38 | 8.63 | 2.94 | 0.68 | 0.11 | 0.01 |  | 11.53 | 17.76 | 25.05 | 23.39 | 14.45 | 5.91 | 1.6 | 0.29 | 0.03 | 0 |  | 100 | 0 |
| 37 | 8.95 | 14.66 | 22.65 | 23.97 | 17.38 | 8.63 | 2.94 | 0.68 | 0.11 | 0.01 |  | 11.53 | 17.76 | 25.05 | 23.39 | 14.45 | 5.91 | 1.6 | 0.29 | 0.03 | 0 |  | 100 | 0 |
| 38 | 8.95 | 14.66 | 22.65 | 23.97 | 17.38 | 8.63 | 2.94 | 0.68 | 0.11 | 0.01 |  | 11.53 | 17.76 | 25.05 | 23.39 | 14.45 | 5.91 | 1.6 | 0.29 | 0.03 | 0 |  | 100 | 0 |
| 39 | 8.95 | 14.66 | 22.65 | 23.97 | 17.38 | 8.63 | 2.94 | 0.68 | 0.11 | 0.01 |  | 11.53 | 17.76 | 25.05 | 23.39 | 14.45 | 5.91 | 1.6 | 0.29 | 0.03 | 0 |  | 100 | 0 |
| 40 | 8.95 | 14.66 | 22.65 | 23.97 | 17.38 | 8.63 | 2.94 | 0.68 | 0.11 | 0.01 |  | 11.46 | 17.68 | 25 | 23.41 | 14.53 | 5.97 | 1.62 | 0.29 | 0.03 | 0 |  | 100 | 0 |
| 41 | 8.95 | 14.66 | 22.65 | 23.97 | 17.38 | 8.63 | 2.94 | 0.68 | 0.11 | 0.01 |  | 11.46 | 17.68 | 25 | 23.41 | 14.53 | 5.97 | 1.62 | 0.29 | 0.03 | 0 |  | 100 | 0 |
| 42 | 8.95 | 14.66 | 22.65 | 23.97 | 17.38 | 8.63 | 2.94 | 0.68 | 0.11 | 0.01 |  | 11.46 | 17.68 | 25 | 23.41 | 14.53 | 5.97 | 1.62 | 0.29 | 0.03 | 0 |  | 100 | 0 |
| 43 | 8.95 | 14.66 | 22.65 | 23.97 | 17.38 | 8.64 | 2.94 | 0.68 | 0.11 | 0.01 |  | 11.46 | 17.68 | 25 | 23.41 | 14.53 | 5.97 | 1.62 | 0.29 | 0.03 | 0 |  | 100 | 0 |
| 44 | 8.95 | 14.66 | 22.65 | 23.97 | 17.38 | 8.64 | 2.94 | 0.68 | 0.11 | 0.01 |  | 11.46 | 17.68 | 25 | 23.41 | 14.53 | 5.97 | 1.62 | 0.29 | 0.03 | 0 |  | 100 | 0 |
| 45 | 8.95 | 14.66 | 22.65 | 23.97 | 17.38 | 8.64 | 2.94 | 0.68 | 0.11 | 0.01 |  | 11.45 | 17.67 | 24.99 | 23.42 | 14.54 | 5.98 | 1.63 | 0.29 | 0.03 | 0 |  | 100 | 0 |
| 46 | 8.95 | 14.66 | 22.65 | 23.97 | 17.39 | 8.64 | 2.94 | 0.68 | 0.11 | 0.01 |  | 11.45 | 17.67 | 24.99 | 23.41 | 14.54 | 5.98 | 1.63 | 0.29 | 0.03 | 0 |  | 100 | 0 |
| 47 | 8.95 | 14.66 | 22.64 | 23.97 | 17.39 | 8.64 | 2.94 | 0.68 | 0.11 | 0.01 |  | 11.45 | 17.67 | 24.99 | 23.41 | 14.54 | 5.98 | 1.63 | 0.29 | 0.03 | 0 |  | 100 | 0 |
| 48 | 8.95 | 14.66 | 22.64 | 23.97 | 17.39 | 8.64 | 2.94 | 0.69 | 0.11 | 0.01 |  | 11.45 | 17.67 | 24.99 | 23.41 | 14.54 | 5.98 | 1.63 | 0.29 | 0.03 | 0 |  | 100 | 0 |
| 49 | 8.95 | 14.66 | 22.64 | 23.97 | 17.39 | 8.64 | 2.94 | 0.69 | 0.11 | 0.01 |  | 11.45 | 17.67 | 24.98 | 23.41 | 14.54 | 5.98 | 1.63 | 0.29 | 0.03 | 0 |  | 100 | 0 |
| 50 | 8.95 | 14.66 | 22.64 | 23.97 | 17.39 | 8.64 | 2.94 | 0.69 | 0.11 | 0.01 |  | 11.32 | 17.52 | 24.89 | 23.46 | 14.68 | 6.1 | 1.68 | 0.31 | 0.04 | 0 |  | 100 | 0 |
| 51 | 8.95 | 14.66 | 22.65 | 23.97 | 17.38 | 8.64 | 2.94 | 0.68 | 0.11 | 0.01 |  | 11.32 | 17.52 | 24.89 | 23.47 | 14.68 | 6.09 | 1.68 | 0.31 | 0.04 | 0 |  | 100 | 0 |
| 52 | 8.95 | 14.67 | 22.66 | 23.98 | 17.38 | 8.63 | 2.93 | 0.68 | 0.11 | 0.01 |  | 11.32 | 17.53 | 24.9 | 23.47 | 14.67 | 6.08 | 1.67 | 0.3 | 0.04 | 0 |  | 100 | 0 |
| 53 | 8.94 | 14.68 | 22.68 | 23.99 | 17.38 | 8.61 | 2.92 | 0.68 | 0.11 | 0.01 |  | 11.31 | 17.55 | 24.93 | 23.48 | 14.66 | 6.07 | 1.66 | 0.3 | 0.04 | 0 |  | 100 | 0 |
| 54 | 8.94 | 14.69 | 22.71 | 24.01 | 17.37 | 8.59 | 2.9 | 0.67 | 0.11 | 0.01 |  | 11.31 | 17.57 | 24.96 | 23.49 | 14.64 | 6.04 | 1.65 | 0.3 | 0.04 | 0 |  | 100 | 0 |
| 55 | 8.92 | 14.71 | 22.75 | 24.04 | 17.35 | 8.56 | 2.88 | 0.66 | 0.1 | 0.01 |  | 11.29 | 17.59 | 25 | 23.51 | 14.62 | 6.02 | 1.64 | 0.29 | 0.03 | 0 |  | 100 | 0 |
| 56 | 8.91 | 14.74 | 22.8 | 24.07 | 17.34 | 8.52 | 2.85 | 0.65 | 0.1 | 0.01 |  | 11.28 | 17.63 | 25.05 | 23.52 | 14.59 | 5.98 | 1.62 | 0.29 | 0.03 | 0 |  | 100 | 0 |
| 57 | 8.9 | 14.76 | 22.85 | 24.1 | 17.32 | 8.48 | 2.83 | 0.64 | 0.1 | 0.01 |  | 11.28 | 17.66 | 25.1 | 23.54 | 14.56 | 5.94 | 1.6 | 0.28 | 0.03 | 0 |  | 100 | 0 |
| 58 | 8.89 | 14.77 | 22.87 | 24.12 | 17.32 | 8.47 | 2.82 | 0.64 | 0.1 | 0.01 |  | 11.27 | 17.67 | 25.12 | 23.55 | 14.55 | 5.93 | 1.59 | 0.28 | 0.03 | 0 |  | 100 | 0 |
| 59 | 8.9 | 14.75 | 22.83 | 24.09 | 17.33 | 8.5 | 2.84 | 0.65 | 0.1 | 0.01 |  | 11.28 | 17.64 | 25.08 | 23.54 | 14.58 | 5.96 | 1.61 | 0.29 | 0.03 | 0 |  | 100 | 0 |
| 60 | 8.94 | 14.68 | 22.68 | 23.99 | 17.37 | 8.61 | 2.92 | 0.68 | 0.11 | 0.01 |  | 11.24 | 17.47 | 24.87 | 23.51 | 14.74 | 6.13 | 1.69 | 0.31 | 0.04 | 0 |  | 100 | 0 |
| 61 | 9.02 | 14.53 | 22.38 | 23.79 | 17.46 | 8.84 | 3.09 | 0.74 | 0.12 | 0.02 |  | 11.3 | 17.26 | 24.55 | 23.39 | 14.92 | 6.37 | 1.82 | 0.35 | 0.04 | 0 |  | 100 | 0 |
| 62 | 9.16 | 14.28 | 21.87 | 23.44 | 17.58 | 9.23 | 3.39 | 0.87 | 0.16 | 0.02 |  | 11.41 | 16.91 | 24 | 23.16 | 15.2 | 6.78 | 2.05 | 0.42 | 0.06 | 0.01 |  | 100 | 0 |
| 63 | 9.37 | 13.93 | 21.14 | 22.9 | 17.71 | 9.78 | 3.85 | 1.08 | 0.22 | 0.03 |  | 11.56 | 16.41 | 23.21 | 22.81 | 15.56 | 7.37 | 2.42 | 0.55 | 0.09 | 0.01 |  | 100 | 0 |
| 64 | 9.63 | 13.49 | 20.25 | 22.21 | 17.79 | 10.41 | 4.45 | 1.39 | 0.32 | 0.06 |  | 11.77 | 15.81 | 22.25 | 22.31 | 15.93 | 8.1 | 2.93 | 0.76 | 0.14 | 0.02 |  | 100 | 0 |
| 65 | 9.92 | 13.04 | 19.33 | 21.44 | 17.79 | 11.03 | 5.12 | 1.77 | 0.46 | 0.1 |  | 11.9 | 15.09 | 21.17 | 21.72 | 16.29 | 8.93 | 3.58 | 1.05 | 0.22 | 0.04 |  | 100 | 0 |
| 66 | 10.17 | 12.65 | 18.57 | 20.77 | 17.71 | 11.5 | 5.7 | 2.15 | 0.62 | 0.16 |  | 12.1 | 14.58 | 20.33 | 21.16 | 16.44 | 9.54 | 4.13 | 1.33 | 0.32 | 0.07 |  | 100 | 0 |
| 67 | 10.37 | 12.37 | 18.01 | 20.25 | 17.6 | 11.82 | 6.13 | 2.46 | 0.76 | 0.22 |  | 12.25 | 14.2 | 19.71 | 20.72 | 16.51 | 9.96 | 4.56 | 1.58 | 0.41 | 0.1 |  | 100 | 0 |
| 68 | 10.5 | 12.19 | 17.65 | 19.91 | 17.52 | 12.01 | 6.42 | 2.67 | 0.87 | 0.27 |  | 12.35 | 13.96 | 19.31 | 20.43 | 16.53 | 10.23 | 4.84 | 1.75 | 0.48 | 0.12 |  | 100 | 0 |
| 69 | 10.57 | 12.09 | 17.45 | 19.72 | 17.46 | 12.11 | 6.58 | 2.8 | 0.93 | 0.3 |  | 12.41 | 13.82 | 19.08 | 20.26 | 16.53 | 10.37 | 5 | 1.85 | 0.53 | 0.14 |  | 100 | 0 |
| 70 | 10.61 | 12.04 | 17.35 | 19.63 | 17.43 | 12.15 | 6.65 | 2.86 | 0.96 | 0.32 |  | 12.39 | 13.71 | 18.94 | 20.17 | 16.56 | 10.48 | 5.12 | 1.93 | 0.56 | 0.15 |  | 100 | 0 |
| 71 | 10.62 | 12.03 | 17.33 | 19.61 | 17.43 | 12.16 | 6.67 | 2.87 | 0.97 | 0.32 |  | 12.4 | 13.7 | 18.91 | 20.14 | 16.56 | 10.5 | 5.14 | 1.94 | 0.56 | 0.15 |  | 100 | 0 |
| 72 | 10.61 | 12.03 | 17.34 | 19.62 | 17.43 | 12.16 | 6.66 | 2.86 | 0.97 | 0.32 |  | 12.39 | 13.7 | 18.92 | 20.15 | 16.56 | 10.49 | 5.13 | 1.93 | 0.56 | 0.15 |  | 100 | 0 |
| 73 | 10.6 | 12.05 | 17.37 | 19.65 | 17.44 | 12.14 | 6.64 | 2.85 | 0.96 | 0.31 |  | 12.38 | 13.72 | 18.95 | 20.18 | 16.56 | 10.47 | 5.1 | 1.92 | 0.56 | 0.15 |  | 100 | 0 |
| 74 | 10.59 | 12.06 | 17.4 | 19.68 | 17.45 | 12.13 | 6.61 | 2.83 | 0.95 | 0.31 |  | 12.38 | 13.74 | 18.99 | 20.21 | 16.56 | 10.45 | 5.08 | 1.9 | 0.55 | 0.14 |  | 100 | 0 |
| 75 | 10.58 | 12.08 | 17.43 | 19.7 | 17.46 | 12.12 | 6.59 | 2.81 | 0.94 | 0.3 |  | 12.32 | 13.72 | 18.98 | 20.22 | 16.59 | 10.48 | 5.1 | 1.91 | 0.55 | 0.15 |  | 100 | 0 |
| 76 | 10.57 | 12.09 | 17.45 | 19.72 | 17.46 | 12.11 | 6.57 | 2.8 | 0.93 | 0.3 |  | 12.31 | 13.73 | 19 | 20.24 | 16.59 | 10.46 | 5.08 | 1.9 | 0.55 | 0.14 |  | 100 | 0 |
| 77 | 10.57 | 12.1 | 17.46 | 19.74 | 17.46 | 12.1 | 6.56 | 2.79 | 0.93 | 0.3 |  | 12.31 | 13.74 | 19.02 | 20.25 | 16.59 | 10.46 | 5.07 | 1.89 | 0.54 | 0.14 |  | 100 | 0 |
| 78 | 10.56 | 12.1 | 17.47 | 19.74 | 17.47 | 12.1 | 6.56 | 2.78 | 0.92 | 0.3 |  | 12.3 | 13.74 | 19.02 | 20.26 | 16.59 | 10.45 | 5.06 | 1.89 | 0.54 | 0.14 |  | 100 | 0 |
| 79 | 10.56 | 12.1 | 17.47 | 19.75 | 17.47 | 12.1 | 6.55 | 2.78 | 0.92 | 0.3 |  | 12.3 | 13.75 | 19.03 | 20.26 | 16.59 | 10.45 | 5.06 | 1.88 | 0.54 | 0.14 |  | 100 | 0 |
| 80 | 10.56 | 12.1 | 17.47 | 19.75 | 17.47 | 12.1 | 6.55 | 2.78 | 0.92 | 0.3 |  | 12.25 | 13.7 | 18.99 | 20.25 | 16.62 | 10.49 | 5.1 | 1.9 | 0.55 | 0.14 |  | 100 | 0 |
| 81 | 10.56 | 12.1 | 17.47 | 19.75 | 17.47 | 12.1 | 6.55 | 2.78 | 0.92 | 0.3 |  | 12.25 | 13.7 | 18.99 | 20.25 | 16.62 | 10.49 | 5.1 | 1.9 | 0.55 | 0.14 |  | 100 | 0 |
| 82 | 10.56 | 12.1 | 17.47 | 19.74 | 17.47 | 12.1 | 6.56 | 2.78 | 0.92 | 0.3 |  | 12.25 | 13.7 | 18.99 | 20.25 | 16.62 | 10.5 | 5.1 | 1.91 | 0.55 | 0.14 |  | 100 | 0 |
| 83 | 10.56 | 12.1 | 17.47 | 19.74 | 17.47 | 12.1 | 6.56 | 2.78 | 0.92 | 0.3 |  | 12.25 | 13.7 | 18.99 | 20.25 | 16.62 | 10.5 | 5.1 | 1.91 | 0.55 | 0.14 |  | 100 | 0 |
| 84 | 10.56 | 12.1 | 17.47 | 19.74 | 17.47 | 12.1 | 6.56 | 2.78 | 0.92 | 0.3 |  | 12.25 | 13.7 | 18.98 | 20.25 | 16.62 | 10.5 | 5.1 | 1.91 | 0.55 | 0.14 |  | 100 | 0 |
| 85 | 10.56 | 12.1 | 17.47 | 19.74 | 17.47 | 12.1 | 6.56 | 2.78 | 0.92 | 0.3 |  | 12.16 | 13.61 | 18.9 | 20.23 | 16.67 | 10.59 | 5.18 | 1.95 | 0.56 | 0.15 |  | 100 | 0 |
| 86 | 10.56 | 12.1 | 17.47 | 19.74 | 17.47 | 12.1 | 6.56 | 2.78 | 0.92 | 0.3 |  | 12.16 | 13.61 | 18.9 | 20.23 | 16.67 | 10.59 | 5.18 | 1.95 | 0.57 | 0.15 |  | 100 | 0 |
| 87 | 10.56 | 12.1 | 17.47 | 19.74 | 17.47 | 12.1 | 6.56 | 2.78 | 0.92 | 0.3 |  | 12.16 | 13.61 | 18.9 | 20.23 | 16.67 | 10.59 | 5.18 | 1.95 | 0.57 | 0.15 |  | 100 | 0 |
| 88 | 10.56 | 12.1 | 17.47 | 19.74 | 17.47 | 12.1 | 6.56 | 2.78 | 0.92 | 0.3 |  | 12.16 | 13.61 | 18.9 | 20.23 | 16.67 | 10.59 | 5.18 | 1.95 | 0.57 | 0.15 |  | 100 | 0 |
| 89 | 10.56 | 12.1 | 17.47 | 19.74 | 17.47 | 12.1 | 6.56 | 2.78 | 0.92 | 0.3 |  | 12.16 | 13.61 | 18.9 | 20.23 | 16.67 | 10.59 | 5.18 | 1.95 | 0.57 | 0.15 |  | 100 | 0 |
| 90 | 10.56 | 12.1 | 17.47 | 19.74 | 17.47 | 12.1 | 6.56 | 2.78 | 0.92 | 0.3 |  | 12.16 | 13.61 | 18.9 | 20.23 | 16.67 | 10.59 | 5.18 | 1.95 | 0.57 | 0.15 |  | 100 | 0 |
| 91 | 10.56 | 12.1 | 17.47 | 19.74 | 17.47 | 12.1 | 6.56 | 2.78 | 0.92 | 0.3 |  | 12.16 | 13.61 | 18.9 | 20.23 | 16.67 | 10.59 | 5.18 | 1.95 | 0.57 | 0.15 |  | 100 | 0 |
| 92 | 10.56 | 12.1 | 17.47 | 19.74 | 17.47 | 12.1 | 6.56 | 2.78 | 0.92 | 0.3 |  | 12.16 | 13.61 | 18.9 | 20.23 | 16.67 | 10.59 | 5.18 | 1.95 | 0.57 | 0.15 |  | 100 | 0 |
| 93 | 10.56 | 12.1 | 17.47 | 19.74 | 17.47 | 12.1 | 6.56 | 2.78 | 0.92 | 0.3 |  | 12.16 | 13.61 | 18.9 | 20.23 | 16.67 | 10.59 | 5.18 | 1.95 | 0.57 | 0.15 |  | 100 | 0 |
| 94 | 10.56 | 12.1 | 17.47 | 19.74 | 17.47 | 12.1 | 6.56 | 2.78 | 0.92 | 0.3 |  | 12.16 | 13.61 | 18.9 | 20.23 | 16.67 | 10.59 | 5.18 | 1.95 | 0.57 | 0.15 |  | 100 | 0 |
| 95 | 10.56 | 12.1 | 17.47 | 19.74 | 17.47 | 12.1 | 6.56 | 2.78 | 0.92 | 0.3 |  | 12.16 | 13.61 | 18.9 | 20.23 | 16.67 | 10.59 | 5.18 | 1.95 | 0.57 | 0.15 |  | 100 | 0 |
|  | |  | Females | | | | | | | | | | | | | | | | | | | | | |
| 0 | 100 | 0 | 0 | 0 | 0 | 0 | 0 | 0 | 0 | 0 |  | 100 | 0 | 0 | 0 | 0 | 0 | 0 | 0 | 0 | 0 |  | 100 | 0 |
| 1 | 100 | 0 | 0 | 0 | 0 | 0 | 0 | 0 | 0 | 0 |  | 100 | 0 | 0 | 0 | 0 | 0 | 0 | 0 | 0 | 0 |  | 100 | 0 |
| 2 | 100 | 0 | 0 | 0 | 0 | 0 | 0 | 0 | 0 | 0 |  | 100 | 0 | 0 | 0 | 0 | 0 | 0 | 0 | 0 | 0 |  | 100 | 0 |
| 3 | 100 | 0 | 0 | 0 | 0 | 0 | 0 | 0 | 0 | 0 |  | 100 | 0 | 0 | 0 | 0 | 0 | 0 | 0 | 0 | 0 |  | 100 | 0 |
| 4 | 100 | 0 | 0 | 0 | 0 | 0 | 0 | 0 | 0 | 0 |  | 100 | 0 | 0 | 0 | 0 | 0 | 0 | 0 | 0 | 0 |  | 100 | 0 |
| 5 | 100 | 0 | 0 | 0 | 0 | 0 | 0 | 0 | 0 | 0 |  | 100 | 0 | 0 | 0 | 0 | 0 | 0 | 0 | 0 | 0 |  | 100 | 0 |
| 6 | 11.93 | 24.85 | 32.48 | 21.8 | 7.5 | 1.32 | 0.12 | 0 | 0 | 0 |  | 11.93 | 24.85 | 32.48 | 21.8 | 7.5 | 1.32 | 0.12 | 0 | 0 | 0 |  | 100 | 0 |
| 7 | 10.67 | 21.22 | 30 | 24.01 | 10.87 | 2.78 | 0.4 | 0.03 | 0 | 0 |  | 10.67 | 21.22 | 30 | 24.01 | 10.87 | 2.78 | 0.4 | 0.03 | 0 | 0 |  | 100 | 0 |
| 8 | 9.88 | 18.74 | 27.71 | 24.81 | 13.45 | 4.41 | 0.87 | 0.1 | 0.01 | 0 |  | 9.88 | 18.74 | 27.71 | 24.81 | 13.45 | 4.41 | 0.87 | 0.1 | 0.01 | 0 |  | 100 | 0 |
| 9 | 9.43 | 17.24 | 26.08 | 24.91 | 15.02 | 5.72 | 1.37 | 0.21 | 0.02 | 0 |  | 9.43 | 17.24 | 26.08 | 24.91 | 15.02 | 5.72 | 1.37 | 0.21 | 0.02 | 0 |  | 100 | 0 |
| 10 | 9.18 | 16.39 | 25.08 | 24.82 | 15.88 | 6.56 | 1.75 | 0.3 | 0.03 | 0 |  | 9.18 | 16.39 | 25.08 | 24.82 | 15.88 | 6.56 | 1.75 | 0.3 | 0.03 | 0 |  | 100 | 0 |
| 11 | 9.07 | 15.98 | 24.58 | 24.73 | 16.27 | 7 | 1.97 | 0.36 | 0.04 | 0 |  | 9.07 | 15.98 | 24.58 | 24.73 | 16.27 | 7 | 1.97 | 0.36 | 0.04 | 0 |  | 100 | 0 |
| 12 | 9.02 | 15.82 | 24.38 | 24.69 | 16.42 | 7.17 | 2.06 | 0.39 | 0.05 | 0 |  | 9.02 | 15.82 | 24.38 | 24.69 | 16.42 | 7.17 | 2.06 | 0.39 | 0.05 | 0 |  | 100 | 0 |
| 13 | 9.02 | 15.8 | 24.35 | 24.68 | 16.44 | 7.2 | 2.07 | 0.39 | 0.05 | 0 |  | 9.02 | 15.8 | 24.35 | 24.68 | 16.44 | 7.2 | 2.07 | 0.39 | 0.05 | 0 |  | 100 | 0 |
| 14 | 9.03 | 15.8 | 24.34 | 24.67 | 16.44 | 7.2 | 2.08 | 0.39 | 0.05 | 0 |  | 9.03 | 15.8 | 24.34 | 24.67 | 16.44 | 7.2 | 2.08 | 0.39 | 0.05 | 0 |  | 100 | 0 |
| 15 | 9.03 | 15.74 | 24.25 | 24.63 | 16.49 | 7.28 | 2.11 | 0.4 | 0.05 | 0 |  | 10.86 | 18.05 | 25.95 | 23.96 | 14.21 | 5.41 | 1.32 | 0.21 | 0.02 | 0 |  | 100 | 0 |
| 16 | 9.03 | 15.58 | 24.02 | 24.54 | 16.63 | 7.47 | 2.23 | 0.44 | 0.06 | 0 |  | 10.83 | 17.86 | 25.72 | 23.94 | 14.4 | 5.6 | 1.4 | 0.23 | 0.02 | 0 |  | 100 | 0 |
| 17 | 9 | 15.29 | 23.59 | 24.38 | 16.89 | 7.84 | 2.44 | 0.51 | 0.07 | 0.01 |  | 10.77 | 17.5 | 25.29 | 23.88 | 14.74 | 5.95 | 1.57 | 0.27 | 0.03 | 0 |  | 100 | 0 |
| 18 | 8.95 | 14.87 | 22.98 | 24.13 | 17.23 | 8.36 | 2.76 | 0.62 | 0.09 | 0.01 |  | 10.67 | 17 | 24.68 | 23.78 | 15.21 | 6.46 | 1.82 | 0.34 | 0.04 | 0 |  | 100 | 0 |
| 19 | 8.91 | 14.42 | 22.31 | 23.82 | 17.56 | 8.94 | 3.14 | 0.76 | 0.13 | 0.02 |  | 10.65 | 16.56 | 24.08 | 23.61 | 15.6 | 6.95 | 2.08 | 0.42 | 0.06 | 0.01 |  | 100 | 0 |
| 20 | 8.86 | 14.03 | 21.71 | 23.52 | 17.82 | 9.45 | 3.51 | 0.91 | 0.16 | 0.02 |  | 10.55 | 16.07 | 23.46 | 23.43 | 16.01 | 7.48 | 2.39 | 0.52 | 0.08 | 0.01 |  | 100 | 0 |
| 21 | 8.83 | 13.73 | 21.25 | 23.26 | 18 | 9.84 | 3.81 | 1.04 | 0.2 | 0.03 |  | 10.48 | 15.71 | 23 | 23.28 | 16.29 | 7.89 | 2.64 | 0.61 | 0.1 | 0.01 |  | 100 | 0 |
| 22 | 8.82 | 13.53 | 20.95 | 23.08 | 18.1 | 10.11 | 4.01 | 1.13 | 0.23 | 0.04 |  | 10.44 | 15.47 | 22.68 | 23.16 | 16.47 | 8.16 | 2.82 | 0.68 | 0.11 | 0.01 |  | 100 | 0 |
| 23 | 8.8 | 13.42 | 20.77 | 22.98 | 18.16 | 10.26 | 4.14 | 1.19 | 0.24 | 0.04 |  | 10.41 | 15.33 | 22.49 | 23.09 | 16.57 | 8.32 | 2.92 | 0.72 | 0.12 | 0.02 |  | 100 | 0 |
| 24 | 8.8 | 13.36 | 20.69 | 22.92 | 18.19 | 10.33 | 4.2 | 1.22 | 0.25 | 0.04 |  | 10.4 | 15.26 | 22.4 | 23.05 | 16.62 | 8.4 | 2.97 | 0.74 | 0.13 | 0.02 |  | 100 | 0 |
| 25 | 8.8 | 13.35 | 20.66 | 22.91 | 18.19 | 10.35 | 4.22 | 1.23 | 0.26 | 0.04 |  | 10.45 | 15.3 | 22.43 | 23.04 | 16.59 | 8.37 | 2.96 | 0.73 | 0.13 | 0.02 |  | 100 | 0 |
| 26 | 8.8 | 13.35 | 20.67 | 22.91 | 18.19 | 10.34 | 4.21 | 1.23 | 0.26 | 0.04 |  | 10.45 | 15.31 | 22.43 | 23.04 | 16.58 | 8.36 | 2.95 | 0.73 | 0.13 | 0.02 |  | 100 | 0 |
| 27 | 8.8 | 13.36 | 20.69 | 22.93 | 18.19 | 10.32 | 4.2 | 1.22 | 0.25 | 0.04 |  | 10.45 | 15.32 | 22.46 | 23.05 | 16.57 | 8.34 | 2.94 | 0.73 | 0.12 | 0.02 |  | 100 | 0 |
| 28 | 8.8 | 13.38 | 20.72 | 22.94 | 18.18 | 10.3 | 4.18 | 1.21 | 0.25 | 0.04 |  | 10.46 | 15.35 | 22.48 | 23.06 | 16.55 | 8.32 | 2.92 | 0.72 | 0.12 | 0.02 |  | 100 | 0 |
| 29 | 8.8 | 13.4 | 20.74 | 22.96 | 18.17 | 10.28 | 4.16 | 1.2 | 0.25 | 0.04 |  | 10.46 | 15.36 | 22.51 | 23.07 | 16.54 | 8.3 | 2.91 | 0.71 | 0.12 | 0.02 |  | 100 | 0 |
| 30 | 8.8 | 13.41 | 20.76 | 22.97 | 18.16 | 10.27 | 4.15 | 1.2 | 0.25 | 0.04 |  | 10.57 | 15.5 | 22.63 | 23.07 | 16.42 | 8.16 | 2.83 | 0.69 | 0.12 | 0.02 |  | 100 | 0 |
| 31 | 8.8 | 13.41 | 20.77 | 22.98 | 18.16 | 10.26 | 4.14 | 1.19 | 0.24 | 0.04 |  | 10.57 | 15.51 | 22.64 | 23.07 | 16.42 | 8.15 | 2.83 | 0.68 | 0.12 | 0.02 |  | 100 | 0 |
| 32 | 8.8 | 13.42 | 20.77 | 22.98 | 18.16 | 10.25 | 4.13 | 1.19 | 0.24 | 0.04 |  | 10.57 | 15.51 | 22.64 | 23.08 | 16.41 | 8.15 | 2.82 | 0.68 | 0.12 | 0.02 |  | 100 | 0 |
| 33 | 8.8 | 13.42 | 20.78 | 22.98 | 18.16 | 10.25 | 4.13 | 1.19 | 0.24 | 0.04 |  | 10.57 | 15.52 | 22.65 | 23.08 | 16.41 | 8.15 | 2.82 | 0.68 | 0.12 | 0.02 |  | 100 | 0 |
| 34 | 8.8 | 13.42 | 20.78 | 22.98 | 18.16 | 10.25 | 4.13 | 1.19 | 0.24 | 0.04 |  | 10.57 | 15.52 | 22.65 | 23.08 | 16.41 | 8.14 | 2.82 | 0.68 | 0.12 | 0.02 |  | 100 | 0 |
| 35 | 8.8 | 13.42 | 20.78 | 22.98 | 18.16 | 10.25 | 4.13 | 1.19 | 0.24 | 0.04 |  | 10.57 | 15.52 | 22.65 | 23.08 | 16.41 | 8.15 | 2.82 | 0.68 | 0.12 | 0.02 |  | 100 | 0 |
| 36 | 8.8 | 13.42 | 20.78 | 22.98 | 18.16 | 10.25 | 4.13 | 1.19 | 0.24 | 0.04 |  | 10.57 | 15.51 | 22.65 | 23.08 | 16.41 | 8.15 | 2.82 | 0.68 | 0.12 | 0.02 |  | 100 | 0 |
| 37 | 8.8 | 13.42 | 20.78 | 22.98 | 18.16 | 10.25 | 4.13 | 1.19 | 0.24 | 0.04 |  | 10.57 | 15.51 | 22.65 | 23.08 | 16.41 | 8.15 | 2.82 | 0.68 | 0.12 | 0.02 |  | 100 | 0 |
| 38 | 8.8 | 13.42 | 20.77 | 22.98 | 18.16 | 10.25 | 4.13 | 1.19 | 0.24 | 0.04 |  | 10.57 | 15.51 | 22.64 | 23.08 | 16.41 | 8.15 | 2.82 | 0.68 | 0.12 | 0.02 |  | 100 | 0 |
| 39 | 8.8 | 13.42 | 20.77 | 22.98 | 18.16 | 10.25 | 4.13 | 1.19 | 0.24 | 0.04 |  | 10.57 | 15.51 | 22.64 | 23.08 | 16.41 | 8.15 | 2.82 | 0.68 | 0.12 | 0.02 |  | 100 | 0 |
| 40 | 8.8 | 13.42 | 20.77 | 22.98 | 18.16 | 10.25 | 4.13 | 1.19 | 0.24 | 0.04 |  | 10.68 | 15.63 | 22.74 | 23.06 | 16.31 | 8.04 | 2.76 | 0.66 | 0.11 | 0.01 |  | 100 | 0 |
| 41 | 8.8 | 13.42 | 20.77 | 22.98 | 18.16 | 10.25 | 4.14 | 1.19 | 0.24 | 0.04 |  | 10.68 | 15.63 | 22.74 | 23.06 | 16.31 | 8.04 | 2.76 | 0.66 | 0.11 | 0.01 |  | 100 | 0 |
| 42 | 8.8 | 13.42 | 20.77 | 22.98 | 18.16 | 10.25 | 4.14 | 1.19 | 0.24 | 0.04 |  | 10.68 | 15.63 | 22.74 | 23.06 | 16.31 | 8.04 | 2.76 | 0.66 | 0.11 | 0.01 |  | 100 | 0 |
| 43 | 8.8 | 13.42 | 20.77 | 22.98 | 18.16 | 10.25 | 4.14 | 1.19 | 0.24 | 0.04 |  | 10.68 | 15.63 | 22.74 | 23.06 | 16.31 | 8.04 | 2.76 | 0.66 | 0.11 | 0.01 |  | 100 | 0 |
| 44 | 8.8 | 13.42 | 20.77 | 22.98 | 18.16 | 10.25 | 4.14 | 1.19 | 0.24 | 0.04 |  | 10.68 | 15.63 | 22.74 | 23.06 | 16.31 | 8.04 | 2.76 | 0.66 | 0.11 | 0.01 |  | 100 | 0 |
| 45 | 8.8 | 13.42 | 20.77 | 22.98 | 18.16 | 10.26 | 4.14 | 1.19 | 0.24 | 0.04 |  | 10.68 | 15.63 | 22.74 | 23.06 | 16.31 | 8.04 | 2.76 | 0.66 | 0.11 | 0.01 |  | 100 | 0 |
| 46 | 8.8 | 13.42 | 20.77 | 22.98 | 18.16 | 10.26 | 4.14 | 1.19 | 0.24 | 0.04 |  | 10.68 | 15.63 | 22.74 | 23.06 | 16.31 | 8.04 | 2.76 | 0.66 | 0.11 | 0.01 |  | 100 | 0 |
| 47 | 8.8 | 13.42 | 20.77 | 22.98 | 18.16 | 10.26 | 4.14 | 1.19 | 0.24 | 0.04 |  | 10.68 | 15.63 | 22.74 | 23.06 | 16.31 | 8.04 | 2.76 | 0.66 | 0.11 | 0.01 |  | 100 | 0 |
| 48 | 8.81 | 13.42 | 20.77 | 22.97 | 18.16 | 10.26 | 4.14 | 1.19 | 0.24 | 0.04 |  | 10.68 | 15.63 | 22.74 | 23.06 | 16.31 | 8.04 | 2.76 | 0.66 | 0.11 | 0.01 |  | 100 | 0 |
| 49 | 8.81 | 13.42 | 20.77 | 22.97 | 18.16 | 10.26 | 4.14 | 1.19 | 0.25 | 0.04 |  | 10.68 | 15.63 | 22.73 | 23.06 | 16.31 | 8.04 | 2.76 | 0.66 | 0.11 | 0.01 |  | 100 | 0 |
| 50 | 8.81 | 13.42 | 20.77 | 22.97 | 18.16 | 10.26 | 4.14 | 1.19 | 0.24 | 0.04 |  | 10.73 | 15.69 | 22.78 | 23.05 | 16.25 | 7.98 | 2.73 | 0.65 | 0.11 | 0.01 |  | 100 | 0 |
| 51 | 8.8 | 13.42 | 20.77 | 22.98 | 18.16 | 10.26 | 4.14 | 1.19 | 0.24 | 0.04 |  | 10.73 | 15.69 | 22.79 | 23.06 | 16.25 | 7.98 | 2.73 | 0.65 | 0.11 | 0.01 |  | 100 | 0 |
| 52 | 8.8 | 13.42 | 20.78 | 22.98 | 18.16 | 10.25 | 4.13 | 1.19 | 0.24 | 0.04 |  | 10.73 | 15.7 | 22.8 | 23.06 | 16.25 | 7.98 | 2.73 | 0.65 | 0.11 | 0.01 |  | 100 | 0 |
| 53 | 8.8 | 13.43 | 20.79 | 22.99 | 18.16 | 10.24 | 4.12 | 1.19 | 0.24 | 0.04 |  | 10.72 | 15.7 | 22.81 | 23.07 | 16.25 | 7.97 | 2.72 | 0.64 | 0.11 | 0.01 |  | 100 | 0 |
| 54 | 8.79 | 13.43 | 20.81 | 23.01 | 18.16 | 10.23 | 4.11 | 1.18 | 0.24 | 0.04 |  | 10.71 | 15.71 | 22.83 | 23.08 | 16.24 | 7.95 | 2.71 | 0.64 | 0.1 | 0.01 |  | 100 | 0 |
| 55 | 8.77 | 13.44 | 20.84 | 23.04 | 18.16 | 10.21 | 4.09 | 1.17 | 0.24 | 0.04 |  | 10.76 | 15.79 | 22.91 | 23.1 | 16.18 | 7.87 | 2.66 | 0.62 | 0.1 | 0.01 |  | 100 | 0 |
| 56 | 8.76 | 13.45 | 20.87 | 23.06 | 18.16 | 10.19 | 4.07 | 1.16 | 0.24 | 0.04 |  | 10.74 | 15.8 | 22.94 | 23.12 | 16.17 | 7.85 | 2.64 | 0.62 | 0.1 | 0.01 |  | 100 | 0 |
| 57 | 8.75 | 13.46 | 20.89 | 23.09 | 18.16 | 10.17 | 4.06 | 1.15 | 0.23 | 0.04 |  | 10.73 | 15.82 | 22.97 | 23.14 | 16.16 | 7.83 | 2.63 | 0.61 | 0.1 | 0.01 |  | 100 | 0 |
| 58 | 8.74 | 13.47 | 20.91 | 23.1 | 18.16 | 10.17 | 4.05 | 1.15 | 0.23 | 0.04 |  | 10.73 | 15.83 | 22.99 | 23.14 | 16.16 | 7.82 | 2.62 | 0.61 | 0.1 | 0.01 |  | 100 | 0 |
| 59 | 8.75 | 13.46 | 20.88 | 23.08 | 18.16 | 10.18 | 4.06 | 1.16 | 0.23 | 0.04 |  | 10.74 | 15.81 | 22.96 | 23.13 | 16.16 | 7.84 | 2.63 | 0.61 | 0.1 | 0.01 |  | 100 | 0 |
| 60 | 8.8 | 13.43 | 20.79 | 22.99 | 18.16 | 10.24 | 4.12 | 1.19 | 0.24 | 0.04 |  | 10.83 | 15.82 | 22.91 | 23.06 | 16.14 | 7.85 | 2.66 | 0.62 | 0.1 | 0.01 |  | 100 | 0 |
| 61 | 8.88 | 13.36 | 20.61 | 22.83 | 18.15 | 10.37 | 4.25 | 1.25 | 0.26 | 0.04 |  | 10.91 | 15.72 | 22.7 | 22.93 | 16.19 | 8 | 2.76 | 0.67 | 0.11 | 0.01 |  | 100 | 0 |
| 62 | 9.04 | 13.24 | 20.3 | 22.54 | 18.14 | 10.57 | 4.46 | 1.36 | 0.3 | 0.05 |  | 11.05 | 15.54 | 22.35 | 22.7 | 16.28 | 8.24 | 2.95 | 0.74 | 0.13 | 0.02 |  | 100 | 0 |
| 63 | 9.26 | 13.07 | 19.85 | 22.12 | 18.09 | 10.85 | 4.78 | 1.54 | 0.37 | 0.07 |  | 11.26 | 15.28 | 21.84 | 22.35 | 16.38 | 8.6 | 3.23 | 0.87 | 0.17 | 0.02 |  | 100 | 0 |
| 64 | 9.55 | 12.85 | 19.29 | 21.59 | 18 | 11.19 | 5.18 | 1.79 | 0.46 | 0.1 |  | 11.52 | 14.96 | 21.21 | 21.9 | 16.47 | 9.02 | 3.6 | 1.04 | 0.22 | 0.04 |  | 100 | 0 |
| 65 | 9.86 | 12.62 | 18.7 | 21 | 17.87 | 11.52 | 5.62 | 2.08 | 0.58 | 0.15 |  | 11.88 | 14.68 | 20.6 | 21.41 | 16.48 | 9.4 | 3.97 | 1.24 | 0.29 | 0.06 |  | 100 | 0 |
| 66 | 10.14 | 12.41 | 18.2 | 20.5 | 17.73 | 11.77 | 6 | 2.35 | 0.7 | 0.19 |  | 12.13 | 14.38 | 20.03 | 20.97 | 16.5 | 9.76 | 4.34 | 1.45 | 0.36 | 0.08 |  | 100 | 0 |
| 67 | 10.35 | 12.25 | 17.83 | 20.12 | 17.6 | 11.94 | 6.28 | 2.56 | 0.81 | 0.24 |  | 12.32 | 14.16 | 19.61 | 20.63 | 16.49 | 10.02 | 4.62 | 1.62 | 0.43 | 0.1 |  | 100 | 0 |
| 68 | 10.49 | 12.15 | 17.59 | 19.87 | 17.52 | 12.05 | 6.47 | 2.71 | 0.88 | 0.28 |  | 12.45 | 14.01 | 19.33 | 20.41 | 16.48 | 10.18 | 4.81 | 1.74 | 0.48 | 0.12 |  | 100 | 0 |
| 69 | 10.57 | 12.09 | 17.45 | 19.73 | 17.46 | 12.1 | 6.57 | 2.79 | 0.93 | 0.3 |  | 12.52 | 13.93 | 19.18 | 20.28 | 16.47 | 10.27 | 4.92 | 1.81 | 0.51 | 0.13 |  | 100 | 0 |
| 70 | 10.61 | 12.06 | 17.39 | 19.66 | 17.44 | 12.13 | 6.62 | 2.83 | 0.95 | 0.31 |  | 12.5 | 13.84 | 19.06 | 20.21 | 16.49 | 10.36 | 5.01 | 1.86 | 0.53 | 0.14 |  | 100 | 0 |
| 71 | 10.62 | 12.06 | 17.37 | 19.64 | 17.43 | 12.14 | 6.63 | 2.84 | 0.96 | 0.31 |  | 12.51 | 13.83 | 19.04 | 20.19 | 16.49 | 10.37 | 5.02 | 1.87 | 0.54 | 0.14 |  | 100 | 0 |
| 72 | 10.62 | 12.06 | 17.38 | 19.65 | 17.43 | 12.13 | 6.62 | 2.84 | 0.95 | 0.31 |  | 12.51 | 13.83 | 19.05 | 20.2 | 16.49 | 10.36 | 5.01 | 1.87 | 0.53 | 0.14 |  | 100 | 0 |
| 73 | 10.61 | 12.07 | 17.4 | 19.67 | 17.44 | 12.12 | 6.61 | 2.83 | 0.95 | 0.31 |  | 12.5 | 13.84 | 19.07 | 20.22 | 16.49 | 10.35 | 5 | 1.86 | 0.53 | 0.14 |  | 100 | 0 |
| 74 | 10.59 | 12.08 | 17.42 | 19.69 | 17.45 | 12.12 | 6.59 | 2.81 | 0.94 | 0.31 |  | 12.49 | 13.86 | 19.1 | 20.24 | 16.49 | 10.34 | 4.98 | 1.85 | 0.53 | 0.14 |  | 100 | 0 |
| 75 | 10.58 | 12.09 | 17.44 | 19.71 | 17.46 | 12.11 | 6.58 | 2.8 | 0.93 | 0.3 |  | 12.47 | 13.87 | 19.12 | 20.26 | 16.5 | 10.33 | 4.97 | 1.84 | 0.52 | 0.14 |  | 100 | 0 |
| 76 | 10.57 | 12.09 | 17.45 | 19.73 | 17.46 | 12.1 | 6.57 | 2.79 | 0.93 | 0.3 |  | 12.47 | 13.88 | 19.13 | 20.27 | 16.5 | 10.32 | 4.96 | 1.83 | 0.52 | 0.13 |  | 100 | 0 |
| 77 | 10.57 | 12.1 | 17.46 | 19.74 | 17.46 | 12.1 | 6.56 | 2.79 | 0.93 | 0.3 |  | 12.46 | 13.88 | 19.14 | 20.28 | 16.5 | 10.31 | 4.95 | 1.82 | 0.52 | 0.13 |  | 100 | 0 |
| 78 | 10.56 | 12.1 | 17.47 | 19.74 | 17.47 | 12.1 | 6.56 | 2.78 | 0.92 | 0.3 |  | 12.46 | 13.88 | 19.15 | 20.28 | 16.5 | 10.31 | 4.95 | 1.82 | 0.52 | 0.13 |  | 100 | 0 |
| 79 | 10.56 | 12.1 | 17.47 | 19.74 | 17.47 | 12.1 | 6.56 | 2.78 | 0.92 | 0.3 |  | 12.46 | 13.89 | 19.15 | 20.29 | 16.5 | 10.31 | 4.94 | 1.82 | 0.52 | 0.13 |  | 100 | 0 |
| 80 | 10.56 | 12.1 | 17.47 | 19.75 | 17.47 | 12.1 | 6.56 | 2.78 | 0.92 | 0.3 |  | 12.35 | 13.79 | 19.07 | 20.27 | 16.56 | 10.4 | 5.02 | 1.86 | 0.53 | 0.14 |  | 100 | 0 |
| 81 | 10.56 | 12.1 | 17.47 | 19.74 | 17.47 | 12.1 | 6.56 | 2.78 | 0.92 | 0.3 |  | 12.35 | 13.79 | 19.07 | 20.27 | 16.56 | 10.4 | 5.02 | 1.86 | 0.53 | 0.14 |  | 100 | 0 |
| 82 | 10.56 | 12.1 | 17.47 | 19.74 | 17.47 | 12.1 | 6.56 | 2.78 | 0.92 | 0.3 |  | 12.35 | 13.79 | 19.07 | 20.27 | 16.56 | 10.4 | 5.02 | 1.86 | 0.53 | 0.14 |  | 100 | 0 |
| 83 | 10.56 | 12.1 | 17.47 | 19.74 | 17.47 | 12.1 | 6.56 | 2.78 | 0.92 | 0.3 |  | 12.35 | 13.79 | 19.07 | 20.27 | 16.56 | 10.4 | 5.02 | 1.86 | 0.53 | 0.14 |  | 100 | 0 |
| 84 | 10.56 | 12.1 | 17.47 | 19.74 | 17.47 | 12.1 | 6.56 | 2.78 | 0.92 | 0.3 |  | 12.35 | 13.79 | 19.07 | 20.27 | 16.56 | 10.4 | 5.02 | 1.86 | 0.53 | 0.14 |  | 100 | 0 |
| 85 | 10.56 | 12.1 | 17.47 | 19.74 | 17.47 | 12.1 | 6.56 | 2.78 | 0.92 | 0.3 |  | 12.3 | 13.74 | 19.02 | 20.26 | 16.59 | 10.45 | 5.06 | 1.89 | 0.54 | 0.14 |  | 100 | 0 |
| 86 | 10.56 | 12.1 | 17.47 | 19.74 | 17.47 | 12.1 | 6.56 | 2.78 | 0.92 | 0.3 |  | 12.3 | 13.74 | 19.02 | 20.26 | 16.59 | 10.45 | 5.06 | 1.89 | 0.54 | 0.14 |  | 100 | 0 |
| 87 | 10.56 | 12.1 | 17.47 | 19.74 | 17.47 | 12.1 | 6.56 | 2.78 | 0.92 | 0.3 |  | 12.3 | 13.74 | 19.02 | 20.26 | 16.59 | 10.45 | 5.06 | 1.89 | 0.54 | 0.14 |  | 100 | 0 |
| 88 | 10.56 | 12.1 | 17.47 | 19.74 | 17.47 | 12.1 | 6.56 | 2.78 | 0.92 | 0.3 |  | 12.3 | 13.74 | 19.02 | 20.26 | 16.59 | 10.45 | 5.06 | 1.89 | 0.54 | 0.14 |  | 100 | 0 |
| 89 | 10.56 | 12.1 | 17.47 | 19.74 | 17.47 | 12.1 | 6.56 | 2.78 | 0.92 | 0.3 |  | 12.3 | 13.74 | 19.02 | 20.26 | 16.59 | 10.45 | 5.06 | 1.89 | 0.54 | 0.14 |  | 100 | 0 |
| 90 | 10.56 | 12.1 | 17.47 | 19.74 | 17.47 | 12.1 | 6.56 | 2.78 | 0.92 | 0.3 |  | 12.3 | 13.74 | 19.02 | 20.26 | 16.59 | 10.45 | 5.06 | 1.89 | 0.54 | 0.14 |  | 100 | 0 |
| 91 | 10.56 | 12.1 | 17.47 | 19.74 | 17.47 | 12.1 | 6.56 | 2.78 | 0.92 | 0.3 |  | 12.3 | 13.74 | 19.02 | 20.26 | 16.59 | 10.45 | 5.06 | 1.89 | 0.54 | 0.14 |  | 100 | 0 |
| 92 | 10.56 | 12.1 | 17.47 | 19.74 | 17.47 | 12.1 | 6.56 | 2.78 | 0.92 | 0.3 |  | 12.3 | 13.74 | 19.02 | 20.26 | 16.59 | 10.45 | 5.06 | 1.89 | 0.54 | 0.14 |  | 100 | 0 |
| 93 | 10.56 | 12.1 | 17.47 | 19.74 | 17.47 | 12.1 | 6.56 | 2.78 | 0.92 | 0.3 |  | 12.3 | 13.74 | 19.02 | 20.26 | 16.59 | 10.45 | 5.06 | 1.89 | 0.54 | 0.14 |  | 100 | 0 |
| 94 | 10.56 | 12.1 | 17.47 | 19.74 | 17.47 | 12.1 | 6.56 | 2.78 | 0.92 | 0.3 |  | 12.3 | 13.74 | 19.02 | 20.26 | 16.59 | 10.45 | 5.06 | 1.89 | 0.54 | 0.14 |  | 100 | 0 |
| 95 | 10.56 | 12.1 | 17.47 | 19.74 | 17.47 | 12.1 | 6.56 | 2.78 | 0.92 | 0.3 |  | 12.3 | 13.74 | 19.02 | 20.26 | 16.59 | 10.45 | 5.06 | 1.89 | 0.54 | 0.14 |  | 100 | 0 |

^a^ Rounded to two decimal points

^b^ %E = Percent of total energy intake
