# Supplementary material for: Substituting polyunsaturated fat for saturated fat: A health impact assessment of a fat tax in seven European countries
Source: PLoS One. 2019 Jul 10;14(7):e0218464. doi: 10.1371/journal.pone.0218464 (PMC6619676; doi:10.1371/journal.pone.0218464)
Supplement: S13 Table — (DOCX) [file pone.0218464.s013.docx]

# S13 Table. Proportion of persons in the respective saturated fat intake categories across scenarios in Poland.

| Age | Reference scenario^a^ | | | | | | | | | |  | Fat tax scenario^a^ | | | | | | | | | |  | Guideline scenario | |
| --- | --- | --- | --- | --- | --- | --- | --- | --- | --- | --- | --- | --- | --- | --- | --- | --- | --- | --- | --- | --- | --- | --- | --- | --- |
|  | Category of saturated fat intake (in %E)^b^ | | | | | | | | | |  | Category of saturated fat intake (in %E)^b^ | | | | | | | | | |  | Category of saturated fat intake (in %E)^b^ | |
|  | ≤10 | >10 ≤12 | >12 ≤14 | >14 ≤16 | >16 ≤18 | >18 ≤20 | >20 ≤22 | >22 ≤24 | >24 ≤26 | >26 ≤100 |  | ≤10 | >10 ≤12 | >12 ≤14 | >14 ≤16 | >16 ≤18 | >18 ≤20 | >20 ≤22 | >22 ≤24 | >24 ≤26 | >26 ≤100 |  | ≤10 | >10 ≤100 |
|  |  |  | Males | | | | | | | | | | | | | | | | | | | | | |
| 0 | 100 | 0 | 0 | 0 | 0 | 0 | 0 | 0 | 0 | 0 |  | 100 | 0 | 0 | 0 | 0 | 0 | 0 | 0 | 0 | 0 |  | 100 | 0 |
| 1 | 100 | 0 | 0 | 0 | 0 | 0 | 0 | 0 | 0 | 0 |  | 100 | 0 | 0 | 0 | 0 | 0 | 0 | 0 | 0 | 0 |  | 100 | 0 |
| 2 | 100 | 0 | 0 | 0 | 0 | 0 | 0 | 0 | 0 | 0 |  | 100 | 0 | 0 | 0 | 0 | 0 | 0 | 0 | 0 | 0 |  | 100 | 0 |
| 3 | 100 | 0 | 0 | 0 | 0 | 0 | 0 | 0 | 0 | 0 |  | 100 | 0 | 0 | 0 | 0 | 0 | 0 | 0 | 0 | 0 |  | 100 | 0 |
| 4 | 100 | 0 | 0 | 0 | 0 | 0 | 0 | 0 | 0 | 0 |  | 100 | 0 | 0 | 0 | 0 | 0 | 0 | 0 | 0 | 0 |  | 100 | 0 |
| 5 | 100 | 0 | 0 | 0 | 0 | 0 | 0 | 0 | 0 | 0 |  | 100 | 0 | 0 | 0 | 0 | 0 | 0 | 0 | 0 | 0 |  | 100 | 0 |
| 6 | 32.4 | 20.05 | 19.43 | 14.49 | 8.31 | 3.67 | 1.24 | 0.32 | 0.06 | 0.01 |  | 32.4 | 20.05 | 19.43 | 14.49 | 8.31 | 3.67 | 1.24 | 0.32 | 0.06 | 0.01 |  | 100 | 0 |
| 7 | 31.45 | 20.05 | 19.67 | 14.8 | 8.55 | 3.78 | 1.29 | 0.34 | 0.07 | 0.01 |  | 31.45 | 20.05 | 19.67 | 14.8 | 8.55 | 3.78 | 1.29 | 0.34 | 0.07 | 0.01 |  | 100 | 0 |
| 8 | 30.61 | 20.04 | 19.87 | 15.08 | 8.76 | 3.89 | 1.32 | 0.34 | 0.07 | 0.01 |  | 30.61 | 20.04 | 19.87 | 15.08 | 8.76 | 3.89 | 1.32 | 0.34 | 0.07 | 0.01 |  | 100 | 0 |
| 9 | 29.96 | 20.02 | 20.03 | 15.3 | 8.93 | 3.98 | 1.35 | 0.35 | 0.07 | 0.01 |  | 29.96 | 20.02 | 20.03 | 15.3 | 8.93 | 3.98 | 1.35 | 0.35 | 0.07 | 0.01 |  | 100 | 0 |
| 10 | 29.49 | 20 | 20.13 | 15.46 | 9.05 | 4.04 | 1.38 | 0.36 | 0.07 | 0.01 |  | 29.49 | 20 | 20.13 | 15.46 | 9.05 | 4.04 | 1.38 | 0.36 | 0.07 | 0.01 |  | 100 | 0 |
| 11 | 29.18 | 19.98 | 20.2 | 15.56 | 9.14 | 4.09 | 1.4 | 0.36 | 0.07 | 0.01 |  | 29.18 | 19.98 | 20.2 | 15.56 | 9.14 | 4.09 | 1.4 | 0.36 | 0.07 | 0.01 |  | 100 | 0 |
| 12 | 29.02 | 19.97 | 20.23 | 15.62 | 9.19 | 4.12 | 1.41 | 0.37 | 0.07 | 0.01 |  | 29.02 | 19.97 | 20.23 | 15.62 | 9.19 | 4.12 | 1.41 | 0.37 | 0.07 | 0.01 |  | 100 | 0 |
| 13 | 28.93 | 19.95 | 20.24 | 15.65 | 9.22 | 4.14 | 1.42 | 0.37 | 0.07 | 0.01 |  | 28.93 | 19.95 | 20.24 | 15.65 | 9.22 | 4.14 | 1.42 | 0.37 | 0.07 | 0.01 |  | 100 | 0 |
| 14 | 28.89 | 19.93 | 20.23 | 15.66 | 9.25 | 4.16 | 1.43 | 0.37 | 0.07 | 0.01 |  | 28.89 | 19.93 | 20.23 | 15.66 | 9.25 | 4.16 | 1.43 | 0.37 | 0.07 | 0.01 |  | 100 | 0 |
| 15 | 28.84 | 19.89 | 20.21 | 15.68 | 9.28 | 4.19 | 1.44 | 0.38 | 0.08 | 0.01 |  | 34.3 | 21.76 | 20.03 | 13.63 | 6.86 | 2.55 | 0.7 | 0.14 | 0.02 | 0 |  | 100 | 0 |
| 16 | 28.77 | 19.82 | 20.18 | 15.7 | 9.33 | 4.24 | 1.47 | 0.39 | 0.08 | 0.01 |  | 34.2 | 21.7 | 20.02 | 13.68 | 6.92 | 2.59 | 0.72 | 0.15 | 0.02 | 0 |  | 100 | 0 |
| 17 | 28.64 | 19.73 | 20.14 | 15.74 | 9.42 | 4.31 | 1.51 | 0.4 | 0.08 | 0.02 |  | 34.04 | 21.61 | 20.02 | 13.75 | 7.01 | 2.65 | 0.74 | 0.15 | 0.02 | 0 |  | 100 | 0 |
| 18 | 28.46 | 19.61 | 20.1 | 15.8 | 9.53 | 4.41 | 1.56 | 0.43 | 0.09 | 0.02 |  | 33.8 | 21.49 | 20.01 | 13.86 | 7.13 | 2.73 | 0.78 | 0.16 | 0.03 | 0 |  | 100 | 0 |
| 19 | 28.26 | 19.47 | 20.04 | 15.86 | 9.65 | 4.52 | 1.62 | 0.45 | 0.1 | 0.02 |  | 33.62 | 21.39 | 20 | 13.93 | 7.24 | 2.8 | 0.81 | 0.17 | 0.03 | 0 |  | 100 | 0 |
| 20 | 28.07 | 19.35 | 19.99 | 15.92 | 9.77 | 4.62 | 1.68 | 0.47 | 0.1 | 0.02 |  | 33.26 | 21.24 | 20 | 14.08 | 7.42 | 2.92 | 0.86 | 0.19 | 0.03 | 0 |  | 100 | 0 |
| 21 | 27.93 | 19.26 | 19.96 | 15.96 | 9.85 | 4.7 | 1.73 | 0.49 | 0.11 | 0.02 |  | 33.08 | 21.15 | 19.99 | 14.16 | 7.51 | 2.99 | 0.89 | 0.2 | 0.03 | 0 |  | 100 | 0 |
| 22 | 27.83 | 19.2 | 19.93 | 15.99 | 9.91 | 4.75 | 1.76 | 0.5 | 0.11 | 0.02 |  | 32.95 | 21.09 | 19.99 | 14.21 | 7.58 | 3.03 | 0.91 | 0.2 | 0.03 | 0 |  | 100 | 0 |
| 23 | 27.77 | 19.16 | 19.92 | 16.01 | 9.95 | 4.78 | 1.77 | 0.51 | 0.11 | 0.02 |  | 32.88 | 21.05 | 19.98 | 14.24 | 7.62 | 3.06 | 0.92 | 0.21 | 0.03 | 0 |  | 100 | 0 |
| 24 | 27.74 | 19.15 | 19.91 | 16.01 | 9.96 | 4.79 | 1.78 | 0.51 | 0.11 | 0.02 |  | 32.84 | 21.04 | 19.98 | 14.25 | 7.63 | 3.07 | 0.93 | 0.21 | 0.04 | 0 |  | 100 | 0 |
| 25 | 27.73 | 19.14 | 19.91 | 16.02 | 9.97 | 4.8 | 1.79 | 0.51 | 0.11 | 0.02 |  | 32.7 | 20.99 | 19.99 | 14.31 | 7.7 | 3.11 | 0.94 | 0.22 | 0.04 | 0 |  | 100 | 0 |
| 26 | 27.73 | 19.14 | 19.91 | 16.02 | 9.97 | 4.8 | 1.79 | 0.51 | 0.11 | 0.02 |  | 32.7 | 21 | 19.99 | 14.31 | 7.69 | 3.11 | 0.94 | 0.22 | 0.04 | 0 |  | 100 | 0 |
| 27 | 27.74 | 19.15 | 19.91 | 16.01 | 9.96 | 4.79 | 1.78 | 0.51 | 0.11 | 0.02 |  | 32.71 | 21 | 19.99 | 14.3 | 7.69 | 3.1 | 0.94 | 0.22 | 0.04 | 0 |  | 100 | 0 |
| 28 | 27.75 | 19.15 | 19.91 | 16.01 | 9.96 | 4.79 | 1.78 | 0.51 | 0.11 | 0.02 |  | 32.73 | 21.01 | 19.99 | 14.3 | 7.68 | 3.1 | 0.94 | 0.21 | 0.04 | 0 |  | 100 | 0 |
| 29 | 27.76 | 19.16 | 19.92 | 16.01 | 9.95 | 4.78 | 1.78 | 0.51 | 0.11 | 0.02 |  | 32.73 | 21.01 | 19.99 | 14.29 | 7.68 | 3.1 | 0.94 | 0.21 | 0.04 | 0 |  | 100 | 0 |
| 30 | 27.76 | 19.16 | 19.92 | 16.01 | 9.95 | 4.78 | 1.77 | 0.51 | 0.11 | 0.02 |  | 32.61 | 20.97 | 20 | 14.34 | 7.73 | 3.13 | 0.95 | 0.22 | 0.04 | 0 |  | 100 | 0 |
| 31 | 27.77 | 19.16 | 19.92 | 16.01 | 9.95 | 4.78 | 1.77 | 0.51 | 0.11 | 0.02 |  | 32.62 | 20.97 | 20 | 14.34 | 7.73 | 3.13 | 0.95 | 0.22 | 0.04 | 0 |  | 100 | 0 |
| 32 | 27.77 | 19.17 | 19.92 | 16.01 | 9.95 | 4.78 | 1.77 | 0.51 | 0.11 | 0.02 |  | 32.62 | 20.98 | 20 | 14.34 | 7.72 | 3.13 | 0.95 | 0.22 | 0.04 | 0 |  | 100 | 0 |
| 33 | 27.77 | 19.17 | 19.92 | 16.01 | 9.94 | 4.78 | 1.77 | 0.51 | 0.11 | 0.02 |  | 32.62 | 20.98 | 20 | 14.34 | 7.72 | 3.13 | 0.95 | 0.22 | 0.04 | 0 |  | 100 | 0 |
| 34 | 27.77 | 19.17 | 19.92 | 16.01 | 9.94 | 4.78 | 1.77 | 0.51 | 0.11 | 0.02 |  | 32.62 | 20.98 | 20 | 14.34 | 7.72 | 3.13 | 0.95 | 0.22 | 0.04 | 0 |  | 100 | 0 |
| 35 | 27.77 | 19.17 | 19.92 | 16.01 | 9.94 | 4.78 | 1.77 | 0.51 | 0.11 | 0.02 |  | 32.61 | 20.97 | 20 | 14.34 | 7.73 | 3.13 | 0.95 | 0.22 | 0.04 | 0 |  | 100 | 0 |
| 36 | 27.77 | 19.17 | 19.92 | 16.01 | 9.94 | 4.78 | 1.77 | 0.51 | 0.11 | 0.02 |  | 32.61 | 20.97 | 20 | 14.34 | 7.73 | 3.13 | 0.95 | 0.22 | 0.04 | 0 |  | 100 | 0 |
| 37 | 27.77 | 19.17 | 19.92 | 16.01 | 9.94 | 4.78 | 1.77 | 0.51 | 0.11 | 0.02 |  | 32.61 | 20.97 | 20 | 14.34 | 7.73 | 3.13 | 0.95 | 0.22 | 0.04 | 0 |  | 100 | 0 |
| 38 | 27.77 | 19.17 | 19.92 | 16.01 | 9.95 | 4.78 | 1.77 | 0.51 | 0.11 | 0.02 |  | 32.61 | 20.97 | 20 | 14.34 | 7.73 | 3.13 | 0.95 | 0.22 | 0.04 | 0 |  | 100 | 0 |
| 39 | 27.77 | 19.17 | 19.92 | 16.01 | 9.95 | 4.78 | 1.77 | 0.51 | 0.11 | 0.02 |  | 32.61 | 20.97 | 20 | 14.34 | 7.73 | 3.13 | 0.95 | 0.22 | 0.04 | 0 |  | 100 | 0 |
| 40 | 27.77 | 19.17 | 19.92 | 16.01 | 9.95 | 4.78 | 1.77 | 0.51 | 0.11 | 0.02 |  | 32.49 | 20.93 | 20.01 | 14.39 | 7.78 | 3.17 | 0.97 | 0.22 | 0.04 | 0.01 |  | 100 | 0 |
| 41 | 27.77 | 19.17 | 19.92 | 16.01 | 9.95 | 4.78 | 1.77 | 0.51 | 0.11 | 0.02 |  | 32.49 | 20.93 | 20.01 | 14.39 | 7.78 | 3.17 | 0.97 | 0.22 | 0.04 | 0.01 |  | 100 | 0 |
| 42 | 27.77 | 19.17 | 19.92 | 16.01 | 9.95 | 4.78 | 1.77 | 0.51 | 0.11 | 0.02 |  | 32.49 | 20.93 | 20.01 | 14.39 | 7.78 | 3.17 | 0.97 | 0.22 | 0.04 | 0.01 |  | 100 | 0 |
| 43 | 27.77 | 19.17 | 19.92 | 16.01 | 9.95 | 4.78 | 1.77 | 0.51 | 0.11 | 0.02 |  | 32.49 | 20.93 | 20.01 | 14.39 | 7.78 | 3.17 | 0.97 | 0.22 | 0.04 | 0.01 |  | 100 | 0 |
| 44 | 27.77 | 19.17 | 19.92 | 16.01 | 9.95 | 4.78 | 1.77 | 0.51 | 0.11 | 0.02 |  | 32.49 | 20.93 | 20.01 | 14.39 | 7.78 | 3.17 | 0.97 | 0.22 | 0.04 | 0.01 |  | 100 | 0 |
| 45 | 27.77 | 19.16 | 19.92 | 16.01 | 9.95 | 4.78 | 1.77 | 0.51 | 0.11 | 0.02 |  | 32.48 | 20.93 | 20.01 | 14.39 | 7.79 | 3.17 | 0.97 | 0.22 | 0.04 | 0.01 |  | 100 | 0 |
| 46 | 27.77 | 19.16 | 19.92 | 16.01 | 9.95 | 4.78 | 1.77 | 0.51 | 0.11 | 0.02 |  | 32.48 | 20.93 | 20.01 | 14.39 | 7.79 | 3.17 | 0.97 | 0.22 | 0.04 | 0.01 |  | 100 | 0 |
| 47 | 27.77 | 19.16 | 19.92 | 16.01 | 9.95 | 4.78 | 1.77 | 0.51 | 0.11 | 0.02 |  | 32.47 | 20.93 | 20.01 | 14.39 | 7.79 | 3.17 | 0.97 | 0.22 | 0.04 | 0.01 |  | 100 | 0 |
| 48 | 27.77 | 19.16 | 19.92 | 16.01 | 9.95 | 4.78 | 1.77 | 0.51 | 0.11 | 0.02 |  | 32.47 | 20.93 | 20.01 | 14.39 | 7.79 | 3.17 | 0.97 | 0.22 | 0.04 | 0.01 |  | 100 | 0 |
| 49 | 27.76 | 19.16 | 19.92 | 16.01 | 9.95 | 4.78 | 1.77 | 0.51 | 0.11 | 0.02 |  | 32.47 | 20.93 | 20.01 | 14.39 | 7.79 | 3.17 | 0.97 | 0.22 | 0.04 | 0.01 |  | 100 | 0 |
| 50 | 27.76 | 19.16 | 19.92 | 16.01 | 9.95 | 4.78 | 1.77 | 0.51 | 0.11 | 0.02 |  | 32.24 | 20.85 | 20.02 | 14.48 | 7.89 | 3.24 | 1 | 0.23 | 0.04 | 0.01 |  | 100 | 0 |
| 51 | 27.77 | 19.16 | 19.92 | 16.01 | 9.95 | 4.78 | 1.77 | 0.51 | 0.11 | 0.02 |  | 32.24 | 20.85 | 20.02 | 14.48 | 7.89 | 3.24 | 1 | 0.23 | 0.04 | 0.01 |  | 100 | 0 |
| 52 | 27.77 | 19.17 | 19.92 | 16.01 | 9.94 | 4.77 | 1.77 | 0.51 | 0.11 | 0.02 |  | 32.25 | 20.85 | 20.02 | 14.48 | 7.89 | 3.24 | 1 | 0.23 | 0.04 | 0.01 |  | 100 | 0 |
| 53 | 27.78 | 19.18 | 19.93 | 16 | 9.94 | 4.77 | 1.77 | 0.51 | 0.11 | 0.02 |  | 32.26 | 20.86 | 20.02 | 14.47 | 7.88 | 3.23 | 1 | 0.23 | 0.04 | 0.01 |  | 100 | 0 |
| 54 | 27.79 | 19.19 | 19.93 | 16 | 9.93 | 4.76 | 1.76 | 0.5 | 0.11 | 0.02 |  | 32.27 | 20.87 | 20.02 | 14.47 | 7.87 | 3.22 | 0.99 | 0.23 | 0.04 | 0.01 |  | 100 | 0 |
| 55 | 27.81 | 19.2 | 19.94 | 16 | 9.92 | 4.75 | 1.76 | 0.5 | 0.11 | 0.02 |  | 32.29 | 20.88 | 20.03 | 14.46 | 7.86 | 3.22 | 0.99 | 0.23 | 0.04 | 0.01 |  | 100 | 0 |
| 56 | 27.83 | 19.21 | 19.94 | 15.99 | 9.9 | 4.74 | 1.75 | 0.5 | 0.11 | 0.02 |  | 32.32 | 20.9 | 20.03 | 14.45 | 7.85 | 3.21 | 0.99 | 0.23 | 0.04 | 0.01 |  | 100 | 0 |
| 57 | 27.86 | 19.23 | 19.95 | 15.98 | 9.89 | 4.72 | 1.74 | 0.5 | 0.11 | 0.02 |  | 32.34 | 20.91 | 20.03 | 14.44 | 7.83 | 3.19 | 0.98 | 0.23 | 0.04 | 0.01 |  | 100 | 0 |
| 58 | 27.86 | 19.24 | 19.95 | 15.98 | 9.88 | 4.72 | 1.74 | 0.5 | 0.11 | 0.02 |  | 32.35 | 20.92 | 20.03 | 14.43 | 7.82 | 3.19 | 0.98 | 0.23 | 0.04 | 0.01 |  | 100 | 0 |
| 59 | 27.85 | 19.22 | 19.95 | 15.99 | 9.89 | 4.73 | 1.75 | 0.5 | 0.11 | 0.02 |  | 32.33 | 20.91 | 20.03 | 14.44 | 7.84 | 3.2 | 0.98 | 0.23 | 0.04 | 0.01 |  | 100 | 0 |
| 60 | 27.78 | 19.18 | 19.93 | 16 | 9.94 | 4.77 | 1.77 | 0.51 | 0.11 | 0.02 |  | 32.13 | 20.82 | 20.03 | 14.52 | 7.94 | 3.27 | 1.01 | 0.24 | 0.04 | 0.01 |  | 100 | 0 |
| 61 | 27.65 | 19.08 | 19.88 | 16.04 | 10.02 | 4.85 | 1.82 | 0.53 | 0.12 | 0.02 |  | 31.96 | 20.72 | 20 | 14.59 | 8.04 | 3.34 | 1.05 | 0.25 | 0.04 | 0.01 |  | 100 | 0 |
| 62 | 27.42 | 18.91 | 19.79 | 16.1 | 10.17 | 4.99 | 1.9 | 0.56 | 0.13 | 0.03 |  | 31.67 | 20.55 | 19.96 | 14.7 | 8.2 | 3.47 | 1.11 | 0.27 | 0.05 | 0.01 |  | 100 | 0 |
| 63 | 27.08 | 18.66 | 19.66 | 16.18 | 10.38 | 5.2 | 2.03 | 0.62 | 0.15 | 0.03 |  | 31.25 | 20.3 | 19.89 | 14.86 | 8.45 | 3.66 | 1.21 | 0.3 | 0.06 | 0.01 |  | 100 | 0 |
| 64 | 26.67 | 18.33 | 19.49 | 16.26 | 10.65 | 5.47 | 2.21 | 0.7 | 0.17 | 0.04 |  | 30.73 | 19.97 | 19.79 | 15.04 | 8.77 | 3.92 | 1.34 | 0.35 | 0.07 | 0.01 |  | 100 | 0 |
| 65 | 26.22 | 17.98 | 19.29 | 16.34 | 10.93 | 5.78 | 2.41 | 0.79 | 0.21 | 0.05 |  | 29.99 | 19.55 | 19.66 | 15.29 | 9.18 | 4.26 | 1.53 | 0.42 | 0.09 | 0.02 |  | 100 | 0 |
| 66 | 25.83 | 17.67 | 19.1 | 16.39 | 11.17 | 6.04 | 2.6 | 0.89 | 0.24 | 0.06 |  | 29.51 | 19.23 | 19.54 | 15.43 | 9.48 | 4.52 | 1.68 | 0.48 | 0.11 | 0.02 |  | 100 | 0 |
| 67 | 25.54 | 17.44 | 18.95 | 16.43 | 11.35 | 6.25 | 2.75 | 0.96 | 0.27 | 0.07 |  | 29.14 | 18.99 | 19.43 | 15.53 | 9.7 | 4.73 | 1.8 | 0.54 | 0.12 | 0.03 |  | 100 | 0 |
| 68 | 25.35 | 17.28 | 18.85 | 16.44 | 11.46 | 6.39 | 2.85 | 1.01 | 0.29 | 0.08 |  | 28.9 | 18.82 | 19.36 | 15.59 | 9.84 | 4.87 | 1.88 | 0.57 | 0.14 | 0.03 |  | 100 | 0 |
| 69 | 25.24 | 17.19 | 18.79 | 16.45 | 11.53 | 6.47 | 2.9 | 1.04 | 0.3 | 0.08 |  | 28.77 | 18.73 | 19.31 | 15.62 | 9.92 | 4.94 | 1.93 | 0.59 | 0.14 | 0.03 |  | 100 | 0 |
| 70 | 25.19 | 17.15 | 18.76 | 16.45 | 11.56 | 6.51 | 2.93 | 1.06 | 0.31 | 0.09 |  | 28.61 | 18.64 | 19.28 | 15.67 | 10.01 | 5.02 | 1.98 | 0.61 | 0.15 | 0.03 |  | 100 | 0 |
| 71 | 25.17 | 17.13 | 18.76 | 16.45 | 11.57 | 6.52 | 2.94 | 1.06 | 0.31 | 0.09 |  | 28.59 | 18.63 | 19.28 | 15.67 | 10.02 | 5.03 | 1.99 | 0.62 | 0.15 | 0.03 |  | 100 | 0 |
| 72 | 25.18 | 17.14 | 18.76 | 16.45 | 11.56 | 6.51 | 2.94 | 1.06 | 0.31 | 0.09 |  | 28.6 | 18.64 | 19.28 | 15.67 | 10.01 | 5.03 | 1.98 | 0.62 | 0.15 | 0.03 |  | 100 | 0 |
| 73 | 25.2 | 17.15 | 18.77 | 16.45 | 11.55 | 6.5 | 2.93 | 1.06 | 0.31 | 0.09 |  | 28.62 | 18.65 | 19.28 | 15.67 | 10 | 5.01 | 1.98 | 0.61 | 0.15 | 0.03 |  | 100 | 0 |
| 74 | 25.21 | 17.17 | 18.78 | 16.45 | 11.54 | 6.49 | 2.92 | 1.05 | 0.3 | 0.08 |  | 28.64 | 18.67 | 19.29 | 15.66 | 9.99 | 5 | 1.97 | 0.61 | 0.15 | 0.03 |  | 100 | 0 |
| 75 | 25.23 | 17.18 | 18.79 | 16.45 | 11.53 | 6.48 | 2.91 | 1.05 | 0.3 | 0.08 |  | 28.56 | 18.64 | 19.29 | 15.68 | 10.02 | 5.03 | 1.98 | 0.61 | 0.15 | 0.03 |  | 100 | 0 |
| 76 | 25.24 | 17.19 | 18.79 | 16.45 | 11.53 | 6.47 | 2.9 | 1.04 | 0.3 | 0.08 |  | 28.57 | 18.65 | 19.29 | 15.68 | 10.01 | 5.02 | 1.98 | 0.61 | 0.15 | 0.03 |  | 100 | 0 |
| 77 | 25.25 | 17.2 | 18.8 | 16.45 | 11.52 | 6.46 | 2.9 | 1.04 | 0.3 | 0.08 |  | 28.58 | 18.66 | 19.3 | 15.68 | 10.01 | 5.02 | 1.98 | 0.61 | 0.15 | 0.03 |  | 100 | 0 |
| 78 | 25.25 | 17.2 | 18.8 | 16.45 | 11.52 | 6.46 | 2.9 | 1.04 | 0.3 | 0.08 |  | 28.59 | 18.66 | 19.3 | 15.68 | 10 | 5.01 | 1.97 | 0.61 | 0.15 | 0.03 |  | 100 | 0 |
| 79 | 25.25 | 17.2 | 18.8 | 16.45 | 11.52 | 6.46 | 2.9 | 1.04 | 0.3 | 0.08 |  | 28.59 | 18.66 | 19.3 | 15.68 | 10 | 5.01 | 1.97 | 0.61 | 0.15 | 0.03 |  | 100 | 0 |
| 80 | 25.25 | 17.2 | 18.8 | 16.45 | 11.52 | 6.46 | 2.9 | 1.04 | 0.3 | 0.08 |  | 28.5 | 18.62 | 19.29 | 15.7 | 10.04 | 5.05 | 1.99 | 0.62 | 0.15 | 0.03 |  | 100 | 0 |
| 81 | 25.25 | 17.2 | 18.8 | 16.45 | 11.52 | 6.46 | 2.9 | 1.04 | 0.3 | 0.08 |  | 28.49 | 18.62 | 19.29 | 15.7 | 10.04 | 5.05 | 1.99 | 0.62 | 0.15 | 0.03 |  | 100 | 0 |
| 82 | 25.25 | 17.2 | 18.8 | 16.45 | 11.52 | 6.46 | 2.9 | 1.04 | 0.3 | 0.08 |  | 28.49 | 18.62 | 19.29 | 15.7 | 10.04 | 5.05 | 1.99 | 0.62 | 0.15 | 0.03 |  | 100 | 0 |
| 83 | 25.25 | 17.2 | 18.8 | 16.45 | 11.52 | 6.46 | 2.9 | 1.04 | 0.3 | 0.08 |  | 28.49 | 18.62 | 19.29 | 15.7 | 10.04 | 5.05 | 1.99 | 0.62 | 0.15 | 0.03 |  | 100 | 0 |
| 84 | 25.25 | 17.2 | 18.8 | 16.45 | 11.52 | 6.46 | 2.9 | 1.04 | 0.3 | 0.08 |  | 28.49 | 18.62 | 19.29 | 15.7 | 10.04 | 5.05 | 1.99 | 0.62 | 0.15 | 0.03 |  | 100 | 0 |
| 85 | 25.25 | 17.2 | 18.8 | 16.45 | 11.52 | 6.46 | 2.9 | 1.04 | 0.3 | 0.08 |  | 28.31 | 18.55 | 19.27 | 15.75 | 10.13 | 5.12 | 2.04 | 0.64 | 0.16 | 0.04 |  | 100 | 0 |
| 86 | 25.25 | 17.2 | 18.8 | 16.45 | 11.52 | 6.46 | 2.9 | 1.04 | 0.3 | 0.08 |  | 28.31 | 18.55 | 19.27 | 15.75 | 10.13 | 5.12 | 2.04 | 0.64 | 0.16 | 0.04 |  | 100 | 0 |
| 87 | 25.25 | 17.2 | 18.8 | 16.45 | 11.52 | 6.46 | 2.9 | 1.04 | 0.3 | 0.08 |  | 28.31 | 18.55 | 19.27 | 15.75 | 10.13 | 5.12 | 2.04 | 0.64 | 0.16 | 0.04 |  | 100 | 0 |
| 88 | 25.25 | 17.2 | 18.8 | 16.45 | 11.52 | 6.46 | 2.9 | 1.04 | 0.3 | 0.08 |  | 28.31 | 18.55 | 19.27 | 15.75 | 10.13 | 5.12 | 2.04 | 0.64 | 0.16 | 0.04 |  | 100 | 0 |
| 89 | 25.25 | 17.2 | 18.8 | 16.45 | 11.52 | 6.46 | 2.9 | 1.04 | 0.3 | 0.08 |  | 28.31 | 18.55 | 19.27 | 15.75 | 10.13 | 5.12 | 2.04 | 0.64 | 0.16 | 0.04 |  | 100 | 0 |
| 90 | 25.25 | 17.2 | 18.8 | 16.45 | 11.52 | 6.46 | 2.9 | 1.04 | 0.3 | 0.08 |  | 28.31 | 18.55 | 19.27 | 15.75 | 10.13 | 5.12 | 2.04 | 0.64 | 0.16 | 0.04 |  | 100 | 0 |
| 91 | 25.25 | 17.2 | 18.8 | 16.45 | 11.52 | 6.46 | 2.9 | 1.04 | 0.3 | 0.08 |  | 28.31 | 18.55 | 19.27 | 15.75 | 10.13 | 5.12 | 2.04 | 0.64 | 0.16 | 0.04 |  | 100 | 0 |
| 92 | 25.25 | 17.2 | 18.8 | 16.45 | 11.52 | 6.46 | 2.9 | 1.04 | 0.3 | 0.08 |  | 28.31 | 18.55 | 19.27 | 15.75 | 10.13 | 5.12 | 2.04 | 0.64 | 0.16 | 0.04 |  | 100 | 0 |
| 93 | 25.25 | 17.2 | 18.8 | 16.45 | 11.52 | 6.46 | 2.9 | 1.04 | 0.3 | 0.08 |  | 28.31 | 18.55 | 19.27 | 15.75 | 10.13 | 5.12 | 2.04 | 0.64 | 0.16 | 0.04 |  | 100 | 0 |
| 94 | 25.25 | 17.2 | 18.8 | 16.45 | 11.52 | 6.46 | 2.9 | 1.04 | 0.3 | 0.08 |  | 28.31 | 18.55 | 19.27 | 15.75 | 10.13 | 5.12 | 2.04 | 0.64 | 0.16 | 0.04 |  | 100 | 0 |
| 95 | 25.25 | 17.2 | 18.8 | 16.45 | 11.52 | 6.46 | 2.9 | 1.04 | 0.3 | 0.08 |  | 28.31 | 18.55 | 19.27 | 15.75 | 10.13 | 5.12 | 2.04 | 0.64 | 0.16 | 0.04 |  | 100 | 0 |
|  | |  | Females | | | | | | | | | | | | | | | | | | | | | |
| 0 | 100 | 0 | 0 | 0 | 0 | 0 | 0 | 0 | 0 | 0 |  | 100 | 0 | 0 | 0 | 0 | 0 | 0 | 0 | 0 | 0 |  | 100 | 0 |
| 1 | 100 | 0 | 0 | 0 | 0 | 0 | 0 | 0 | 0 | 0 |  | 100 | 0 | 0 | 0 | 0 | 0 | 0 | 0 | 0 | 0 |  | 100 | 0 |
| 2 | 100 | 0 | 0 | 0 | 0 | 0 | 0 | 0 | 0 | 0 |  | 100 | 0 | 0 | 0 | 0 | 0 | 0 | 0 | 0 | 0 |  | 100 | 0 |
| 3 | 100 | 0 | 0 | 0 | 0 | 0 | 0 | 0 | 0 | 0 |  | 100 | 0 | 0 | 0 | 0 | 0 | 0 | 0 | 0 | 0 |  | 100 | 0 |
| 4 | 100 | 0 | 0 | 0 | 0 | 0 | 0 | 0 | 0 | 0 |  | 100 | 0 | 0 | 0 | 0 | 0 | 0 | 0 | 0 | 0 |  | 100 | 0 |
| 5 | 100 | 0 | 0 | 0 | 0 | 0 | 0 | 0 | 0 | 0 |  | 100 | 0 | 0 | 0 | 0 | 0 | 0 | 0 | 0 | 0 |  | 100 | 0 |
| 6 | 28.56 | 22.23 | 21.98 | 15.57 | 7.89 | 2.86 | 0.74 | 0.14 | 0.02 | 0 |  | 28.56 | 22.23 | 21.98 | 15.57 | 7.89 | 2.86 | 0.74 | 0.14 | 0.02 | 0 |  | 100 | 0 |
| 7 | 29.13 | 21.87 | 21.56 | 15.42 | 8 | 3.01 | 0.82 | 0.16 | 0.02 | 0 |  | 29.13 | 21.87 | 21.56 | 15.42 | 8 | 3.01 | 0.82 | 0.16 | 0.02 | 0 |  | 100 | 0 |
| 8 | 29.54 | 21.52 | 21.21 | 15.33 | 8.13 | 3.16 | 0.9 | 0.19 | 0.03 | 0 |  | 29.54 | 21.52 | 21.21 | 15.33 | 8.13 | 3.16 | 0.9 | 0.19 | 0.03 | 0 |  | 100 | 0 |
| 9 | 29.77 | 21.2 | 20.93 | 15.29 | 8.27 | 3.31 | 0.98 | 0.22 | 0.03 | 0 |  | 29.77 | 21.2 | 20.93 | 15.29 | 8.27 | 3.31 | 0.98 | 0.22 | 0.03 | 0 |  | 100 | 0 |
| 10 | 29.91 | 20.9 | 20.67 | 15.27 | 8.42 | 3.47 | 1.06 | 0.24 | 0.04 | 0.01 |  | 29.91 | 20.9 | 20.67 | 15.27 | 8.42 | 3.47 | 1.06 | 0.24 | 0.04 | 0.01 |  | 100 | 0 |
| 11 | 30.09 | 20.61 | 20.42 | 15.23 | 8.55 | 3.61 | 1.15 | 0.27 | 0.05 | 0.01 |  | 30.09 | 20.61 | 20.42 | 15.23 | 8.55 | 3.61 | 1.15 | 0.27 | 0.05 | 0.01 |  | 100 | 0 |
| 12 | 30.42 | 20.32 | 20.12 | 15.14 | 8.64 | 3.75 | 1.23 | 0.31 | 0.06 | 0.01 |  | 30.42 | 20.32 | 20.12 | 15.14 | 8.64 | 3.75 | 1.23 | 0.31 | 0.06 | 0.01 |  | 100 | 0 |
| 13 | 30.94 | 20 | 19.76 | 14.97 | 8.7 | 3.88 | 1.32 | 0.35 | 0.07 | 0.01 |  | 30.94 | 20 | 19.76 | 14.97 | 8.7 | 3.88 | 1.32 | 0.35 | 0.07 | 0.01 |  | 100 | 0 |
| 14 | 31.65 | 19.66 | 19.34 | 14.74 | 8.71 | 3.99 | 1.42 | 0.39 | 0.08 | 0.02 |  | 31.65 | 19.66 | 19.34 | 14.74 | 8.71 | 3.99 | 1.42 | 0.39 | 0.08 | 0.02 |  | 100 | 0 |
| 15 | 32.43 | 19.32 | 18.91 | 14.49 | 8.7 | 4.09 | 1.5 | 0.43 | 0.1 | 0.02 |  | 35.93 | 20.36 | 18.79 | 13.33 | 7.27 | 3.05 | 0.98 | 0.24 | 0.05 | 0.01 |  | 100 | 0 |
| 16 | 33.12 | 19.05 | 18.56 | 14.27 | 8.67 | 4.16 | 1.57 | 0.47 | 0.11 | 0.02 |  | 36.6 | 20.04 | 18.44 | 13.15 | 7.28 | 3.12 | 1.04 | 0.27 | 0.05 | 0.01 |  | 100 | 0 |
| 17 | 33.62 | 18.88 | 18.32 | 14.12 | 8.63 | 4.19 | 1.61 | 0.49 | 0.12 | 0.03 |  | 37.08 | 19.84 | 18.21 | 13.02 | 7.26 | 3.16 | 1.07 | 0.28 | 0.06 | 0.01 |  | 100 | 0 |
| 18 | 33.92 | 18.8 | 18.2 | 14.02 | 8.59 | 4.19 | 1.62 | 0.5 | 0.12 | 0.03 |  | 37.39 | 19.75 | 18.08 | 12.94 | 7.24 | 3.16 | 1.08 | 0.29 | 0.06 | 0.01 |  | 100 | 0 |
| 19 | 34.07 | 18.8 | 18.17 | 13.98 | 8.55 | 4.17 | 1.62 | 0.5 | 0.12 | 0.03 |  | 37.72 | 19.78 | 18.02 | 12.83 | 7.14 | 3.1 | 1.05 | 0.28 | 0.06 | 0.01 |  | 100 | 0 |
| 20 | 34.12 | 18.83 | 18.18 | 13.96 | 8.53 | 4.14 | 1.6 | 0.49 | 0.12 | 0.03 |  | 37.77 | 19.81 | 18.03 | 12.81 | 7.11 | 3.08 | 1.04 | 0.27 | 0.06 | 0.01 |  | 100 | 0 |
| 21 | 34.11 | 18.88 | 18.22 | 13.97 | 8.51 | 4.12 | 1.58 | 0.48 | 0.12 | 0.03 |  | 37.77 | 19.86 | 18.06 | 12.81 | 7.08 | 3.06 | 1.03 | 0.27 | 0.06 | 0.01 |  | 100 | 0 |
| 22 | 34.07 | 18.92 | 18.26 | 13.98 | 8.5 | 4.1 | 1.57 | 0.48 | 0.12 | 0.03 |  | 37.74 | 19.91 | 18.1 | 12.81 | 7.07 | 3.03 | 1.01 | 0.26 | 0.05 | 0.01 |  | 100 | 0 |
| 23 | 34.02 | 18.95 | 18.29 | 13.99 | 8.49 | 4.08 | 1.56 | 0.47 | 0.11 | 0.02 |  | 37.7 | 19.95 | 18.13 | 12.82 | 7.06 | 3.02 | 1.01 | 0.26 | 0.05 | 0.01 |  | 100 | 0 |
| 24 | 33.98 | 18.98 | 18.32 | 14.01 | 8.49 | 4.07 | 1.55 | 0.47 | 0.11 | 0.02 |  | 37.66 | 19.97 | 18.15 | 12.83 | 7.05 | 3.01 | 1 | 0.26 | 0.05 | 0.01 |  | 100 | 0 |
| 25 | 33.95 | 18.99 | 18.34 | 14.02 | 8.49 | 4.07 | 1.54 | 0.46 | 0.11 | 0.02 |  | 37.74 | 20.02 | 18.16 | 12.8 | 7 | 2.98 | 0.98 | 0.25 | 0.05 | 0.01 |  | 100 | 0 |
| 26 | 33.93 | 19 | 18.34 | 14.02 | 8.49 | 4.07 | 1.54 | 0.46 | 0.11 | 0.02 |  | 37.73 | 20.03 | 18.17 | 12.8 | 7 | 2.98 | 0.98 | 0.25 | 0.05 | 0.01 |  | 100 | 0 |
| 27 | 33.92 | 19 | 18.35 | 14.03 | 8.49 | 4.07 | 1.54 | 0.46 | 0.11 | 0.02 |  | 37.71 | 20.03 | 18.18 | 12.81 | 7.01 | 2.98 | 0.98 | 0.25 | 0.05 | 0.01 |  | 100 | 0 |
| 28 | 33.91 | 19 | 18.35 | 14.03 | 8.49 | 4.07 | 1.54 | 0.46 | 0.11 | 0.02 |  | 37.71 | 20.03 | 18.18 | 12.81 | 7.01 | 2.98 | 0.98 | 0.25 | 0.05 | 0.01 |  | 100 | 0 |
| 29 | 33.91 | 19 | 18.35 | 14.03 | 8.49 | 4.07 | 1.54 | 0.46 | 0.11 | 0.02 |  | 37.71 | 20.03 | 18.18 | 12.81 | 7.01 | 2.98 | 0.98 | 0.25 | 0.05 | 0.01 |  | 100 | 0 |
| 30 | 33.91 | 19 | 18.35 | 14.03 | 8.49 | 4.07 | 1.54 | 0.46 | 0.11 | 0.02 |  | 37.94 | 20.08 | 18.15 | 12.73 | 6.92 | 2.92 | 0.96 | 0.24 | 0.05 | 0.01 |  | 100 | 0 |
| 31 | 33.91 | 19 | 18.35 | 14.03 | 8.49 | 4.07 | 1.55 | 0.46 | 0.11 | 0.02 |  | 37.94 | 20.08 | 18.15 | 12.73 | 6.92 | 2.92 | 0.96 | 0.24 | 0.05 | 0.01 |  | 100 | 0 |
| 32 | 33.92 | 19 | 18.34 | 14.03 | 8.49 | 4.07 | 1.55 | 0.46 | 0.11 | 0.02 |  | 37.94 | 20.08 | 18.15 | 12.73 | 6.92 | 2.92 | 0.96 | 0.24 | 0.05 | 0.01 |  | 100 | 0 |
| 33 | 33.92 | 19 | 18.34 | 14.03 | 8.49 | 4.07 | 1.55 | 0.46 | 0.11 | 0.02 |  | 37.95 | 20.08 | 18.15 | 12.73 | 6.92 | 2.92 | 0.96 | 0.24 | 0.05 | 0.01 |  | 100 | 0 |
| 34 | 33.92 | 19 | 18.34 | 14.03 | 8.49 | 4.07 | 1.55 | 0.46 | 0.11 | 0.02 |  | 37.95 | 20.08 | 18.15 | 12.73 | 6.92 | 2.92 | 0.96 | 0.24 | 0.05 | 0.01 |  | 100 | 0 |
| 35 | 33.92 | 19 | 18.34 | 14.03 | 8.49 | 4.07 | 1.55 | 0.46 | 0.11 | 0.02 |  | 37.95 | 20.08 | 18.15 | 12.73 | 6.92 | 2.92 | 0.96 | 0.24 | 0.05 | 0.01 |  | 100 | 0 |
| 36 | 33.92 | 19 | 18.34 | 14.03 | 8.49 | 4.07 | 1.55 | 0.46 | 0.11 | 0.02 |  | 37.95 | 20.08 | 18.15 | 12.73 | 6.92 | 2.92 | 0.96 | 0.24 | 0.05 | 0.01 |  | 100 | 0 |
| 37 | 33.92 | 19 | 18.34 | 14.03 | 8.49 | 4.07 | 1.55 | 0.46 | 0.11 | 0.02 |  | 37.95 | 20.08 | 18.15 | 12.73 | 6.92 | 2.92 | 0.96 | 0.24 | 0.05 | 0.01 |  | 100 | 0 |
| 38 | 33.92 | 19 | 18.34 | 14.03 | 8.49 | 4.07 | 1.55 | 0.46 | 0.11 | 0.02 |  | 37.95 | 20.08 | 18.15 | 12.73 | 6.92 | 2.92 | 0.96 | 0.24 | 0.05 | 0.01 |  | 100 | 0 |
| 39 | 33.92 | 19 | 18.34 | 14.03 | 8.49 | 4.07 | 1.55 | 0.46 | 0.11 | 0.02 |  | 37.95 | 20.08 | 18.15 | 12.73 | 6.92 | 2.92 | 0.96 | 0.24 | 0.05 | 0.01 |  | 100 | 0 |
| 40 | 33.92 | 19 | 18.34 | 14.03 | 8.49 | 4.07 | 1.55 | 0.46 | 0.11 | 0.02 |  | 38.18 | 20.13 | 18.13 | 12.65 | 6.84 | 2.86 | 0.93 | 0.23 | 0.04 | 0.01 |  | 100 | 0 |
| 41 | 33.92 | 19 | 18.34 | 14.03 | 8.49 | 4.07 | 1.55 | 0.46 | 0.11 | 0.02 |  | 38.18 | 20.13 | 18.13 | 12.65 | 6.84 | 2.86 | 0.93 | 0.23 | 0.04 | 0.01 |  | 100 | 0 |
| 42 | 33.92 | 19 | 18.34 | 14.03 | 8.49 | 4.07 | 1.55 | 0.46 | 0.11 | 0.02 |  | 38.18 | 20.13 | 18.13 | 12.65 | 6.84 | 2.86 | 0.93 | 0.23 | 0.04 | 0.01 |  | 100 | 0 |
| 43 | 33.92 | 19 | 18.34 | 14.03 | 8.49 | 4.07 | 1.55 | 0.46 | 0.11 | 0.02 |  | 38.18 | 20.13 | 18.13 | 12.65 | 6.84 | 2.86 | 0.93 | 0.23 | 0.04 | 0.01 |  | 100 | 0 |
| 44 | 33.92 | 19 | 18.34 | 14.03 | 8.49 | 4.07 | 1.55 | 0.46 | 0.11 | 0.02 |  | 38.18 | 20.13 | 18.13 | 12.65 | 6.84 | 2.86 | 0.93 | 0.23 | 0.04 | 0.01 |  | 100 | 0 |
| 45 | 33.92 | 19 | 18.34 | 14.03 | 8.49 | 4.07 | 1.55 | 0.46 | 0.11 | 0.02 |  | 38.18 | 20.13 | 18.13 | 12.65 | 6.84 | 2.86 | 0.93 | 0.23 | 0.04 | 0.01 |  | 100 | 0 |
| 46 | 33.91 | 19 | 18.34 | 14.03 | 8.5 | 4.07 | 1.55 | 0.46 | 0.11 | 0.02 |  | 38.17 | 20.13 | 18.13 | 12.65 | 6.84 | 2.86 | 0.93 | 0.23 | 0.04 | 0.01 |  | 100 | 0 |
| 47 | 33.91 | 19 | 18.34 | 14.03 | 8.5 | 4.08 | 1.55 | 0.46 | 0.11 | 0.02 |  | 38.17 | 20.13 | 18.13 | 12.65 | 6.84 | 2.86 | 0.93 | 0.23 | 0.04 | 0.01 |  | 100 | 0 |
| 48 | 33.91 | 19 | 18.34 | 14.03 | 8.5 | 4.08 | 1.55 | 0.46 | 0.11 | 0.02 |  | 38.17 | 20.13 | 18.13 | 12.65 | 6.84 | 2.86 | 0.93 | 0.23 | 0.04 | 0.01 |  | 100 | 0 |
| 49 | 33.91 | 19 | 18.34 | 14.03 | 8.5 | 4.08 | 1.55 | 0.46 | 0.11 | 0.02 |  | 38.17 | 20.13 | 18.13 | 12.65 | 6.84 | 2.86 | 0.93 | 0.23 | 0.04 | 0.01 |  | 100 | 0 |
| 50 | 33.91 | 19 | 18.34 | 14.03 | 8.5 | 4.08 | 1.55 | 0.46 | 0.11 | 0.02 |  | 38.28 | 20.16 | 18.12 | 12.61 | 6.8 | 2.84 | 0.92 | 0.23 | 0.04 | 0.01 |  | 100 | 0 |
| 51 | 33.92 | 19 | 18.34 | 14.03 | 8.49 | 4.07 | 1.55 | 0.46 | 0.11 | 0.02 |  | 38.29 | 20.16 | 18.12 | 12.61 | 6.8 | 2.84 | 0.92 | 0.23 | 0.04 | 0.01 |  | 100 | 0 |
| 52 | 33.93 | 19 | 18.34 | 14.02 | 8.49 | 4.07 | 1.55 | 0.46 | 0.11 | 0.02 |  | 38.31 | 20.16 | 18.11 | 12.6 | 6.79 | 2.83 | 0.92 | 0.23 | 0.04 | 0.01 |  | 100 | 0 |
| 53 | 33.96 | 19 | 18.34 | 14.02 | 8.48 | 4.07 | 1.54 | 0.46 | 0.11 | 0.02 |  | 38.34 | 20.16 | 18.11 | 12.6 | 6.78 | 2.83 | 0.91 | 0.23 | 0.04 | 0.01 |  | 100 | 0 |
| 54 | 34 | 19 | 18.33 | 14 | 8.47 | 4.06 | 1.54 | 0.46 | 0.11 | 0.02 |  | 38.38 | 20.16 | 18.09 | 12.58 | 6.77 | 2.82 | 0.91 | 0.23 | 0.04 | 0.01 |  | 100 | 0 |
| 55 | 34.05 | 19 | 18.32 | 13.99 | 8.46 | 4.05 | 1.54 | 0.46 | 0.11 | 0.02 |  | 38.56 | 20.18 | 18.07 | 12.52 | 6.71 | 2.79 | 0.9 | 0.22 | 0.04 | 0.01 |  | 100 | 0 |
| 56 | 34.12 | 19 | 18.31 | 13.97 | 8.44 | 4.04 | 1.53 | 0.46 | 0.11 | 0.02 |  | 38.63 | 20.18 | 18.05 | 12.5 | 6.7 | 2.78 | 0.89 | 0.22 | 0.04 | 0.01 |  | 100 | 0 |
| 57 | 34.18 | 19.01 | 18.3 | 13.95 | 8.42 | 4.03 | 1.53 | 0.46 | 0.11 | 0.02 |  | 38.69 | 20.18 | 18.04 | 12.48 | 6.68 | 2.77 | 0.89 | 0.22 | 0.04 | 0.01 |  | 100 | 0 |
| 58 | 34.2 | 19.01 | 18.29 | 13.94 | 8.42 | 4.03 | 1.52 | 0.46 | 0.11 | 0.02 |  | 38.72 | 20.18 | 18.03 | 12.47 | 6.68 | 2.77 | 0.89 | 0.22 | 0.04 | 0.01 |  | 100 | 0 |
| 59 | 34.15 | 19.01 | 18.3 | 13.96 | 8.43 | 4.03 | 1.53 | 0.46 | 0.11 | 0.02 |  | 38.66 | 20.18 | 18.04 | 12.49 | 6.69 | 2.77 | 0.89 | 0.22 | 0.04 | 0.01 |  | 100 | 0 |
| 60 | 33.96 | 19 | 18.34 | 14.01 | 8.48 | 4.07 | 1.54 | 0.46 | 0.11 | 0.02 |  | 38.58 | 20.21 | 18.08 | 12.51 | 6.7 | 2.77 | 0.89 | 0.22 | 0.04 | 0.01 |  | 100 | 0 |
| 61 | 33.56 | 18.98 | 18.41 | 14.13 | 8.59 | 4.13 | 1.57 | 0.48 | 0.11 | 0.02 |  | 38.15 | 20.21 | 18.18 | 12.65 | 6.8 | 2.83 | 0.91 | 0.23 | 0.04 | 0.01 |  | 100 | 0 |
| 62 | 32.88 | 18.94 | 18.53 | 14.34 | 8.78 | 4.25 | 1.63 | 0.49 | 0.12 | 0.03 |  | 37.4 | 20.22 | 18.36 | 12.88 | 6.98 | 2.93 | 0.95 | 0.24 | 0.05 | 0.01 |  | 100 | 0 |
| 63 | 31.85 | 18.88 | 18.71 | 14.66 | 9.07 | 4.44 | 1.71 | 0.52 | 0.13 | 0.03 |  | 36.29 | 20.21 | 18.61 | 13.23 | 7.26 | 3.08 | 1 | 0.25 | 0.05 | 0.01 |  | 100 | 0 |
| 64 | 30.54 | 18.78 | 18.93 | 15.06 | 9.45 | 4.68 | 1.83 | 0.56 | 0.14 | 0.03 |  | 34.87 | 20.18 | 18.93 | 13.69 | 7.63 | 3.28 | 1.08 | 0.28 | 0.05 | 0.01 |  | 100 | 0 |
| 65 | 29.08 | 18.65 | 19.17 | 15.51 | 9.88 | 4.96 | 1.96 | 0.61 | 0.15 | 0.03 |  | 33.42 | 20.18 | 19.27 | 14.15 | 7.99 | 3.47 | 1.16 | 0.3 | 0.06 | 0.01 |  | 100 | 0 |
| 66 | 27.79 | 18.51 | 19.36 | 15.92 | 10.28 | 5.21 | 2.08 | 0.65 | 0.16 | 0.04 |  | 32.01 | 20.11 | 19.57 | 14.62 | 8.38 | 3.68 | 1.24 | 0.32 | 0.06 | 0.01 |  | 100 | 0 |
| 67 | 26.79 | 18.39 | 19.51 | 16.23 | 10.59 | 5.42 | 2.18 | 0.68 | 0.17 | 0.04 |  | 30.92 | 20.04 | 19.8 | 14.98 | 8.68 | 3.85 | 1.31 | 0.34 | 0.07 | 0.01 |  | 100 | 0 |
| 68 | 26.13 | 18.3 | 19.6 | 16.44 | 10.8 | 5.56 | 2.24 | 0.71 | 0.18 | 0.04 |  | 30.19 | 19.99 | 19.94 | 15.22 | 8.89 | 3.97 | 1.36 | 0.35 | 0.07 | 0.01 |  | 100 | 0 |
| 69 | 25.75 | 18.25 | 19.65 | 16.56 | 10.93 | 5.64 | 2.28 | 0.72 | 0.18 | 0.04 |  | 29.77 | 19.96 | 20.03 | 15.36 | 9.01 | 4.04 | 1.38 | 0.36 | 0.07 | 0.01 |  | 100 | 0 |
| 70 | 25.56 | 18.22 | 19.67 | 16.62 | 10.99 | 5.68 | 2.3 | 0.73 | 0.18 | 0.04 |  | 29.47 | 19.9 | 20.06 | 15.47 | 9.12 | 4.11 | 1.42 | 0.37 | 0.08 | 0.01 |  | 100 | 0 |
| 71 | 25.52 | 18.21 | 19.68 | 16.64 | 11 | 5.69 | 2.31 | 0.73 | 0.18 | 0.04 |  | 29.42 | 19.89 | 20.07 | 15.48 | 9.13 | 4.12 | 1.42 | 0.37 | 0.08 | 0.01 |  | 100 | 0 |
| 72 | 25.54 | 18.22 | 19.68 | 16.63 | 11 | 5.69 | 2.3 | 0.73 | 0.18 | 0.04 |  | 29.44 | 19.9 | 20.07 | 15.47 | 9.13 | 4.12 | 1.42 | 0.37 | 0.08 | 0.01 |  | 100 | 0 |
| 73 | 25.6 | 18.23 | 19.67 | 16.61 | 10.98 | 5.68 | 2.3 | 0.73 | 0.18 | 0.04 |  | 29.5 | 19.9 | 20.05 | 15.45 | 9.11 | 4.1 | 1.41 | 0.37 | 0.08 | 0.01 |  | 100 | 0 |
| 74 | 25.66 | 18.24 | 19.66 | 16.59 | 10.96 | 5.66 | 2.29 | 0.73 | 0.18 | 0.04 |  | 29.57 | 19.91 | 20.04 | 15.43 | 9.09 | 4.09 | 1.41 | 0.37 | 0.08 | 0.01 |  | 100 | 0 |
| 75 | 25.71 | 18.24 | 19.66 | 16.57 | 10.94 | 5.65 | 2.28 | 0.72 | 0.18 | 0.04 |  | 29.62 | 19.91 | 20.03 | 15.41 | 9.07 | 4.08 | 1.41 | 0.37 | 0.08 | 0.01 |  | 100 | 0 |
| 76 | 25.75 | 18.25 | 19.65 | 16.56 | 10.93 | 5.64 | 2.28 | 0.72 | 0.18 | 0.04 |  | 29.67 | 19.91 | 20.02 | 15.4 | 9.06 | 4.08 | 1.4 | 0.37 | 0.07 | 0.01 |  | 100 | 0 |
| 77 | 25.77 | 18.25 | 19.65 | 16.55 | 10.92 | 5.64 | 2.28 | 0.72 | 0.18 | 0.04 |  | 29.7 | 19.92 | 20.02 | 15.39 | 9.05 | 4.07 | 1.4 | 0.37 | 0.07 | 0.01 |  | 100 | 0 |
| 78 | 25.79 | 18.25 | 19.64 | 16.55 | 10.91 | 5.63 | 2.28 | 0.72 | 0.18 | 0.04 |  | 29.71 | 19.92 | 20.01 | 15.38 | 9.05 | 4.07 | 1.4 | 0.37 | 0.07 | 0.01 |  | 100 | 0 |
| 79 | 25.8 | 18.25 | 19.64 | 16.55 | 10.91 | 5.63 | 2.28 | 0.72 | 0.18 | 0.04 |  | 29.72 | 19.92 | 20.01 | 15.38 | 9.05 | 4.07 | 1.4 | 0.37 | 0.07 | 0.01 |  | 100 | 0 |
| 80 | 25.8 | 18.25 | 19.64 | 16.55 | 10.91 | 5.63 | 2.28 | 0.72 | 0.18 | 0.04 |  | 29.51 | 19.84 | 20 | 15.45 | 9.14 | 4.14 | 1.44 | 0.38 | 0.08 | 0.01 |  | 100 | 0 |
| 81 | 25.8 | 18.25 | 19.64 | 16.55 | 10.91 | 5.63 | 2.28 | 0.72 | 0.18 | 0.04 |  | 29.51 | 19.84 | 20 | 15.45 | 9.14 | 4.14 | 1.44 | 0.38 | 0.08 | 0.01 |  | 100 | 0 |
| 82 | 25.79 | 18.25 | 19.64 | 16.55 | 10.91 | 5.63 | 2.28 | 0.72 | 0.18 | 0.04 |  | 29.5 | 19.84 | 20 | 15.45 | 9.14 | 4.15 | 1.44 | 0.38 | 0.08 | 0.01 |  | 100 | 0 |
| 83 | 25.79 | 18.25 | 19.64 | 16.55 | 10.91 | 5.63 | 2.28 | 0.72 | 0.18 | 0.04 |  | 29.5 | 19.84 | 20 | 15.46 | 9.14 | 4.15 | 1.44 | 0.38 | 0.08 | 0.01 |  | 100 | 0 |
| 84 | 25.79 | 18.25 | 19.64 | 16.55 | 10.91 | 5.63 | 2.28 | 0.72 | 0.18 | 0.04 |  | 29.5 | 19.84 | 20.01 | 15.46 | 9.15 | 4.15 | 1.44 | 0.38 | 0.08 | 0.01 |  | 100 | 0 |
| 85 | 25.79 | 18.25 | 19.65 | 16.55 | 10.91 | 5.63 | 2.28 | 0.72 | 0.18 | 0.04 |  | 29.39 | 19.79 | 20 | 15.49 | 9.2 | 4.18 | 1.46 | 0.39 | 0.08 | 0.01 |  | 100 | 0 |
| 86 | 25.79 | 18.25 | 19.65 | 16.55 | 10.91 | 5.63 | 2.28 | 0.72 | 0.18 | 0.04 |  | 29.39 | 19.79 | 20 | 15.49 | 9.2 | 4.18 | 1.46 | 0.39 | 0.08 | 0.01 |  | 100 | 0 |
| 87 | 25.78 | 18.25 | 19.65 | 16.55 | 10.92 | 5.63 | 2.28 | 0.72 | 0.18 | 0.04 |  | 29.39 | 19.79 | 20 | 15.49 | 9.2 | 4.18 | 1.46 | 0.39 | 0.08 | 0.01 |  | 100 | 0 |
| 88 | 25.78 | 18.25 | 19.65 | 16.55 | 10.92 | 5.63 | 2.28 | 0.72 | 0.18 | 0.04 |  | 29.39 | 19.79 | 20 | 15.49 | 9.2 | 4.18 | 1.46 | 0.39 | 0.08 | 0.01 |  | 100 | 0 |
| 89 | 25.78 | 18.25 | 19.65 | 16.55 | 10.92 | 5.63 | 2.28 | 0.72 | 0.18 | 0.04 |  | 29.39 | 19.79 | 20 | 15.49 | 9.2 | 4.18 | 1.46 | 0.39 | 0.08 | 0.01 |  | 100 | 0 |
| 90 | 25.78 | 18.25 | 19.65 | 16.55 | 10.92 | 5.63 | 2.28 | 0.72 | 0.18 | 0.04 |  | 29.39 | 19.79 | 20 | 15.49 | 9.2 | 4.18 | 1.46 | 0.39 | 0.08 | 0.01 |  | 100 | 0 |
| 91 | 25.78 | 18.25 | 19.65 | 16.55 | 10.92 | 5.63 | 2.28 | 0.72 | 0.18 | 0.04 |  | 29.39 | 19.79 | 20 | 15.49 | 9.2 | 4.18 | 1.46 | 0.39 | 0.08 | 0.01 |  | 100 | 0 |
| 92 | 25.78 | 18.25 | 19.65 | 16.55 | 10.92 | 5.63 | 2.28 | 0.72 | 0.18 | 0.04 |  | 29.39 | 19.79 | 20 | 15.49 | 9.2 | 4.18 | 1.46 | 0.39 | 0.08 | 0.01 |  | 100 | 0 |
| 93 | 25.79 | 18.25 | 19.65 | 16.55 | 10.91 | 5.63 | 2.28 | 0.72 | 0.18 | 0.04 |  | 29.39 | 19.79 | 20 | 15.49 | 9.2 | 4.18 | 1.46 | 0.39 | 0.08 | 0.01 |  | 100 | 0 |
| 94 | 25.79 | 18.25 | 19.65 | 16.55 | 10.91 | 5.63 | 2.28 | 0.72 | 0.18 | 0.04 |  | 29.39 | 19.79 | 20 | 15.49 | 9.2 | 4.18 | 1.46 | 0.39 | 0.08 | 0.01 |  | 100 | 0 |
| 95 | 25.79 | 18.25 | 19.65 | 16.55 | 10.91 | 5.63 | 2.28 | 0.72 | 0.18 | 0.04 |  | 29.39 | 19.79 | 20 | 15.49 | 9.2 | 4.18 | 1.46 | 0.39 | 0.08 | 0.01 |  | 100 | 0 |

^a^ Rounded to two decimal points

^b^ %E = Percent of total energy intake
