# Supplementary material for: Substituting polyunsaturated fat for saturated fat: A health impact assessment of a fat tax in seven European countries
Source: PLoS One. 2019 Jul 10;14(7):e0218464. doi: 10.1371/journal.pone.0218464 (PMC6619676; doi:10.1371/journal.pone.0218464)
Supplement: S14 Table — (DOCX) [file pone.0218464.s014.docx]

# S14 Table. Proportion of persons in the respective saturated fat intake categories across scenarios in Spain.

| Age | Reference scenario^a^ | | | | | | | | | |  | Fat tax scenario^a^ | | | | | | | | | |  | Guideline scenario | |
| --- | --- | --- | --- | --- | --- | --- | --- | --- | --- | --- | --- | --- | --- | --- | --- | --- | --- | --- | --- | --- | --- | --- | --- | --- |
|  | Category of saturated fat intake (in %E)^b^ | | | | | | | | | |  | Category of saturated fat intake (in %E)^b^ | | | | | | | | | |  | Category of saturated fat intake (in %E)^b^ | |
|  | ≤10 | >10 ≤12 | >12 ≤14 | >14 ≤16 | >16 ≤18 | >18 ≤20 | >20 ≤22 | >22 ≤24 | >24 ≤26 | >26 ≤100 |  | ≤10 | >10 ≤12 | >12 ≤14 | >14 ≤16 | >16 ≤18 | >18 ≤20 | >20 ≤22 | >22 ≤24 | >24 ≤26 | >26 ≤100 |  | ≤10 | >10 ≤100 |
|  |  |  | Males | | | | | | | | | | | | | | | | | | | | | |
| 0 | 100 | 0 | 0 | 0 | 0 | 0 | 0 | 0 | 0 | 0 |  | 100 | 0 | 0 | 0 | 0 | 0 | 0 | 0 | 0 | 0 |  | 100 | 0 |
| 1 | 100 | 0 | 0 | 0 | 0 | 0 | 0 | 0 | 0 | 0 |  | 100 | 0 | 0 | 0 | 0 | 0 | 0 | 0 | 0 | 0 |  | 100 | 0 |
| 2 | 100 | 0 | 0 | 0 | 0 | 0 | 0 | 0 | 0 | 0 |  | 100 | 0 | 0 | 0 | 0 | 0 | 0 | 0 | 0 | 0 |  | 100 | 0 |
| 3 | 100 | 0 | 0 | 0 | 0 | 0 | 0 | 0 | 0 | 0 |  | 100 | 0 | 0 | 0 | 0 | 0 | 0 | 0 | 0 | 0 |  | 100 | 0 |
| 4 | 100 | 0 | 0 | 0 | 0 | 0 | 0 | 0 | 0 | 0 |  | 100 | 0 | 0 | 0 | 0 | 0 | 0 | 0 | 0 | 0 |  | 100 | 0 |
| 5 | 100 | 0 | 0 | 0 | 0 | 0 | 0 | 0 | 0 | 0 |  | 100 | 0 | 0 | 0 | 0 | 0 | 0 | 0 | 0 | 0 |  | 100 | 0 |
| 6 | 0.53 | 6.94 | 29.64 | 41.22 | 18.77 | 2.77 | 0.13 | 0 | 0 | 0 |  | 0.53 | 6.94 | 29.64 | 41.22 | 18.77 | 2.77 | 0.13 | 0 | 0 | 0 |  | 100 | 0 |
| 7 | 0.57 | 7.27 | 30.22 | 40.98 | 18.21 | 2.62 | 0.12 | 0 | 0 | 0 |  | 0.57 | 7.27 | 30.22 | 40.98 | 18.21 | 2.62 | 0.12 | 0 | 0 | 0 |  | 100 | 0 |
| 8 | 0.62 | 7.61 | 30.8 | 40.72 | 17.65 | 2.48 | 0.11 | 0 | 0 | 0 |  | 0.62 | 7.61 | 30.8 | 40.72 | 17.65 | 2.48 | 0.11 | 0 | 0 | 0 |  | 100 | 0 |
| 9 | 0.67 | 7.97 | 31.38 | 40.43 | 17.1 | 2.35 | 0.1 | 0 | 0 | 0 |  | 0.67 | 7.97 | 31.38 | 40.43 | 17.1 | 2.35 | 0.1 | 0 | 0 | 0 |  | 100 | 0 |
| 10 | 0.72 | 8.33 | 31.94 | 40.13 | 16.56 | 2.22 | 0.1 | 0 | 0 | 0 |  | 0.72 | 8.33 | 31.94 | 40.13 | 16.56 | 2.22 | 0.1 | 0 | 0 | 0 |  | 100 | 0 |
| 11 | 0.77 | 8.71 | 32.52 | 39.8 | 16.02 | 2.09 | 0.09 | 0 | 0 | 0 |  | 0.77 | 8.71 | 32.52 | 39.8 | 16.02 | 2.09 | 0.09 | 0 | 0 | 0 |  | 100 | 0 |
| 12 | 0.84 | 9.19 | 33.2 | 39.37 | 15.36 | 1.95 | 0.08 | 0 | 0 | 0 |  | 0.84 | 9.19 | 33.2 | 39.37 | 15.36 | 1.95 | 0.08 | 0 | 0 | 0 |  | 100 | 0 |
| 13 | 0.95 | 9.89 | 34.16 | 38.73 | 14.45 | 1.75 | 0.07 | 0 | 0 | 0 |  | 0.95 | 9.89 | 34.16 | 38.73 | 14.45 | 1.75 | 0.07 | 0 | 0 | 0 |  | 100 | 0 |
| 14 | 1.13 | 10.96 | 35.5 | 37.72 | 13.16 | 1.49 | 0.05 | 0 | 0 | 0 |  | 1.13 | 10.96 | 35.5 | 37.72 | 13.16 | 1.49 | 0.05 | 0 | 0 | 0 |  | 100 | 0 |
| 15 | 1.41 | 12.53 | 37.23 | 36.15 | 11.47 | 1.17 | 0.04 | 0 | 0 | 0 |  | 2.77 | 19.99 | 43.58 | 28.07 | 5.3 | 0.29 | 0 | 0 | 0 | 0 |  | 100 | 0 |
| 16 | 1.83 | 14.69 | 39.19 | 33.92 | 9.5 | 0.85 | 0.02 | 0 | 0 | 0 |  | 3.56 | 22.87 | 44.25 | 25.04 | 4.1 | 0.19 | 0 | 0 | 0 | 0 |  | 100 | 0 |
| 17 | 2.48 | 17.57 | 41.15 | 30.86 | 7.38 | 0.55 | 0.01 | 0 | 0 | 0 |  | 4.74 | 26.55 | 44.37 | 21.33 | 2.91 | 0.11 | 0 | 0 | 0 | 0 |  | 100 | 0 |
| 18 | 3.46 | 21.29 | 42.7 | 26.93 | 5.3 | 0.32 | 0.01 | 0 | 0 | 0 |  | 6.5 | 30.99 | 43.47 | 17.13 | 1.86 | 0.05 | 0 | 0 | 0 | 0 |  | 100 | 0 |
| 19 | 4.8 | 25.44 | 43.31 | 22.67 | 3.61 | 0.17 | 0 | 0 | 0 | 0 |  | 8.91 | 35.71 | 41.27 | 12.99 | 1.09 | 0.02 | 0 | 0 | 0 | 0 |  | 100 | 0 |
| 20 | 6.31 | 29.26 | 42.93 | 18.93 | 2.48 | 0.09 | 0 | 0 | 0 | 0 |  | 11.36 | 39.28 | 38.6 | 10.07 | 0.68 | 0.01 | 0 | 0 | 0 | 0 |  | 100 | 0 |
| 21 | 7.62 | 32.02 | 42.1 | 16.36 | 1.85 | 0.06 | 0 | 0 | 0 | 0 |  | 13.53 | 41.74 | 36.14 | 8.12 | 0.46 | 0.01 | 0 | 0 | 0 | 0 |  | 100 | 0 |
| 22 | 8.54 | 33.72 | 41.36 | 14.81 | 1.52 | 0.04 | 0 | 0 | 0 | 0 |  | 15.03 | 43.14 | 34.44 | 7.03 | 0.35 | 0 | 0 | 0 | 0 | 0 |  | 100 | 0 |
| 23 | 9.06 | 34.61 | 40.9 | 14.02 | 1.37 | 0.04 | 0 | 0 | 0 | 0 |  | 15.87 | 43.82 | 33.5 | 6.49 | 0.31 | 0 | 0 | 0 | 0 | 0 |  | 100 | 0 |
| 24 | 9.26 | 34.94 | 40.72 | 13.73 | 1.31 | 0.03 | 0 | 0 | 0 | 0 |  | 16.19 | 44.07 | 33.15 | 6.3 | 0.29 | 0 | 0 | 0 | 0 | 0 |  | 100 | 0 |
| 25 | 9.26 | 34.95 | 40.72 | 13.72 | 1.31 | 0.03 | 0 | 0 | 0 | 0 |  | 16 | 43.9 | 33.37 | 6.43 | 0.3 | 0 | 0 | 0 | 0 | 0 |  | 100 | 0 |
| 26 | 9.17 | 34.8 | 40.8 | 13.85 | 1.33 | 0.03 | 0 | 0 | 0 | 0 |  | 15.85 | 43.79 | 33.53 | 6.52 | 0.31 | 0 | 0 | 0 | 0 | 0 |  | 100 | 0 |
| 27 | 9.06 | 34.62 | 40.91 | 14.01 | 1.36 | 0.04 | 0 | 0 | 0 | 0 |  | 15.67 | 43.65 | 33.73 | 6.63 | 0.32 | 0 | 0 | 0 | 0 | 0 |  | 100 | 0 |
| 28 | 8.95 | 34.45 | 41 | 14.16 | 1.39 | 0.04 | 0 | 0 | 0 | 0 |  | 15.51 | 43.52 | 33.92 | 6.73 | 0.33 | 0 | 0 | 0 | 0 | 0 |  | 100 | 0 |
| 29 | 8.88 | 34.31 | 41.08 | 14.28 | 1.42 | 0.04 | 0 | 0 | 0 | 0 |  | 15.38 | 43.42 | 34.06 | 6.81 | 0.34 | 0 | 0 | 0 | 0 | 0 |  | 100 | 0 |
| 30 | 8.82 | 34.23 | 41.12 | 14.36 | 1.43 | 0.04 | 0 | 0 | 0 | 0 |  | 15.11 | 43.16 | 34.37 | 7 | 0.35 | 0 | 0 | 0 | 0 | 0 |  | 100 | 0 |
| 31 | 8.8 | 34.18 | 41.14 | 14.4 | 1.44 | 0.04 | 0 | 0 | 0 | 0 |  | 15.06 | 43.12 | 34.42 | 7.03 | 0.36 | 0 | 0 | 0 | 0 | 0 |  | 100 | 0 |
| 32 | 8.79 | 34.16 | 41.15 | 14.42 | 1.44 | 0.04 | 0 | 0 | 0 | 0 |  | 15.04 | 43.11 | 34.44 | 7.05 | 0.36 | 0 | 0 | 0 | 0 | 0 |  | 100 | 0 |
| 33 | 8.78 | 34.16 | 41.16 | 14.42 | 1.44 | 0.04 | 0 | 0 | 0 | 0 |  | 15.04 | 43.1 | 34.44 | 7.05 | 0.36 | 0 | 0 | 0 | 0 | 0 |  | 100 | 0 |
| 34 | 8.79 | 34.16 | 41.15 | 14.41 | 1.44 | 0.04 | 0 | 0 | 0 | 0 |  | 15.05 | 43.11 | 34.44 | 7.05 | 0.36 | 0 | 0 | 0 | 0 | 0 |  | 100 | 0 |
| 35 | 8.79 | 34.17 | 41.15 | 14.41 | 1.44 | 0.04 | 0 | 0 | 0 | 0 |  | 15.04 | 43.1 | 34.45 | 7.05 | 0.36 | 0 | 0 | 0 | 0 | 0 |  | 100 | 0 |
| 36 | 8.8 | 34.18 | 41.14 | 14.4 | 1.44 | 0.04 | 0 | 0 | 0 | 0 |  | 15.05 | 43.1 | 34.44 | 7.05 | 0.36 | 0 | 0 | 0 | 0 | 0 |  | 100 | 0 |
| 37 | 8.8 | 34.19 | 41.14 | 14.4 | 1.44 | 0.04 | 0 | 0 | 0 | 0 |  | 15.05 | 43.11 | 34.43 | 7.04 | 0.36 | 0 | 0 | 0 | 0 | 0 |  | 100 | 0 |
| 38 | 8.8 | 34.19 | 41.14 | 14.39 | 1.44 | 0.04 | 0 | 0 | 0 | 0 |  | 15.05 | 43.11 | 34.43 | 7.04 | 0.36 | 0 | 0 | 0 | 0 | 0 |  | 100 | 0 |
| 39 | 8.8 | 34.19 | 41.14 | 14.39 | 1.44 | 0.04 | 0 | 0 | 0 | 0 |  | 15.06 | 43.11 | 34.43 | 7.04 | 0.36 | 0 | 0 | 0 | 0 | 0 |  | 100 | 0 |
| 40 | 8.8 | 34.19 | 41.14 | 14.39 | 1.44 | 0.04 | 0 | 0 | 0 | 0 |  | 14.88 | 42.93 | 34.64 | 7.18 | 0.37 | 0 | 0 | 0 | 0 | 0 |  | 100 | 0 |
| 41 | 8.8 | 34.19 | 41.14 | 14.39 | 1.44 | 0.04 | 0 | 0 | 0 | 0 |  | 14.88 | 42.93 | 34.64 | 7.18 | 0.37 | 0 | 0 | 0 | 0 | 0 |  | 100 | 0 |
| 42 | 8.8 | 34.19 | 41.14 | 14.39 | 1.44 | 0.04 | 0 | 0 | 0 | 0 |  | 14.88 | 42.93 | 34.64 | 7.18 | 0.37 | 0 | 0 | 0 | 0 | 0 |  | 100 | 0 |
| 43 | 8.8 | 34.19 | 41.14 | 14.39 | 1.44 | 0.04 | 0 | 0 | 0 | 0 |  | 14.88 | 42.93 | 34.64 | 7.18 | 0.37 | 0 | 0 | 0 | 0 | 0 |  | 100 | 0 |
| 44 | 8.8 | 34.19 | 41.14 | 14.39 | 1.44 | 0.04 | 0 | 0 | 0 | 0 |  | 14.88 | 42.93 | 34.64 | 7.18 | 0.37 | 0 | 0 | 0 | 0 | 0 |  | 100 | 0 |
| 45 | 8.81 | 34.2 | 41.14 | 14.39 | 1.44 | 0.04 | 0 | 0 | 0 | 0 |  | 14.87 | 42.92 | 34.65 | 7.19 | 0.37 | 0 | 0 | 0 | 0 | 0 |  | 100 | 0 |
| 46 | 8.81 | 34.2 | 41.13 | 14.38 | 1.44 | 0.04 | 0 | 0 | 0 | 0 |  | 14.87 | 42.92 | 34.64 | 7.19 | 0.37 | 0 | 0 | 0 | 0 | 0 |  | 100 | 0 |
| 47 | 8.82 | 34.21 | 41.13 | 14.37 | 1.43 | 0.04 | 0 | 0 | 0 | 0 |  | 14.88 | 42.93 | 34.64 | 7.18 | 0.37 | 0 | 0 | 0 | 0 | 0 |  | 100 | 0 |
| 48 | 8.82 | 34.22 | 41.12 | 14.37 | 1.43 | 0.04 | 0 | 0 | 0 | 0 |  | 14.89 | 42.94 | 34.63 | 7.18 | 0.37 | 0 | 0 | 0 | 0 | 0 |  | 100 | 0 |
| 49 | 8.83 | 34.23 | 41.12 | 14.36 | 1.43 | 0.04 | 0 | 0 | 0 | 0 |  | 14.9 | 42.94 | 34.62 | 7.17 | 0.37 | 0 | 0 | 0 | 0 | 0 |  | 100 | 0 |
| 50 | 8.83 | 34.23 | 41.11 | 14.35 | 1.43 | 0.04 | 0 | 0 | 0 | 0 |  | 14.55 | 42.58 | 35.02 | 7.45 | 0.4 | 0 | 0 | 0 | 0 | 0 |  | 100 | 0 |
| 51 | 8.82 | 34.23 | 41.12 | 14.36 | 1.43 | 0.04 | 0 | 0 | 0 | 0 |  | 14.55 | 42.58 | 35.02 | 7.45 | 0.4 | 0 | 0 | 0 | 0 | 0 |  | 100 | 0 |
| 52 | 8.81 | 34.2 | 41.14 | 14.39 | 1.44 | 0.04 | 0 | 0 | 0 | 0 |  | 14.52 | 42.55 | 35.06 | 7.47 | 0.4 | 0 | 0 | 0 | 0 | 0 |  | 100 | 0 |
| 53 | 8.77 | 34.13 | 41.17 | 14.44 | 1.45 | 0.04 | 0 | 0 | 0 | 0 |  | 14.46 | 42.5 | 35.12 | 7.51 | 0.4 | 0 | 0 | 0 | 0 | 0 |  | 100 | 0 |
| 54 | 8.69 | 34.02 | 41.24 | 14.54 | 1.46 | 0.04 | 0 | 0 | 0 | 0 |  | 14.34 | 42.42 | 35.25 | 7.58 | 0.41 | 0 | 0 | 0 | 0 | 0 |  | 100 | 0 |
| 55 | 8.59 | 33.85 | 41.34 | 14.69 | 1.49 | 0.04 | 0 | 0 | 0 | 0 |  | 14.17 | 42.27 | 35.44 | 7.7 | 0.42 | 0 | 0 | 0 | 0 | 0 |  | 100 | 0 |
| 56 | 8.45 | 33.63 | 41.46 | 14.89 | 1.53 | 0.04 | 0 | 0 | 0 | 0 |  | 13.97 | 42.09 | 35.66 | 7.84 | 0.43 | 0.01 | 0 | 0 | 0 | 0 |  | 100 | 0 |
| 57 | 8.31 | 33.4 | 41.59 | 15.09 | 1.57 | 0.04 | 0 | 0 | 0 | 0 |  | 13.75 | 41.91 | 35.9 | 7.99 | 0.45 | 0.01 | 0 | 0 | 0 | 0 |  | 100 | 0 |
| 58 | 8.22 | 33.25 | 41.67 | 15.23 | 1.59 | 0.05 | 0 | 0 | 0 | 0 |  | 13.61 | 41.78 | 36.06 | 8.08 | 0.46 | 0.01 | 0 | 0 | 0 | 0 |  | 100 | 0 |
| 59 | 8.26 | 33.31 | 41.63 | 15.17 | 1.58 | 0.05 | 0 | 0 | 0 | 0 |  | 13.67 | 41.84 | 35.99 | 8.04 | 0.45 | 0.01 | 0 | 0 | 0 | 0 |  | 100 | 0 |
| 60 | 8.57 | 33.82 | 41.35 | 14.72 | 1.5 | 0.04 | 0 | 0 | 0 | 0 |  | 13.98 | 42.05 | 35.67 | 7.86 | 0.44 | 0.01 | 0 | 0 | 0 | 0 |  | 100 | 0 |
| 61 | 9.37 | 35.04 | 40.61 | 13.64 | 1.3 | 0.03 | 0 | 0 | 0 | 0 |  | 15.16 | 43 | 34.37 | 7.1 | 0.37 | 0 | 0 | 0 | 0 | 0 |  | 100 | 0 |
| 62 | 10.98 | 37.2 | 39.03 | 11.77 | 1 | 0.02 | 0 | 0 | 0 | 0 |  | 17.53 | 44.54 | 31.85 | 5.82 | 0.26 | 0 | 0 | 0 | 0 | 0 |  | 100 | 0 |
| 63 | 13.92 | 40.25 | 36.04 | 9.14 | 0.64 | 0.01 | 0 | 0 | 0 | 0 |  | 21.72 | 46.28 | 27.7 | 4.15 | 0.15 | 0 | 0 | 0 | 0 | 0 |  | 100 | 0 |
| 64 | 18.59 | 43.44 | 31.36 | 6.27 | 0.34 | 0 | 0 | 0 | 0 | 0 |  | 28.14 | 47.14 | 22.13 | 2.52 | 0.07 | 0 | 0 | 0 | 0 | 0 |  | 100 | 0 |
| 65 | 25.03 | 45.49 | 25.49 | 3.83 | 0.15 | 0 | 0 | 0 | 0 | 0 |  | 36.03 | 46.05 | 16.51 | 1.38 | 0.03 | 0 | 0 | 0 | 0 | 0 |  | 100 | 0 |
| 66 | 31.7 | 45.66 | 20.24 | 2.33 | 0.07 | 0 | 0 | 0 | 0 | 0 |  | 44.19 | 43.24 | 11.84 | 0.73 | 0.01 | 0 | 0 | 0 | 0 | 0 |  | 100 | 0 |
| 67 | 37.31 | 44.68 | 16.45 | 1.53 | 0.03 | 0 | 0 | 0 | 0 | 0 |  | 50.72 | 40.03 | 8.83 | 0.42 | 0 | 0 | 0 | 0 | 0 | 0 |  | 100 | 0 |
| 68 | 41.09 | 43.56 | 14.18 | 1.14 | 0.02 | 0 | 0 | 0 | 0 | 0 |  | 54.95 | 37.57 | 7.19 | 0.29 | 0 | 0 | 0 | 0 | 0 | 0 |  | 100 | 0 |
| 69 | 43.2 | 42.79 | 13.02 | 0.97 | 0.02 | 0 | 0 | 0 | 0 | 0 |  | 57.25 | 36.13 | 6.38 | 0.23 | 0 | 0 | 0 | 0 | 0 | 0 |  | 100 | 0 |
| 70 | 44.08 | 42.45 | 12.55 | 0.9 | 0.02 | 0 | 0 | 0 | 0 | 0 |  | 57.82 | 35.75 | 6.21 | 0.22 | 0 | 0 | 0 | 0 | 0 | 0 |  | 100 | 0 |
| 71 | 44.19 | 42.4 | 12.49 | 0.9 | 0.02 | 0 | 0 | 0 | 0 | 0 |  | 57.94 | 35.68 | 6.17 | 0.22 | 0 | 0 | 0 | 0 | 0 | 0 |  | 100 | 0 |
| 72 | 43.93 | 42.51 | 12.63 | 0.92 | 0.02 | 0 | 0 | 0 | 0 | 0 |  | 57.66 | 35.86 | 6.26 | 0.23 | 0 | 0 | 0 | 0 | 0 | 0 |  | 100 | 0 |
| 73 | 43.53 | 42.67 | 12.84 | 0.94 | 0.02 | 0 | 0 | 0 | 0 | 0 |  | 57.22 | 36.14 | 6.4 | 0.24 | 0 | 0 | 0 | 0 | 0 | 0 |  | 100 | 0 |
| 74 | 43.16 | 42.81 | 13.04 | 0.97 | 0.02 | 0 | 0 | 0 | 0 | 0 |  | 56.82 | 36.39 | 6.54 | 0.24 | 0 | 0 | 0 | 0 | 0 | 0 |  | 100 | 0 |
| 75 | 42.86 | 42.92 | 13.2 | 1 | 0.02 | 0 | 0 | 0 | 0 | 0 |  | 56.12 | 36.82 | 6.79 | 0.26 | 0 | 0 | 0 | 0 | 0 | 0 |  | 100 | 0 |
| 76 | 42.67 | 43 | 13.31 | 1.01 | 0.02 | 0 | 0 | 0 | 0 | 0 |  | 55.91 | 36.95 | 6.87 | 0.27 | 0 | 0 | 0 | 0 | 0 | 0 |  | 100 | 0 |
| 77 | 42.56 | 43.04 | 13.37 | 1.02 | 0.02 | 0 | 0 | 0 | 0 | 0 |  | 55.79 | 37.03 | 6.91 | 0.27 | 0 | 0 | 0 | 0 | 0 | 0 |  | 100 | 0 |
| 78 | 42.5 | 43.06 | 13.4 | 1.02 | 0.02 | 0 | 0 | 0 | 0 | 0 |  | 55.73 | 37.06 | 6.93 | 0.27 | 0 | 0 | 0 | 0 | 0 | 0 |  | 100 | 0 |
| 79 | 42.49 | 43.06 | 13.4 | 1.03 | 0.02 | 0 | 0 | 0 | 0 | 0 |  | 55.72 | 37.07 | 6.93 | 0.27 | 0 | 0 | 0 | 0 | 0 | 0 |  | 100 | 0 |
| 80 | 42.5 | 43.06 | 13.4 | 1.02 | 0.02 | 0 | 0 | 0 | 0 | 0 |  | 55.37 | 37.28 | 7.07 | 0.28 | 0 | 0 | 0 | 0 | 0 | 0 |  | 100 | 0 |
| 81 | 42.52 | 43.05 | 13.39 | 1.02 | 0.02 | 0 | 0 | 0 | 0 | 0 |  | 55.38 | 37.26 | 7.07 | 0.28 | 0 | 0 | 0 | 0 | 0 | 0 |  | 100 | 0 |
| 82 | 42.53 | 43.05 | 13.38 | 1.02 | 0.02 | 0 | 0 | 0 | 0 | 0 |  | 55.4 | 37.25 | 7.06 | 0.28 | 0 | 0 | 0 | 0 | 0 | 0 |  | 100 | 0 |
| 83 | 42.55 | 43.04 | 13.37 | 1.02 | 0.02 | 0 | 0 | 0 | 0 | 0 |  | 55.42 | 37.24 | 7.05 | 0.28 | 0 | 0 | 0 | 0 | 0 | 0 |  | 100 | 0 |
| 84 | 42.56 | 43.04 | 13.37 | 1.02 | 0.02 | 0 | 0 | 0 | 0 | 0 |  | 55.43 | 37.24 | 7.05 | 0.28 | 0 | 0 | 0 | 0 | 0 | 0 |  | 100 | 0 |
| 85 | 42.56 | 43.04 | 13.36 | 1.02 | 0.02 | 0 | 0 | 0 | 0 | 0 |  | 54.72 | 37.64 | 7.33 | 0.3 | 0 | 0 | 0 | 0 | 0 | 0 |  | 100 | 0 |
| 86 | 42.57 | 43.04 | 13.36 | 1.02 | 0.02 | 0 | 0 | 0 | 0 | 0 |  | 54.72 | 37.64 | 7.33 | 0.3 | 0 | 0 | 0 | 0 | 0 | 0 |  | 100 | 0 |
| 87 | 42.57 | 43.04 | 13.36 | 1.02 | 0.02 | 0 | 0 | 0 | 0 | 0 |  | 54.72 | 37.64 | 7.33 | 0.3 | 0 | 0 | 0 | 0 | 0 | 0 |  | 100 | 0 |
| 88 | 42.57 | 43.04 | 13.36 | 1.02 | 0.02 | 0 | 0 | 0 | 0 | 0 |  | 54.72 | 37.64 | 7.33 | 0.3 | 0 | 0 | 0 | 0 | 0 | 0 |  | 100 | 0 |
| 89 | 42.57 | 43.04 | 13.36 | 1.02 | 0.02 | 0 | 0 | 0 | 0 | 0 |  | 54.72 | 37.64 | 7.33 | 0.3 | 0 | 0 | 0 | 0 | 0 | 0 |  | 100 | 0 |
| 90 | 42.56 | 43.04 | 13.36 | 1.02 | 0.02 | 0 | 0 | 0 | 0 | 0 |  | 54.72 | 37.64 | 7.33 | 0.3 | 0 | 0 | 0 | 0 | 0 | 0 |  | 100 | 0 |
| 91 | 42.56 | 43.04 | 13.36 | 1.02 | 0.02 | 0 | 0 | 0 | 0 | 0 |  | 54.72 | 37.64 | 7.33 | 0.3 | 0 | 0 | 0 | 0 | 0 | 0 |  | 100 | 0 |
| 92 | 42.56 | 43.04 | 13.36 | 1.02 | 0.02 | 0 | 0 | 0 | 0 | 0 |  | 54.72 | 37.64 | 7.33 | 0.3 | 0 | 0 | 0 | 0 | 0 | 0 |  | 100 | 0 |
| 93 | 42.56 | 43.04 | 13.36 | 1.02 | 0.02 | 0 | 0 | 0 | 0 | 0 |  | 54.72 | 37.64 | 7.33 | 0.3 | 0 | 0 | 0 | 0 | 0 | 0 |  | 100 | 0 |
| 94 | 42.56 | 43.04 | 13.36 | 1.02 | 0.02 | 0 | 0 | 0 | 0 | 0 |  | 54.72 | 37.64 | 7.33 | 0.3 | 0 | 0 | 0 | 0 | 0 | 0 |  | 100 | 0 |
| 95 | 42.56 | 43.04 | 13.36 | 1.02 | 0.02 | 0 | 0 | 0 | 0 | 0 |  | 54.72 | 37.64 | 7.33 | 0.3 | 0 | 0 | 0 | 0 | 0 | 0 |  | 100 | 0 |
|  | |  | Females | | | | | | | | | | | | | | | | | | | | | |
| 0 | 100 | 0 | 0 | 0 | 0 | 0 | 0 | 0 | 0 | 0 |  | 100 | 0 | 0 | 0 | 0 | 0 | 0 | 0 | 0 | 0 |  | 100 | 0 |
| 1 | 100 | 0 | 0 | 0 | 0 | 0 | 0 | 0 | 0 | 0 |  | 100 | 0 | 0 | 0 | 0 | 0 | 0 | 0 | 0 | 0 |  | 100 | 0 |
| 2 | 100 | 0 | 0 | 0 | 0 | 0 | 0 | 0 | 0 | 0 |  | 100 | 0 | 0 | 0 | 0 | 0 | 0 | 0 | 0 | 0 |  | 100 | 0 |
| 3 | 100 | 0 | 0 | 0 | 0 | 0 | 0 | 0 | 0 | 0 |  | 100 | 0 | 0 | 0 | 0 | 0 | 0 | 0 | 0 | 0 |  | 100 | 0 |
| 4 | 100 | 0 | 0 | 0 | 0 | 0 | 0 | 0 | 0 | 0 |  | 100 | 0 | 0 | 0 | 0 | 0 | 0 | 0 | 0 | 0 |  | 100 | 0 |
| 5 | 100 | 0 | 0 | 0 | 0 | 0 | 0 | 0 | 0 | 0 |  | 100 | 0 | 0 | 0 | 0 | 0 | 0 | 0 | 0 | 0 |  | 100 | 0 |
| 6 | 0.1 | 8.78 | 56.74 | 32.81 | 1.56 | 0 | 0 | 0 | 0 | 0 |  | 0.1 | 8.78 | 56.74 | 32.81 | 1.56 | 0 | 0 | 0 | 0 | 0 |  | 100 | 0 |
| 7 | 0.1 | 8.63 | 56.18 | 33.41 | 1.68 | 0.01 | 0 | 0 | 0 | 0 |  | 0.1 | 8.63 | 56.18 | 33.41 | 1.68 | 0.01 | 0 | 0 | 0 | 0 |  | 100 | 0 |
| 8 | 0.1 | 8.47 | 55.62 | 34 | 1.8 | 0.01 | 0 | 0 | 0 | 0 |  | 0.1 | 8.47 | 55.62 | 34 | 1.8 | 0.01 | 0 | 0 | 0 | 0 |  | 100 | 0 |
| 9 | 0.1 | 8.32 | 55.05 | 34.59 | 1.93 | 0.01 | 0 | 0 | 0 | 0 |  | 0.1 | 8.32 | 55.05 | 34.59 | 1.93 | 0.01 | 0 | 0 | 0 | 0 |  | 100 | 0 |
| 10 | 0.1 | 8.18 | 54.48 | 35.16 | 2.07 | 0.01 | 0 | 0 | 0 | 0 |  | 0.1 | 8.18 | 54.48 | 35.16 | 2.07 | 0.01 | 0 | 0 | 0 | 0 |  | 100 | 0 |
| 11 | 0.1 | 8.05 | 53.91 | 35.72 | 2.21 | 0.01 | 0 | 0 | 0 | 0 |  | 0.1 | 8.05 | 53.91 | 35.72 | 2.21 | 0.01 | 0 | 0 | 0 | 0 |  | 100 | 0 |
| 12 | 0.1 | 8 | 53.3 | 36.21 | 2.38 | 0.01 | 0 | 0 | 0 | 0 |  | 0.1 | 8 | 53.3 | 36.21 | 2.38 | 0.01 | 0 | 0 | 0 | 0 |  | 100 | 0 |
| 13 | 0.11 | 8.16 | 52.63 | 36.48 | 2.6 | 0.02 | 0 | 0 | 0 | 0 |  | 0.11 | 8.16 | 52.63 | 36.48 | 2.6 | 0.02 | 0 | 0 | 0 | 0 |  | 100 | 0 |
| 14 | 0.15 | 8.76 | 51.96 | 36.25 | 2.86 | 0.02 | 0 | 0 | 0 | 0 |  | 0.15 | 8.76 | 51.96 | 36.25 | 2.86 | 0.02 | 0 | 0 | 0 | 0 |  | 100 | 0 |
| 15 | 0.24 | 10.06 | 51.32 | 35.21 | 3.14 | 0.03 | 0 | 0 | 0 | 0 |  | 0.51 | 16.43 | 57.44 | 24.44 | 1.16 | 0 | 0 | 0 | 0 | 0 |  | 100 | 0 |
| 16 | 0.45 | 12.42 | 50.67 | 33.06 | 3.35 | 0.05 | 0 | 0 | 0 | 0 |  | 0.9 | 19.2 | 55.38 | 23.19 | 1.32 | 0.01 | 0 | 0 | 0 | 0 |  | 100 | 0 |
| 17 | 0.97 | 16.01 | 49.59 | 29.93 | 3.44 | 0.07 | 0 | 0 | 0 | 0 |  | 1.76 | 23.18 | 52.49 | 21.12 | 1.44 | 0.02 | 0 | 0 | 0 | 0 |  | 100 | 0 |
| 18 | 2.1 | 20.55 | 47.62 | 26.25 | 3.38 | 0.1 | 0 | 0 | 0 | 0 |  | 3.49 | 27.81 | 48.62 | 18.56 | 1.5 | 0.02 | 0 | 0 | 0 | 0 |  | 100 | 0 |
| 19 | 4.01 | 24.92 | 44.9 | 22.82 | 3.23 | 0.12 | 0 | 0 | 0 | 0 |  | 6.28 | 32.14 | 44.28 | 15.83 | 1.43 | 0.03 | 0 | 0 | 0 | 0 |  | 100 | 0 |
| 20 | 6.34 | 28.11 | 42.17 | 20.18 | 3.05 | 0.14 | 0 | 0 | 0 | 0 |  | 9.38 | 34.68 | 40.53 | 13.97 | 1.4 | 0.04 | 0 | 0 | 0 | 0 |  | 100 | 0 |
| 21 | 8.36 | 29.97 | 40.13 | 18.49 | 2.9 | 0.15 | 0 | 0 | 0 | 0 |  | 11.95 | 35.97 | 37.92 | 12.77 | 1.35 | 0.04 | 0 | 0 | 0 | 0 |  | 100 | 0 |
| 22 | 9.77 | 30.95 | 38.82 | 17.49 | 2.81 | 0.16 | 0 | 0 | 0 | 0 |  | 13.7 | 36.56 | 36.32 | 12.05 | 1.32 | 0.05 | 0 | 0 | 0 | 0 |  | 100 | 0 |
| 23 | 10.58 | 31.44 | 38.11 | 16.96 | 2.75 | 0.16 | 0 | 0 | 0 | 0 |  | 14.7 | 36.83 | 35.47 | 11.67 | 1.29 | 0.05 | 0 | 0 | 0 | 0 |  | 100 | 0 |
| 24 | 10.91 | 31.63 | 37.83 | 16.74 | 2.72 | 0.16 | 0 | 0 | 0 | 0 |  | 15.1 | 36.93 | 35.12 | 11.51 | 1.28 | 0.05 | 0 | 0 | 0 | 0 |  | 100 | 0 |
| 25 | 10.96 | 31.66 | 37.8 | 16.71 | 2.71 | 0.16 | 0 | 0 | 0 | 0 |  | 15.3 | 37.1 | 34.97 | 11.34 | 1.25 | 0.05 | 0 | 0 | 0 | 0 |  | 100 | 0 |
| 26 | 10.85 | 31.61 | 37.89 | 16.76 | 2.72 | 0.16 | 0 | 0 | 0 | 0 |  | 15.17 | 37.08 | 35.08 | 11.38 | 1.25 | 0.05 | 0 | 0 | 0 | 0 |  | 100 | 0 |
| 27 | 10.7 | 31.54 | 38.03 | 16.85 | 2.72 | 0.16 | 0 | 0 | 0 | 0 |  | 14.99 | 37.05 | 35.23 | 11.43 | 1.25 | 0.04 | 0 | 0 | 0 | 0 |  | 100 | 0 |
| 28 | 10.55 | 31.46 | 38.15 | 16.94 | 2.73 | 0.16 | 0 | 0 | 0 | 0 |  | 14.81 | 37.02 | 35.38 | 11.49 | 1.25 | 0.04 | 0 | 0 | 0 | 0 |  | 100 | 0 |
| 29 | 10.44 | 31.4 | 38.25 | 17.01 | 2.74 | 0.16 | 0 | 0 | 0 | 0 |  | 14.67 | 36.99 | 35.5 | 11.54 | 1.26 | 0.04 | 0 | 0 | 0 | 0 |  | 100 | 0 |
| 30 | 10.37 | 31.36 | 38.32 | 17.05 | 2.74 | 0.16 | 0 | 0 | 0 | 0 |  | 14.87 | 37.27 | 35.34 | 11.28 | 1.2 | 0.04 | 0 | 0 | 0 | 0 |  | 100 | 0 |
| 31 | 10.32 | 31.34 | 38.35 | 17.08 | 2.74 | 0.16 | 0 | 0 | 0 | 0 |  | 14.81 | 37.26 | 35.39 | 11.3 | 1.2 | 0.04 | 0 | 0 | 0 | 0 |  | 100 | 0 |
| 32 | 10.3 | 31.32 | 38.37 | 17.09 | 2.75 | 0.16 | 0 | 0 | 0 | 0 |  | 14.79 | 37.26 | 35.41 | 11.31 | 1.2 | 0.04 | 0 | 0 | 0 | 0 |  | 100 | 0 |
| 33 | 10.3 | 31.32 | 38.38 | 17.1 | 2.75 | 0.16 | 0 | 0 | 0 | 0 |  | 14.78 | 37.25 | 35.41 | 11.31 | 1.2 | 0.04 | 0 | 0 | 0 | 0 |  | 100 | 0 |
| 34 | 10.3 | 31.32 | 38.37 | 17.09 | 2.75 | 0.16 | 0 | 0 | 0 | 0 |  | 14.79 | 37.26 | 35.41 | 11.31 | 1.2 | 0.04 | 0 | 0 | 0 | 0 |  | 100 | 0 |
| 35 | 10.31 | 31.33 | 38.37 | 17.09 | 2.75 | 0.16 | 0 | 0 | 0 | 0 |  | 14.8 | 37.26 | 35.4 | 11.31 | 1.2 | 0.04 | 0 | 0 | 0 | 0 |  | 100 | 0 |
| 36 | 10.32 | 31.33 | 38.36 | 17.09 | 2.75 | 0.16 | 0 | 0 | 0 | 0 |  | 14.8 | 37.26 | 35.4 | 11.3 | 1.2 | 0.04 | 0 | 0 | 0 | 0 |  | 100 | 0 |
| 37 | 10.32 | 31.33 | 38.36 | 17.08 | 2.75 | 0.16 | 0 | 0 | 0 | 0 |  | 14.81 | 37.26 | 35.39 | 11.3 | 1.2 | 0.04 | 0 | 0 | 0 | 0 |  | 100 | 0 |
| 38 | 10.32 | 31.33 | 38.35 | 17.08 | 2.75 | 0.16 | 0 | 0 | 0 | 0 |  | 14.81 | 37.26 | 35.39 | 11.3 | 1.2 | 0.04 | 0 | 0 | 0 | 0 |  | 100 | 0 |
| 39 | 10.33 | 31.34 | 38.35 | 17.08 | 2.75 | 0.16 | 0 | 0 | 0 | 0 |  | 14.82 | 37.26 | 35.38 | 11.3 | 1.2 | 0.04 | 0 | 0 | 0 | 0 |  | 100 | 0 |
| 40 | 10.33 | 31.34 | 38.35 | 17.08 | 2.75 | 0.16 | 0 | 0 | 0 | 0 |  | 15.11 | 37.56 | 35.14 | 11.01 | 1.14 | 0.04 | 0 | 0 | 0 | 0 |  | 100 | 0 |
| 41 | 10.33 | 31.34 | 38.35 | 17.08 | 2.75 | 0.16 | 0 | 0 | 0 | 0 |  | 15.11 | 37.56 | 35.14 | 11.01 | 1.14 | 0.04 | 0 | 0 | 0 | 0 |  | 100 | 0 |
| 42 | 10.33 | 31.34 | 38.35 | 17.08 | 2.75 | 0.16 | 0 | 0 | 0 | 0 |  | 15.11 | 37.56 | 35.14 | 11.01 | 1.14 | 0.04 | 0 | 0 | 0 | 0 |  | 100 | 0 |
| 43 | 10.33 | 31.34 | 38.35 | 17.08 | 2.75 | 0.16 | 0 | 0 | 0 | 0 |  | 15.11 | 37.56 | 35.14 | 11.01 | 1.14 | 0.04 | 0 | 0 | 0 | 0 |  | 100 | 0 |
| 44 | 10.33 | 31.34 | 38.35 | 17.08 | 2.75 | 0.16 | 0 | 0 | 0 | 0 |  | 15.11 | 37.57 | 35.14 | 11 | 1.14 | 0.04 | 0 | 0 | 0 | 0 |  | 100 | 0 |
| 45 | 10.33 | 31.34 | 38.35 | 17.08 | 2.74 | 0.16 | 0 | 0 | 0 | 0 |  | 15.11 | 37.57 | 35.14 | 11 | 1.14 | 0.04 | 0 | 0 | 0 | 0 |  | 100 | 0 |
| 46 | 10.33 | 31.34 | 38.35 | 17.07 | 2.74 | 0.16 | 0 | 0 | 0 | 0 |  | 15.11 | 37.57 | 35.14 | 11 | 1.14 | 0.04 | 0 | 0 | 0 | 0 |  | 100 | 0 |
| 47 | 10.33 | 31.35 | 38.35 | 17.07 | 2.74 | 0.16 | 0 | 0 | 0 | 0 |  | 15.11 | 37.58 | 35.14 | 10.99 | 1.13 | 0.04 | 0 | 0 | 0 | 0 |  | 100 | 0 |
| 48 | 10.33 | 31.36 | 38.36 | 17.06 | 2.74 | 0.16 | 0 | 0 | 0 | 0 |  | 15.12 | 37.59 | 35.14 | 10.98 | 1.13 | 0.04 | 0 | 0 | 0 | 0 |  | 100 | 0 |
| 49 | 10.33 | 31.37 | 38.36 | 17.05 | 2.73 | 0.16 | 0 | 0 | 0 | 0 |  | 15.12 | 37.6 | 35.14 | 10.98 | 1.13 | 0.04 | 0 | 0 | 0 | 0 |  | 100 | 0 |
| 50 | 10.33 | 31.37 | 38.36 | 17.05 | 2.73 | 0.16 | 0 | 0 | 0 | 0 |  | 15.27 | 37.75 | 35.01 | 10.83 | 1.1 | 0.04 | 0 | 0 | 0 | 0 |  | 100 | 0 |
| 51 | 10.33 | 31.37 | 38.36 | 17.05 | 2.73 | 0.16 | 0 | 0 | 0 | 0 |  | 15.26 | 37.75 | 35.02 | 10.84 | 1.1 | 0.04 | 0 | 0 | 0 | 0 |  | 100 | 0 |
| 52 | 10.33 | 31.34 | 38.35 | 17.08 | 2.74 | 0.16 | 0 | 0 | 0 | 0 |  | 15.25 | 37.72 | 35.02 | 10.86 | 1.11 | 0.04 | 0 | 0 | 0 | 0 |  | 100 | 0 |
| 53 | 10.32 | 31.28 | 38.34 | 17.13 | 2.77 | 0.16 | 0 | 0 | 0 | 0 |  | 15.23 | 37.66 | 35.04 | 10.91 | 1.12 | 0.04 | 0 | 0 | 0 | 0 |  | 100 | 0 |
| 54 | 10.3 | 31.18 | 38.32 | 17.22 | 2.81 | 0.16 | 0 | 0 | 0 | 0 |  | 15.19 | 37.55 | 35.07 | 11.01 | 1.14 | 0.04 | 0 | 0 | 0 | 0 |  | 100 | 0 |
| 55 | 10.27 | 31.04 | 38.28 | 17.36 | 2.87 | 0.17 | 0 | 0 | 0 | 0 |  | 15.29 | 37.54 | 34.99 | 10.99 | 1.15 | 0.04 | 0 | 0 | 0 | 0 |  | 100 | 0 |
| 56 | 10.24 | 30.86 | 38.23 | 17.54 | 2.95 | 0.18 | 0 | 0 | 0 | 0 |  | 15.21 | 37.33 | 35.05 | 11.17 | 1.19 | 0.04 | 0 | 0 | 0 | 0 |  | 100 | 0 |
| 57 | 10.2 | 30.66 | 38.18 | 17.72 | 3.04 | 0.19 | 0 | 0 | 0 | 0 |  | 15.14 | 37.11 | 35.1 | 11.36 | 1.24 | 0.04 | 0 | 0 | 0 | 0 |  | 100 | 0 |
| 58 | 10.18 | 30.53 | 38.15 | 17.84 | 3.1 | 0.2 | 0 | 0 | 0 | 0 |  | 15.09 | 36.97 | 35.14 | 11.48 | 1.27 | 0.05 | 0 | 0 | 0 | 0 |  | 100 | 0 |
| 59 | 10.19 | 30.59 | 38.16 | 17.79 | 3.08 | 0.19 | 0 | 0 | 0 | 0 |  | 15.11 | 37.03 | 35.12 | 11.43 | 1.26 | 0.05 | 0 | 0 | 0 | 0 |  | 100 | 0 |
| 60 | 10.27 | 31.01 | 38.27 | 17.38 | 2.88 | 0.17 | 0 | 0 | 0 | 0 |  | 15.42 | 37.66 | 34.88 | 10.88 | 1.13 | 0.04 | 0 | 0 | 0 | 0 |  | 100 | 0 |
| 61 | 10.47 | 32.1 | 38.5 | 16.36 | 2.44 | 0.13 | 0 | 0 | 0 | 0 |  | 15.85 | 38.85 | 34.49 | 9.88 | 0.9 | 0.02 | 0 | 0 | 0 | 0 |  | 100 | 0 |
| 62 | 10.89 | 34.21 | 38.7 | 14.39 | 1.74 | 0.07 | 0 | 0 | 0 | 0 |  | 16.73 | 41.11 | 33.48 | 8.1 | 0.57 | 0.01 | 0 | 0 | 0 | 0 |  | 100 | 0 |
| 63 | 11.67 | 37.88 | 38.33 | 11.18 | 0.92 | 0.02 | 0 | 0 | 0 | 0 |  | 18.35 | 44.81 | 31.07 | 5.53 | 0.24 | 0 | 0 | 0 | 0 | 0 |  | 100 | 0 |
| 64 | 13.01 | 43.33 | 36.23 | 7.11 | 0.31 | 0 | 0 | 0 | 0 | 0 |  | 21.1 | 49.68 | 26.32 | 2.83 | 0.06 | 0 | 0 | 0 | 0 | 0 |  | 100 | 0 |
| 65 | 15.12 | 50.16 | 31.26 | 3.4 | 0.06 | 0 | 0 | 0 | 0 | 0 |  | 25.71 | 54.46 | 18.89 | 0.94 | 0.01 | 0 | 0 | 0 | 0 | 0 |  | 100 | 0 |
| 66 | 17.73 | 56.23 | 24.68 | 1.35 | 0.01 | 0 | 0 | 0 | 0 | 0 |  | 30.93 | 56.68 | 12.14 | 0.25 | 0 | 0 | 0 | 0 | 0 | 0 |  | 100 | 0 |
| 67 | 20.38 | 60.28 | 18.82 | 0.52 | 0 | 0 | 0 | 0 | 0 | 0 |  | 36.05 | 56.32 | 7.56 | 0.06 | 0 | 0 | 0 | 0 | 0 | 0 |  | 100 | 0 |
| 68 | 22.46 | 62.29 | 15 | 0.25 | 0 | 0 | 0 | 0 | 0 | 0 |  | 39.96 | 54.87 | 5.15 | 0.02 | 0 | 0 | 0 | 0 | 0 | 0 |  | 100 | 0 |
| 69 | 23.76 | 63.1 | 12.98 | 0.16 | 0 | 0 | 0 | 0 | 0 | 0 |  | 42.31 | 53.63 | 4.05 | 0.01 | 0 | 0 | 0 | 0 | 0 | 0 |  | 100 | 0 |
| 70 | 24.32 | 63.37 | 12.18 | 0.13 | 0 | 0 | 0 | 0 | 0 | 0 |  | 42.79 | 53.43 | 3.77 | 0.01 | 0 | 0 | 0 | 0 | 0 | 0 |  | 100 | 0 |
| 71 | 24.39 | 63.4 | 12.08 | 0.12 | 0 | 0 | 0 | 0 | 0 | 0 |  | 42.92 | 53.35 | 3.72 | 0.01 | 0 | 0 | 0 | 0 | 0 | 0 |  | 100 | 0 |
| 72 | 24.22 | 63.33 | 12.31 | 0.13 | 0 | 0 | 0 | 0 | 0 | 0 |  | 42.62 | 53.53 | 3.84 | 0.01 | 0 | 0 | 0 | 0 | 0 | 0 |  | 100 | 0 |
| 73 | 23.96 | 63.21 | 12.68 | 0.15 | 0 | 0 | 0 | 0 | 0 | 0 |  | 42.15 | 53.8 | 4.03 | 0.01 | 0 | 0 | 0 | 0 | 0 | 0 |  | 100 | 0 |
| 74 | 23.72 | 63.09 | 13.03 | 0.16 | 0 | 0 | 0 | 0 | 0 | 0 |  | 41.73 | 54.05 | 4.21 | 0.01 | 0 | 0 | 0 | 0 | 0 | 0 |  | 100 | 0 |
| 75 | 23.54 | 62.99 | 13.3 | 0.17 | 0 | 0 | 0 | 0 | 0 | 0 |  | 41.38 | 54.24 | 4.36 | 0.01 | 0 | 0 | 0 | 0 | 0 | 0 |  | 100 | 0 |
| 76 | 23.42 | 62.92 | 13.48 | 0.18 | 0 | 0 | 0 | 0 | 0 | 0 |  | 41.16 | 54.36 | 4.46 | 0.02 | 0 | 0 | 0 | 0 | 0 | 0 |  | 100 | 0 |
| 77 | 23.35 | 62.88 | 13.59 | 0.18 | 0 | 0 | 0 | 0 | 0 | 0 |  | 41.04 | 54.43 | 4.52 | 0.02 | 0 | 0 | 0 | 0 | 0 | 0 |  | 100 | 0 |
| 78 | 23.32 | 62.86 | 13.64 | 0.18 | 0 | 0 | 0 | 0 | 0 | 0 |  | 40.98 | 54.46 | 4.55 | 0.02 | 0 | 0 | 0 | 0 | 0 | 0 |  | 100 | 0 |
| 79 | 23.31 | 62.86 | 13.65 | 0.18 | 0 | 0 | 0 | 0 | 0 | 0 |  | 40.97 | 54.46 | 4.55 | 0.02 | 0 | 0 | 0 | 0 | 0 | 0 |  | 100 | 0 |
| 80 | 23.31 | 62.86 | 13.64 | 0.18 | 0 | 0 | 0 | 0 | 0 | 0 |  | 39.95 | 55.17 | 4.86 | 0.02 | 0 | 0 | 0 | 0 | 0 | 0 |  | 100 | 0 |
| 81 | 23.32 | 62.86 | 13.63 | 0.18 | 0 | 0 | 0 | 0 | 0 | 0 |  | 39.97 | 55.16 | 4.85 | 0.02 | 0 | 0 | 0 | 0 | 0 | 0 |  | 100 | 0 |
| 82 | 23.33 | 62.87 | 13.61 | 0.18 | 0 | 0 | 0 | 0 | 0 | 0 |  | 39.99 | 55.15 | 4.84 | 0.02 | 0 | 0 | 0 | 0 | 0 | 0 |  | 100 | 0 |
| 83 | 23.34 | 62.88 | 13.6 | 0.18 | 0 | 0 | 0 | 0 | 0 | 0 |  | 40.01 | 55.14 | 4.83 | 0.02 | 0 | 0 | 0 | 0 | 0 | 0 |  | 100 | 0 |
| 84 | 23.35 | 62.88 | 13.59 | 0.18 | 0 | 0 | 0 | 0 | 0 | 0 |  | 40.02 | 55.14 | 4.83 | 0.02 | 0 | 0 | 0 | 0 | 0 | 0 |  | 100 | 0 |
| 85 | 23.35 | 62.88 | 13.58 | 0.18 | 0 | 0 | 0 | 0 | 0 | 0 |  | 39.52 | 55.48 | 4.98 | 0.02 | 0 | 0 | 0 | 0 | 0 | 0 |  | 100 | 0 |
| 86 | 23.35 | 62.88 | 13.58 | 0.18 | 0 | 0 | 0 | 0 | 0 | 0 |  | 39.52 | 55.48 | 4.98 | 0.02 | 0 | 0 | 0 | 0 | 0 | 0 |  | 100 | 0 |
| 87 | 23.36 | 62.88 | 13.58 | 0.18 | 0 | 0 | 0 | 0 | 0 | 0 |  | 39.52 | 55.48 | 4.98 | 0.02 | 0 | 0 | 0 | 0 | 0 | 0 |  | 100 | 0 |
| 88 | 23.36 | 62.88 | 13.58 | 0.18 | 0 | 0 | 0 | 0 | 0 | 0 |  | 39.52 | 55.48 | 4.98 | 0.02 | 0 | 0 | 0 | 0 | 0 | 0 |  | 100 | 0 |
| 89 | 23.35 | 62.88 | 13.58 | 0.18 | 0 | 0 | 0 | 0 | 0 | 0 |  | 39.52 | 55.48 | 4.98 | 0.02 | 0 | 0 | 0 | 0 | 0 | 0 |  | 100 | 0 |
| 90 | 23.35 | 62.88 | 13.58 | 0.18 | 0 | 0 | 0 | 0 | 0 | 0 |  | 39.52 | 55.48 | 4.98 | 0.02 | 0 | 0 | 0 | 0 | 0 | 0 |  | 100 | 0 |
| 91 | 23.35 | 62.88 | 13.58 | 0.18 | 0 | 0 | 0 | 0 | 0 | 0 |  | 39.52 | 55.48 | 4.98 | 0.02 | 0 | 0 | 0 | 0 | 0 | 0 |  | 100 | 0 |
| 92 | 23.35 | 62.88 | 13.58 | 0.18 | 0 | 0 | 0 | 0 | 0 | 0 |  | 39.52 | 55.48 | 4.98 | 0.02 | 0 | 0 | 0 | 0 | 0 | 0 |  | 100 | 0 |
| 93 | 23.35 | 62.88 | 13.58 | 0.18 | 0 | 0 | 0 | 0 | 0 | 0 |  | 39.52 | 55.48 | 4.98 | 0.02 | 0 | 0 | 0 | 0 | 0 | 0 |  | 100 | 0 |
| 94 | 23.35 | 62.88 | 13.58 | 0.18 | 0 | 0 | 0 | 0 | 0 | 0 |  | 39.52 | 55.48 | 4.98 | 0.02 | 0 | 0 | 0 | 0 | 0 | 0 |  | 100 | 0 |
| 95 | 23.35 | 62.88 | 13.58 | 0.18 | 0 | 0 | 0 | 0 | 0 | 0 |  | 39.52 | 55.48 | 4.98 | 0.02 | 0 | 0 | 0 | 0 | 0 | 0 |  | 100 | 0 |

^a^ Rounded to two decimal points

^b^ %E = Percent of total energy intake
