# Supplementary material for: Substituting polyunsaturated fat for saturated fat: A health impact assessment of a fat tax in seven European countries
Source: PLoS One. 2019 Jul 10;14(7):e0218464. doi: 10.1371/journal.pone.0218464 (PMC6619676; doi:10.1371/journal.pone.0218464)
Supplement: S15 Table — (DOCX) [file pone.0218464.s015.docx]

# S15 Table. Proportion of persons in the respective saturated fat intake categories across scenarios in Sweden.

| Age | Reference scenario^a^ | | | | | | | | | |  | Fat tax scenario^a^ | | | | | | | | | |  | Guideline scenario | |
| --- | --- | --- | --- | --- | --- | --- | --- | --- | --- | --- | --- | --- | --- | --- | --- | --- | --- | --- | --- | --- | --- | --- | --- | --- |
|  | Category of saturated fat intake (in %E)^b^ | | | | | | | | | |  | Category of saturated fat intake (in %E)^b^ | | | | | | | | | |  | Category of saturated fat intake (in %E)^b^ | |
|  | ≤10 | >10 ≤12 | >12 ≤14 | >14 ≤16 | >16 ≤18 | >18 ≤20 | >20 ≤22 | >22 ≤24 | >24 ≤26 | >26 ≤100 |  | ≤10 | >10 ≤12 | >12 ≤14 | >14 ≤16 | >16 ≤18 | >18 ≤20 | >20 ≤22 | >22 ≤24 | >24 ≤26 | >26 ≤100 |  | ≤10 | >10 ≤100 |
|  |  |  | Males | | | | | | | | | | | | | | | | | | | | | |
| 0 | 100 | 0 | 0 | 0 | 0 | 0 | 0 | 0 | 0 | 0 |  | 100 | 0 | 0 | 0 | 0 | 0 | 0 | 0 | 0 | 0 |  | 100 | 0 |
| 1 | 100 | 0 | 0 | 0 | 0 | 0 | 0 | 0 | 0 | 0 |  | 100 | 0 | 0 | 0 | 0 | 0 | 0 | 0 | 0 | 0 |  | 100 | 0 |
| 2 | 100 | 0 | 0 | 0 | 0 | 0 | 0 | 0 | 0 | 0 |  | 100 | 0 | 0 | 0 | 0 | 0 | 0 | 0 | 0 | 0 |  | 100 | 0 |
| 3 | 100 | 0 | 0 | 0 | 0 | 0 | 0 | 0 | 0 | 0 |  | 100 | 0 | 0 | 0 | 0 | 0 | 0 | 0 | 0 | 0 |  | 100 | 0 |
| 4 | 100 | 0 | 0 | 0 | 0 | 0 | 0 | 0 | 0 | 0 |  | 100 | 0 | 0 | 0 | 0 | 0 | 0 | 0 | 0 | 0 |  | 100 | 0 |
| 5 | 100 | 0 | 0 | 0 | 0 | 0 | 0 | 0 | 0 | 0 |  | 100 | 0 | 0 | 0 | 0 | 0 | 0 | 0 | 0 | 0 |  | 100 | 0 |
| 6 | 3.68 | 13.65 | 28.95 | 31.19 | 17.08 | 4.74 | 0.67 | 0.05 | 0 | 0 |  | 3.68 | 13.65 | 28.95 | 31.19 | 17.08 | 4.74 | 0.67 | 0.05 | 0 | 0 |  | 100 | 0 |
| 7 | 3.75 | 13.96 | 29.41 | 31.17 | 16.62 | 4.45 | 0.6 | 0.04 | 0 | 0 |  | 3.75 | 13.96 | 29.41 | 31.17 | 16.62 | 4.45 | 0.6 | 0.04 | 0 | 0 |  | 100 | 0 |
| 8 | 3.84 | 14.25 | 29.73 | 31.07 | 16.26 | 4.26 | 0.56 | 0.04 | 0 | 0 |  | 3.84 | 14.25 | 29.73 | 31.07 | 16.26 | 4.26 | 0.56 | 0.04 | 0 | 0 |  | 100 | 0 |
| 9 | 3.98 | 14.48 | 29.83 | 30.88 | 16.06 | 4.19 | 0.55 | 0.03 | 0 | 0 |  | 3.98 | 14.48 | 29.83 | 30.88 | 16.06 | 4.19 | 0.55 | 0.03 | 0 | 0 |  | 100 | 0 |
| 10 | 4.13 | 14.65 | 29.78 | 30.65 | 15.98 | 4.21 | 0.56 | 0.04 | 0 | 0 |  | 4.13 | 14.65 | 29.78 | 30.65 | 15.98 | 4.21 | 0.56 | 0.04 | 0 | 0 |  | 100 | 0 |
| 11 | 4.25 | 14.73 | 29.62 | 30.45 | 16.01 | 4.3 | 0.59 | 0.04 | 0 | 0 |  | 4.25 | 14.73 | 29.62 | 30.45 | 16.01 | 4.3 | 0.59 | 0.04 | 0 | 0 |  | 100 | 0 |
| 12 | 4.34 | 14.72 | 29.39 | 30.3 | 16.14 | 4.43 | 0.62 | 0.04 | 0 | 0 |  | 4.34 | 14.72 | 29.39 | 30.3 | 16.14 | 4.43 | 0.62 | 0.04 | 0 | 0 |  | 100 | 0 |
| 13 | 4.39 | 14.61 | 29.05 | 30.18 | 16.38 | 4.64 | 0.68 | 0.05 | 0 | 0 |  | 4.39 | 14.61 | 29.05 | 30.18 | 16.38 | 4.64 | 0.68 | 0.05 | 0 | 0 |  | 100 | 0 |
| 14 | 4.42 | 14.36 | 28.51 | 30.04 | 16.82 | 4.99 | 0.78 | 0.06 | 0 | 0 |  | 4.42 | 14.36 | 28.51 | 30.04 | 16.82 | 4.99 | 0.78 | 0.06 | 0 | 0 |  | 100 | 0 |
| 15 | 4.44 | 14.02 | 27.8 | 29.87 | 17.38 | 5.47 | 0.93 | 0.08 | 0 | 0 |  | 6.63 | 18.83 | 31.83 | 27.5 | 12.14 | 2.73 | 0.31 | 0.02 | 0 | 0 |  | 100 | 0 |
| 16 | 4.44 | 13.64 | 27.03 | 29.64 | 17.98 | 6.03 | 1.12 | 0.11 | 0.01 | 0 |  | 6.57 | 18.25 | 31.06 | 27.67 | 12.89 | 3.13 | 0.4 | 0.03 | 0 | 0 |  | 100 | 0 |
| 17 | 4.44 | 13.26 | 26.26 | 29.38 | 18.56 | 6.62 | 1.33 | 0.15 | 0.01 | 0 |  | 6.51 | 17.67 | 30.29 | 27.78 | 13.63 | 3.57 | 0.5 | 0.04 | 0 | 0 |  | 100 | 0 |
| 18 | 4.43 | 12.91 | 25.57 | 29.11 | 19.06 | 7.17 | 1.55 | 0.19 | 0.01 | 0 |  | 6.45 | 17.16 | 29.58 | 27.84 | 14.3 | 4 | 0.61 | 0.05 | 0 | 0 |  | 100 | 0 |
| 19 | 4.43 | 12.64 | 25.01 | 28.86 | 19.43 | 7.63 | 1.75 | 0.23 | 0.02 | 0 |  | 6.44 | 16.81 | 29.05 | 27.82 | 14.76 | 4.34 | 0.7 | 0.06 | 0 | 0 |  | 100 | 0 |
| 20 | 4.43 | 12.46 | 24.63 | 28.68 | 19.68 | 7.95 | 1.89 | 0.26 | 0.02 | 0 |  | 6.36 | 16.45 | 28.58 | 27.86 | 15.22 | 4.66 | 0.8 | 0.08 | 0 | 0 |  | 100 | 0 |
| 21 | 4.42 | 12.36 | 24.43 | 28.59 | 19.81 | 8.12 | 1.97 | 0.28 | 0.02 | 0 |  | 6.34 | 16.3 | 28.37 | 27.84 | 15.41 | 4.81 | 0.84 | 0.08 | 0 | 0 |  | 100 | 0 |
| 22 | 4.42 | 12.32 | 24.35 | 28.54 | 19.86 | 8.19 | 2 | 0.29 | 0.02 | 0 |  | 6.33 | 16.24 | 28.28 | 27.84 | 15.49 | 4.87 | 0.86 | 0.09 | 0 | 0 |  | 100 | 0 |
| 23 | 4.42 | 12.31 | 24.33 | 28.54 | 19.87 | 8.2 | 2.01 | 0.29 | 0.02 | 0 |  | 6.33 | 16.23 | 28.27 | 27.84 | 15.5 | 4.88 | 0.86 | 0.09 | 0 | 0 |  | 100 | 0 |
| 24 | 4.42 | 12.32 | 24.35 | 28.55 | 19.85 | 8.19 | 2 | 0.29 | 0.02 | 0 |  | 6.33 | 16.24 | 28.29 | 27.84 | 15.48 | 4.86 | 0.86 | 0.09 | 0 | 0 |  | 100 | 0 |
| 25 | 4.42 | 12.33 | 24.38 | 28.56 | 19.84 | 8.16 | 1.99 | 0.29 | 0.02 | 0 |  | 6.28 | 16.16 | 28.23 | 27.88 | 15.57 | 4.91 | 0.87 | 0.09 | 0 | 0 |  | 100 | 0 |
| 26 | 4.42 | 12.35 | 24.4 | 28.58 | 19.82 | 8.14 | 1.98 | 0.28 | 0.02 | 0 |  | 6.28 | 16.18 | 28.26 | 27.89 | 15.55 | 4.89 | 0.87 | 0.09 | 0 | 0 |  | 100 | 0 |
| 27 | 4.42 | 12.35 | 24.42 | 28.58 | 19.81 | 8.13 | 1.97 | 0.28 | 0.02 | 0 |  | 6.28 | 16.19 | 28.27 | 27.89 | 15.53 | 4.88 | 0.86 | 0.09 | 0 | 0 |  | 100 | 0 |
| 28 | 4.42 | 12.36 | 24.43 | 28.59 | 19.81 | 8.12 | 1.97 | 0.28 | 0.02 | 0 |  | 6.28 | 16.2 | 28.28 | 27.89 | 15.52 | 4.87 | 0.86 | 0.09 | 0 | 0 |  | 100 | 0 |
| 29 | 4.42 | 12.36 | 24.43 | 28.59 | 19.81 | 8.12 | 1.97 | 0.28 | 0.02 | 0 |  | 6.29 | 16.2 | 28.29 | 27.89 | 15.52 | 4.87 | 0.86 | 0.08 | 0 | 0 |  | 100 | 0 |
| 30 | 4.42 | 12.36 | 24.43 | 28.59 | 19.8 | 8.12 | 1.97 | 0.28 | 0.02 | 0 |  | 6.23 | 16.1 | 28.2 | 27.93 | 15.63 | 4.94 | 0.88 | 0.09 | 0 | 0 |  | 100 | 0 |
| 31 | 4.42 | 12.36 | 24.43 | 28.59 | 19.81 | 8.12 | 1.97 | 0.28 | 0.02 | 0 |  | 6.23 | 16.1 | 28.2 | 27.93 | 15.63 | 4.94 | 0.88 | 0.09 | 0 | 0 |  | 100 | 0 |
| 32 | 4.42 | 12.36 | 24.43 | 28.59 | 19.81 | 8.12 | 1.97 | 0.28 | 0.02 | 0 |  | 6.23 | 16.1 | 28.2 | 27.93 | 15.63 | 4.94 | 0.88 | 0.09 | 0 | 0 |  | 100 | 0 |
| 33 | 4.42 | 12.36 | 24.43 | 28.59 | 19.81 | 8.12 | 1.97 | 0.28 | 0.02 | 0 |  | 6.23 | 16.1 | 28.2 | 27.93 | 15.63 | 4.94 | 0.88 | 0.09 | 0 | 0 |  | 100 | 0 |
| 34 | 4.42 | 12.36 | 24.43 | 28.59 | 19.81 | 8.12 | 1.97 | 0.28 | 0.02 | 0 |  | 6.23 | 16.09 | 28.2 | 27.93 | 15.63 | 4.94 | 0.88 | 0.09 | 0 | 0 |  | 100 | 0 |
| 35 | 4.42 | 12.36 | 24.43 | 28.59 | 19.81 | 8.12 | 1.97 | 0.28 | 0.02 | 0 |  | 6.23 | 16.09 | 28.19 | 27.93 | 15.64 | 4.95 | 0.88 | 0.09 | 0 | 0 |  | 100 | 0 |
| 36 | 4.42 | 12.36 | 24.43 | 28.59 | 19.81 | 8.12 | 1.97 | 0.28 | 0.02 | 0 |  | 6.23 | 16.09 | 28.19 | 27.93 | 15.64 | 4.95 | 0.88 | 0.09 | 0 | 0 |  | 100 | 0 |
| 37 | 4.42 | 12.36 | 24.43 | 28.59 | 19.81 | 8.12 | 1.97 | 0.28 | 0.02 | 0 |  | 6.23 | 16.09 | 28.19 | 27.93 | 15.64 | 4.95 | 0.88 | 0.09 | 0 | 0 |  | 100 | 0 |
| 38 | 4.42 | 12.36 | 24.43 | 28.59 | 19.81 | 8.12 | 1.97 | 0.28 | 0.02 | 0 |  | 6.23 | 16.09 | 28.19 | 27.93 | 15.64 | 4.95 | 0.88 | 0.09 | 0 | 0 |  | 100 | 0 |
| 39 | 4.42 | 12.36 | 24.43 | 28.59 | 19.81 | 8.12 | 1.97 | 0.28 | 0.02 | 0 |  | 6.23 | 16.09 | 28.19 | 27.93 | 15.64 | 4.95 | 0.88 | 0.09 | 0 | 0 |  | 100 | 0 |
| 40 | 4.42 | 12.36 | 24.43 | 28.59 | 19.81 | 8.12 | 1.97 | 0.28 | 0.02 | 0 |  | 6.18 | 15.99 | 28.11 | 27.97 | 15.75 | 5.01 | 0.9 | 0.09 | 0 | 0 |  | 100 | 0 |
| 41 | 4.42 | 12.36 | 24.43 | 28.59 | 19.81 | 8.12 | 1.97 | 0.28 | 0.02 | 0 |  | 6.18 | 15.99 | 28.11 | 27.97 | 15.75 | 5.01 | 0.9 | 0.09 | 0 | 0 |  | 100 | 0 |
| 42 | 4.42 | 12.36 | 24.43 | 28.59 | 19.81 | 8.12 | 1.97 | 0.28 | 0.02 | 0 |  | 6.18 | 15.99 | 28.11 | 27.97 | 15.75 | 5.01 | 0.9 | 0.09 | 0 | 0 |  | 100 | 0 |
| 43 | 4.42 | 12.36 | 24.43 | 28.59 | 19.81 | 8.12 | 1.97 | 0.28 | 0.02 | 0 |  | 6.18 | 15.99 | 28.11 | 27.97 | 15.75 | 5.01 | 0.9 | 0.09 | 0 | 0 |  | 100 | 0 |
| 44 | 4.42 | 12.36 | 24.43 | 28.59 | 19.81 | 8.12 | 1.97 | 0.28 | 0.02 | 0 |  | 6.18 | 15.99 | 28.11 | 27.97 | 15.75 | 5.01 | 0.9 | 0.09 | 0 | 0 |  | 100 | 0 |
| 45 | 4.42 | 12.36 | 24.43 | 28.59 | 19.81 | 8.12 | 1.97 | 0.28 | 0.02 | 0 |  | 6.17 | 15.98 | 28.1 | 27.97 | 15.75 | 5.02 | 0.9 | 0.09 | 0 | 0 |  | 100 | 0 |
| 46 | 4.42 | 12.36 | 24.43 | 28.59 | 19.81 | 8.12 | 1.97 | 0.28 | 0.02 | 0 |  | 6.17 | 15.98 | 28.1 | 27.97 | 15.75 | 5.02 | 0.9 | 0.09 | 0 | 0 |  | 100 | 0 |
| 47 | 4.42 | 12.36 | 24.43 | 28.59 | 19.81 | 8.12 | 1.97 | 0.28 | 0.02 | 0 |  | 6.17 | 15.98 | 28.1 | 27.97 | 15.75 | 5.02 | 0.9 | 0.09 | 0 | 0 |  | 100 | 0 |
| 48 | 4.42 | 12.36 | 24.43 | 28.59 | 19.81 | 8.12 | 1.97 | 0.28 | 0.02 | 0 |  | 6.17 | 15.98 | 28.1 | 27.97 | 15.75 | 5.02 | 0.9 | 0.09 | 0 | 0 |  | 100 | 0 |
| 49 | 4.42 | 12.36 | 24.43 | 28.59 | 19.81 | 8.12 | 1.97 | 0.28 | 0.02 | 0 |  | 6.17 | 15.98 | 28.1 | 27.97 | 15.76 | 5.02 | 0.9 | 0.09 | 0 | 0 |  | 100 | 0 |
| 50 | 4.42 | 12.36 | 24.43 | 28.59 | 19.81 | 8.12 | 1.97 | 0.28 | 0.02 | 0 |  | 6.08 | 15.8 | 27.94 | 28.03 | 15.96 | 5.15 | 0.94 | 0.1 | 0.01 | 0 |  | 100 | 0 |
| 51 | 4.42 | 12.36 | 24.42 | 28.58 | 19.81 | 8.13 | 1.97 | 0.28 | 0.02 | 0 |  | 6.08 | 15.8 | 27.94 | 28.03 | 15.96 | 5.15 | 0.94 | 0.1 | 0.01 | 0 |  | 100 | 0 |
| 52 | 4.42 | 12.35 | 24.42 | 28.58 | 19.81 | 8.13 | 1.97 | 0.28 | 0.02 | 0 |  | 6.08 | 15.8 | 27.93 | 28.03 | 15.96 | 5.15 | 0.94 | 0.1 | 0.01 | 0 |  | 100 | 0 |
| 53 | 4.42 | 12.35 | 24.42 | 28.58 | 19.81 | 8.13 | 1.97 | 0.28 | 0.02 | 0 |  | 6.08 | 15.8 | 27.93 | 28.03 | 15.96 | 5.15 | 0.94 | 0.1 | 0.01 | 0 |  | 100 | 0 |
| 54 | 4.42 | 12.36 | 24.43 | 28.59 | 19.81 | 8.12 | 1.97 | 0.28 | 0.02 | 0 |  | 6.08 | 15.8 | 27.94 | 28.04 | 15.96 | 5.15 | 0.94 | 0.1 | 0.01 | 0 |  | 100 | 0 |
| 55 | 4.42 | 12.36 | 24.44 | 28.6 | 19.8 | 8.11 | 1.96 | 0.28 | 0.02 | 0 |  | 6.07 | 15.8 | 27.95 | 28.05 | 15.96 | 5.14 | 0.94 | 0.1 | 0.01 | 0 |  | 100 | 0 |
| 56 | 4.41 | 12.36 | 24.47 | 28.62 | 19.79 | 8.09 | 1.95 | 0.28 | 0.02 | 0 |  | 6.06 | 15.81 | 27.98 | 28.06 | 15.94 | 5.12 | 0.93 | 0.1 | 0 | 0 |  | 100 | 0 |
| 57 | 4.4 | 12.37 | 24.51 | 28.66 | 19.78 | 8.05 | 1.93 | 0.27 | 0.02 | 0 |  | 6.05 | 15.82 | 28.03 | 28.08 | 15.91 | 5.09 | 0.92 | 0.09 | 0 | 0 |  | 100 | 0 |
| 58 | 4.38 | 12.38 | 24.56 | 28.7 | 19.76 | 8.01 | 1.91 | 0.27 | 0.02 | 0 |  | 6.03 | 15.84 | 28.09 | 28.11 | 15.87 | 5.05 | 0.91 | 0.09 | 0 | 0 |  | 100 | 0 |
| 59 | 4.37 | 12.38 | 24.59 | 28.73 | 19.75 | 7.99 | 1.9 | 0.26 | 0.02 | 0 |  | 6.02 | 15.85 | 28.13 | 28.13 | 15.85 | 5.03 | 0.9 | 0.09 | 0 | 0 |  | 100 | 0 |
| 60 | 4.38 | 12.38 | 24.57 | 28.72 | 19.76 | 8 | 1.9 | 0.27 | 0.02 | 0 |  | 5.98 | 15.75 | 28.03 | 28.15 | 15.96 | 5.11 | 0.92 | 0.09 | 0 | 0 |  | 100 | 0 |
| 61 | 4.42 | 12.36 | 24.44 | 28.6 | 19.8 | 8.11 | 1.96 | 0.28 | 0.02 | 0 |  | 6.02 | 15.7 | 27.87 | 28.08 | 16.06 | 5.21 | 0.96 | 0.1 | 0.01 | 0 |  | 100 | 0 |
| 62 | 4.52 | 12.3 | 24.11 | 28.3 | 19.91 | 8.39 | 2.12 | 0.32 | 0.03 | 0 |  | 6.13 | 15.57 | 27.47 | 27.89 | 16.29 | 5.47 | 1.05 | 0.12 | 0.01 | 0 |  | 100 | 0 |
| 63 | 4.72 | 12.19 | 23.5 | 27.75 | 20.08 | 8.9 | 2.41 | 0.4 | 0.04 | 0 |  | 6.33 | 15.33 | 26.76 | 27.52 | 16.68 | 5.96 | 1.25 | 0.15 | 0.01 | 0 |  | 100 | 0 |
| 64 | 5 | 12.03 | 22.67 | 26.97 | 20.25 | 9.6 | 2.87 | 0.54 | 0.06 | 0 |  | 6.61 | 15 | 25.77 | 26.96 | 17.18 | 6.66 | 1.57 | 0.22 | 0.02 | 0 |  | 100 | 0 |
| 65 | 5.34 | 11.84 | 21.75 | 26.05 | 20.35 | 10.37 | 3.44 | 0.74 | 0.1 | 0.01 |  | 6.88 | 14.5 | 24.57 | 26.29 | 17.77 | 7.58 | 2.04 | 0.35 | 0.04 | 0 |  | 100 | 0 |
| 66 | 5.62 | 11.68 | 21.01 | 25.29 | 20.36 | 10.96 | 3.95 | 0.95 | 0.15 | 0.02 |  | 7.16 | 14.19 | 23.7 | 25.68 | 18.06 | 8.24 | 2.44 | 0.47 | 0.06 | 0 |  | 100 | 0 |
| 67 | 5.81 | 11.57 | 20.55 | 24.79 | 20.33 | 11.33 | 4.29 | 1.1 | 0.19 | 0.02 |  | 7.35 | 14 | 23.14 | 25.26 | 18.21 | 8.67 | 2.72 | 0.56 | 0.08 | 0.01 |  | 100 | 0 |
| 68 | 5.91 | 11.51 | 20.3 | 24.52 | 20.31 | 11.52 | 4.48 | 1.19 | 0.22 | 0.03 |  | 7.45 | 13.89 | 22.85 | 25.04 | 18.28 | 8.89 | 2.88 | 0.62 | 0.09 | 0.01 |  | 100 | 0 |
| 69 | 5.95 | 11.49 | 20.2 | 24.42 | 20.29 | 11.6 | 4.56 | 1.23 | 0.23 | 0.03 |  | 7.49 | 13.85 | 22.73 | 24.95 | 18.31 | 8.98 | 2.94 | 0.64 | 0.09 | 0.01 |  | 100 | 0 |
| 70 | 5.96 | 11.49 | 20.19 | 24.4 | 20.29 | 11.61 | 4.57 | 1.23 | 0.23 | 0.03 |  | 7.45 | 13.78 | 22.65 | 24.93 | 18.37 | 9.06 | 2.99 | 0.66 | 0.1 | 0.01 |  | 100 | 0 |
| 71 | 5.95 | 11.49 | 20.21 | 24.43 | 20.3 | 11.59 | 4.55 | 1.23 | 0.23 | 0.03 |  | 7.44 | 13.79 | 22.68 | 24.96 | 18.37 | 9.04 | 2.97 | 0.65 | 0.1 | 0.01 |  | 100 | 0 |
| 72 | 5.93 | 11.5 | 20.25 | 24.47 | 20.3 | 11.56 | 4.52 | 1.21 | 0.22 | 0.03 |  | 7.42 | 13.8 | 22.73 | 24.99 | 18.36 | 9.01 | 2.95 | 0.64 | 0.09 | 0.01 |  | 100 | 0 |
| 73 | 5.92 | 11.51 | 20.29 | 24.51 | 20.3 | 11.53 | 4.49 | 1.2 | 0.22 | 0.03 |  | 7.41 | 13.82 | 22.77 | 25.02 | 18.35 | 8.97 | 2.92 | 0.64 | 0.09 | 0.01 |  | 100 | 0 |
| 74 | 5.9 | 11.52 | 20.32 | 24.55 | 20.31 | 11.51 | 4.46 | 1.18 | 0.22 | 0.03 |  | 7.39 | 13.83 | 22.81 | 25.06 | 18.34 | 8.94 | 2.9 | 0.63 | 0.09 | 0.01 |  | 100 | 0 |
| 75 | 5.89 | 11.53 | 20.36 | 24.59 | 20.31 | 11.48 | 4.44 | 1.17 | 0.21 | 0.03 |  | 7.33 | 13.78 | 22.79 | 25.09 | 18.39 | 8.97 | 2.92 | 0.63 | 0.09 | 0.01 |  | 100 | 0 |
| 76 | 5.87 | 11.54 | 20.39 | 24.63 | 20.32 | 11.45 | 4.41 | 1.16 | 0.21 | 0.03 |  | 7.32 | 13.8 | 22.83 | 25.12 | 18.38 | 8.94 | 2.89 | 0.62 | 0.09 | 0.01 |  | 100 | 0 |
| 77 | 5.86 | 11.54 | 20.43 | 24.67 | 20.32 | 11.42 | 4.38 | 1.15 | 0.2 | 0.03 |  | 7.3 | 13.81 | 22.87 | 25.15 | 18.37 | 8.91 | 2.87 | 0.61 | 0.09 | 0.01 |  | 100 | 0 |
| 78 | 5.84 | 11.55 | 20.46 | 24.7 | 20.32 | 11.4 | 4.35 | 1.13 | 0.2 | 0.03 |  | 7.29 | 13.83 | 22.91 | 25.19 | 18.36 | 8.88 | 2.84 | 0.6 | 0.08 | 0.01 |  | 100 | 0 |
| 79 | 5.83 | 11.56 | 20.5 | 24.74 | 20.33 | 11.37 | 4.33 | 1.12 | 0.2 | 0.03 |  | 7.27 | 13.84 | 22.96 | 25.22 | 18.35 | 8.85 | 2.82 | 0.6 | 0.08 | 0.01 |  | 100 | 0 |
| 80 | 5.81 | 11.57 | 20.54 | 24.78 | 20.33 | 11.34 | 4.3 | 1.11 | 0.19 | 0.02 |  | 7.22 | 13.79 | 22.93 | 25.25 | 18.4 | 8.88 | 2.83 | 0.6 | 0.08 | 0.01 |  | 100 | 0 |
| 81 | 5.8 | 11.58 | 20.57 | 24.82 | 20.34 | 11.31 | 4.27 | 1.09 | 0.19 | 0.02 |  | 7.2 | 13.81 | 22.98 | 25.28 | 18.39 | 8.85 | 2.81 | 0.59 | 0.08 | 0.01 |  | 100 | 0 |
| 82 | 5.78 | 11.59 | 20.61 | 24.86 | 20.34 | 11.28 | 4.25 | 1.08 | 0.19 | 0.02 |  | 7.19 | 13.82 | 23.02 | 25.32 | 18.38 | 8.81 | 2.79 | 0.58 | 0.08 | 0.01 |  | 100 | 0 |
| 83 | 5.77 | 11.6 | 20.65 | 24.9 | 20.34 | 11.26 | 4.22 | 1.07 | 0.18 | 0.02 |  | 7.17 | 13.84 | 23.06 | 25.35 | 18.37 | 8.78 | 2.77 | 0.57 | 0.08 | 0.01 |  | 100 | 0 |
| 84 | 5.75 | 11.6 | 20.68 | 24.94 | 20.34 | 11.23 | 4.19 | 1.06 | 0.18 | 0.02 |  | 7.16 | 13.85 | 23.11 | 25.38 | 18.36 | 8.75 | 2.74 | 0.56 | 0.08 | 0.01 |  | 100 | 0 |
| 85 | 5.74 | 11.61 | 20.72 | 24.98 | 20.35 | 11.2 | 4.16 | 1.04 | 0.18 | 0.02 |  | 7.06 | 13.74 | 23.02 | 25.41 | 18.47 | 8.85 | 2.79 | 0.58 | 0.08 | 0.01 |  | 100 | 0 |
| 86 | 5.72 | 11.62 | 20.76 | 25.02 | 20.35 | 11.17 | 4.14 | 1.03 | 0.17 | 0.02 |  | 7.04 | 13.75 | 23.06 | 25.44 | 18.46 | 8.81 | 2.77 | 0.57 | 0.08 | 0.01 |  | 100 | 0 |
| 87 | 5.71 | 11.63 | 20.79 | 25.06 | 20.35 | 11.14 | 4.11 | 1.02 | 0.17 | 0.02 |  | 7.03 | 13.77 | 23.11 | 25.48 | 18.45 | 8.78 | 2.74 | 0.56 | 0.08 | 0.01 |  | 100 | 0 |
| 88 | 5.69 | 11.64 | 20.83 | 25.09 | 20.35 | 11.11 | 4.08 | 1.01 | 0.17 | 0.02 |  | 7.02 | 13.78 | 23.15 | 25.51 | 18.44 | 8.75 | 2.72 | 0.55 | 0.07 | 0.01 |  | 100 | 0 |
| 89 | 5.68 | 11.65 | 20.87 | 25.13 | 20.36 | 11.08 | 4.06 | 1 | 0.16 | 0.02 |  | 7 | 13.8 | 23.19 | 25.54 | 18.43 | 8.71 | 2.7 | 0.55 | 0.07 | 0.01 |  | 100 | 0 |
| 90 | 5.66 | 11.65 | 20.9 | 25.17 | 20.36 | 11.05 | 4.03 | 0.98 | 0.16 | 0.02 |  | 6.99 | 13.81 | 23.24 | 25.58 | 18.42 | 8.68 | 2.67 | 0.54 | 0.07 | 0.01 |  | 100 | 0 |
| 91 | 5.65 | 11.66 | 20.94 | 25.21 | 20.36 | 11.02 | 4 | 0.97 | 0.16 | 0.02 |  | 6.97 | 13.83 | 23.28 | 25.61 | 18.41 | 8.64 | 2.65 | 0.53 | 0.07 | 0.01 |  | 100 | 0 |
| 92 | 5.63 | 11.67 | 20.98 | 25.25 | 20.36 | 10.99 | 3.97 | 0.96 | 0.16 | 0.02 |  | 6.96 | 13.84 | 23.32 | 25.64 | 18.4 | 8.61 | 2.63 | 0.52 | 0.07 | 0.01 |  | 100 | 0 |
| 93 | 5.62 | 11.68 | 21.02 | 25.29 | 20.36 | 10.96 | 3.95 | 0.95 | 0.15 | 0.02 |  | 6.94 | 13.86 | 23.37 | 25.68 | 18.39 | 8.58 | 2.6 | 0.52 | 0.07 | 0.01 |  | 100 | 0 |
| 94 | 5.6 | 11.69 | 21.05 | 25.33 | 20.36 | 10.93 | 3.92 | 0.94 | 0.15 | 0.02 |  | 6.93 | 13.87 | 23.41 | 25.71 | 18.38 | 8.54 | 2.58 | 0.51 | 0.06 | 0.01 |  | 100 | 0 |
| 95 | 5.59 | 11.7 | 21.09 | 25.37 | 20.36 | 10.9 | 3.89 | 0.93 | 0.15 | 0.02 |  | 6.91 | 13.88 | 23.46 | 25.75 | 18.36 | 8.51 | 2.56 | 0.5 | 0.06 | 0 |  | 100 | 0 |
|  | |  | Females | | | | | | | | | | | | | | | | | | | | | |
| 0 | 100 | 0 | 0 | 0 | 0 | 0 | 0 | 0 | 0 | 0 |  | 100 | 0 | 0 | 0 | 0 | 0 | 0 | 0 | 0 | 0 |  | 100 | 0 |
| 1 | 100 | 0 | 0 | 0 | 0 | 0 | 0 | 0 | 0 | 0 |  | 100 | 0 | 0 | 0 | 0 | 0 | 0 | 0 | 0 | 0 |  | 100 | 0 |
| 2 | 100 | 0 | 0 | 0 | 0 | 0 | 0 | 0 | 0 | 0 |  | 100 | 0 | 0 | 0 | 0 | 0 | 0 | 0 | 0 | 0 |  | 100 | 0 |
| 3 | 100 | 0 | 0 | 0 | 0 | 0 | 0 | 0 | 0 | 0 |  | 100 | 0 | 0 | 0 | 0 | 0 | 0 | 0 | 0 | 0 |  | 100 | 0 |
| 4 | 100 | 0 | 0 | 0 | 0 | 0 | 0 | 0 | 0 | 0 |  | 100 | 0 | 0 | 0 | 0 | 0 | 0 | 0 | 0 | 0 |  | 100 | 0 |
| 5 | 100 | 0 | 0 | 0 | 0 | 0 | 0 | 0 | 0 | 0 |  | 100 | 0 | 0 | 0 | 0 | 0 | 0 | 0 | 0 | 0 |  | 100 | 0 |
| 6 | 3.44 | 12.59 | 27.34 | 31.14 | 18.62 | 5.83 | 0.95 | 0.08 | 0 | 0 |  | 3.44 | 12.59 | 27.34 | 31.14 | 18.62 | 5.83 | 0.95 | 0.08 | 0 | 0 |  | 100 | 0 |
| 7 | 3.58 | 13.32 | 28.54 | 31.26 | 17.51 | 5.01 | 0.73 | 0.05 | 0 | 0 |  | 3.58 | 13.32 | 28.54 | 31.26 | 17.51 | 5.01 | 0.73 | 0.05 | 0 | 0 |  | 100 | 0 |
| 8 | 3.73 | 14 | 29.54 | 31.22 | 16.53 | 4.37 | 0.58 | 0.04 | 0 | 0 |  | 3.73 | 14 | 29.54 | 31.22 | 16.53 | 4.37 | 0.58 | 0.04 | 0 | 0 |  | 100 | 0 |
| 9 | 3.87 | 14.54 | 30.25 | 31.09 | 15.79 | 3.95 | 0.49 | 0.03 | 0 | 0 |  | 3.87 | 14.54 | 30.25 | 31.09 | 15.79 | 3.95 | 0.49 | 0.03 | 0 | 0 |  | 100 | 0 |
| 10 | 3.99 | 14.92 | 30.67 | 30.94 | 15.31 | 3.71 | 0.44 | 0.02 | 0 | 0 |  | 3.99 | 14.92 | 30.67 | 30.94 | 15.31 | 3.71 | 0.44 | 0.02 | 0 | 0 |  | 100 | 0 |
| 11 | 4.07 | 15.14 | 30.88 | 30.82 | 15.06 | 3.59 | 0.42 | 0.02 | 0 | 0 |  | 4.07 | 15.14 | 30.88 | 30.82 | 15.06 | 3.59 | 0.42 | 0.02 | 0 | 0 |  | 100 | 0 |
| 12 | 4.1 | 15.2 | 30.92 | 30.78 | 15 | 3.57 | 0.41 | 0.02 | 0 | 0 |  | 4.1 | 15.2 | 30.92 | 30.78 | 15 | 3.57 | 0.41 | 0.02 | 0 | 0 |  | 100 | 0 |
| 13 | 4.07 | 15.12 | 30.84 | 30.82 | 15.09 | 3.61 | 0.42 | 0.02 | 0 | 0 |  | 4.07 | 15.12 | 30.84 | 30.82 | 15.09 | 3.61 | 0.42 | 0.02 | 0 | 0 |  | 100 | 0 |
| 14 | 3.98 | 14.9 | 30.67 | 30.95 | 15.32 | 3.71 | 0.44 | 0.02 | 0 | 0 |  | 3.98 | 14.9 | 30.67 | 30.95 | 15.32 | 3.71 | 0.44 | 0.02 | 0 | 0 |  | 100 | 0 |
| 15 | 3.84 | 14.58 | 30.42 | 31.15 | 15.67 | 3.86 | 0.46 | 0.03 | 0 | 0 |  | 5.14 | 18.03 | 33.38 | 29.06 | 11.89 | 2.28 | 0.2 | 0.01 | 0 | 0 |  | 100 | 0 |
| 16 | 3.67 | 14.19 | 30.12 | 31.39 | 16.07 | 4.03 | 0.49 | 0.03 | 0 | 0 |  | 4.92 | 17.6 | 33.16 | 29.41 | 12.28 | 2.4 | 0.22 | 0.01 | 0 | 0 |  | 100 | 0 |
| 17 | 3.5 | 13.8 | 29.8 | 31.63 | 16.5 | 4.22 | 0.53 | 0.03 | 0 | 0 |  | 4.7 | 17.14 | 32.92 | 29.78 | 12.68 | 2.53 | 0.24 | 0.01 | 0 | 0 |  | 100 | 0 |
| 18 | 3.34 | 13.42 | 29.49 | 31.84 | 16.91 | 4.4 | 0.56 | 0.03 | 0 | 0 |  | 4.49 | 16.72 | 32.69 | 30.11 | 13.06 | 2.66 | 0.25 | 0.01 | 0 | 0 |  | 100 | 0 |
| 19 | 3.21 | 13.12 | 29.23 | 32.02 | 17.24 | 4.56 | 0.59 | 0.04 | 0 | 0 |  | 4.39 | 16.53 | 32.62 | 30.28 | 13.21 | 2.7 | 0.26 | 0.01 | 0 | 0 |  | 100 | 0 |
| 20 | 3.12 | 12.91 | 29.05 | 32.13 | 17.48 | 4.66 | 0.61 | 0.04 | 0 | 0 |  | 4.27 | 16.29 | 32.48 | 30.46 | 13.43 | 2.78 | 0.27 | 0.01 | 0 | 0 |  | 100 | 0 |
| 21 | 3.08 | 12.79 | 28.95 | 32.2 | 17.61 | 4.72 | 0.62 | 0.04 | 0 | 0 |  | 4.21 | 16.16 | 32.4 | 30.57 | 13.56 | 2.82 | 0.27 | 0.01 | 0 | 0 |  | 100 | 0 |
| 22 | 3.06 | 12.74 | 28.9 | 32.22 | 17.66 | 4.75 | 0.62 | 0.04 | 0 | 0 |  | 4.19 | 16.1 | 32.37 | 30.61 | 13.61 | 2.84 | 0.27 | 0.01 | 0 | 0 |  | 100 | 0 |
| 23 | 3.06 | 12.73 | 28.9 | 32.23 | 17.67 | 4.75 | 0.62 | 0.04 | 0 | 0 |  | 4.18 | 16.09 | 32.37 | 30.61 | 13.62 | 2.84 | 0.28 | 0.01 | 0 | 0 |  | 100 | 0 |
| 24 | 3.06 | 12.75 | 28.91 | 32.22 | 17.66 | 4.75 | 0.62 | 0.04 | 0 | 0 |  | 4.19 | 16.11 | 32.38 | 30.61 | 13.6 | 2.83 | 0.27 | 0.01 | 0 | 0 |  | 100 | 0 |
| 25 | 3.07 | 12.76 | 28.92 | 32.21 | 17.64 | 4.74 | 0.62 | 0.04 | 0 | 0 |  | 4.23 | 16.23 | 32.48 | 30.52 | 13.47 | 2.78 | 0.27 | 0.01 | 0 | 0 |  | 100 | 0 |
| 26 | 3.07 | 12.77 | 28.93 | 32.2 | 17.62 | 4.73 | 0.62 | 0.04 | 0 | 0 |  | 4.24 | 16.25 | 32.49 | 30.51 | 13.46 | 2.78 | 0.27 | 0.01 | 0 | 0 |  | 100 | 0 |
| 27 | 3.08 | 12.78 | 28.94 | 32.2 | 17.61 | 4.73 | 0.62 | 0.04 | 0 | 0 |  | 4.25 | 16.26 | 32.5 | 30.5 | 13.44 | 2.77 | 0.27 | 0.01 | 0 | 0 |  | 100 | 0 |
| 28 | 3.08 | 12.79 | 28.95 | 32.2 | 17.61 | 4.72 | 0.62 | 0.04 | 0 | 0 |  | 4.25 | 16.26 | 32.5 | 30.5 | 13.44 | 2.77 | 0.27 | 0.01 | 0 | 0 |  | 100 | 0 |
| 29 | 3.08 | 12.79 | 28.95 | 32.2 | 17.61 | 4.72 | 0.62 | 0.04 | 0 | 0 |  | 4.25 | 16.27 | 32.5 | 30.5 | 13.44 | 2.77 | 0.27 | 0.01 | 0 | 0 |  | 100 | 0 |
| 30 | 3.08 | 12.79 | 28.95 | 32.2 | 17.61 | 4.72 | 0.62 | 0.04 | 0 | 0 |  | 4.33 | 16.49 | 32.69 | 30.35 | 13.2 | 2.68 | 0.25 | 0.01 | 0 | 0 |  | 100 | 0 |
| 31 | 3.08 | 12.79 | 28.95 | 32.2 | 17.61 | 4.72 | 0.62 | 0.04 | 0 | 0 |  | 4.33 | 16.49 | 32.69 | 30.35 | 13.2 | 2.68 | 0.25 | 0.01 | 0 | 0 |  | 100 | 0 |
| 32 | 3.08 | 12.79 | 28.95 | 32.2 | 17.61 | 4.72 | 0.62 | 0.04 | 0 | 0 |  | 4.33 | 16.48 | 32.69 | 30.36 | 13.2 | 2.68 | 0.25 | 0.01 | 0 | 0 |  | 100 | 0 |
| 33 | 3.08 | 12.79 | 28.95 | 32.2 | 17.61 | 4.72 | 0.62 | 0.04 | 0 | 0 |  | 4.33 | 16.48 | 32.69 | 30.36 | 13.2 | 2.68 | 0.25 | 0.01 | 0 | 0 |  | 100 | 0 |
| 34 | 3.08 | 12.79 | 28.95 | 32.2 | 17.61 | 4.72 | 0.62 | 0.04 | 0 | 0 |  | 4.33 | 16.48 | 32.69 | 30.36 | 13.2 | 2.68 | 0.25 | 0.01 | 0 | 0 |  | 100 | 0 |
| 35 | 3.08 | 12.79 | 28.95 | 32.2 | 17.61 | 4.72 | 0.62 | 0.04 | 0 | 0 |  | 4.33 | 16.48 | 32.69 | 30.36 | 13.2 | 2.68 | 0.25 | 0.01 | 0 | 0 |  | 100 | 0 |
| 36 | 3.08 | 12.79 | 28.95 | 32.2 | 17.61 | 4.72 | 0.62 | 0.04 | 0 | 0 |  | 4.33 | 16.48 | 32.69 | 30.36 | 13.2 | 2.68 | 0.25 | 0.01 | 0 | 0 |  | 100 | 0 |
| 37 | 3.08 | 12.79 | 28.95 | 32.2 | 17.61 | 4.72 | 0.62 | 0.04 | 0 | 0 |  | 4.33 | 16.48 | 32.69 | 30.36 | 13.2 | 2.68 | 0.25 | 0.01 | 0 | 0 |  | 100 | 0 |
| 38 | 3.08 | 12.79 | 28.95 | 32.2 | 17.61 | 4.72 | 0.62 | 0.04 | 0 | 0 |  | 4.33 | 16.48 | 32.69 | 30.36 | 13.2 | 2.68 | 0.25 | 0.01 | 0 | 0 |  | 100 | 0 |
| 39 | 3.08 | 12.79 | 28.95 | 32.2 | 17.61 | 4.72 | 0.62 | 0.04 | 0 | 0 |  | 4.33 | 16.48 | 32.69 | 30.36 | 13.2 | 2.68 | 0.25 | 0.01 | 0 | 0 |  | 100 | 0 |
| 40 | 3.08 | 12.79 | 28.95 | 32.2 | 17.61 | 4.72 | 0.62 | 0.04 | 0 | 0 |  | 4.41 | 16.71 | 32.87 | 30.21 | 12.96 | 2.59 | 0.24 | 0.01 | 0 | 0 |  | 100 | 0 |
| 41 | 3.08 | 12.79 | 28.95 | 32.2 | 17.61 | 4.72 | 0.62 | 0.04 | 0 | 0 |  | 4.41 | 16.71 | 32.87 | 30.21 | 12.96 | 2.59 | 0.24 | 0.01 | 0 | 0 |  | 100 | 0 |
| 42 | 3.08 | 12.79 | 28.95 | 32.2 | 17.61 | 4.72 | 0.62 | 0.04 | 0 | 0 |  | 4.41 | 16.71 | 32.87 | 30.21 | 12.96 | 2.59 | 0.24 | 0.01 | 0 | 0 |  | 100 | 0 |
| 43 | 3.08 | 12.79 | 28.95 | 32.2 | 17.61 | 4.72 | 0.62 | 0.04 | 0 | 0 |  | 4.41 | 16.71 | 32.87 | 30.21 | 12.96 | 2.59 | 0.24 | 0.01 | 0 | 0 |  | 100 | 0 |
| 44 | 3.08 | 12.79 | 28.95 | 32.2 | 17.61 | 4.72 | 0.62 | 0.04 | 0 | 0 |  | 4.41 | 16.71 | 32.87 | 30.21 | 12.96 | 2.59 | 0.24 | 0.01 | 0 | 0 |  | 100 | 0 |
| 45 | 3.08 | 12.79 | 28.95 | 32.2 | 17.61 | 4.72 | 0.62 | 0.04 | 0 | 0 |  | 4.41 | 16.71 | 32.87 | 30.21 | 12.96 | 2.59 | 0.24 | 0.01 | 0 | 0 |  | 100 | 0 |
| 46 | 3.08 | 12.79 | 28.95 | 32.2 | 17.61 | 4.72 | 0.62 | 0.04 | 0 | 0 |  | 4.41 | 16.71 | 32.87 | 30.21 | 12.96 | 2.59 | 0.24 | 0.01 | 0 | 0 |  | 100 | 0 |
| 47 | 3.08 | 12.79 | 28.95 | 32.2 | 17.61 | 4.72 | 0.62 | 0.04 | 0 | 0 |  | 4.41 | 16.71 | 32.87 | 30.21 | 12.96 | 2.59 | 0.24 | 0.01 | 0 | 0 |  | 100 | 0 |
| 48 | 3.08 | 12.79 | 28.95 | 32.2 | 17.61 | 4.72 | 0.62 | 0.04 | 0 | 0 |  | 4.41 | 16.71 | 32.87 | 30.21 | 12.96 | 2.59 | 0.24 | 0.01 | 0 | 0 |  | 100 | 0 |
| 49 | 3.08 | 12.79 | 28.95 | 32.2 | 17.61 | 4.72 | 0.62 | 0.04 | 0 | 0 |  | 4.41 | 16.71 | 32.87 | 30.21 | 12.96 | 2.59 | 0.24 | 0.01 | 0 | 0 |  | 100 | 0 |
| 50 | 3.08 | 12.79 | 28.95 | 32.2 | 17.61 | 4.72 | 0.62 | 0.04 | 0 | 0 |  | 4.45 | 16.82 | 32.96 | 30.14 | 12.85 | 2.54 | 0.23 | 0.01 | 0 | 0 |  | 100 | 0 |
| 51 | 3.08 | 12.79 | 28.95 | 32.2 | 17.61 | 4.72 | 0.62 | 0.04 | 0 | 0 |  | 4.45 | 16.82 | 32.96 | 30.14 | 12.85 | 2.54 | 0.23 | 0.01 | 0 | 0 |  | 100 | 0 |
| 52 | 3.08 | 12.79 | 28.95 | 32.2 | 17.61 | 4.72 | 0.62 | 0.04 | 0 | 0 |  | 4.45 | 16.82 | 32.96 | 30.14 | 12.85 | 2.54 | 0.23 | 0.01 | 0 | 0 |  | 100 | 0 |
| 53 | 3.08 | 12.79 | 28.95 | 32.2 | 17.61 | 4.72 | 0.62 | 0.04 | 0 | 0 |  | 4.45 | 16.82 | 32.96 | 30.14 | 12.85 | 2.54 | 0.23 | 0.01 | 0 | 0 |  | 100 | 0 |
| 54 | 3.08 | 12.79 | 28.95 | 32.2 | 17.61 | 4.72 | 0.62 | 0.04 | 0 | 0 |  | 4.45 | 16.82 | 32.96 | 30.14 | 12.85 | 2.54 | 0.23 | 0.01 | 0 | 0 |  | 100 | 0 |
| 55 | 3.08 | 12.79 | 28.94 | 32.19 | 17.61 | 4.73 | 0.62 | 0.04 | 0 | 0 |  | 4.5 | 16.93 | 33.05 | 30.05 | 12.73 | 2.5 | 0.23 | 0.01 | 0 | 0 |  | 100 | 0 |
| 56 | 3.08 | 12.79 | 28.94 | 32.19 | 17.61 | 4.73 | 0.62 | 0.04 | 0 | 0 |  | 4.5 | 16.93 | 33.05 | 30.05 | 12.73 | 2.5 | 0.23 | 0.01 | 0 | 0 |  | 100 | 0 |
| 57 | 3.09 | 12.8 | 28.93 | 32.18 | 17.61 | 4.73 | 0.62 | 0.04 | 0 | 0 |  | 4.51 | 16.94 | 33.03 | 30.04 | 12.73 | 2.51 | 0.23 | 0.01 | 0 | 0 |  | 100 | 0 |
| 58 | 3.09 | 12.8 | 28.92 | 32.17 | 17.61 | 4.74 | 0.62 | 0.04 | 0 | 0 |  | 4.51 | 16.94 | 33.02 | 30.04 | 12.74 | 2.51 | 0.23 | 0.01 | 0 | 0 |  | 100 | 0 |
| 59 | 3.1 | 12.8 | 28.92 | 32.16 | 17.61 | 4.74 | 0.62 | 0.04 | 0 | 0 |  | 4.52 | 16.94 | 33.02 | 30.03 | 12.74 | 2.51 | 0.23 | 0.01 | 0 | 0 |  | 100 | 0 |
| 60 | 3.09 | 12.8 | 28.92 | 32.16 | 17.61 | 4.74 | 0.62 | 0.04 | 0 | 0 |  | 4.56 | 17.05 | 33.11 | 29.96 | 12.63 | 2.47 | 0.22 | 0.01 | 0 | 0 |  | 100 | 0 |
| 61 | 3.08 | 12.79 | 28.94 | 32.2 | 17.61 | 4.72 | 0.62 | 0.04 | 0 | 0 |  | 4.54 | 17.04 | 33.14 | 29.98 | 12.61 | 2.46 | 0.22 | 0.01 | 0 | 0 |  | 100 | 0 |
| 62 | 3.04 | 12.75 | 29 | 32.28 | 17.59 | 4.69 | 0.61 | 0.04 | 0 | 0 |  | 4.49 | 17.02 | 33.22 | 30.04 | 12.57 | 2.43 | 0.22 | 0.01 | 0 | 0 |  | 100 | 0 |
| 63 | 2.97 | 12.69 | 29.11 | 32.44 | 17.56 | 4.61 | 0.58 | 0.04 | 0 | 0 |  | 4.4 | 16.98 | 33.38 | 30.15 | 12.5 | 2.37 | 0.2 | 0.01 | 0 | 0 |  | 100 | 0 |
| 64 | 2.86 | 12.59 | 29.27 | 32.67 | 17.52 | 4.5 | 0.55 | 0.03 | 0 | 0 |  | 4.27 | 16.92 | 33.62 | 30.31 | 12.4 | 2.29 | 0.19 | 0.01 | 0 | 0 |  | 100 | 0 |
| 65 | 2.74 | 12.47 | 29.46 | 32.95 | 17.46 | 4.38 | 0.52 | 0.03 | 0 | 0 |  | 4.16 | 16.99 | 34.01 | 30.4 | 12.12 | 2.15 | 0.17 | 0.01 | 0 | 0 |  | 100 | 0 |
| 66 | 2.64 | 12.37 | 29.62 | 33.19 | 17.41 | 4.26 | 0.48 | 0.03 | 0 | 0 |  | 4.03 | 16.92 | 34.26 | 30.56 | 12.01 | 2.07 | 0.16 | 0 | 0 | 0 |  | 100 | 0 |
| 67 | 2.57 | 12.3 | 29.73 | 33.35 | 17.37 | 4.19 | 0.47 | 0.02 | 0 | 0 |  | 3.94 | 16.87 | 34.42 | 30.67 | 11.93 | 2.02 | 0.15 | 0 | 0 | 0 |  | 100 | 0 |
| 68 | 2.53 | 12.26 | 29.79 | 33.44 | 17.35 | 4.15 | 0.46 | 0.02 | 0 | 0 |  | 3.89 | 16.84 | 34.51 | 30.73 | 11.88 | 1.99 | 0.14 | 0 | 0 | 0 |  | 100 | 0 |
| 69 | 2.52 | 12.24 | 29.81 | 33.48 | 17.34 | 4.13 | 0.45 | 0.02 | 0 | 0 |  | 3.88 | 16.83 | 34.55 | 30.76 | 11.87 | 1.98 | 0.14 | 0 | 0 | 0 |  | 100 | 0 |
| 70 | 2.51 | 12.24 | 29.82 | 33.48 | 17.34 | 4.13 | 0.45 | 0.02 | 0 | 0 |  | 3.83 | 16.71 | 34.45 | 30.86 | 11.99 | 2.01 | 0.14 | 0 | 0 | 0 |  | 100 | 0 |
| 71 | 2.52 | 12.24 | 29.81 | 33.48 | 17.35 | 4.14 | 0.45 | 0.02 | 0 | 0 |  | 3.84 | 16.71 | 34.44 | 30.85 | 12 | 2.02 | 0.14 | 0 | 0 | 0 |  | 100 | 0 |
| 72 | 2.52 | 12.25 | 29.8 | 33.46 | 17.35 | 4.14 | 0.45 | 0.02 | 0 | 0 |  | 3.84 | 16.71 | 34.43 | 30.84 | 12.01 | 2.02 | 0.15 | 0 | 0 | 0 |  | 100 | 0 |
| 73 | 2.53 | 12.26 | 29.79 | 33.45 | 17.35 | 4.15 | 0.45 | 0.02 | 0 | 0 |  | 3.85 | 16.72 | 34.41 | 30.83 | 12.01 | 2.02 | 0.15 | 0 | 0 | 0 |  | 100 | 0 |
| 74 | 2.53 | 12.26 | 29.78 | 33.43 | 17.36 | 4.15 | 0.46 | 0.02 | 0 | 0 |  | 3.86 | 16.72 | 34.4 | 30.82 | 12.02 | 2.03 | 0.15 | 0 | 0 | 0 |  | 100 | 0 |
| 75 | 2.54 | 12.27 | 29.77 | 33.42 | 17.36 | 4.16 | 0.46 | 0.02 | 0 | 0 |  | 3.86 | 16.72 | 34.38 | 30.82 | 12.03 | 2.04 | 0.15 | 0 | 0 | 0 |  | 100 | 0 |
| 76 | 2.54 | 12.27 | 29.77 | 33.41 | 17.36 | 4.17 | 0.46 | 0.02 | 0 | 0 |  | 3.87 | 16.72 | 34.37 | 30.81 | 12.04 | 2.04 | 0.15 | 0 | 0 | 0 |  | 100 | 0 |
| 77 | 2.55 | 12.28 | 29.76 | 33.4 | 17.36 | 4.17 | 0.46 | 0.02 | 0 | 0 |  | 3.88 | 16.73 | 34.36 | 30.8 | 12.04 | 2.04 | 0.15 | 0 | 0 | 0 |  | 100 | 0 |
| 78 | 2.56 | 12.28 | 29.75 | 33.38 | 17.37 | 4.18 | 0.46 | 0.02 | 0 | 0 |  | 3.88 | 16.73 | 34.34 | 30.79 | 12.05 | 2.05 | 0.15 | 0 | 0 | 0 |  | 100 | 0 |
| 79 | 2.56 | 12.29 | 29.74 | 33.37 | 17.37 | 4.18 | 0.46 | 0.02 | 0 | 0 |  | 3.89 | 16.73 | 34.33 | 30.78 | 12.05 | 2.05 | 0.15 | 0 | 0 | 0 |  | 100 | 0 |
| 80 | 2.57 | 12.3 | 29.73 | 33.36 | 17.37 | 4.19 | 0.46 | 0.02 | 0 | 0 |  | 3.82 | 16.49 | 34.11 | 30.96 | 12.32 | 2.14 | 0.16 | 0 | 0 | 0 |  | 100 | 0 |
| 81 | 2.57 | 12.3 | 29.72 | 33.34 | 17.38 | 4.2 | 0.47 | 0.02 | 0 | 0 |  | 3.82 | 16.5 | 34.1 | 30.95 | 12.32 | 2.14 | 0.16 | 0 | 0 | 0 |  | 100 | 0 |
| 82 | 2.58 | 12.31 | 29.71 | 33.33 | 17.38 | 4.2 | 0.47 | 0.02 | 0 | 0 |  | 3.83 | 16.5 | 34.09 | 30.94 | 12.33 | 2.15 | 0.16 | 0 | 0 | 0 |  | 100 | 0 |
| 83 | 2.58 | 12.31 | 29.7 | 33.32 | 17.38 | 4.21 | 0.47 | 0.02 | 0 | 0 |  | 3.84 | 16.5 | 34.07 | 30.93 | 12.34 | 2.15 | 0.16 | 0 | 0 | 0 |  | 100 | 0 |
| 84 | 2.59 | 12.32 | 29.7 | 33.3 | 17.39 | 4.21 | 0.47 | 0.02 | 0 | 0 |  | 3.84 | 16.51 | 34.06 | 30.92 | 12.34 | 2.16 | 0.16 | 0 | 0 | 0 |  | 100 | 0 |
| 85 | 2.59 | 12.32 | 29.69 | 33.29 | 17.39 | 4.22 | 0.47 | 0.02 | 0 | 0 |  | 3.81 | 16.39 | 33.94 | 31 | 12.48 | 2.2 | 0.17 | 0.01 | 0 | 0 |  | 100 | 0 |
| 86 | 2.6 | 12.33 | 29.68 | 33.28 | 17.39 | 4.22 | 0.47 | 0.02 | 0 | 0 |  | 3.82 | 16.39 | 33.93 | 30.99 | 12.48 | 2.21 | 0.17 | 0.01 | 0 | 0 |  | 100 | 0 |
| 87 | 2.6 | 12.34 | 29.67 | 33.27 | 17.39 | 4.23 | 0.48 | 0.02 | 0 | 0 |  | 3.82 | 16.4 | 33.92 | 30.98 | 12.49 | 2.21 | 0.17 | 0.01 | 0 | 0 |  | 100 | 0 |
| 88 | 2.61 | 12.34 | 29.66 | 33.25 | 17.4 | 4.24 | 0.48 | 0.02 | 0 | 0 |  | 3.83 | 16.4 | 33.91 | 30.97 | 12.5 | 2.22 | 0.17 | 0.01 | 0 | 0 |  | 100 | 0 |
| 89 | 2.61 | 12.35 | 29.65 | 33.24 | 17.4 | 4.24 | 0.48 | 0.02 | 0 | 0 |  | 3.84 | 16.4 | 33.89 | 30.96 | 12.5 | 2.22 | 0.17 | 0.01 | 0 | 0 |  | 100 | 0 |
| 90 | 2.62 | 12.35 | 29.64 | 33.23 | 17.4 | 4.25 | 0.48 | 0.02 | 0 | 0 |  | 3.84 | 16.41 | 33.88 | 30.95 | 12.51 | 2.23 | 0.17 | 0.01 | 0 | 0 |  | 100 | 0 |
| 91 | 2.62 | 12.36 | 29.64 | 33.21 | 17.41 | 4.25 | 0.48 | 0.02 | 0 | 0 |  | 3.85 | 16.41 | 33.87 | 30.95 | 12.51 | 2.23 | 0.17 | 0.01 | 0 | 0 |  | 100 | 0 |
| 92 | 2.63 | 12.36 | 29.63 | 33.2 | 17.41 | 4.26 | 0.48 | 0.02 | 0 | 0 |  | 3.86 | 16.42 | 33.86 | 30.94 | 12.52 | 2.23 | 0.18 | 0.01 | 0 | 0 |  | 100 | 0 |
| 93 | 2.64 | 12.37 | 29.62 | 33.19 | 17.41 | 4.26 | 0.48 | 0.03 | 0 | 0 |  | 3.86 | 16.42 | 33.84 | 30.93 | 12.53 | 2.24 | 0.18 | 0.01 | 0 | 0 |  | 100 | 0 |
| 94 | 2.64 | 12.38 | 29.61 | 33.17 | 17.41 | 4.27 | 0.49 | 0.03 | 0 | 0 |  | 3.87 | 16.42 | 33.83 | 30.92 | 12.53 | 2.24 | 0.18 | 0.01 | 0 | 0 |  | 100 | 0 |
| 95 | 2.65 | 12.38 | 29.6 | 33.16 | 17.42 | 4.28 | 0.49 | 0.03 | 0 | 0 |  | 3.88 | 16.43 | 33.82 | 30.91 | 12.54 | 2.25 | 0.18 | 0.01 | 0 | 0 |  | 100 | 0 |

^a^ Rounded to two decimal points

^b^ %E = Percent of total energy intake
