# Supplementary material for: Substituting polyunsaturated fat for saturated fat: A health impact assessment of a fat tax in seven European countries
Source: PLoS One. 2019 Jul 10;14(7):e0218464. doi: 10.1371/journal.pone.0218464 (PMC6619676; doi:10.1371/journal.pone.0218464)
Supplement: S16 Table — (DOCX) [file pone.0218464.s016.docx]

# S16 Table. Proportion of persons in the respective saturated fat intake categories across scenarios in the UK.

| Age | Reference scenario^a^ | | | | | | | | | |  | Fat tax scenario^a^ | | | | | | | | | |  | Guideline scenario | |
| --- | --- | --- | --- | --- | --- | --- | --- | --- | --- | --- | --- | --- | --- | --- | --- | --- | --- | --- | --- | --- | --- | --- | --- | --- |
|  | Category of saturated fat intake (in %E)^b^ | | | | | | | | | |  | Category of saturated fat intake (in %E)^b^ | | | | | | | | | |  | Category of saturated fat intake (in %E)^b^ | |
|  | ≤10 | >10 ≤12 | >12 ≤14 | >14 ≤16 | >16 ≤18 | >18 ≤20 | >20 ≤22 | >22 ≤24 | >24 ≤26 | >26 ≤100 |  | ≤10 | >10 ≤12 | >12 ≤14 | >14 ≤16 | >16 ≤18 | >18 ≤20 | >20 ≤22 | >22 ≤24 | >24 ≤26 | >26 ≤100 |  | ≤10 | >10 ≤100 |
|  |  |  | Males | | | | | | | | | | | | | | | | | | | | | |
| 0 | 100 | 0 | 0 | 0 | 0 | 0 | 0 | 0 | 0 | 0 |  | 100 | 0 | 0 | 0 | 0 | 0 | 0 | 0 | 0 | 0 |  | 100 | 0 |
| 1 | 100 | 0 | 0 | 0 | 0 | 0 | 0 | 0 | 0 | 0 |  | 100 | 0 | 0 | 0 | 0 | 0 | 0 | 0 | 0 | 0 |  | 100 | 0 |
| 2 | 100 | 0 | 0 | 0 | 0 | 0 | 0 | 0 | 0 | 0 |  | 100 | 0 | 0 | 0 | 0 | 0 | 0 | 0 | 0 | 0 |  | 100 | 0 |
| 3 | 100 | 0 | 0 | 0 | 0 | 0 | 0 | 0 | 0 | 0 |  | 100 | 0 | 0 | 0 | 0 | 0 | 0 | 0 | 0 | 0 |  | 100 | 0 |
| 4 | 100 | 0 | 0 | 0 | 0 | 0 | 0 | 0 | 0 | 0 |  | 100 | 0 | 0 | 0 | 0 | 0 | 0 | 0 | 0 | 0 |  | 100 | 0 |
| 5 | 100 | 0 | 0 | 0 | 0 | 0 | 0 | 0 | 0 | 0 |  | 100 | 0 | 0 | 0 | 0 | 0 | 0 | 0 | 0 | 0 |  | 100 | 0 |
| 6 | 5.96 | 13.31 | 23.67 | 26.63 | 18.96 | 8.54 | 2.43 | 0.44 | 0.05 | 0 |  | 5.96 | 13.31 | 23.67 | 26.63 | 18.96 | 8.54 | 2.43 | 0.44 | 0.05 | 0 |  | 100 | 0 |
| 7 | 5.93 | 13.27 | 23.64 | 26.65 | 19 | 8.57 | 2.44 | 0.44 | 0.05 | 0 |  | 5.93 | 13.27 | 23.64 | 26.65 | 19 | 8.57 | 2.44 | 0.44 | 0.05 | 0 |  | 100 | 0 |
| 8 | 5.9 | 13.24 | 23.62 | 26.66 | 19.04 | 8.59 | 2.45 | 0.44 | 0.05 | 0 |  | 5.9 | 13.24 | 23.62 | 26.66 | 19.04 | 8.59 | 2.45 | 0.44 | 0.05 | 0 |  | 100 | 0 |
| 9 | 5.89 | 13.22 | 23.61 | 26.67 | 19.05 | 8.6 | 2.46 | 0.44 | 0.05 | 0 |  | 5.89 | 13.22 | 23.61 | 26.67 | 19.05 | 8.6 | 2.46 | 0.44 | 0.05 | 0 |  | 100 | 0 |
| 10 | 5.92 | 13.26 | 23.64 | 26.66 | 19.02 | 8.58 | 2.45 | 0.44 | 0.05 | 0 |  | 5.92 | 13.26 | 23.64 | 26.66 | 19.02 | 8.58 | 2.45 | 0.44 | 0.05 | 0 |  | 100 | 0 |
| 11 | 6.02 | 13.37 | 23.71 | 26.6 | 18.9 | 8.5 | 2.42 | 0.43 | 0.05 | 0 |  | 6.02 | 13.37 | 23.71 | 26.6 | 18.9 | 8.5 | 2.42 | 0.43 | 0.05 | 0 |  | 100 | 0 |
| 12 | 6.23 | 13.62 | 23.86 | 26.49 | 18.65 | 8.32 | 2.35 | 0.42 | 0.05 | 0 |  | 6.23 | 13.62 | 23.86 | 26.49 | 18.65 | 8.32 | 2.35 | 0.42 | 0.05 | 0 |  | 100 | 0 |
| 13 | 6.61 | 14.04 | 24.1 | 26.3 | 18.23 | 8.03 | 2.25 | 0.4 | 0.04 | 0 |  | 6.61 | 14.04 | 24.1 | 26.3 | 18.23 | 8.03 | 2.25 | 0.4 | 0.04 | 0 |  | 100 | 0 |
| 14 | 7.2 | 14.66 | 24.44 | 25.98 | 17.61 | 7.61 | 2.09 | 0.37 | 0.04 | 0 |  | 7.2 | 14.66 | 24.44 | 25.98 | 17.61 | 7.61 | 2.09 | 0.37 | 0.04 | 0 |  | 100 | 0 |
| 15 | 8 | 15.45 | 24.83 | 25.54 | 16.82 | 7.09 | 1.91 | 0.33 | 0.04 | 0 |  | 10.83 | 19.29 | 27.56 | 24.14 | 12.95 | 4.25 | 0.86 | 0.1 | 0.01 | 0 |  | 100 | 0 |
| 16 | 8.95 | 16.31 | 25.19 | 25.01 | 15.95 | 6.54 | 1.72 | 0.29 | 0.03 | 0 |  | 12.03 | 20.19 | 27.66 | 23.32 | 12.09 | 3.86 | 0.76 | 0.09 | 0.01 | 0 |  | 100 | 0 |
| 17 | 9.87 | 17.09 | 25.47 | 24.48 | 15.17 | 6.06 | 1.56 | 0.26 | 0.03 | 0 |  | 13.2 | 20.97 | 27.68 | 22.55 | 11.33 | 3.51 | 0.67 | 0.08 | 0.01 | 0 |  | 100 | 0 |
| 18 | 10.62 | 17.67 | 25.64 | 24.05 | 14.59 | 5.72 | 1.45 | 0.24 | 0.02 | 0 |  | 14.13 | 21.55 | 27.64 | 21.94 | 10.78 | 3.27 | 0.61 | 0.07 | 0 | 0 |  | 100 | 0 |
| 19 | 11.16 | 18.04 | 25.69 | 23.73 | 14.22 | 5.52 | 1.39 | 0.23 | 0.02 | 0 |  | 14.86 | 21.95 | 27.57 | 21.48 | 10.38 | 3.11 | 0.58 | 0.07 | 0 | 0 |  | 100 | 0 |
| 20 | 11.53 | 18.21 | 25.64 | 23.5 | 14.04 | 5.46 | 1.38 | 0.23 | 0.02 | 0 |  | 15.21 | 22 | 27.41 | 21.29 | 10.31 | 3.11 | 0.58 | 0.07 | 0 | 0 |  | 100 | 0 |
| 21 | 11.8 | 18.22 | 25.45 | 23.31 | 14.01 | 5.52 | 1.42 | 0.24 | 0.03 | 0 |  | 15.51 | 21.96 | 27.18 | 21.15 | 10.34 | 3.17 | 0.61 | 0.07 | 0.01 | 0 |  | 100 | 0 |
| 22 | 12.04 | 18.1 | 25.11 | 23.11 | 14.1 | 5.7 | 1.53 | 0.27 | 0.03 | 0 |  | 15.74 | 21.74 | 26.83 | 21.07 | 10.52 | 3.34 | 0.67 | 0.09 | 0.01 | 0 |  | 100 | 0 |
| 23 | 12.32 | 17.86 | 24.62 | 22.85 | 14.28 | 6 | 1.7 | 0.32 | 0.04 | 0 |  | 15.99 | 21.37 | 26.34 | 20.99 | 10.82 | 3.6 | 0.78 | 0.11 | 0.01 | 0 |  | 100 | 0 |
| 24 | 12.66 | 17.54 | 23.99 | 22.52 | 14.49 | 6.4 | 1.94 | 0.4 | 0.06 | 0.01 |  | 16.28 | 20.9 | 25.7 | 20.88 | 11.2 | 3.96 | 0.92 | 0.14 | 0.01 | 0 |  | 100 | 0 |
| 25 | 13.01 | 17.2 | 23.34 | 22.15 | 14.69 | 6.81 | 2.21 | 0.5 | 0.08 | 0.01 |  | 16.48 | 20.32 | 25.01 | 20.78 | 11.65 | 4.41 | 1.12 | 0.19 | 0.02 | 0 |  | 100 | 0 |
| 26 | 13.33 | 16.9 | 22.78 | 21.81 | 14.84 | 7.17 | 2.46 | 0.6 | 0.1 | 0.01 |  | 16.75 | 19.89 | 24.43 | 20.62 | 11.96 | 4.76 | 1.3 | 0.24 | 0.03 | 0 |  | 100 | 0 |
| 27 | 13.56 | 16.68 | 22.36 | 21.55 | 14.93 | 7.44 | 2.66 | 0.68 | 0.13 | 0.02 |  | 16.95 | 19.57 | 24 | 20.49 | 12.17 | 5.03 | 1.45 | 0.29 | 0.04 | 0 |  | 100 | 0 |
| 28 | 13.72 | 16.53 | 22.08 | 21.37 | 14.98 | 7.61 | 2.8 | 0.74 | 0.14 | 0.02 |  | 17.09 | 19.36 | 23.71 | 20.39 | 12.31 | 5.21 | 1.55 | 0.32 | 0.05 | 0 |  | 100 | 0 |
| 29 | 13.83 | 16.45 | 21.92 | 21.26 | 15 | 7.7 | 2.88 | 0.78 | 0.15 | 0.02 |  | 17.18 | 19.24 | 23.55 | 20.33 | 12.38 | 5.32 | 1.61 | 0.34 | 0.05 | 0.01 |  | 100 | 0 |
| 30 | 13.92 | 16.42 | 21.84 | 21.19 | 14.99 | 7.74 | 2.91 | 0.8 | 0.16 | 0.03 |  | 17.19 | 19.12 | 23.42 | 20.31 | 12.46 | 5.41 | 1.66 | 0.36 | 0.06 | 0.01 |  | 100 | 0 |
| 31 | 14.03 | 16.44 | 21.8 | 21.13 | 14.95 | 7.73 | 2.92 | 0.8 | 0.16 | 0.03 |  | 17.31 | 19.13 | 23.37 | 20.25 | 12.43 | 5.41 | 1.67 | 0.36 | 0.06 | 0.01 |  | 100 | 0 |
| 32 | 14.19 | 16.49 | 21.79 | 21.05 | 14.88 | 7.69 | 2.91 | 0.8 | 0.16 | 0.03 |  | 17.49 | 19.17 | 23.34 | 20.16 | 12.37 | 5.38 | 1.66 | 0.36 | 0.06 | 0.01 |  | 100 | 0 |
| 33 | 14.42 | 16.57 | 21.77 | 20.96 | 14.77 | 7.63 | 2.89 | 0.8 | 0.16 | 0.03 |  | 17.75 | 19.24 | 23.29 | 20.05 | 12.27 | 5.33 | 1.65 | 0.36 | 0.06 | 0.01 |  | 100 | 0 |
| 34 | 14.73 | 16.67 | 21.75 | 20.83 | 14.64 | 7.55 | 2.86 | 0.79 | 0.16 | 0.03 |  | 18.1 | 19.32 | 23.23 | 19.89 | 12.13 | 5.27 | 1.63 | 0.36 | 0.06 | 0.01 |  | 100 | 0 |
| 35 | 15.07 | 16.78 | 21.71 | 20.68 | 14.49 | 7.46 | 2.83 | 0.79 | 0.16 | 0.03 |  | 18.48 | 19.4 | 23.14 | 19.72 | 12 | 5.21 | 1.62 | 0.36 | 0.06 | 0.01 |  | 100 | 0 |
| 36 | 15.39 | 16.87 | 21.68 | 20.54 | 14.35 | 7.39 | 2.8 | 0.78 | 0.16 | 0.03 |  | 18.84 | 19.47 | 23.07 | 19.56 | 11.87 | 5.16 | 1.6 | 0.36 | 0.06 | 0.01 |  | 100 | 0 |
| 37 | 15.64 | 16.94 | 21.65 | 20.44 | 14.25 | 7.33 | 2.79 | 0.78 | 0.16 | 0.03 |  | 19.11 | 19.53 | 23.01 | 19.44 | 11.78 | 5.12 | 1.59 | 0.36 | 0.06 | 0.01 |  | 100 | 0 |
| 38 | 15.8 | 16.98 | 21.63 | 20.37 | 14.18 | 7.3 | 2.77 | 0.78 | 0.16 | 0.03 |  | 19.3 | 19.56 | 22.97 | 19.36 | 11.72 | 5.09 | 1.59 | 0.36 | 0.06 | 0.01 |  | 100 | 0 |
| 39 | 15.89 | 17 | 21.61 | 20.33 | 14.14 | 7.28 | 2.77 | 0.78 | 0.16 | 0.03 |  | 19.4 | 19.57 | 22.94 | 19.32 | 11.69 | 5.08 | 1.59 | 0.36 | 0.06 | 0.01 |  | 100 | 0 |
| 40 | 15.92 | 16.99 | 21.58 | 20.3 | 14.14 | 7.29 | 2.78 | 0.79 | 0.16 | 0.03 |  | 19.34 | 19.49 | 22.88 | 19.33 | 11.75 | 5.14 | 1.62 | 0.37 | 0.06 | 0.01 |  | 100 | 0 |
| 41 | 15.9 | 16.95 | 21.55 | 20.3 | 14.17 | 7.33 | 2.81 | 0.8 | 0.17 | 0.03 |  | 19.31 | 19.45 | 22.85 | 19.34 | 11.79 | 5.18 | 1.64 | 0.37 | 0.06 | 0.01 |  | 100 | 0 |
| 42 | 15.84 | 16.87 | 21.48 | 20.3 | 14.23 | 7.4 | 2.86 | 0.82 | 0.17 | 0.03 |  | 19.23 | 19.36 | 22.8 | 19.37 | 11.87 | 5.25 | 1.67 | 0.38 | 0.06 | 0.01 |  | 100 | 0 |
| 43 | 15.74 | 16.76 | 21.39 | 20.3 | 14.32 | 7.51 | 2.93 | 0.85 | 0.18 | 0.03 |  | 19.09 | 19.24 | 22.73 | 19.41 | 11.98 | 5.35 | 1.72 | 0.4 | 0.07 | 0.01 |  | 100 | 0 |
| 44 | 15.61 | 16.61 | 21.27 | 20.3 | 14.43 | 7.64 | 3.02 | 0.89 | 0.19 | 0.04 |  | 18.92 | 19.07 | 22.63 | 19.46 | 12.13 | 5.48 | 1.79 | 0.43 | 0.07 | 0.01 |  | 100 | 0 |
| 45 | 15.47 | 16.46 | 21.14 | 20.29 | 14.55 | 7.79 | 3.12 | 0.93 | 0.21 | 0.04 |  | 18.74 | 18.9 | 22.52 | 19.51 | 12.29 | 5.63 | 1.87 | 0.45 | 0.08 | 0.01 |  | 100 | 0 |
| 46 | 15.35 | 16.32 | 21.02 | 20.28 | 14.65 | 7.93 | 3.21 | 0.97 | 0.22 | 0.04 |  | 18.58 | 18.74 | 22.42 | 19.56 | 12.43 | 5.76 | 1.94 | 0.48 | 0.08 | 0.01 |  | 100 | 0 |
| 47 | 15.26 | 16.21 | 20.93 | 20.28 | 14.73 | 8.03 | 3.28 | 1.01 | 0.23 | 0.04 |  | 18.45 | 18.62 | 22.35 | 19.59 | 12.54 | 5.86 | 2 | 0.5 | 0.09 | 0.01 |  | 100 | 0 |
| 48 | 15.2 | 16.14 | 20.87 | 20.27 | 14.78 | 8.1 | 3.33 | 1.03 | 0.24 | 0.05 |  | 18.38 | 18.54 | 22.29 | 19.6 | 12.61 | 5.93 | 2.04 | 0.51 | 0.09 | 0.01 |  | 100 | 0 |
| 49 | 15.18 | 16.1 | 20.83 | 20.26 | 14.81 | 8.14 | 3.36 | 1.04 | 0.24 | 0.05 |  | 18.34 | 18.49 | 22.26 | 19.61 | 12.65 | 5.97 | 2.06 | 0.52 | 0.1 | 0.01 |  | 100 | 0 |
| 50 | 15.19 | 16.08 | 20.8 | 20.24 | 14.81 | 8.15 | 3.38 | 1.05 | 0.25 | 0.05 |  | 18.19 | 18.36 | 22.17 | 19.64 | 12.77 | 6.08 | 2.13 | 0.54 | 0.1 | 0.02 |  | 100 | 0 |
| 51 | 15.25 | 16.09 | 20.78 | 20.21 | 14.79 | 8.15 | 3.38 | 1.05 | 0.25 | 0.05 |  | 18.26 | 18.35 | 22.14 | 19.61 | 12.75 | 6.08 | 2.13 | 0.55 | 0.1 | 0.02 |  | 100 | 0 |
| 52 | 15.37 | 16.11 | 20.75 | 20.15 | 14.75 | 8.13 | 3.38 | 1.06 | 0.25 | 0.05 |  | 18.38 | 18.36 | 22.1 | 19.55 | 12.72 | 6.08 | 2.13 | 0.55 | 0.1 | 0.02 |  | 100 | 0 |
| 53 | 15.54 | 16.13 | 20.71 | 20.07 | 14.68 | 8.11 | 3.38 | 1.06 | 0.25 | 0.05 |  | 18.57 | 18.38 | 22.05 | 19.47 | 12.66 | 6.06 | 2.14 | 0.55 | 0.1 | 0.02 |  | 100 | 0 |
| 54 | 15.77 | 16.17 | 20.67 | 19.97 | 14.6 | 8.07 | 3.38 | 1.07 | 0.26 | 0.05 |  | 18.82 | 18.4 | 21.98 | 19.36 | 12.59 | 6.04 | 2.14 | 0.56 | 0.11 | 0.02 |  | 100 | 0 |
| 55 | 16.02 | 16.22 | 20.61 | 19.86 | 14.51 | 8.03 | 3.37 | 1.07 | 0.26 | 0.05 |  | 19.08 | 18.42 | 21.9 | 19.25 | 12.51 | 6.01 | 2.14 | 0.56 | 0.11 | 0.02 |  | 100 | 0 |
| 56 | 16.25 | 16.26 | 20.57 | 19.76 | 14.42 | 7.99 | 3.36 | 1.07 | 0.26 | 0.06 |  | 19.34 | 18.45 | 21.83 | 19.14 | 12.44 | 5.99 | 2.14 | 0.56 | 0.11 | 0.02 |  | 100 | 0 |
| 57 | 16.43 | 16.29 | 20.54 | 19.69 | 14.35 | 7.96 | 3.36 | 1.08 | 0.26 | 0.06 |  | 19.53 | 18.47 | 21.78 | 19.05 | 12.37 | 5.96 | 2.13 | 0.57 | 0.11 | 0.02 |  | 100 | 0 |
| 58 | 16.54 | 16.31 | 20.51 | 19.63 | 14.31 | 7.94 | 3.35 | 1.08 | 0.26 | 0.06 |  | 19.66 | 18.48 | 21.74 | 19 | 12.34 | 5.95 | 2.13 | 0.57 | 0.11 | 0.02 |  | 100 | 0 |
| 59 | 16.59 | 16.3 | 20.47 | 19.6 | 14.3 | 7.95 | 3.37 | 1.09 | 0.27 | 0.06 |  | 19.7 | 18.46 | 21.7 | 18.98 | 12.34 | 5.97 | 2.15 | 0.57 | 0.11 | 0.02 |  | 100 | 0 |
| 60 | 16.56 | 16.23 | 20.41 | 19.59 | 14.34 | 8.01 | 3.41 | 1.11 | 0.27 | 0.06 |  | 19.57 | 18.34 | 21.62 | 19 | 12.45 | 6.07 | 2.21 | 0.6 | 0.12 | 0.02 |  | 100 | 0 |
| 61 | 16.44 | 16.11 | 20.31 | 19.58 | 14.43 | 8.13 | 3.5 | 1.15 | 0.29 | 0.06 |  | 19.42 | 18.19 | 21.53 | 19.03 | 12.57 | 6.2 | 2.28 | 0.63 | 0.13 | 0.02 |  | 100 | 0 |
| 62 | 16.21 | 15.88 | 20.13 | 19.56 | 14.59 | 8.34 | 3.66 | 1.23 | 0.32 | 0.07 |  | 19.14 | 17.95 | 21.38 | 19.09 | 12.78 | 6.41 | 2.41 | 0.68 | 0.14 | 0.03 |  | 100 | 0 |
| 63 | 15.89 | 15.56 | 19.85 | 19.53 | 14.81 | 8.65 | 3.9 | 1.35 | 0.36 | 0.09 |  | 18.73 | 17.59 | 21.14 | 19.15 | 13.08 | 6.73 | 2.61 | 0.76 | 0.17 | 0.03 |  | 100 | 0 |
| 64 | 15.5 | 15.15 | 19.5 | 19.46 | 15.07 | 9.05 | 4.21 | 1.52 | 0.43 | 0.11 |  | 18.23 | 17.14 | 20.83 | 19.21 | 13.44 | 7.14 | 2.88 | 0.88 | 0.2 | 0.04 |  | 100 | 0 |
| 65 | 15.08 | 14.72 | 19.11 | 19.37 | 15.33 | 9.47 | 4.57 | 1.72 | 0.51 | 0.14 |  | 17.59 | 16.58 | 20.42 | 19.25 | 13.89 | 7.67 | 3.24 | 1.05 | 0.26 | 0.06 |  | 100 | 0 |
| 66 | 14.72 | 14.34 | 18.75 | 19.26 | 15.53 | 9.84 | 4.89 | 1.91 | 0.59 | 0.17 |  | 17.15 | 16.15 | 20.09 | 19.24 | 14.2 | 8.07 | 3.53 | 1.19 | 0.31 | 0.07 |  | 100 | 0 |
| 67 | 14.46 | 14.05 | 18.47 | 19.16 | 15.68 | 10.11 | 5.15 | 2.07 | 0.65 | 0.2 |  | 16.82 | 15.83 | 19.83 | 19.22 | 14.42 | 8.37 | 3.76 | 1.31 | 0.35 | 0.09 |  | 100 | 0 |
| 68 | 14.29 | 13.86 | 18.29 | 19.09 | 15.76 | 10.29 | 5.32 | 2.17 | 0.7 | 0.22 |  | 16.6 | 15.62 | 19.65 | 19.2 | 14.56 | 8.58 | 3.92 | 1.39 | 0.38 | 0.1 |  | 100 | 0 |
| 69 | 14.18 | 13.75 | 18.18 | 19.05 | 15.81 | 10.4 | 5.42 | 2.24 | 0.73 | 0.24 |  | 16.47 | 15.5 | 19.55 | 19.19 | 14.64 | 8.69 | 4.01 | 1.44 | 0.4 | 0.1 |  | 100 | 0 |
| 70 | 14.11 | 13.69 | 18.14 | 19.04 | 15.85 | 10.45 | 5.47 | 2.27 | 0.74 | 0.24 |  | 16.31 | 15.39 | 19.48 | 19.19 | 14.73 | 8.8 | 4.09 | 1.48 | 0.42 | 0.11 |  | 100 | 0 |
| 71 | 14.04 | 13.67 | 18.13 | 19.06 | 15.88 | 10.48 | 5.48 | 2.27 | 0.75 | 0.24 |  | 16.24 | 15.37 | 19.48 | 19.22 | 14.76 | 8.82 | 4.1 | 1.49 | 0.42 | 0.11 |  | 100 | 0 |
| 72 | 13.95 | 13.66 | 18.16 | 19.11 | 15.91 | 10.49 | 5.48 | 2.26 | 0.74 | 0.24 |  | 16.15 | 15.36 | 19.51 | 19.26 | 14.79 | 8.83 | 4.1 | 1.48 | 0.41 | 0.11 |  | 100 | 0 |
| 73 | 13.83 | 13.66 | 18.2 | 19.18 | 15.96 | 10.5 | 5.46 | 2.25 | 0.73 | 0.23 |  | 16.02 | 15.37 | 19.57 | 19.33 | 14.83 | 8.83 | 4.08 | 1.46 | 0.41 | 0.1 |  | 100 | 0 |
| 74 | 13.66 | 13.65 | 18.26 | 19.27 | 16.03 | 10.52 | 5.45 | 2.22 | 0.72 | 0.22 |  | 15.84 | 15.37 | 19.63 | 19.43 | 14.89 | 8.83 | 4.06 | 1.44 | 0.4 | 0.1 |  | 100 | 0 |
| 75 | 13.48 | 13.63 | 18.31 | 19.36 | 16.11 | 10.55 | 5.44 | 2.2 | 0.7 | 0.22 |  | 15.58 | 15.32 | 19.67 | 19.53 | 14.99 | 8.89 | 4.08 | 1.44 | 0.4 | 0.1 |  | 100 | 0 |
| 76 | 13.3 | 13.61 | 18.36 | 19.45 | 16.18 | 10.57 | 5.43 | 2.19 | 0.69 | 0.21 |  | 15.4 | 15.31 | 19.74 | 19.62 | 15.05 | 8.9 | 4.06 | 1.43 | 0.39 | 0.1 |  | 100 | 0 |
| 77 | 13.17 | 13.59 | 18.4 | 19.52 | 16.24 | 10.6 | 5.42 | 2.17 | 0.68 | 0.21 |  | 15.26 | 15.3 | 19.78 | 19.7 | 15.1 | 8.91 | 4.05 | 1.42 | 0.38 | 0.09 |  | 100 | 0 |
| 78 | 13.08 | 13.58 | 18.42 | 19.57 | 16.28 | 10.61 | 5.42 | 2.17 | 0.68 | 0.2 |  | 15.17 | 15.29 | 19.81 | 19.75 | 15.14 | 8.92 | 4.04 | 1.41 | 0.38 | 0.09 |  | 100 | 0 |
| 79 | 13.03 | 13.57 | 18.43 | 19.59 | 16.3 | 10.62 | 5.41 | 2.16 | 0.67 | 0.2 |  | 15.11 | 15.29 | 19.83 | 19.78 | 15.15 | 8.93 | 4.04 | 1.41 | 0.38 | 0.09 |  | 100 | 0 |
| 80 | 13 | 13.57 | 18.44 | 19.61 | 16.32 | 10.62 | 5.41 | 2.16 | 0.67 | 0.2 |  | 15.02 | 15.24 | 19.8 | 19.79 | 15.2 | 8.98 | 4.08 | 1.42 | 0.38 | 0.09 |  | 100 | 0 |
| 81 | 12.99 | 13.56 | 18.44 | 19.61 | 16.32 | 10.63 | 5.41 | 2.16 | 0.67 | 0.2 |  | 15.01 | 15.24 | 19.81 | 19.79 | 15.2 | 8.98 | 4.08 | 1.42 | 0.38 | 0.09 |  | 100 | 0 |
| 82 | 13 | 13.56 | 18.44 | 19.61 | 16.32 | 10.63 | 5.42 | 2.16 | 0.67 | 0.2 |  | 15.02 | 15.24 | 19.8 | 19.79 | 15.2 | 8.98 | 4.08 | 1.42 | 0.38 | 0.09 |  | 100 | 0 |
| 83 | 13 | 13.57 | 18.44 | 19.6 | 16.32 | 10.63 | 5.42 | 2.16 | 0.67 | 0.2 |  | 15.02 | 15.24 | 19.8 | 19.79 | 15.2 | 8.98 | 4.08 | 1.42 | 0.38 | 0.09 |  | 100 | 0 |
| 84 | 13.01 | 13.57 | 18.43 | 19.6 | 16.31 | 10.62 | 5.42 | 2.16 | 0.67 | 0.2 |  | 15.03 | 15.24 | 19.8 | 19.78 | 15.2 | 8.98 | 4.08 | 1.42 | 0.38 | 0.09 |  | 100 | 0 |
| 85 | 13.02 | 13.57 | 18.43 | 19.6 | 16.31 | 10.62 | 5.42 | 2.16 | 0.68 | 0.2 |  | 14.92 | 15.14 | 19.73 | 19.78 | 15.26 | 9.07 | 4.15 | 1.46 | 0.4 | 0.1 |  | 100 | 0 |
| 86 | 13.02 | 13.57 | 18.43 | 19.59 | 16.31 | 10.62 | 5.42 | 2.16 | 0.68 | 0.2 |  | 14.93 | 15.14 | 19.72 | 19.78 | 15.26 | 9.07 | 4.15 | 1.46 | 0.4 | 0.1 |  | 100 | 0 |
| 87 | 13.03 | 13.57 | 18.43 | 19.59 | 16.3 | 10.62 | 5.42 | 2.16 | 0.68 | 0.2 |  | 14.93 | 15.14 | 19.72 | 19.77 | 15.26 | 9.07 | 4.15 | 1.46 | 0.4 | 0.1 |  | 100 | 0 |
| 88 | 13.03 | 13.57 | 18.43 | 19.59 | 16.3 | 10.62 | 5.42 | 2.16 | 0.68 | 0.2 |  | 14.94 | 15.15 | 19.72 | 19.77 | 15.26 | 9.07 | 4.15 | 1.46 | 0.4 | 0.1 |  | 100 | 0 |
| 89 | 13.03 | 13.57 | 18.43 | 19.59 | 16.3 | 10.62 | 5.42 | 2.16 | 0.68 | 0.2 |  | 14.94 | 15.15 | 19.72 | 19.77 | 15.26 | 9.07 | 4.15 | 1.46 | 0.4 | 0.1 |  | 100 | 0 |
| 90 | 13.03 | 13.57 | 18.43 | 19.59 | 16.3 | 10.62 | 5.42 | 2.16 | 0.68 | 0.2 |  | 14.94 | 15.15 | 19.72 | 19.77 | 15.26 | 9.07 | 4.15 | 1.46 | 0.4 | 0.1 |  | 100 | 0 |
| 91 | 13.03 | 13.57 | 18.43 | 19.59 | 16.3 | 10.62 | 5.42 | 2.16 | 0.68 | 0.2 |  | 14.94 | 15.15 | 19.72 | 19.77 | 15.26 | 9.07 | 4.15 | 1.46 | 0.4 | 0.1 |  | 100 | 0 |
| 92 | 13.03 | 13.57 | 18.43 | 19.59 | 16.3 | 10.62 | 5.42 | 2.16 | 0.68 | 0.2 |  | 14.94 | 15.15 | 19.72 | 19.77 | 15.26 | 9.07 | 4.15 | 1.46 | 0.4 | 0.1 |  | 100 | 0 |
| 93 | 13.03 | 13.57 | 18.43 | 19.59 | 16.3 | 10.62 | 5.42 | 2.16 | 0.68 | 0.2 |  | 14.94 | 15.15 | 19.72 | 19.77 | 15.26 | 9.07 | 4.15 | 1.46 | 0.4 | 0.1 |  | 100 | 0 |
| 94 | 13.03 | 13.57 | 18.43 | 19.59 | 16.3 | 10.62 | 5.42 | 2.16 | 0.68 | 0.2 |  | 14.94 | 15.15 | 19.72 | 19.77 | 15.26 | 9.07 | 4.15 | 1.46 | 0.4 | 0.1 |  | 100 | 0 |
| 95 | 13.03 | 13.57 | 18.43 | 19.59 | 16.3 | 10.62 | 5.42 | 2.16 | 0.68 | 0.2 |  | 14.93 | 15.15 | 19.72 | 19.77 | 15.26 | 9.07 | 4.15 | 1.46 | 0.4 | 0.1 |  | 100 | 0 |
|  | |  | Females | | | | | | | | | | | | | | | | | | | | | |
| 0 | 100 | 0 | 0 | 0 | 0 | 0 | 0 | 0 | 0 | 0 |  | 100 | 0 | 0 | 0 | 0 | 0 | 0 | 0 | 0 | 0 |  | 100 | 0 |
| 1 | 100 | 0 | 0 | 0 | 0 | 0 | 0 | 0 | 0 | 0 |  | 100 | 0 | 0 | 0 | 0 | 0 | 0 | 0 | 0 | 0 |  | 100 | 0 |
| 2 | 100 | 0 | 0 | 0 | 0 | 0 | 0 | 0 | 0 | 0 |  | 100 | 0 | 0 | 0 | 0 | 0 | 0 | 0 | 0 | 0 |  | 100 | 0 |
| 3 | 100 | 0 | 0 | 0 | 0 | 0 | 0 | 0 | 0 | 0 |  | 100 | 0 | 0 | 0 | 0 | 0 | 0 | 0 | 0 | 0 |  | 100 | 0 |
| 4 | 100 | 0 | 0 | 0 | 0 | 0 | 0 | 0 | 0 | 0 |  | 100 | 0 | 0 | 0 | 0 | 0 | 0 | 0 | 0 | 0 |  | 100 | 0 |
| 5 | 100 | 0 | 0 | 0 | 0 | 0 | 0 | 0 | 0 | 0 |  | 100 | 0 | 0 | 0 | 0 | 0 | 0 | 0 | 0 | 0 |  | 100 | 0 |
| 6 | 6.95 | 13.92 | 23.46 | 25.72 | 18.33 | 8.49 | 2.56 | 0.5 | 0.06 | 0 |  | 6.95 | 13.92 | 23.46 | 25.72 | 18.33 | 8.49 | 2.56 | 0.5 | 0.06 | 0 |  | 100 | 0 |
| 7 | 6.87 | 13.85 | 23.44 | 25.77 | 18.4 | 8.53 | 2.57 | 0.5 | 0.06 | 0 |  | 6.87 | 13.85 | 23.44 | 25.77 | 18.4 | 8.53 | 2.57 | 0.5 | 0.06 | 0 |  | 100 | 0 |
| 8 | 6.8 | 13.79 | 23.42 | 25.81 | 18.46 | 8.56 | 2.58 | 0.5 | 0.06 | 0 |  | 6.8 | 13.79 | 23.42 | 25.81 | 18.46 | 8.56 | 2.58 | 0.5 | 0.06 | 0 |  | 100 | 0 |
| 9 | 6.78 | 13.77 | 23.42 | 25.83 | 18.48 | 8.58 | 2.58 | 0.5 | 0.06 | 0 |  | 6.78 | 13.77 | 23.42 | 25.83 | 18.48 | 8.58 | 2.58 | 0.5 | 0.06 | 0 |  | 100 | 0 |
| 10 | 6.84 | 13.83 | 23.43 | 25.79 | 18.42 | 8.54 | 2.57 | 0.5 | 0.06 | 0 |  | 6.84 | 13.83 | 23.43 | 25.79 | 18.42 | 8.54 | 2.57 | 0.5 | 0.06 | 0 |  | 100 | 0 |
| 11 | 7.08 | 14.02 | 23.5 | 25.64 | 18.22 | 8.43 | 2.54 | 0.5 | 0.06 | 0.01 |  | 7.08 | 14.02 | 23.5 | 25.64 | 18.22 | 8.43 | 2.54 | 0.5 | 0.06 | 0 |  | 100 | 0 |
| 12 | 7.58 | 14.43 | 23.62 | 25.33 | 17.81 | 8.2 | 2.47 | 0.49 | 0.06 | 0.01 |  | 7.58 | 14.43 | 23.62 | 25.33 | 17.81 | 8.2 | 2.47 | 0.49 | 0.06 | 0.01 |  | 100 | 0 |
| 13 | 8.47 | 15.1 | 23.78 | 24.8 | 17.12 | 7.82 | 2.37 | 0.47 | 0.06 | 0.01 |  | 8.47 | 15.1 | 23.78 | 24.8 | 17.12 | 7.82 | 2.37 | 0.47 | 0.06 | 0.01 |  | 100 | 0 |
| 14 | 9.86 | 16.02 | 23.94 | 24 | 16.16 | 7.3 | 2.21 | 0.45 | 0.06 | 0.01 |  | 9.86 | 16.02 | 23.94 | 24 | 16.16 | 7.3 | 2.21 | 0.45 | 0.06 | 0.01 |  | 100 | 0 |
| 15 | 11.81 | 17.11 | 24 | 22.95 | 14.97 | 6.66 | 2.02 | 0.42 | 0.06 | 0.01 |  | 13.96 | 19.3 | 25.32 | 22.1 | 12.84 | 4.96 | 1.27 | 0.22 | 0.02 | 0 |  | 100 | 0 |
| 16 | 14.1 | 18.17 | 23.91 | 21.79 | 13.75 | 6.01 | 1.82 | 0.38 | 0.06 | 0.01 |  | 16.52 | 20.28 | 24.96 | 20.76 | 11.68 | 4.44 | 1.14 | 0.2 | 0.02 | 0 |  | 100 | 0 |
| 17 | 16.32 | 19.01 | 23.71 | 20.73 | 12.71 | 5.46 | 1.65 | 0.35 | 0.05 | 0.01 |  | 18.98 | 21.03 | 24.51 | 19.56 | 10.69 | 4 | 1.02 | 0.18 | 0.02 | 0 |  | 100 | 0 |
| 18 | 18.06 | 19.55 | 23.48 | 19.94 | 11.97 | 5.08 | 1.53 | 0.32 | 0.05 | 0.01 |  | 20.89 | 21.49 | 24.11 | 18.68 | 10 | 3.7 | 0.94 | 0.17 | 0.02 | 0 |  | 100 | 0 |
| 19 | 19.21 | 19.84 | 23.28 | 19.43 | 11.54 | 4.87 | 1.46 | 0.31 | 0.05 | 0 |  | 22.3 | 21.8 | 23.82 | 18.06 | 9.5 | 3.47 | 0.88 | 0.15 | 0.02 | 0 |  | 100 | 0 |
| 20 | 19.77 | 19.92 | 23.12 | 19.18 | 11.37 | 4.81 | 1.45 | 0.31 | 0.05 | 0.01 |  | 22.9 | 21.84 | 23.61 | 17.81 | 9.36 | 3.43 | 0.88 | 0.16 | 0.02 | 0 |  | 100 | 0 |
| 21 | 19.81 | 19.78 | 22.98 | 19.16 | 11.46 | 4.91 | 1.51 | 0.33 | 0.05 | 0.01 |  | 22.91 | 21.68 | 23.48 | 17.82 | 9.47 | 3.52 | 0.92 | 0.17 | 0.02 | 0 |  | 100 | 0 |
| 22 | 19.41 | 19.44 | 22.81 | 19.3 | 11.77 | 5.18 | 1.64 | 0.38 | 0.06 | 0.01 |  | 22.43 | 21.34 | 23.38 | 18.05 | 9.81 | 3.76 | 1.01 | 0.19 | 0.03 | 0 |  | 100 | 0 |
| 23 | 18.66 | 18.9 | 22.56 | 19.55 | 12.3 | 5.62 | 1.86 | 0.45 | 0.08 | 0.01 |  | 21.55 | 20.79 | 23.24 | 18.44 | 10.39 | 4.15 | 1.18 | 0.24 | 0.03 | 0 |  | 100 | 0 |
| 24 | 17.68 | 18.19 | 22.2 | 19.84 | 12.99 | 6.23 | 2.19 | 0.56 | 0.11 | 0.02 |  | 20.4 | 20.05 | 23 | 18.92 | 11.14 | 4.7 | 1.42 | 0.31 | 0.05 | 0.01 |  | 100 | 0 |
| 25 | 16.68 | 17.42 | 21.76 | 20.09 | 13.7 | 6.91 | 2.57 | 0.71 | 0.14 | 0.02 |  | 19.31 | 19.3 | 22.71 | 19.32 | 11.89 | 5.29 | 1.7 | 0.4 | 0.07 | 0.01 |  | 100 | 0 |
| 26 | 15.84 | 16.74 | 21.32 | 20.23 | 14.3 | 7.53 | 2.96 | 0.86 | 0.19 | 0.03 |  | 18.31 | 18.58 | 22.37 | 19.65 | 12.59 | 5.89 | 2.01 | 0.5 | 0.09 | 0.01 |  | 100 | 0 |
| 27 | 15.23 | 16.22 | 20.96 | 20.3 | 14.74 | 8.02 | 3.27 | 1 | 0.23 | 0.04 |  | 17.59 | 18.04 | 22.07 | 19.84 | 13.11 | 6.36 | 2.27 | 0.59 | 0.11 | 0.02 |  | 100 | 0 |
| 28 | 14.83 | 15.88 | 20.72 | 20.33 | 15.01 | 8.34 | 3.48 | 1.1 | 0.26 | 0.05 |  | 17.12 | 17.68 | 21.87 | 19.96 | 13.44 | 6.68 | 2.45 | 0.66 | 0.13 | 0.02 |  | 100 | 0 |
| 29 | 14.59 | 15.71 | 20.6 | 20.35 | 15.16 | 8.51 | 3.6 | 1.15 | 0.27 | 0.06 |  | 16.85 | 17.49 | 21.77 | 20.03 | 13.62 | 6.84 | 2.54 | 0.7 | 0.14 | 0.02 |  | 100 | 0 |
| 30 | 14.47 | 15.67 | 20.6 | 20.4 | 15.22 | 8.55 | 3.61 | 1.15 | 0.28 | 0.06 |  | 16.85 | 17.56 | 21.85 | 20.05 | 13.58 | 6.78 | 2.5 | 0.68 | 0.14 | 0.02 |  | 100 | 0 |
| 31 | 14.4 | 15.75 | 20.74 | 20.5 | 15.2 | 8.46 | 3.53 | 1.11 | 0.26 | 0.05 |  | 16.79 | 17.66 | 21.99 | 20.12 | 13.53 | 6.68 | 2.43 | 0.65 | 0.13 | 0.02 |  | 100 | 0 |
| 32 | 14.33 | 15.94 | 21.02 | 20.66 | 15.13 | 8.26 | 3.36 | 1.02 | 0.23 | 0.04 |  | 16.75 | 17.9 | 22.28 | 20.22 | 13.39 | 6.47 | 2.28 | 0.58 | 0.11 | 0.02 |  | 100 | 0 |
| 33 | 14.25 | 16.25 | 21.45 | 20.89 | 15 | 7.94 | 3.1 | 0.89 | 0.19 | 0.03 |  | 16.72 | 18.28 | 22.71 | 20.35 | 13.15 | 6.13 | 2.06 | 0.5 | 0.09 | 0.01 |  | 100 | 0 |
| 34 | 14.14 | 16.67 | 22.04 | 21.18 | 14.79 | 7.51 | 2.77 | 0.74 | 0.14 | 0.02 |  | 16.67 | 18.78 | 23.29 | 20.5 | 12.81 | 5.68 | 1.79 | 0.4 | 0.06 | 0.01 |  | 100 | 0 |
| 35 | 14 | 17.14 | 22.7 | 21.48 | 14.52 | 7.02 | 2.42 | 0.6 | 0.1 | 0.01 |  | 16.61 | 19.36 | 23.94 | 20.64 | 12.39 | 5.19 | 1.51 | 0.31 | 0.04 | 0 |  | 100 | 0 |
| 36 | 13.87 | 17.59 | 23.33 | 21.75 | 14.24 | 6.54 | 2.11 | 0.48 | 0.08 | 0.01 |  | 16.55 | 19.92 | 24.56 | 20.73 | 11.97 | 4.73 | 1.28 | 0.24 | 0.03 | 0 |  | 100 | 0 |
| 37 | 13.77 | 17.95 | 23.83 | 21.93 | 13.98 | 6.18 | 1.89 | 0.4 | 0.06 | 0.01 |  | 16.5 | 20.36 | 25.04 | 20.77 | 11.62 | 4.38 | 1.11 | 0.19 | 0.02 | 0 |  | 100 | 0 |
| 38 | 13.7 | 18.19 | 24.17 | 22.04 | 13.8 | 5.93 | 1.75 | 0.35 | 0.05 | 0 |  | 16.47 | 20.66 | 25.36 | 20.79 | 11.37 | 4.15 | 1.01 | 0.16 | 0.02 | 0 |  | 100 | 0 |
| 39 | 13.67 | 18.32 | 24.34 | 22.1 | 13.7 | 5.8 | 1.68 | 0.33 | 0.04 | 0 |  | 16.46 | 20.82 | 25.53 | 20.79 | 11.24 | 4.03 | 0.96 | 0.15 | 0.02 | 0 |  | 100 | 0 |
| 40 | 13.69 | 18.35 | 24.37 | 22.09 | 13.68 | 5.78 | 1.67 | 0.33 | 0.04 | 0 |  | 16.65 | 20.99 | 25.6 | 20.69 | 11.07 | 3.92 | 0.92 | 0.14 | 0.02 | 0 |  | 100 | 0 |
| 41 | 13.75 | 18.28 | 24.25 | 22.04 | 13.72 | 5.86 | 1.71 | 0.34 | 0.05 | 0 |  | 16.71 | 20.9 | 25.48 | 20.66 | 11.14 | 3.99 | 0.95 | 0.15 | 0.02 | 0 |  | 100 | 0 |
| 42 | 13.88 | 18.13 | 23.99 | 21.92 | 13.82 | 6.02 | 1.81 | 0.37 | 0.05 | 0.01 |  | 16.81 | 20.7 | 25.23 | 20.61 | 11.29 | 4.15 | 1.02 | 0.17 | 0.02 | 0 |  | 100 | 0 |
| 43 | 14.06 | 17.91 | 23.62 | 21.74 | 13.96 | 6.26 | 1.96 | 0.43 | 0.06 | 0.01 |  | 16.96 | 20.4 | 24.86 | 20.54 | 11.51 | 4.38 | 1.13 | 0.2 | 0.02 | 0 |  | 100 | 0 |
| 44 | 14.29 | 17.62 | 23.14 | 21.5 | 14.13 | 6.57 | 2.16 | 0.5 | 0.08 | 0.01 |  | 17.16 | 20.03 | 24.38 | 20.43 | 11.78 | 4.67 | 1.27 | 0.24 | 0.03 | 0 |  | 100 | 0 |
| 45 | 14.53 | 17.33 | 22.66 | 21.24 | 14.28 | 6.88 | 2.38 | 0.59 | 0.1 | 0.01 |  | 17.36 | 19.65 | 23.9 | 20.3 | 12.04 | 4.98 | 1.44 | 0.29 | 0.04 | 0 |  | 100 | 0 |
| 46 | 14.74 | 17.08 | 22.24 | 21.01 | 14.39 | 7.15 | 2.57 | 0.67 | 0.13 | 0.02 |  | 17.53 | 19.32 | 23.48 | 20.17 | 12.25 | 5.26 | 1.6 | 0.34 | 0.05 | 0.01 |  | 100 | 0 |
| 47 | 14.89 | 16.89 | 21.94 | 20.84 | 14.47 | 7.34 | 2.72 | 0.74 | 0.15 | 0.02 |  | 17.66 | 19.08 | 23.17 | 20.07 | 12.4 | 5.46 | 1.71 | 0.38 | 0.06 | 0.01 |  | 100 | 0 |
| 48 | 15 | 16.76 | 21.73 | 20.71 | 14.51 | 7.48 | 2.83 | 0.79 | 0.16 | 0.03 |  | 17.75 | 18.92 | 22.96 | 19.99 | 12.49 | 5.6 | 1.8 | 0.42 | 0.07 | 0.01 |  | 100 | 0 |
| 49 | 15.1 | 16.67 | 21.58 | 20.61 | 14.54 | 7.57 | 2.91 | 0.82 | 0.17 | 0.03 |  | 17.84 | 18.8 | 22.8 | 19.93 | 12.55 | 5.7 | 1.86 | 0.44 | 0.07 | 0.01 |  | 100 | 0 |
| 50 | 15.22 | 16.6 | 21.43 | 20.5 | 14.54 | 7.65 | 2.98 | 0.86 | 0.18 | 0.03 |  | 18.03 | 18.76 | 22.67 | 19.82 | 12.54 | 5.74 | 1.9 | 0.46 | 0.08 | 0.01 |  | 100 | 0 |
| 51 | 15.43 | 16.5 | 21.22 | 20.34 | 14.54 | 7.75 | 3.08 | 0.91 | 0.2 | 0.04 |  | 18.22 | 18.62 | 22.44 | 19.7 | 12.58 | 5.85 | 1.98 | 0.49 | 0.09 | 0.01 |  | 100 | 0 |
| 52 | 15.75 | 16.36 | 20.9 | 20.08 | 14.51 | 7.89 | 3.23 | 0.99 | 0.23 | 0.04 |  | 18.54 | 18.42 | 22.11 | 19.5 | 12.64 | 6.02 | 2.11 | 0.54 | 0.1 | 0.02 |  | 100 | 0 |
| 53 | 16.22 | 16.16 | 20.46 | 19.72 | 14.47 | 8.08 | 3.44 | 1.11 | 0.27 | 0.06 |  | 18.99 | 18.14 | 21.63 | 19.21 | 12.7 | 6.26 | 2.29 | 0.63 | 0.13 | 0.02 |  | 100 | 0 |
| 54 | 16.82 | 15.9 | 19.91 | 19.26 | 14.4 | 8.31 | 3.71 | 1.28 | 0.34 | 0.08 |  | 19.57 | 17.77 | 21.03 | 18.84 | 12.77 | 6.54 | 2.54 | 0.74 | 0.16 | 0.03 |  | 100 | 0 |
| 55 | 17.46 | 15.6 | 19.32 | 18.77 | 14.3 | 8.54 | 4 | 1.47 | 0.42 | 0.12 |  | 20.25 | 17.43 | 20.43 | 18.42 | 12.76 | 6.8 | 2.78 | 0.88 | 0.21 | 0.04 |  | 100 | 0 |
| 56 | 18.03 | 15.34 | 18.81 | 18.33 | 14.19 | 8.72 | 4.26 | 1.65 | 0.51 | 0.15 |  | 20.79 | 17.07 | 19.89 | 18.06 | 12.78 | 7.05 | 3.03 | 1.01 | 0.26 | 0.06 |  | 100 | 0 |
| 57 | 18.45 | 15.14 | 18.44 | 18.01 | 14.1 | 8.85 | 4.45 | 1.8 | 0.58 | 0.19 |  | 21.19 | 16.81 | 19.49 | 17.79 | 12.78 | 7.22 | 3.21 | 1.13 | 0.31 | 0.08 |  | 100 | 0 |
| 58 | 18.73 | 15 | 18.2 | 17.8 | 14.04 | 8.93 | 4.58 | 1.89 | 0.63 | 0.21 |  | 21.45 | 16.63 | 19.23 | 17.61 | 12.77 | 7.34 | 3.34 | 1.2 | 0.34 | 0.09 |  | 100 | 0 |
| 59 | 18.88 | 14.92 | 18.06 | 17.68 | 14 | 8.97 | 4.65 | 1.95 | 0.66 | 0.23 |  | 21.59 | 16.53 | 19.08 | 17.51 | 12.77 | 7.4 | 3.41 | 1.25 | 0.36 | 0.1 |  | 100 | 0 |
| 60 | 18.93 | 14.89 | 18 | 17.63 | 13.99 | 8.99 | 4.68 | 1.98 | 0.68 | 0.24 |  | 21.7 | 16.52 | 19.04 | 17.46 | 12.74 | 7.4 | 3.42 | 1.26 | 0.37 | 0.1 |  | 100 | 0 |
| 61 | 18.9 | 14.88 | 18 | 17.64 | 14 | 9 | 4.69 | 1.98 | 0.68 | 0.24 |  | 21.67 | 16.51 | 19.04 | 17.47 | 12.75 | 7.41 | 3.42 | 1.26 | 0.37 | 0.1 |  | 100 | 0 |
| 62 | 18.8 | 14.88 | 18.03 | 17.68 | 14.03 | 9.01 | 4.68 | 1.97 | 0.67 | 0.23 |  | 21.57 | 16.53 | 19.08 | 17.51 | 12.77 | 7.41 | 3.41 | 1.25 | 0.36 | 0.1 |  | 100 | 0 |
| 63 | 18.66 | 14.9 | 18.1 | 17.76 | 14.08 | 9.01 | 4.66 | 1.95 | 0.66 | 0.23 |  | 21.42 | 16.55 | 19.15 | 17.58 | 12.81 | 7.4 | 3.39 | 1.23 | 0.36 | 0.1 |  | 100 | 0 |
| 64 | 18.48 | 14.92 | 18.17 | 17.85 | 14.13 | 9.02 | 4.64 | 1.93 | 0.64 | 0.22 |  | 21.24 | 16.59 | 19.24 | 17.67 | 12.85 | 7.39 | 3.37 | 1.21 | 0.35 | 0.09 |  | 100 | 0 |
| 65 | 18.29 | 14.94 | 18.26 | 17.95 | 14.19 | 9.03 | 4.62 | 1.9 | 0.63 | 0.21 |  | 21.14 | 16.68 | 19.37 | 17.75 | 12.84 | 7.33 | 3.3 | 1.17 | 0.33 | 0.09 |  | 100 | 0 |
| 66 | 18.13 | 14.96 | 18.33 | 18.03 | 14.24 | 9.02 | 4.59 | 1.88 | 0.62 | 0.2 |  | 20.98 | 16.72 | 19.45 | 17.83 | 12.87 | 7.32 | 3.28 | 1.15 | 0.32 | 0.08 |  | 100 | 0 |
| 67 | 18.02 | 14.98 | 18.39 | 18.09 | 14.27 | 9.02 | 4.57 | 1.86 | 0.6 | 0.2 |  | 20.86 | 16.75 | 19.52 | 17.89 | 12.89 | 7.3 | 3.25 | 1.14 | 0.31 | 0.08 |  | 100 | 0 |
| 68 | 17.93 | 14.99 | 18.42 | 18.14 | 14.3 | 9.02 | 4.56 | 1.85 | 0.6 | 0.19 |  | 20.77 | 16.76 | 19.56 | 17.93 | 12.91 | 7.3 | 3.24 | 1.13 | 0.31 | 0.08 |  | 100 | 0 |
| 69 | 17.83 | 14.97 | 18.44 | 18.17 | 14.33 | 9.05 | 4.57 | 1.85 | 0.6 | 0.19 |  | 20.66 | 16.75 | 19.58 | 17.97 | 12.95 | 7.32 | 3.25 | 1.13 | 0.31 | 0.08 |  | 100 | 0 |
| 70 | 17.65 | 14.91 | 18.42 | 18.21 | 14.41 | 9.12 | 4.62 | 1.87 | 0.61 | 0.2 |  | 20.38 | 16.64 | 19.55 | 18.04 | 13.07 | 7.43 | 3.32 | 1.16 | 0.32 | 0.08 |  | 100 | 0 |
| 71 | 17.32 | 14.77 | 18.36 | 18.27 | 14.54 | 9.26 | 4.72 | 1.92 | 0.63 | 0.2 |  | 20.01 | 16.51 | 19.52 | 18.13 | 13.22 | 7.57 | 3.41 | 1.2 | 0.33 | 0.09 |  | 100 | 0 |
| 72 | 16.78 | 14.55 | 18.26 | 18.35 | 14.76 | 9.5 | 4.9 | 2.02 | 0.67 | 0.22 |  | 19.4 | 16.28 | 19.46 | 18.28 | 13.48 | 7.81 | 3.56 | 1.27 | 0.36 | 0.1 |  | 100 | 0 |
| 73 | 16 | 14.21 | 18.1 | 18.46 | 15.08 | 9.86 | 5.16 | 2.16 | 0.73 | 0.24 |  | 18.52 | 15.94 | 19.36 | 18.48 | 13.86 | 8.18 | 3.79 | 1.38 | 0.4 | 0.11 |  | 100 | 0 |
| 74 | 15.02 | 13.76 | 17.87 | 18.59 | 15.48 | 10.33 | 5.52 | 2.36 | 0.81 | 0.28 |  | 17.4 | 15.48 | 19.19 | 18.71 | 14.35 | 8.65 | 4.1 | 1.53 | 0.45 | 0.12 |  | 100 | 0 |
| 75 | 13.98 | 13.26 | 17.59 | 18.69 | 15.91 | 10.84 | 5.92 | 2.59 | 0.91 | 0.32 |  | 16.22 | 14.96 | 18.98 | 18.95 | 14.87 | 9.19 | 4.46 | 1.71 | 0.51 | 0.15 |  | 100 | 0 |
| 76 | 13.05 | 12.79 | 17.31 | 18.76 | 16.29 | 11.32 | 6.3 | 2.81 | 1 | 0.37 |  | 15.17 | 14.48 | 18.76 | 19.13 | 15.34 | 9.68 | 4.81 | 1.88 | 0.58 | 0.17 |  | 100 | 0 |
| 77 | 12.37 | 12.43 | 17.08 | 18.8 | 16.56 | 11.68 | 6.6 | 2.99 | 1.08 | 0.4 |  | 14.4 | 14.11 | 18.58 | 19.25 | 15.69 | 10.07 | 5.08 | 2.02 | 0.63 | 0.19 |  | 100 | 0 |
| 78 | 11.93 | 12.19 | 16.93 | 18.81 | 16.74 | 11.93 | 6.8 | 3.11 | 1.14 | 0.43 |  | 13.89 | 13.85 | 18.44 | 19.32 | 15.92 | 10.32 | 5.27 | 2.11 | 0.67 | 0.2 |  | 100 | 0 |
| 79 | 11.67 | 12.05 | 16.83 | 18.82 | 16.85 | 12.07 | 6.93 | 3.18 | 1.17 | 0.44 |  | 13.6 | 13.7 | 18.36 | 19.36 | 16.05 | 10.48 | 5.38 | 2.17 | 0.69 | 0.21 |  | 100 | 0 |
| 80 | 11.55 | 11.98 | 16.78 | 18.82 | 16.9 | 12.14 | 6.98 | 3.21 | 1.19 | 0.45 |  | 13.35 | 13.54 | 18.24 | 19.36 | 16.17 | 10.63 | 5.51 | 2.25 | 0.72 | 0.22 |  | 100 | 0 |
| 81 | 11.52 | 11.96 | 16.77 | 18.82 | 16.91 | 12.16 | 7 | 3.22 | 1.19 | 0.45 |  | 13.32 | 13.52 | 18.23 | 19.36 | 16.18 | 10.66 | 5.52 | 2.25 | 0.72 | 0.23 |  | 100 | 0 |
| 82 | 11.53 | 11.97 | 16.78 | 18.82 | 16.9 | 12.15 | 6.99 | 3.22 | 1.19 | 0.45 |  | 13.33 | 13.53 | 18.24 | 19.36 | 16.18 | 10.65 | 5.52 | 2.25 | 0.72 | 0.22 |  | 100 | 0 |
| 83 | 11.57 | 11.99 | 16.79 | 18.82 | 16.89 | 12.13 | 6.97 | 3.21 | 1.18 | 0.45 |  | 13.37 | 13.55 | 18.25 | 19.35 | 16.16 | 10.62 | 5.5 | 2.24 | 0.72 | 0.22 |  | 100 | 0 |
| 84 | 11.61 | 12.01 | 16.8 | 18.82 | 16.87 | 12.11 | 6.96 | 3.2 | 1.18 | 0.45 |  | 13.42 | 13.58 | 18.26 | 19.35 | 16.14 | 10.6 | 5.48 | 2.23 | 0.72 | 0.22 |  | 100 | 0 |
| 85 | 11.64 | 12.03 | 16.82 | 18.82 | 16.86 | 12.09 | 6.94 | 3.19 | 1.17 | 0.44 |  | 13.41 | 13.55 | 18.24 | 19.33 | 16.14 | 10.62 | 5.5 | 2.25 | 0.72 | 0.23 |  | 100 | 0 |
| 86 | 11.67 | 12.05 | 16.83 | 18.82 | 16.85 | 12.07 | 6.93 | 3.18 | 1.17 | 0.44 |  | 13.44 | 13.57 | 18.25 | 19.33 | 16.13 | 10.61 | 5.49 | 2.24 | 0.72 | 0.22 |  | 100 | 0 |
| 87 | 11.69 | 12.05 | 16.84 | 18.82 | 16.84 | 12.06 | 6.92 | 3.17 | 1.17 | 0.44 |  | 13.46 | 13.58 | 18.25 | 19.33 | 16.12 | 10.6 | 5.49 | 2.24 | 0.72 | 0.22 |  | 100 | 0 |
| 88 | 11.7 | 12.06 | 16.84 | 18.82 | 16.84 | 12.06 | 6.91 | 3.17 | 1.17 | 0.44 |  | 13.47 | 13.58 | 18.26 | 19.33 | 16.12 | 10.59 | 5.48 | 2.23 | 0.72 | 0.22 |  | 100 | 0 |
| 89 | 11.7 | 12.06 | 16.84 | 18.82 | 16.84 | 12.06 | 6.91 | 3.17 | 1.16 | 0.44 |  | 13.47 | 13.59 | 18.26 | 19.33 | 16.12 | 10.59 | 5.48 | 2.23 | 0.72 | 0.22 |  | 100 | 0 |
| 90 | 11.7 | 12.06 | 16.84 | 18.82 | 16.84 | 12.05 | 6.91 | 3.17 | 1.16 | 0.44 |  | 13.47 | 13.59 | 18.26 | 19.32 | 16.11 | 10.59 | 5.48 | 2.23 | 0.72 | 0.22 |  | 100 | 0 |
| 91 | 11.7 | 12.06 | 16.84 | 18.82 | 16.84 | 12.06 | 6.91 | 3.17 | 1.16 | 0.44 |  | 13.47 | 13.59 | 18.26 | 19.33 | 16.12 | 10.59 | 5.48 | 2.23 | 0.72 | 0.22 |  | 100 | 0 |
| 92 | 11.7 | 12.06 | 16.84 | 18.82 | 16.84 | 12.06 | 6.91 | 3.17 | 1.17 | 0.44 |  | 13.47 | 13.59 | 18.26 | 19.33 | 16.12 | 10.59 | 5.48 | 2.23 | 0.72 | 0.22 |  | 100 | 0 |
| 93 | 11.7 | 12.06 | 16.84 | 18.82 | 16.84 | 12.06 | 6.91 | 3.17 | 1.17 | 0.44 |  | 13.47 | 13.59 | 18.26 | 19.33 | 16.12 | 10.59 | 5.48 | 2.23 | 0.72 | 0.22 |  | 100 | 0 |
| 94 | 11.69 | 12.06 | 16.84 | 18.82 | 16.84 | 12.06 | 6.91 | 3.17 | 1.17 | 0.44 |  | 13.47 | 13.58 | 18.25 | 19.33 | 16.12 | 10.59 | 5.48 | 2.23 | 0.72 | 0.22 |  | 100 | 0 |
| 95 | 11.69 | 12.06 | 16.84 | 18.82 | 16.84 | 12.06 | 6.91 | 3.17 | 1.17 | 0.44 |  | 13.46 | 13.58 | 18.25 | 19.33 | 16.12 | 10.59 | 5.48 | 2.23 | 0.72 | 0.22 |  | 100 | 0 |

^a^ Rounded to two decimal points

^b^ %E = Percent of total energy intake
