# Supplementary material for: Substituting polyunsaturated fat for saturated fat: A health impact assessment of a fat tax in seven European countries
Source: PLoS One. 2019 Jul 10;14(7):e0218464. doi: 10.1371/journal.pone.0218464 (PMC6619676; doi:10.1371/journal.pone.0218464)
Supplement: S17 Table — (DOCX) [file pone.0218464.s017.docx]

S17 Table. Population size and deaths postponed in projection year 10.

| Coun­try^a^ | Scenario | Males | | | Females | | |
| --- | --- | --- | --- | --- | --- | --- | --- |
|  |  | Population size^b^ | Deaths postponed^c^ | % of guideline scenario^d^ | Population size^b^ | Deaths postponed^c^ | % of guideline scenario^d^ |
| DK | Reference scenario | 2,703,591 | - | - | 2,757,179 | - | - |
|  | Fat tax scenario | 2,703,656 | 65 | 12.72% | 2,757,218 | 39 | 12.90% |
|  | Guideline scenario | 2,704,102 | 511 | 100.00% | 2,757,480 | 301 | 100.00% |
| IT | Reference scenario | 27,586,010 | - | - | 29,251,524 | - | - |
|  | Fat tax scenario | 27,586,407 | 397 | 25.83% | 29,251,750 | 226 | 31.35% |
|  | Guideline scenario | 27,587,548 | 1,538 | 100.00% | 29,252,245 | 721 | 100.00% |
| PL | Reference scenario | 18,098,128 | - | - | 19,574,866 | - | - |
|  | Fat tax scenario | 18,098,770 | 642 | 15.09% | 19,575,198 | 332 | 14.72% |
|  | Guideline scenario | 18,102,381 | 4,252 | 100.00% | 19,577,123 | 2,257 | 100.00% |
| ES | Reference scenario | 20,755,665 | - | - | 21,638,814 | - | - |
|  | Fat tax scenario | 20,755,930 | 265 | 25.54% | 21,638,969 | 156 | 29.24% |
|  | Guideline scenario | 20,756,703 | 1,038 | 100.00% | 21,639,346 | 532 | 100.00% |
| SE | Reference scenario | 4,564,385 | - | - | 4,625,358 | - | - |
|  | Fat tax scenario | 4,564,515 | 130 | 11.11% | 4,625,445 | 86 | 12.57% |
|  | Guideline scenario | 4,565,553 | 1,168 | 100.00% | 4,626,046 | 688 | 100.00% |
| NL | Reference scenario | 8,294,049 | - | - | 8,453,579 | - | - |
|  | Fat tax scenario | 8,294,304 | 255 | 11.10% | 8,453,721 | 143 | 10.34% |
|  | Guideline scenario | 8,296,347 | 2,298 | 100.00% | 8,454,958 | 1,379 | 100.00% |
| UK | Reference scenario | 29,858,542 | - | - | 30,963,890 | - | - |
|  | Fat tax scenario | 29,859,335 | 793 | 11.62% | 30,964,439 | 549 | 10.80% |
|  | Guideline scenario | 29,865,373 | 6,831 | 100.00% | 30,968,973 | 5,083 | 100.00% |

^a^ DK=Denmark, IT=Italy, PL=Poland, ES= Spain, SE=Sweden, NL=The Netherlands, UK= United Kingdom

^b^ Rounded to nearest full number

^c^ Deaths postponed= Absolute difference of population size in fat tax and guideline scenario compared to reference scenario.

^d^ % of guideline scenario= Deaths postponed in fat tax scenario measured against deaths postponed in guideline scenario
