# Supplementary material for: Substituting polyunsaturated fat for saturated fat: A health impact assessment of a fat tax in seven European countries
Source: PLoS One. 2019 Jul 10;14(7):e0218464. doi: 10.1371/journal.pone.0218464 (PMC6619676; doi:10.1371/journal.pone.0218464)
Supplement: S1 Text — (DOCX) [file pone.0218464.s018.docx]

# S1 Text. Calculating relative risks of ischemic heart disease by intake category of saturated fat.

Relative risks relating each 5%E of increased saturated fat (SFA), replacing polyunsaturated fat (PUFA), with ischemic heart disease (IHD), were taken from the GBD study 2016 [1].

In order to obtain relative risk relating matching our ten intake categories (≤10%E, >10≤12%, >12 ≤14%E, >14 ≤16%E, >16 ≤18%E, >18 ≤20%E, >20 ≤22%E, >22 ≤24%E, >24 ≤26%E, >26 ≤100%E), we transformed these relative risks according to the following steps:

1. Our reference category with a SFA intake of 0–10%E was assigned a relative risks of 1.

2. We calculated the log relative risk per unit change of SFA intake (in percent of total energy intake (%E)) with $\beta=\frac{\ln{RR}_{5}}{5}$, where RR_5_ is the relative risk for a 5%E of increased SFA intake/day.

3. We defined the midpoint x_i_ for each of the remaining nine SFA intake categories, i.e. 11%E, 13%E, 15%E, 17%E, 19%E, 21%E, 23%E, 25%E, and 27%E.

4. We calculated the relative risks for each of the category specific midpoints with ${RR}_{i}=\exp\left( \beta*(x_{i}-10) \right)$, where x_i_ is defined to be the SFA intake category specific midpoint and the value of 10 corresponds to the upper bound of the reference category, because we assume that the relative risks are only increasing from an SFA intake of 10%E onwards.

**References**

1. GBD 2016 Risk Factor Collaborators. Global, regional, and national comparative risk assessment of 84 behavioural, environmental and occupational, and metabolic risks or clusters of risks, 1990–2016: a systematic analysis for the Global Burden of Disease Study 2016. Lancet. 2017;390(10100):1345-422.
