# Supplementary material for: Substituting polyunsaturated fat for saturated fat: A health impact assessment of a fat tax in seven European countries
Source: PLoS One. 2019 Jul 10;14(7):e0218464. doi: 10.1371/journal.pone.0218464 (PMC6619676; doi:10.1371/journal.pone.0218464)
Supplement: S2 File — (DOCX) [file pone.0218464.s020.docx]

**S2 File. Excess number of persons being alive in Denmark under the guideline scenario compared to the reference scenario.**
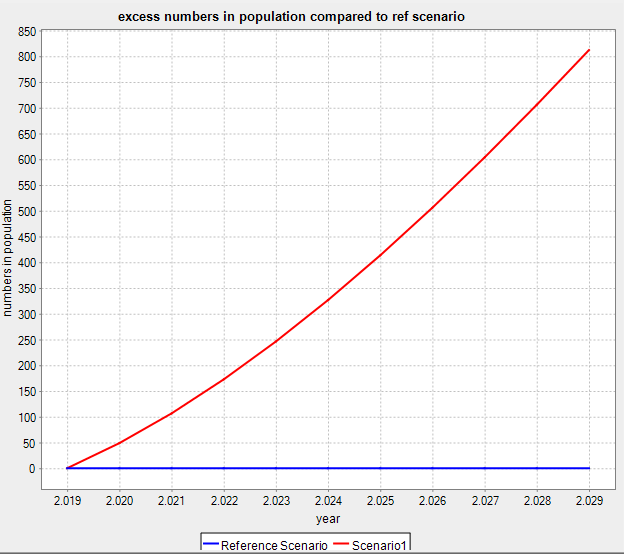


Scenario 1 = Guideline scenario
